# Coordinate Transcriptomic and Metabolomic Effects of the Insulin Sensitizer Rosiglitazone on Fundamental Metabolic Pathways in Liver, Soleus Muscle, and Adipose Tissue in Diabetic db/db Mice

**DOI:** 10.1155/2010/679184

**Published:** 2010-10-10

**Authors:** Sabrina Le Bouter, Marianne Rodriguez, Nolwen Guigal-Stephan, Sophie Courtade-Gaïani, Laura Xuereb, Catherine de Montrion, Vincent Croixmarie, Thierry Umbdenstock, Claire Boursier-Neyret, Michel Lonchampt, Manuel Brun, Catherine Dacquet, Alain Ktorza, Brian-Paul Lockhart, Jean-Pierre Galizzi

**Affiliations:** ^1^Division of Molecular Pharmacology and Pathophysiology, Institut de Recherches Servier, 125 Chemin de Ronde, 78290 Croissy-sur-Seine, France; ^2^Department of Biostatistics, Institut de Recherches Servier, 11 rue des Moulineaux, 92150 Suresnes, France; ^3^Metabolisme BA, Technologie Servier, 5 rue Bel Air, 45000 Orleans, France; ^4^Division of Diabetes Metabolic Diseases Research, Institut de Recherches Servier, 11 rue des Moulineaux, 92150 Suresnes, France

## Abstract

Rosiglitazone (RSG), developed for the treatment of type 2 diabetes mellitus, is known to have potent effects on carbohydrate and lipid metabolism leading to the improvement of insulin sensitivity in target tissues. To further assess the capacity of RSG to normalize gene expression in insulin-sensitive tissues, we compared groups of 18-day-treated db/db mice with increasing oral doses of RSG (10, 30, and 100 mg/kg/d) with untreated non-diabetic littermates (db/+). For this aim, transcriptional changes were measured in liver, inguinal adipose tissue (IAT) and soleus muscle using microarrays and real-time PCR. In parallel, targeted metabolomic assessment of lipids (triglycerides (TGs) and free fatty acids (FFAs)) in plasma and tissues was performed by UPLC-MS methods. Multivariate analyses revealed a relationship between the differential gene expressions in liver and liver trioleate content and between blood glucose levels and a combination of differentially expressed genes measured in liver, IAT, and muscle. In summary, we have integrated gene expression and targeted metabolomic data to present a comprehensive overview of RSG-induced changes in a diabetes mouse model and improved the molecular understanding of how RSG ameliorates diabetes through its effect on the major insulin-sensitive tissues.

## 1. Introduction

Type 2 diabetes, also known as non-insulin-dependent diabetes mellitus, is a chronic disease that affects more than 100 million people worldwide, and its prevalence is soaring in western countries driven by high-fat diets and sedentary lifestyles. This pathology is characterized by resistance to the effects of insulin in peripheral tissues, which is manifested as reduced insulin-stimulated glucose uptake into skeletal muscle and adipose tissue, defective insulin-dependent suppression of hepatic glucose output, and reduced insulin secretion from pancreatic *β*-cells. Insulin-sensitising drugs, such as RSG, are widely used in clinical practice to improve diabetes alteration in glucose metabolism. Thiazolidinediones (TZDs) are already known to decrease blood glucose concentration, to improve liver and muscle insulin sensitivity [1, 2], and to have significant impact on adipose tissue by inducing adipose differentiation, lipogenesis, and TG storage [3–6]. The mechanisms of action of TZDs are mediated through binding and activation of the peroxisome proliferator-activated receptor *γ* (PPAR*γ*), a nuclear receptor that has a regulatory role in lipid metabolism and in cell differentiation, particularly in adipocytes [7, 8]. PPAR*γ* is also expressed in several other tissues, including muscle, liver, pancreas, heart, and spleen [9]. Among the multiple actions of TZDs is the normalisation of blood glucose level, by increasing glucose uptake and decreasing hepatic glucose production [10]. Moreover, TZDs induce shifts in systemic lipid profiles, with a decrease in FFAs and TGs levels in the circulation, partly by improving adipocyte function [11]. Taken together, these effects on carbohydrate and lipid metabolism are associated with the improvement of insulin sensitivity of peripheral tissues. However, TZDs actions are also accompanied by increased adipogenesis and lipid accumulation in tissues [12, 13]. RSG is a prototype of the TZD chemical class developed for the treatment of type 2 diabetes mellitus, and some of these effects are known to be mediated via gene transcriptional regulation [14–17]; however, the relationship between the RSG-mediated gene expression regulation in insulin-dependent tissues and the subsequent physiological changes remains unclear.

Diabetic db/db mice, which have a defect in the leptin receptor, reproduce many of the metabolic disturbances present in patients with type II diabetes mellitus, including hyperglycaemia, hyperinsulinemia, hyperlipidemia, and insulin resistance [18]. To address the question of whether RSG-mediated normalisation of blood and tissue biological characteristics in treated db/db is accompanied by normalized gene expression in three major insulin-dependent tissues, liver, IAT (inguinal adipose tissue), and skeletal muscle, we analysed transcriptomic profiles of RSG-treated diabetic mice db/db and compared them with those of untreated non diabetic littermates db/+. Multivariate analysis was then used to establish relationships between the RSG-regulated gene expression profiles and metabolomic data obtained from the insulin-sensitive tissues or blood samples. 

This study provides a comprehensive evaluation of RSG-induced changes in genome-wide expression and its relationships with RSG-mediated physiological changes in three main insulin-sensitive tissues known to be involved in lipid and glucose homeostasis.

## 2. Materials and Methods

### 2.1. Animals and Treatment

An eight-week old male BKS- Cg-*m*+/+ Lepr*db/J* diabetic (db/db) mice and their nondiabetic strain (db/+) were obtained from Charles River Laboratories (L'Arbresle, France). Three animals were housed per cage and acclimatised for 1 week under standard light (12 h light/dark), feeding (db/+: standard laboratory chow (A03, Safe, France); db/db: 5K52, Safe, France). Mice were randomly divided into 5 different groups (*n* = 6/group) as follows: vehicle- (1% HEC-) treated nondiabetic db/+ and diabetic db/db mice (control db/db mice), and diabetic db/db mice treated orally with the PPAR*γ* agonist RSG (Syntheval, Caen, France) at 3 separate doses (10, 30, 100 mg/kg/day) for 18 days. Body weight was measured daily, and blood was collected by orbital sinus puncture before and after treatment (18h after the last treatment) to assess glucose levels. All experimental procedures were in accordance with the International European Ethical Standards (86/609-EEC) and the French National Committee (decree 87/848) for the care and use of laboratory animals.

### 2.2. Tissue and Blood Analyses

At the end of treatment, mice were euthanized by cervical dislocation. Liver, IAT, and soleus muscle were immediately taken, weighed, flash frozen in liquid nitrogen, and kept at –80°C. Blood samples were drawn into heparin-containing tubes (1.5 I.U. heparin for 100 *μ*L of blood), and plasma was aliquoted and stored at 4°C. Blood glucose, Hb, and HbA1c levels were determined using the automatic analyser COBAS system (Roche, Basel, Switzerland). FFA and TG Liver and adipose tissues (50 mg aliquots) were extracted with CHCl_3_. After evaporation, the organic dry extract was reconstituted in CH_3_CN/iPrOH (1/1). Plasma (50 ***μ***L) was extracted with a mixture of CHCl_3_/MeOH (2/1). FFA and TG analyses were performed by UPLC-MS using a C18 BEH Acquity analytical column, a gradient of CH_3_CN, iPrOH, and formic acid (0.1%) (0.5 mL/min, 50°C), and a 4000 QTRAP (Applied Biosystems, Foster City, CA, USA) and LCT premier (Waters).

### 2.3. RNA Preparation

Total RNA was prepared from frozen liver, soleus, and IAT, using Trizol reagent (Invitrogen, France) and was further purified by ammonium acetate precipitation according to standard protocols. Purified total RNA concentration and 260/280 nm or 260/230 nm ratios were determined using a Nanodrop ND-1000 spectrophotometer (Nyxor Biotech, France). Then, total RNA integrity was checked by microelectrophoresis on acrylamide gel (Agilent 2100 Bioanalyzer, Agilent, Santa Clara, CA, USA).

### 2.4. Microarray Hybridization and Data Analysis

Total RNA (500 ng) samples were labelled with cyanine Cy5- or Cy3-CTP dyes using Agilent Low RNA Input Linear Amplification Kit (Agilent Technology, PN 5184-3523), according to the manufacturer's protocols. Following in vitro transcription, 825 ng of test sample (individual db/+: *n* = 6) or RSG-treated db/db cRNA were mixed with 825 ng of reference sample (pooled: *n* = 6 db/db cRNA). The samples were hybridized (dye-swap replicates) on 4 × 44 k whole-Genome 60-mer oligo-microarrays (Agilent technologies, PN G4122F) for 17 hours at 65°C. Microarrays were scanned using a dynamic auto focus microarray scanner (Agilent technology). Raw data were normalised using local background subtraction and local Lowess dye normalisation. Data were analysed using Rosetta Resolver Gene Expression Analysis System v6.0 (Rosetta Inpharmatics LLC, Seattle, WA). Dye-swap replicates were precombined before any statistical analysis. For each group, statistically significant regulated sequences were defined as the sequences whose expression in the db/+ or in the RSG-treated db/db samples was statistically different from the expression in the untreated db/db (reference), as calculated by Rosetta error-weighted model [19]. For this study a *P*-value < .001 was considered to be significant, and a fold-change value cutoff was ≥|1.3|. Ingenuity Pathway Analysis application (Ingenuity Systems, CA, USA) was used to allow functional interpretation of the data. Promoter gene sequences were investigated for the presence of proximal (~500 bp from initiation site) PPAR response element (PPRE) in their promoters with MatInspector (Genomatix; GmbH) [20]. The partial least-squares (PLSs) projection to latent structure analysis [21] was performed using Simca-P12 software (Umetrics, Umea, Sweden).

### 2.5. Real-Time RT-PCR

Total RNA (1 *μ*g) from each animal (*n* = 6/group) and from pooled reference db/db was reverse transcribed into cDNA using a transcription kit (Applied Biosystems, PN 4368813). PCR reactions were then proceeded in microfluidic cards using the ABI PRISM 7900HT Sequence Detection System (Applied Biosystems). Each microfluidic card was preloaded with predesigned Taqman probes and primers for genes and RNA 18S reference gene. cDNA (100 ng) was mixed with 2X Taqman Universal PCR master Mix (Applied Biosystems, PN 4324020) and loaded in each well (*n* = 3). The following temperature profile was used: 10 min at 94.5°C, followed by 40 cycles of 97°C for 30 sec and 59.7°C for 1 min. Normalised data with RNA 18S were analysed using the threshold cycle (*Ct*) relative quantification method [22], and the ΔΔCt method was used to compare the amounts of RNA in test and reference groups.

### 2.6. Immunocytochemistry

Adipose tissue was fixed in 4% neutral buffered formaldehyde, and cryostat sections of 7 *μ*m were prepared. The labelling was performed using a Discovery XT automat (Ventana Medical Systems, Tucson, USA). Sections were incubated 1h30 at 37°C with Alexa fluor 488-conjugated anti-OxPhos Complex IV subunit I antibody (1/100 dilution, Molecular Probes, PN A21296). Immunolabelling was visualized and imaged using an LSM 510 confocal microscope (Carl Zeiss SAS, France).

## 3. Results

### 3.1. Effects of RSG on Physiological Characteristics

At the beginning of the experiment the RSG-treated and control db/db groups of mice were comparably obese and diabetic. Blood glucose concentrations and body weights for these two groups were similar and were both significantly different from db/+ nondiabetic mice (Table 1). Eighteen days of dose-dependent treatment with RSG resulted in a reversion of hyperglycaemia. Indeed, a reduction of blood glucose concentrations and HbA1c were observed for all RSG doses tested. Blood glucose normalisation was obtained from 30 mg/kg (0.98 ± 0.12 versus 1.1 ± 0.1 g/L in db/+); HbA1c levels from db/db-treated mice were all significantly above the db/+ control mice, showing only a partial normalisation of this parameter (Table 1).

### 3.2. Targeted Metabolomic Assessment of Plasma and Tissue Lipids

Diabetic db/db mice displayed higher plasma trioleate concentrations than db/+. RSG-treated db/db mice showed a clear decrease in the levels of the two principal plasma TGs, trilinoleate and trioleate. When compared to db/+, TG normalisation was achieved from 10 mg/kg RSG treatment (16.8 ± 6.5 versus 26.8 ± 10.2 nmol/mL and 6.7 ± 1.6 versus 11.8 ± 3.4 nmol/mL for trilinoleate and trioleate, respectively). Plasma FFA concentrations showed a tendency to be higher in db/db than db/+ mice, but the difference remained significant only for linoleic acid. RSG treatment further decreased plasma FFA concentrations compared to both db/db and db/+, but in this latter case it did not achieve the significance threshold (Table 1). 

In the liver of diabetic db/db mice, both TG and FFA levels reached a higher concentration compared to db/+. From 10 mg/kg the RSG treatment of db/db further increased trioleate and oleic and palmitic acids. In parallel, the liver weight of RSG-treated db/db mice increased (+86%, +64%, and +46% at 10, 30, and 100 mg/kg RSG, resp.) versus the control db/db mice. 

As expected, IAT weight was higher in both RSG-treated and control db/db mice compared to the db/+ mice (2.10 ± 0.06 in untreated db/db mice; 1.99 ± 0.11, 2.09 ± 0.13, and 2.16 ± 0.09 g in treated db/db mice at 10, 30, and 100 mg/kg RSG, respectively *versus *0.17 ± 0.02 g in db/+ mice). In contrast, the TG concentrations relative to adipose tissue mass in db/+ were markedly higher than in RSG-treated or untreated db/db. FFA adipose tissue concentrations remained basically unchanged either between db/db and db/+ or following RSG treatment except for oleic acid the levels of which in db/db with or without RSG treatment far exceeded those of db/+.

Soleus muscle weight was unaltered by both pathology and treatment (Table 1), although fat accumulation was observed for control db/db versus RSG-treated db/db during the soleus muscle dissection.

### 3.3. Transcriptomic Profile of the Insulin-Sensitive Tissues of db/+ and RSG-Treated db/db

Changes in global gene expression were assessed in three insulin-sensitive tissues, liver, IAT, and soleus muscle, involved in glucose and lipid metabolism using whole-mouse genome microarrays. Differences in gene expression between db/+ or RSG-treated db/db versus the control db/db group were filtered according to a *P*-value < .001 and a fold change ≥|1.3| (Rosetta error- weighted model [19]). One gene could be represented by different gene (probes) sequences. Gene alteration between nondiabetic db/+ versus diabetic db/db mice (see Supplementary Table 1 in material available online at doi: 10.1155/2010/679184) was determined in the liver (6539 regulated sequences), in the soleus muscle (7895 regulated sequences), and in the IAT (13207 regulated sequences). RSG treatment of db/db mice elicited significant changes in the expression of a comparable number of genes to that observed between db/+ and the diabetic control db/db reference group, namely in the liver (5567, 4788, and 4549 regulated sequences at 10, 30, and 100 mg/kg RSG, resp.), in the IAT (5710, 9005, and 10335 regulated sequences at 10, 30, and 100 mg/kg RSG, resp.), and in the soleus muscle (3413, 3644, and 3620 regulated sequences at 10, 30, and 100 mg/kg RSG, resp.).

### 3.4. Functional Interpretation of the Changes in Transcriptomic Profiles

To interpret the biological alteration, differentially expressed genes (db/+ or RSG-treated db/db versus control db/db reference group, Supplemental Table 1) with known gene symbol (HUGO) were submitted to Ingenuity Pathway analysis. Each gene symbol was mapped to its corresponding gene in the Ingenuity Pathways Knowledge database, and biological functions and diseases were assigned to the pattern of gene expression. Functions were listed from most significant to least, and the horizontal line shows the cutoff value for significance (*P* < .05, adjusted Benjamini-Hochberg; Supplemental Figure 1). As expected, either in the nondiabetic db/+ (diabetes effect given as reciprocal (db/+ versus db/db), see below) or in the RSG-treated db/db mice, the changes in expression mainly involved genes related to lipid and carbohydrate metabolism not only in the liver but also in the IAT and the soleus muscle (Supplemental Figure 1). Moreover, groups of genes were also enriched in several functions related to cell signalling, movement, or development in liver, adipose tissue, and soleus muscle. These results could be explained by diabetes-induced morphological and/or growth alteration of hepatocytes, adipocytes, and myocytes in db/db mice as well as in db/db under RSG treatment. However, in the attempt to link transcriptomic and metabolomic data we focussed our study on genes involved in lipid and carbohydrate metabolism and potentially associated with diabetes. This list included 506 gene sequences involved in glycolysis, gluconeogenesis, TG and FFA metabolism, and pentose phosphate synthesis, as well as genes involved in mitochondrial functions, that is, *β*-oxidation, citrate cycle, and oxidative phosphorylation. Thereafter, to facilitate direct comparison with RSG treatment effect, we used the reciprocal form of the diabetes effect, that is, untreated nondiabetic db/+ versus diabetic mice db/db. Consequently, RSG-induced normalisation of gene expression is defined as genes that were significantly changed by RSG (RSG-treated db/db versus db/db) in the same direction as nondiabetic (db/+ versus db/db) mice.

### 3.5. Effect of RSG on the Liver Metabolic-Related mRNA Expression

**Figure 1:**
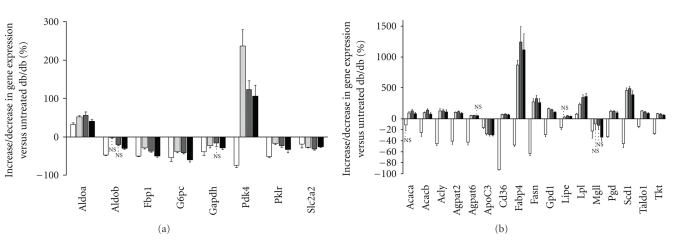
Expression profiles of key genes involved in liver glucose and lipid metabolism. Differentially expressed genes in db/+ (the white square) as well as 10 (the off-white square), 30 (the grey square), and 100 (the black square)  mg/kg RSG-treated db/db mice were measured *versus* untreated db/db and plotted as the mean (% increase/decrease of db/db control) ±SEM (*n* = 6). NS; *P* ≥ .001 otherwise *P* < .001. (a) Genes related to glucose metabolism: Aldoa, Aldob, aldolase 1A, 2B; Fbp1, fructose bisphosphatase 1; G6pc, glucose-6-phosphatase, catalytic; Gapdh, glyceraldehyde-3-phosphate dehydrogenase; Pdk4, pyruvate dehydrogenase kinase, isoenzyme 4; Pklr, pyruvate kinase liver and red blood cell; Slc2a2, solute carrier family 2 (facilitated glucose transporter), member 2. (b) Genes related to FA transport, FA and TG synthesis, and pentose pathway: Acaca, Acacb, acetyl-Coenzyme A carboxylase alpha, beta; Acly, ATP citrate lyase; Agpat2, Agpat6, 1-acylglycerol-3-phosphate O-acyltransferase 2, 6; Apoc3, apolipoprotein C-III; Cd36, CD36 antigen; Fabp2, fatty acid-binding protein 2, intestinal; Fabp4, fatty acid-binding protein 4, adipocyte; Fasn, fatty acid synthase; Gpd1, glycerol-3-phosphate dehydrogenase 1 (soluble); Lpl, lipoprotein lipase; Mgll, monoglyceride lipase; Pgd, phosphogluconate dehydrogenase; Scd1, stearoyl-Coenzyme A desaturase 1; Taldo1, transaldolase 1; Tkt, transketolase.

**Figure 2:**
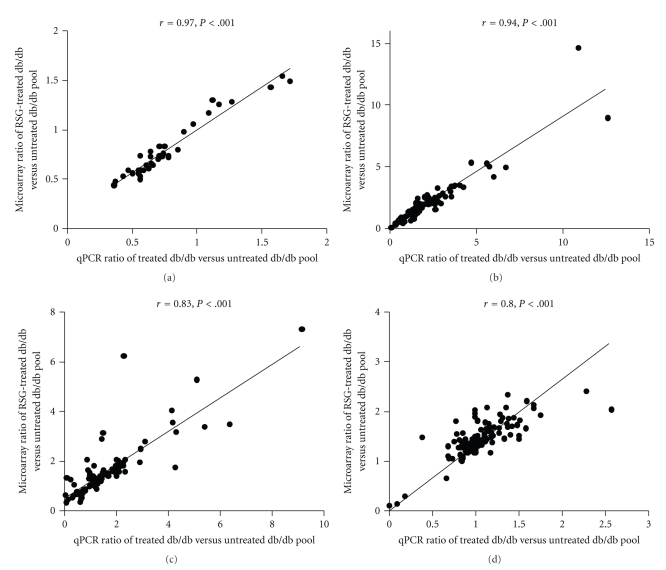
Correlation plot between microarray and qPCR data. Genes involved in (a) liver carbohydrate metabolism, (b) liver FA and TG metabolism, (c) IAT FA and TG metabolism, and (d) IAT citrate cycle and oxidative phosphorylation. Microarray ratio of differentially expressed genes in db/+ and RSG-treated db/db *versus* untreated db/db mice were plotted on the *y*-axis and qPCR ratio data on the *x*-axis. Correlations were assessed using Pearson's correlation coefficient (*r*) and *P*-value <.001.

In the liver among the 506 gene sequences 276 were differentially expressed in either db/+ versus db/db (Supplemental Table 2) or RSG-treated db/db versus db/db. Supplemental Figure 2(a) displays the microarray data overview of differentially expressed genes between lean db/+ or RSG-treated mice and the diabetic db/db strain involved in glucose metabolism. As shown in Figure 1(a), remarkably, RSG treatment resulted in a partial or complete normalisation in mRNA expression of genes encoding for glycolysis/gluconeogenesis key enzymes such as Glut-2, Gapdh, Pklr or Fbp1, and G6pc. These effects were particularly pronounced at 100 mg/kg of RSG. The only exception is Pdk4 mRNA expression which is downregulated in db/+ mice but increased in RSG-treated db/db mice when compared to the control db/db. Real-time PCR was used to confirm the differential expression of genes involved in glucose metabolism (Supplemental Table 3). Figure 2(a) shows that microarray ratio measurements were strongly correlated to that obtained by PCR (*r* = 0.97; *P* < .001; number of xy pairs = 23).

Contrary to gluconeogenesis-related genes, which seemed to be normalised by RSG treatment, microarray data overview shows that a large number of genes involved in lipid metabolism was regulated in the opposite direction to db/+ (Supplemental Figures 2(b) and 1(b)). This included genes that were downregulated in db/+ but were all markedly upregulated in RSG-treated mice when compared to db/db. For example, at 10 mg/kg RSG-mediated upregulation of genes implicated in lipid metabolism synthesis (Figure 1(b)), such as the lipid transporters Cd36 (+62%, *P* < .001), the fatty acid binding protein Fabp4 (+870%, *P* < .001), the key enzymes in FFA and TG synthesis such as the ATP citrate lyase (Acly, +124%, *P* < .001), the acetyl-CoA-carboxylase (Acaca, +87%, *P* < .001), the fatty acid synthase (FAS, +270%, *P* < .001), and the stearoyl-Coenzyme A desaturase (Scd1, +455%, *P* < .001) or was involved in the pentose phosphate pathway such as the transketolase (Tkt, +74%, *P* < .001) and phosphogluconate dehydrogenase (Pgd, +117%, *P* < .001). All these results were confirmed by real-time qPCR with a very strong correlation (Supplemental Table 3 and Figure 2(b); *r* = 0.94, *P* < .001; number of xy pairs = 155). The increased expression of genes encoding lipogenic enzymes and the pentose phosphate pathway could account for the increase in liver TG, FFA content, and weight liver (Table 1).

### 3.6. Effect of RSG on IAT Metabolic-Related mRNA Expression

**Figure 3:**
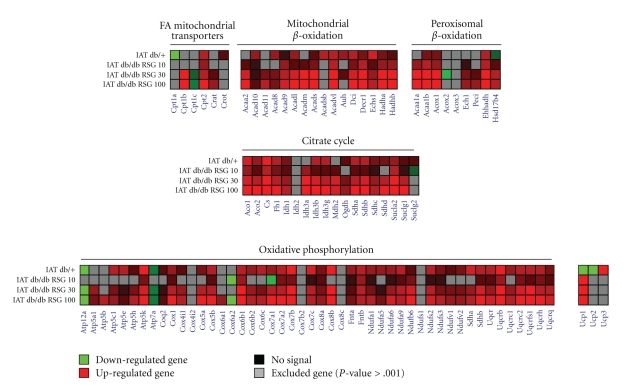
Two-dimensional representation of differentially expressed genes involved in IAT mitochondrial and peroxisomal functions. Ratio for differentially expressed genes in db/+ and RSG-treated db/db *versus* untreated db/db were plotted. Each coloured box represents differential expression ratio ranging from bright green (lowest) to bright red (highest). Missing value are in grey when *P*-value ≥ .001. Genes involved in mitochondrial transport of FA, in mitochondrial and peroxisomal *β*-oxidation and citrate cycle and in oxidative phosphorylation were shown.

In adipose tissue, among the 506 gene sequences 391 were differentially expressed in either db/+ versus db/db or RSG-treated db/db versus db/db (Supplemental Table 2). Numerous upregulated genes in db/+ or RSG-treated db/db relative to db/db diabetic mice (Supplemental Figure 3 and Figure 3) potentially stimulate lipid metabolism and mitochondrial functions. 

**Figure 4:**
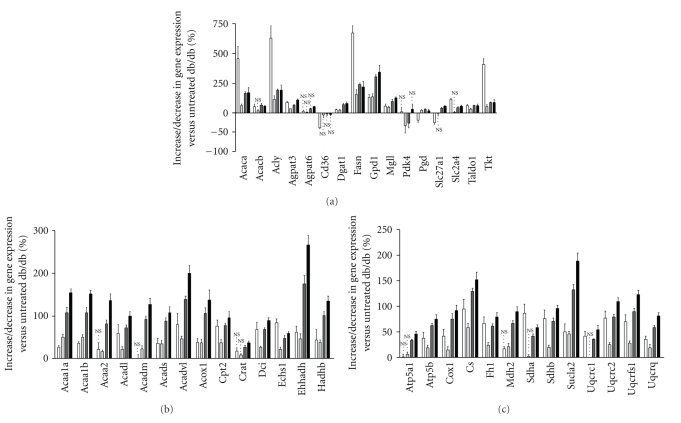
Expression profiles of key genes involved in IAT FA metabolism. Differentially expressed genes in db/+ (the white square) as well as 10 (the off-white square), 30 (the grey square), and 100 (the black square)  mg/kg RSG-treated db/db mice were measured *versus* untreated db/db and plotted as the mean (% increase/decrease of db/db control) ±SEM (*n* = 6). NS; *P* ≥ .001 otherwise *P* < .001. (a) Genes related to FA transport, glucose, FA and TG synthesis and pentose pathway*: Agpat3, 1-acylglycerol-3-phosphate O-acyltransferase 3; Dgat1, diacylglycerol O-acyltransferase 1; Slc27a1, solute carrier family 27 (fatty acid transporter), member 1; Slc2a4, solute carrier family 2 (facilitated glucose transporter), member 4. (b) Genes related to mitochondrial and peroxisomal *β*-oxidation: Acaa1a, Acaa1b, acetyl-Coenzyme A acyltransferase 1A, 1B; Acaa2, acetyl-Coenzyme A acyltransferase 2; Acadl, acyl-Coenzyme A dehydrogenase, long chain; Acadm, acyl-Coenzyme A dehydrogenase, medium chain; Acads, acyl-Coenzyme A dehydrogenase, short chain; Acadvl, acyl-Coenzyme A dehydrogenase, very long chain; Acox1, acyl-Coenzyme A oxidase 1, palmitoyl; Cpt2, carnitine palmitoyltransferase 2; Crat, carnitine acetyltransferase; Dci, dodecenoyl-Coenzyme A delta isomerase (3,2 trans-enoyl-Coenzyme A isomerase); Echs1, enoyl-Coenzyme A hydratase, short chain 1 mitochondrial; Ehhadh, enoyl-Coenzyme A, hydratase/3-hydroxyacyl-Coenzyme A dehydrogenase; Hadhb, hydroxyacyl-Coenzyme A dehydrogenase/3-ketoacyl-Coenzyme A thiolase/enoyl-Coenzyme A hydratase, beta subunit. (c) Genes related to citrate metabolism and oxidative phosphorylation: Atp5a1, ATP synthase, H+ transporting, mitochondrial F1 complex, alpha subunit, isoform 1; Atp5b, ATP synthase, H+ transporting mitochondrial F1 complex, beta subunit; Cox1, M. musculus mRNA for mitochondrial gene for subunit I of cytochrome c oxidase; Cs, citrate synthase; Fh1, fumarate hydratase 1; Mdh2, malate dehydrogenase 2, NAD (mitochondrial); Sdha, succinate dehydrogenase complex, subunit A, flavoprotein (Fp); Sdhb, succinate dehydrogenase complex, subunit B, iron sulfur (Ip); Sucla2, succinate-Coenzyme A ligase, ADP-forming, beta subunit; Uqcrc1, Uqcrc 2, ubiquinol-cytochrome c reductase core protein 1, 2; Uqcrfs1, ubiquinol-cytochrome c reductase, Rieske iron-sulfur polypeptide 1; Uqcrq, ubiquinol-cytochrome c reductase, complex III subunit VII. *gene abbreviations given in Figure 1 were not mentioned.

Contrary to the liver, RSG treatment in IAT regulated the majority of metabolism-related genes in the same direction as observed in db/+ mice when compared to db/db. Indeed, genes involved in glucose metabolism, FFA and TG metabolism, pentose phosphate synthesis, and mitochondrial function were upregulated and/or normalised by RSG-treatment, some of them with a clear dose effect (Figures 4(a), 4(b), 4(c)). RSG treatment (10–100 mg/kg) partially normalised mRNA expression (Figure 4(a)) of two key genes involved in glucose metabolism such as the glucose transporter Glut4 (Slc2a4, +45%, *P* < .001 in 30 mg/kg RSG group and +114%, *P* < .001 in db/+ group) and the pyruvate dehydrogenase kinase 4 the downregulation of which stimulates glucose metabolism (Pdk4, *‒*37%, *P* < .001 in 30 mg/kg RSG group and no change in db/+ group). On one hand, we observed partial normalisation of genes encoding FFA or TG metabolic enzymes including Acaca (+163%, in 30 mg/kg RSG group and +474%, in db/+ group, *P* < .001) and Fasn (+240%, in 30 mg/kg RSG group and +674%, in db/+ group, *P* < .001) or the pentose phosphate pathway gene Tkt (+86%, in 30 mg/kg RSG group and +409% in db/+ group, *P* < .001) (Figure 4(a)). On the other hand, for almost all genes involved in mitochondrial *β*-oxidation, citrate cycle, and oxidative phosphorylation, the level of upregulated genes observed in RSG-treated mice far exceeded that of db/+ regulated genes (Figures 4(b), 4(c)). All these results were validated with qPCR technique with strong correlation (Supplemental Table 3 and Figure 2(c); *r* = 0.83, *P* < .001; number of xy pairs = 96; Figure 2(d); *r* = 0.80, *P* < .001; number of xy pairs = 156).

### 3.7. Mitochondrial Renewal in db/db IAT Treated with RSG

**Figure 5:**
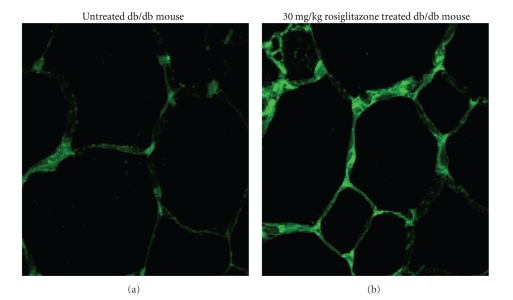
Immunochemical detection in db/db and RSG-treated db/db of the IAT OxPhos complex IV subunit I. IAT from untreated db/db (a) and from 30 mg/kg RSG-treated db/db (b) were isolated, fixed on slide, and stained with anti-OxPhos complex IV subunit I. Nuclei were identified with Hoechst 33342.

The gene expression changes observed in adipose tissue suggested a mitochondrial dysfunction in db/db diabetic mice, but the RSG treatment appears to normalise a part of these alterations. To test whether diabetes and RSG also affected mitochondrial biogenesis and functioning at cellular level, the mitochondrial content of adipocytes was determined by using a monoclonal antibody specific for OxPhos Complex IV subunit I (Cox1), a mitochondrial membrane-bound protein complex. We have compared control db/db versus 30 mg/kg RSG-treated db/db adipocytes mitochondrial content, to avoid any possible side effects of the 100 mg/kg dose. As shown in Figure 5, signal intensity was stronger in adipocytes treated with RSG than in control db/db diabetic mice (*P* < .05). These results confirmed the transcriptional upregulation of Cox1 (+74%; *P* < .001) observed at 30 mg/kg dose and suggested that RSG induced mitochondrial biogenesis in adipocytes, as previously described [23, 24].

### 3.8. Effect of RSG on Metabolic-Related mRNA Expression in Soleus Muscle

**Figure 6:**
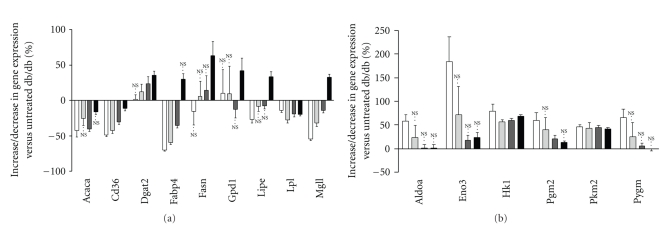
Expression profiles of key genes involved in Soleus muscle glucose and lipid metabolism. Differentially expressed genes in db/+ (the white square) and 10 (the off-white square), 30 (the grey square), and 100 (the black square)  mg/kg RSG-treated db/db mice were measured *versus* untreated db/db and plotted as the mean (% increase/decrease of db/db control) ±SEM (*n* = 6). NS; *P* ≥ .001 otherwise *P* < .001. (a) Genes related to glucose metabolism*: Eno3, enolase 3, beta muscle; Hk1, hexokinase 1; Pkm2, pyruvate kinase, muscle; Pgm2, phosphoglucomutase 2; Pygm, muscle glycogen phosphorylase. (b) Genes related to lipid metabolism: Dgat2, diacylglycerol O-acyltransferase 2; Lipe, lipase hormone sensitive; *gene abbreviations given in Figures 1, and 4 were not mentioned.

In soleus muscle among the 506 regulated gene sequences 186 were differentially expressed either in db/+ versus db/db or RSG-treated db/db versus db/db (Supplemental Table 2). Soleus muscle plays an important role in both carbohydrate and FFA metabolism. It is well established that in obesity there is a dysfunction in the capacity of skeletal muscles to store glycogen. Also, increased TG storage is positively correlated with markers of insulin resistance. Supplemental Figure 4 displayed microarray data overview of differentially expressed genes between lean db/+ or RSG-treated db/db mice and the untreated diabetic db/db involved in glucose and FFA metabolism. Fewer genes were regulated by RSG treatment in muscle in comparison to the liver and IAT. However, similarly to the differential gene expression observed between the db/+ and db/db diabetic mice, RSG induced an upregulation of key genes involved in carbohydrate metabolism such as Pkm2 (+44%, +45%, and +42% at 10, 30, and 100 mg/kg RSG, resp.) and HK1 (+57%, +60%, and +69%). Moreover, RSG treatment resulted in the repression of genes involved in lipid transport (Figure 6(a)), like Cd36 (*‒*42%, *‒*30%, and *‒*11% at 10, 30, and 100 mg/kg RSG, resp.) and Fabp4 (*‒*59%, *‒*35%, and +30%). RSG also decreased the expression of genes involved in FA and TG metabolism like Acaca (*‒*25%, *‒*40%, and *‒*16% at 10, 30, and 100 mg/kg RSG, resp.) and MgII (*‒*32%, *‒*14%, and +33%) (Figure 6(a)). It is noteworthy that the changes induced in gene expression by 10 mg/kg RSG tended to be most similar to db/+ versus db/db than the 30 and 100 mg/kg doses (see Figure 6(a), Acaca, Fabp4 or Mgll). This tendency seemed to be specific to soleus muscle and suggests possible toxic or secondary effects of high RSG concentrations. Taken together, these results suggest a decrease in FFA uptake and an increase in glucose utilization in soleus muscle of RSG-treated animals.

### 3.9. PLS Analysis

**Figure 7:**
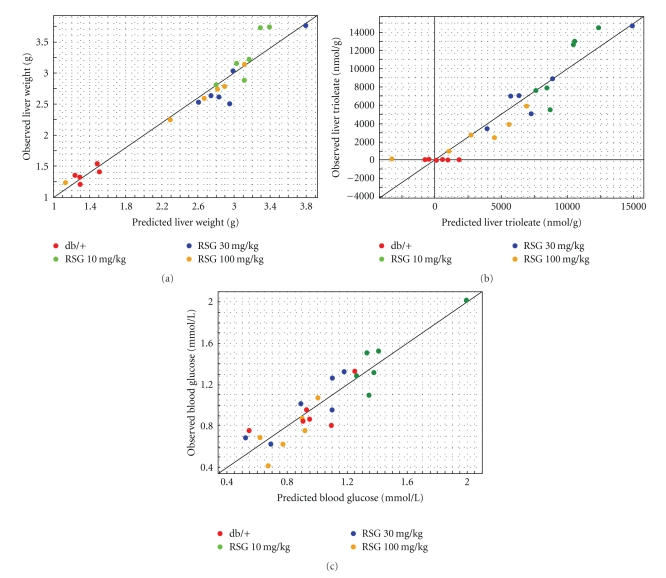
Generation of PLS models to predict physiological changes from multivariate gene expression data. Results show the correlations between the actual liver weight (a), the liver trioleate concentrations (b), the blood glucose level (c) and the predicted parameters from the PLS model. The normalised logs (ratio) of genes involved in glucose and lipid metabolism were used as predictor variables (X) and physiological parameters as response variables (Y). All variables were centred and scaled to unit variance, before the PLS analysis was performed.

Multivariate statistical approach (PLS) has been applied in order to establish potential relationships between the changes in transcriptional profile and metabolic parameters under RSG treatments (Table 1). This method utilizes a linear regression model on latent structure to find correlations between two data matrices (X and Y). The significant differentially expressed sequences (Supplemental Table 2) involved in metabolism were defined as predictor variables (X) and FFA, TG (tissues and plasma) and glucose concentrations were defined as observation variables (Y). The models were retained based on *R* ^2^_*Y*cum_ (% of explained sum of squares), *Q* ^2^_*Y*cum_ (% of predictive sum of squares) and *P*-value. No model based on differential gene expression using data from IAT (391 sequences) and soleus (186 sequences) could be obtained. However, the predictor variables corresponding to differentially expressed sequences in liver (276) enable us to derive significant PLS models regarding the Y variables such as oleic acid and trioleate tissue concentrations (Table 2) as well as the liver weight. Figures 7(a), 7(b) show the correlation between the observed trioleate concentrations or liver weight and the predicted ones obtained from PLS models built using the 40 best sequence predictors (*R* ^2^_*Y*cum_ = 0.89 and 0.96, resp.). Among the 40 sequence predictors for liver trioleate concentrations and weight, 20 are common. Removing redundancy among the 20 predictors led to 16 genes from which 13 displayed PPRE in their proximal promoters as determined by MatInspector from Genomatix suite (see Section 2 and Supplemental Table 4). An interesting observation is that using the combination of differential gene expression from the three tissues (853 gene sequences) we were able to find a PLS model that made the link between predictor gene sequences and the blood glucose concentrations. Correlation between observed and predicted glucose using the 40 best predictors led to an acceptable model (*P* = .007 and *R* ^2^_*Y*cum_ = 0.83; Figure 7(c)) but predictive potency of the model remained rather weak (*Q* ^2^_*Y*cum_ = 0.68). Among the 40 predictors 35 belong to the IAT, 4 to the liver, and 1 to the soleus. A great deal of IAT mRNA- (24/35 sequences) encoded proteins involved in the mitochondrial respiration and tricarboxylic acid cycle (Table 3) and 3 out of 4 genes from liver are involved in gluconeogenesis. Finally, among the 32 unique genes (corresponding to the 40 gene sequences), 21 presented a PPRE in their promoter regulatory sequences. 

These observations underline the link between RSG-mediated PPAR*γ* activation and expression of genes involved in IAT mitochondrial function, liver gluconeogenesis, and regulation of blood glucose.

## 4. Discussion

In this paper, for the first time we have attempted to establish a link between metabolic status of RSG-treated diabetic mice (db/db) and RSG-mediated transcriptomic changes in the three major insulin-dependent tissues including liver, adipose tissue, and skeletal muscle. 

As expected, numerous genes regulated by RSG and identified in this study fall into key metabolic pathways involved in carbohydrate and lipid metabolism. PPAR*γ* agonist also regulates many more genes in adipose tissue than in liver or skeletal muscle, as might be expected, based on PPAR*γ* expression levels in the respective tissues. Despite this difference, our data showed that PPAR*γ* activation has coordinated effects on fundamental metabolic pathways in each of these tissues, including glucose and lipid metabolism in skeletal muscle, gluconeogenesis in liver, as well as lipogenesis, TG storage, and mitochondrial function in adipose tissue. Gene transcription modulation by RSG could be classified into two types, namely, those which counteract diabetes-induced alteration and correlated to the modulation observed in db/+ versus db/db and those which accentuate diabetes induced alteration and are inversely correlated. The first group could explain the glucose and lipid-lowering actions of PPAR*γ* agonists, and the second group could be linked to TZDs compound-mediated side effects.

### 4.1. Liver

One of the most interesting observations is that RSG treatment normalised glucose homeostasis in db/db mice (result presented here in and in [15]) and the mRNA expression of gluconeogenic key enzymes such as the G6pc, Fbp1, and the glucose transporter Glut-2. These results suggest that transcriptional regulation of G6pc and Fbp1 mRNA play a role in RSG-mediated decreased gluconeogenesis and blood glucose normalisation. This observation is in agreement with previous studies showing an increase in G6pc and Fbp1 activities in db/db mice and the subsequent increase in glucose production by the db/db liver [25].

 In human, hepatic gluconeogenesis is known to be significantly enhanced in type 2 diabetes and normalised by RSG [10, 26]. Therefore, the transcriptional status of G6Pc and Fbp1, related to RSG-mediated gluconeogenesis normalisation, deserves to be further investigated in humans.

In rodents one of the thiazolidinedione treatment side effects is associated with liver dysfunction. RSG-treated db/db mice have shown a rise in their liver weight and steatosis appearance, as previously shown [12]. Indeed, the potent antihyperglycaemic effect of RSG was accompanied by an increase of *de novo* synthesis of fatty acids when compared to either db/+ or db/db. Lipid metabolome analysis was concordant with our gene expression profiling obtained in liver and corroborate with the results obtained in a previous study [13]. A rise in hepatic expression of genes belonging to numerous steps of the TG synthesis in liver was observed under RSG treatment including those that encode for FA transporters (Lpl, Cd36, Fabp2, 4), TG (Gpd1) and FFA synthesis (Acly, Fasn, Acaca, Gpd1, Agpat2, 6, Scd1) and are likely associated to the RSG-mediated increase in hepatic trioleate levels. This result is in contrast with the RSG effects in humans and normal mice, where chronic RSG treatment reduced liver fat [27, 28]. However db/db is a leptin signalling deficient paradigm, and numerous publications demonstrated in wild-type mice that leptin decreases hepatic *de novo* synthesis of FA through the decrease in mRNA, proteins, or enzymatic activities of FFA and TG metabolism enzymes including Acly, Acaca, Fas, Scd-1, or Agpat [29–33]. Therefore, mice lacking leptin signalling are not the best paradigm to foresee PPAR agonist secondary effects in human liver.

### 4.2. Inguinal Adipose Tissue

In adipose tissue, mitochondria are not only the major site of fatty acid oxidation but may also play a critical role in lipogenesis by providing key intermediates for TG synthesis. Impaired mitochondria may lead to the lack of ATP and subsequent reduction in lipid metabolism [23]. This dysfunction could be linked to a reduced ability of glucose utilization, participating in high blood glucose levels in diabetic mice. A decrease in ATP may also impair the synthesis and secretion of adipokines, which were previously shown to be associated with diabetes [34]. In our study, RSG significantly induced a dose-dependent increase in a number of genes implicated in mitochondrial activities. Genes encoding for enzymes involved in *β*-oxidation, citrate cycle, and oxidative phosphorylation were upregulated in a similar manner to that observed in db/+ versus db/db mice. Moreover, we have observed an upregulation of PPAR*γ* coactivator PGC1*α* (data not shown), which is known to potently activate mitochondrial biogenesis in adipose and muscle tissues [24, 35]. Combined with immunocytochemistry results, these data showed that RSG treatment led to an increase in mitochondrial biogenesis. The effects of PPAR*γ* agonist on both mitochondrial number and morphology were previously observed in adipose tissues of rat and dog treated with RSG [36]. RSG is also known to induce adipocyte differentiation and mitochondrial biogenesis [37]. These new small adipocytes are insulin sensitive and possess a higher lipid metabolism capacity that could explain the RSG-increased mRNA of genes involved in lipid transport and oxidation. The IAT is composed of both brown adipose tissue (BAT) and white adipose tissue (WAT). Our data show the upregulation of BAT markers Ucp1 or Cpt1b in IAT of treated mice, as described by others [34]. This observation supports the view of RSG converting WAT to BAT, transforming IAT into fat-oxidizing machinery. They also suggest that decreasing the exposure of peripheral tissues to lipids may improve the whole-body insulin sensitivity.

### 4.3. Soleus Muscle

Normalisation of hyperglycaemia by RSG-decreased hepatic glucose synthesis and output seems to be combined with better use of glucose in soleus muscle. Indeed, RSG-treated mice showed an increased expression of genes coding for key enzymes involved in glycolysis, like Hexokinase1 Hk1, Enolase Eno3, or Pyruvate kinase Pkm2, similar to our observation in db/+ versus db/db. RSG treatment of db/db also resulted in a coordinated decrease in the expression of some genes involved in fatty acid transport and metabolism in muscle. It is noteworthy that the expression of these genes was upregulated in liver and to a lesser extent in IAT in response to RSG, suggesting that PPAR*γ* activation promotes a flux of fatty acids into hepatic and adipose tissues and away from muscle. Altogether, these data suggest a decreased reliance on fatty acids and an increased reliance on glucose as an energy source in muscle, as previously described [2, 38]. Surprisingly, the RSG-mediated gene expression normalisation in muscular cells was observed mainly at 10 mg/kg dose, whereas the highest doses induced the expression of genes related to glucose and lipid pathways in opposite direction to that measured in db/+ versus db/db, suggesting a possible adverse effect at higher drug concentrations in this tissue.

### 4.4. PLS Models

Taking advantage of the experimental design including both transcriptomic and metabolomic approaches, we applied a multivariate linear regression model (PLS) in order to predict the metabolic parameters (i.e., blood glucose and FFA and TG from blood and tissues) based on regulated genes (predictor (X) variables). Only the variables X from liver allowed us to derive models that significantly predicted some of the measured biological parameters such as liver oleic acid and trioleate as well as the liver weight. It is worth noticing that among the 40 best predictors 20 (16 genes) are common between trioleate and the liver weight model. The fact that among these 16 genes 13 display a PPRE and therefore are supposed to be regulated by PPAR agonist reinforces the predictory status of these 20 gene sequences. Obviously more experiments should be undertaken with different RSG dose treatments to confirm these predictors. Moreover, no significant model could be built with differential gene expression data from liver and blood metabolic parameters, and no model could be obtained with IAT or soleus muscle regarding both tissue and blood metabolic parameters. However, the most interesting observation is that a model was validated with blood glucose levels and the combination of gene expression data from the 3 tissues. When looking at the 40 best predictors, we found that a large majority of gene sequences implied IAT-expressed genes (35/40), and among the 35, 25 encoded mitochondrial proteins involved in energy cell production and 4 gene sequences were originated from the liver and are involved in gluconeogenesis. In that case we may hypothesise that those blood glucose predictors are linked to molecular mechanisms because; (i) mitochondrial dysfunction is linked to diabetes type 2 and thiazolidinediones were known to improve the diabetes status by the production of more functional adipocytes; (ii) in db/db mice high levels of blood glucose partially is due to gluconeogenesis [25] and RSG by decreasing mRNA encoding gluconeogenic enzymes may indeed regulate blood glucose. 

Therefore, PLS reveals not only IAT/liver predictors for blood glucose regulation but also a potential molecular mechanism that could explain in part the glucose regulation. Whether or not this mechanism is due to direct PPAR*γ*-induced transcriptional effects or PPAR interaction with the mitochondrial protein such as MitoNEET [39] that subsequently activate mitochondrial functions remains to be investigated, but a majority of genes in the top 40 predictor sequences (21/32 genes) displayed predicted PPRE in their respective promoter.

## 5. Conclusion

To our knowledge, this is the first study comparing gene expression profiles between db/+ and RSG-treated db/db with control diabetic db/db in liver, muscle, and adipose tissues. The combination of transcriptomic and metabolomic approaches led to a comprehensive molecular portrait and hypothesis on the dose-dependent effects of RSG in db/db diabetic mice and highlights the role of the respective insulin-dependent tissues. This approach could be useful in the future to discriminate between selective PPAR modulators regarding their specific molecular profiles in relation to specific target tissues.

## Supplementary Material

Supplementary Figure 1 : Cellular and molecular functions altered by type II diabetes mellitus and Rosiglitazone treatment.
Supplementary Figure 2: Two dimensional representation of differentially expressed genes involved in liver carbohydrates metabolism (A) and in liver FA and TG metabolisms (B).
Supplementary Figure 3: Two dimensional representation of differentially expressed genes involved in IAT FFA and TG metabolisms.
Supplementary Figure 4: Two dimensional representation of differentially expressed genes involved in Soleus muscle lipid and carbohydrate metabolisms.
Supplementary Table 1: Overview of the number of sequences up- and down-regulated in db/+ and RSG-treated samples compared to db/db.
Supplementary Table 2: Supplemental Table 2: Differentially expressed genes (506 sequences probes) involved in lipid and carbohydrate metabolisms.
Supplementary Table 3: Supplemental Table 3: Liver and IAT real-time PCR data analysis and comparison with corresponding microarray results.
Supplementary Table 4: Supplemental Table 4: Common sequence predictors for liver trioleate concentrations and weight, determined using PLS analysis.

## Figures and Tables

**Table 1:** Animal characteristics, blood and tissue analysis: db/db were compared by Student's “*t*” test versus db^+^, ^#^ *P* < .05; ^##^ *P* < .01; ^###^ *P* < .001. RSG-treated groups were compared by ANOVA and Dunnett test versus untreated db/db, *P < .05; **P < .01; ***P < .001, or versus db/+, ^†^ *P* < .05; ^††^ *P* < .01; ^†††^ *P* < .001. *n* = 6 animals otherwise specified.

	db/db				db/+
Parameters (units)	Untreated	RSG 10 mg/kg	RSG 30 mg/kg	RSG 100 mg/kg	Untreated
Body weight 0d	38.9 ± 0.4^###^	39.4 ± 0.6	39.8 ± 0.7	39.5 ± 1.1	24.6 ± 0.7
Body weight (g) 18d	40.6 ± 0.7^###^	46.2 ± 0.9**	47.8 ± 0.6***	46.8 ± 2.1**	25.9 ± 0.4
Liver weight (g)	1.75 ± 0.03^###^	3.25 ± 0.17^∗∗∗ †††^	2.83 ± 0.20^∗∗ †††^	2.47 ± 0.27^∗ ††^	1.37 ± 0.05
Inguinal Adipose weight (g)	2.09 ± 0.06^###^	1.99 ± 0.11^†††^	2.09 ± 0.13^†††^	2.16 ± 0.09^†††^	0.17 ± 0.02
Soleus muscle weight (mg)	6.67 ± 1.33	6.00 ± 0.85	5.05 ± 0.5	4.79 ± 0.36	6.13 ± 1.82
Blood Glucose (mmol/l) 0d	16.9 ± 1.3^###^	16.3 ± 1.3^†††^	15.8 ± 0.9^†††^	16.4 ± 1.1^†††^	5.8 ± 0.4
Blood Glucose (mmol/l) 18d	15.1 ± 0.9^###^	8.0 ± 0.7^∗∗∗††^	5.4 ± 0.6***	4.1 ± 0.5***	5.1 ± 0.5
HbA1c %	7.14 ± 0.28^###^	6.16 ± 0.34^†††^	5.76 ± 0.32^∗∗†††^	5.52 ± 0.23^∗∗†††^	3.61 ± 0.13

Plasmatic triglycerides (nmol/mL)					

Trilinoleate (*n* = 3 − 5)	49.6 ± 2.3	16.8 ± 6.5***	10.2 ± 3.0***	23.2 ± 5.4**	26.8 ± 10.2
Trioleate (*n* = 3 − 5)	19.7 ± 1.4^#^	6.7 ± 1.6***	3.2 ± 0.9***	8.0 ± 2.5***	11.8 ± 3.4

Plasmatic FFA (nmol/mL)					

Palmitic acid	91.8 ± 5.5	70.4 ± 7.0	51.4 ± 7.8***	64.9 ± 7.0*	85.9 ± 10.5
Linoleic acid	83.8 ± 4.5^#^	70.5 ± 6.0	43.0 ± 10.2*	54.5 ± 12.7	65.3 ± 4.4
Oleic acid	91.1 ± 12.0	72.1 ± 5.4	38.4 ± 12.1**	47.4 ± 13*	70.2 ± 8.9

Liver triglycerides (nmol/g)					

Trilinoleate	116 ± 24^##^	76.0 ± 21.9^†^	43.2 ± 8.9*	33.5 ± 4.4**	20.0 ± 3.9
Trioleate	1033 ± 314^#^	10240 ± 1489^∗∗∗ †††^	7746 ± 1603^∗∗ †††^	2708 ± 846	59 ± 13

Liver FFA (nmol/g)					

Palmitic acid	481 ± 49	583 ± 43^††^	573 ± 47^†^	702 ± 67^∗∗ †††^	366 ± 13
Linoleic acid	289 ± 27^#^	236 ± 7	253 ± 17	331 ± 46^††^	209 ± 11
Oleic acid	484 ± 87^#^	1031 ± 110^∗∗ †††^	997 ± 82^∗∗ †††^	945 ± 148^∗ †††^	186 ± 18

IAT triglycerides (nmol/g)					

Trilinoleate	5965 ± 2373	3670 ± 1177^††^	5343 ± 1542^†^	4118 ± 674^†^	15524 ± 4320
Trioleate	7766 ± 1962	6515 ± 2729	15382 ± 5458	11836 ± 3059	16016 ± 5333

IAT FFA (nmol/g)					

Palmitic acid	236 ± 61	265 ± 24	302 ± 67	462 ± 36*	424 ± 92
Linoleic acid	329 ± 53^#^	328 ± 25	454 ± 97	570 ± 57*	558 ± 81
Oleic acid	281 ± 48^##^	310 ± 32^†^	416 ± 110^††^	514 ± 56^†††^	65 ± 10

**Table 2:** Model parameters from multivariate analysis (PLS) based on liver, IAT and muscle gene expression. R^2^X: % of variation of X that explained Y; *R* ^2^_*Y*cum_: % of variation of Y explained by the model; *Q* ^2^_*Y*cum_: % of variation of Y predicted by the model. Cum are for all PLS components.

	PLS component	R^2^X(cum)	R^2^Y(cum)	Q^2^(cum)	*P*-value
Liver					
Oleic 275 gene sequences	1	0.67	0.78	0.76	3.06E-07
Oleic 40-top gene sequences	1	0.94	0.80	0.80	5.00E-08
Trioleate 275 gene sequences	2	0.74	0.86	0.80	3.11E-06
Trioleate 40-top gene sequences	2	0.72	0.89	0.85	2.30E-07
Weight 275 gene sequences	2	0.77	0.96	0.95	4.92E-11
Weight 40-top gene sequences	1	0.75	0.96	0.93	1.80E-14
Liver+IAT+Soleus					
Glycemia 846 gene sequences	4	0.71	0.94	0.73	1.00E+00
Glycemia 40-top gene sequences	3	0.86	0.84	0.68	7.00E-03

**Table 3:** Differential expression of the 40 best predictor gene sequences from PLS analysis of combined tissues: liver, IAT, and soleus. x: MatInspector-predicted PPRE.

Tissues	Gene name	Function	Sequence description	PPRE	db/+	RSG 10 MK	RSG 30 MK	RSG 100 MK
					F. C.	F. C.	F. C.	F. C.
S	Cyp4a12a	Fatty acid metabolism	cytochrome P450, family 4, subfamily a, polypeptide 12a		1.72	1.25	1.27	1.46
TA	Dci	Fatty acid metabolism	dodecenoyl-Coenzyme A delta isomerase (3,2 trans-enoyl-Coenzyme A isomerase)	x	1.51	1.28	1.68	1.85
TA	Mcat	Fatty acid metabolism	malonyl CoA: ACP acyltransferase (mitochondrial)		1.57	1.29	1.54	1.66
TA	Ppargc1b	Fatty acid metabolism	peroxisome proliferative activated receptor, gamma, coactivator 1 beta		1.63	−1.06	1.37	1.83
TA	Ppargc1b	Fatty acid metabolism	peroxisome proliferative activated receptor, gamma, coactivator 1 beta		2.11	1.10	1.67	2.33
TA	Ppargc1b	Fatty acid metabolism	peroxisome proliferative activated receptor, gamma, coactivator 1 beta		1.41	−1.10	1.30	1.80
TA	Dlat	Gluconeogenesis	dihydrolipoamide S-acetyltransferase (E2 component of pyruvate dehydrogenase complex)	x	1.77	1.49	2.10	2.72
TA	Ldhb	Gluconeogenesis	lactate dehydrogenase B		1.76	1.07	1.36	1.81
TA	Pdhb	Gluconeogenesis	pyruvate dehydrogenase (lipoamide) beta		2.19	1.69	2.49	3.05
TA	Pdhb	Gluconeogenesis	pyruvate dehydrogenase (lipoamide) beta		2.04	1.65	2.32	2.82
L	Pfkfb2	Gluconeogenesis	6-phosphofructo-2-kinase/fructose-2,6-biphosphatase 2	x	−1.16	−1.72	−1.59	−1.56
L	Ppargc1a	Gluconeogenesis	peroxisome proliferative activated receptor, gamma, coactivator 1 alpha	x	−1.32	−1.53	−1.61	−1.59
L	Ppargc1a	Gluconeogenesis	peroxisome proliferative activated receptor, gamma, coactivator 1 alpha		−1.64	−1.71	−1.75	−1.75
TA	Atp5h	Mitochondrial respiratory chain	ATP synthase, H+ transporting, mitochondrial F0 complex, subunit d	x	1.21	1.07	1.26	1.41
TA	Atp5h	Mitochondrial respiratory chain	ATP synthase, H+ transporting, mitochondrial F0 complex, subunit d		1.23	1.06	1.26	1.47
TA	Cox6b1	Mitochondrial respiratory chain	cytochrome c oxidase, subunit VIb polypeptide 1		1.54	1.39	1.82	2.16
TA	Cox6b2	Mitochondrial respiratory chain	cytochrome c oxidase, subunit VIb polypeptide 2		1.35	1.08	1.37	1.59
TA	Cox7a1	Mitochondrial respiratory chain	cytochrome c oxidase, subunit VIIa 1	x	1.37	−1.52	1.45	1.98
TA	Cox7b	Mitochondrial respiratory chain	cytochrome c oxidase, subunit VIIb	x	1.96	1.33	1.87	2.15
TA	Ndufa1	Mitochondrial respiratory chain	NADH dehydrogenase (ubiquinone) 1 alpha subcomplex, 1	x	1.44	1.13	1.42	1.52
TA	Ndufa1	Mitochondrial respiratory chain	NADH dehydrogenase (ubiquinone) 1 alpha subcomplex, 1		1.35	1.09	1.31	1.46
TA	Uqcrb	Mitochondrial respiratory chain	ubiquinol-cytochrome c reductase binding protein	x	1.81	1.40	1.82	2.14
TA	Uqcrfs1	Mitochondrial respiratory chain	ubiquinol-cytochrome c reductase, Rieske iron-sulfur polypeptide 1		1.57	1.24	1.87	2.25
TA	Uqcrh	Mitochondrial respiratory chain	ubiquinol-cytochrome c reductase hinge protein		1.89	1.43	1.81	1.97
TA	Uqcrh	Mitochondrial respiratory chain	ubiquinol-cytochrome c reductase hinge protein		1.65	1.28	1.57	1.83
TA	Fntb	Steroid biosynthesis	farnesyltransferase, CAAX box, beta	x	1.48	1.43	1.55	1.49
L	Hmgcs2	Steroid biosynthesis	3-hydroxy-3-methylglutaryl-Coenzyme A synthase 2	x	−1.32	−1.09	−1.28	−1.37
TA	Agpat3	Triglyceride	1-acylglycerol-3-phosphate O-acyltransferase 3	x	1.81	1.31	1.65	2.07
TA	Aco1	Tricarboxylic acid cycle (mit oxidation)	aconitase 1	x	1.77	1.37	1.70	1.94
TA	Aco2	Tricarboxylic acid cycle (mit oxidation)	aconitase 2, mitochondrial	x	1.38	1.18	1.66	1.98
TA	Aldh5a1	Tricarboxylic acid cycle (mit oxidation)	aldhehyde dehydrogenase family 5, subfamily A1	x	1.60	1.33	1.65	1.69
TA	Dlst	Tricarboxylic acid cycle (mit oxidation)	dihydrolipoamide S-succinyltransferase (E2 component of 2-oxo-glutarate complex)	x	1.43	1.16	1.63	1.83
TA	Fh1	Tricarboxylic acid cycle (mit oxidation)	fumarate hydratase 1	x	1.56	1.28	1.63	1.87
TA	Idh3b	Tricarboxylic acid cycle (mit oxidation)	isocitrate dehydrogenase 3 (NAD+), beta	x	1.49	1.18	1.73	1.91
TA	Idh3g	Tricarboxylic acid cycle (mit oxidation)	isocitrate dehydrogenase 3 (NAD+), gamma	x	1.67	1.18	1.59	1.91
TA	Mdh1	Tricarboxylic acid cycle (mit oxidation)	malate dehydrogenase 1, NAD (soluble)		1.93	1.17	1.91	1.95
TA	Mdh1	Tricarboxylic acid cycle (mit oxidation)	malate dehydrogenase 1, NAD (soluble)		1.63	1.44	2.01	2.17
TA	Sdhb	Tricarboxylic acid cycle (mit oxidation)	succinate dehydrogenase complex, subunit B, iron sulfur (Ip)		1.60	1.21	1.67	1.97
TA	Sdhc	Tricarboxylic acid cycle (mit oxidation)	succinate dehydrogenase complex, subunit C, integral membrane protein	x	1.32	1.10	1.35	1.48
TA	Sdhd	Tricarboxylic acid cycle (mit oxidation)	succinate dehydrogenase complex, subunit D, integral membrane protein	x	1.29	1.11	1.57	1.90

## Abbreviations

FFAs: Free fatty acids
RSG: Rosiglitazone
IAT: Inguinal adipose tissue
PPAR: Peroxisome proliferator-activated receptor
PPRE: PPAR response element
PLSs: Partial least squares
TZD: Thiazolidinedione
TGs: Triglycerides
UPLC-MS: Ultra performance liquid chromatography-mass spectrometry.
