# Supplementary material for: Coordinate Transcriptomic and Metabolomic Effects of the Insulin Sensitizer Rosiglitazone on Fundamental Metabolic Pathways in Liver, Soleus Muscle, and Adipose Tissue in Diabetic db/db Mice
Source: PPAR Res. 2010 Oct 10;2010:679184. doi: 10.1155/2010/679184 (PMC2953354; doi:10.1155/2010/679184)
Supplement: Supplementary file 1 — Supplementary Figure 1 : Cellular and molecular functions altered by type II diabetes mellitus and Rosiglitazone treatment. Supplementary Figure 2: Two dimensional representation of differentially expressed genes involved in liver carbohydrates metabolism (A) and in liver FA and TG metabolisms (B). Supplementary Figure 3: Two dimensional representation of differentially expressed genes involved in IAT FFA and TG metabolisms. Supplementary Figure 4: Two dimensional representation of differentially expressed genes involved in Soleus muscle lipid and carbohydrate metabolisms. Supplementary Table 1: Overview of the number of sequences up- and down-regulated in db/+ and RSG-treated samples compared to db/db. Supplementary Table 2: Supplemental Table 2: Differentially expressed genes (506 sequences probes) involved in lipid and carbohydrate metabolisms. Supplementary Table 3: Supplemental Table 3: Liver and IAT real-time PCR data analysis and comparison with corresponding microarray results. Supplementary Table 4: Supplemental Table 4: Common sequence predictors for liver trioleate concentrations and weight, determined using PLS analysis. [file 679184.f1.pdf]

Supplemental Figure 1 : Cellular and molecular functions altered by type II diabetes mellitus and Rosiglitazone treatment. Microarray data were analysed by IPA. *Dark blue bars*, db/+ *vs.* db/db; *medium-blue bars,* db/db mice treated with 10 mg/kg RSG vs. db/db; *bright-blue bars*, db/db mice treated with 30 mg/kg RSG vs. db/db, *black bars,* db/db mice treated with 100 mg/kg RSG vs. db/db; *bold line*, threshold p-value <0.05, Fisher test and Benjamini-Hochberg correction. * The diabetes effect was given as reciprocal (db/+ vs. db/db) to facilitate direct comparisons with normalizing effect of RSG.

Supplemental Figure 2: Two dimensional representation of differentially expressed genes involved in liver carbohydrates metabolism (A) and in liver FA and TG metabolisms (B). The input consisted of db/+ or RSG-treated db/db *vs.* untreated db/db reference group. Each gene is represented by a column of coloured boxes and each group by a single row. Each coloured box represent the gene expression value in one group, with expression values ranging from bright green (lowest) to bright red (highest). Missing value are in grey when *p*-value ≥0.001.

Supplemental Figure 3: Two dimensional representation of differentially expressed genes involved in IAT FFA and TG metabolisms. Same overall format as in Supplemental Figure 2. Missing value are in grey when *p*-value ≥0.001.

Supplemental Figure 4: Two dimensional representation of differentially expressed genes involved in Soleus muscle lipid and carbohydrate metabolisms. Same overall format as in Supplemental Fig. 2. Genes involved in glucose, FA, and TG metabolisms were studied. Missing value are in grey when *p*-value ≥0.001.

Supplemental Fig.1

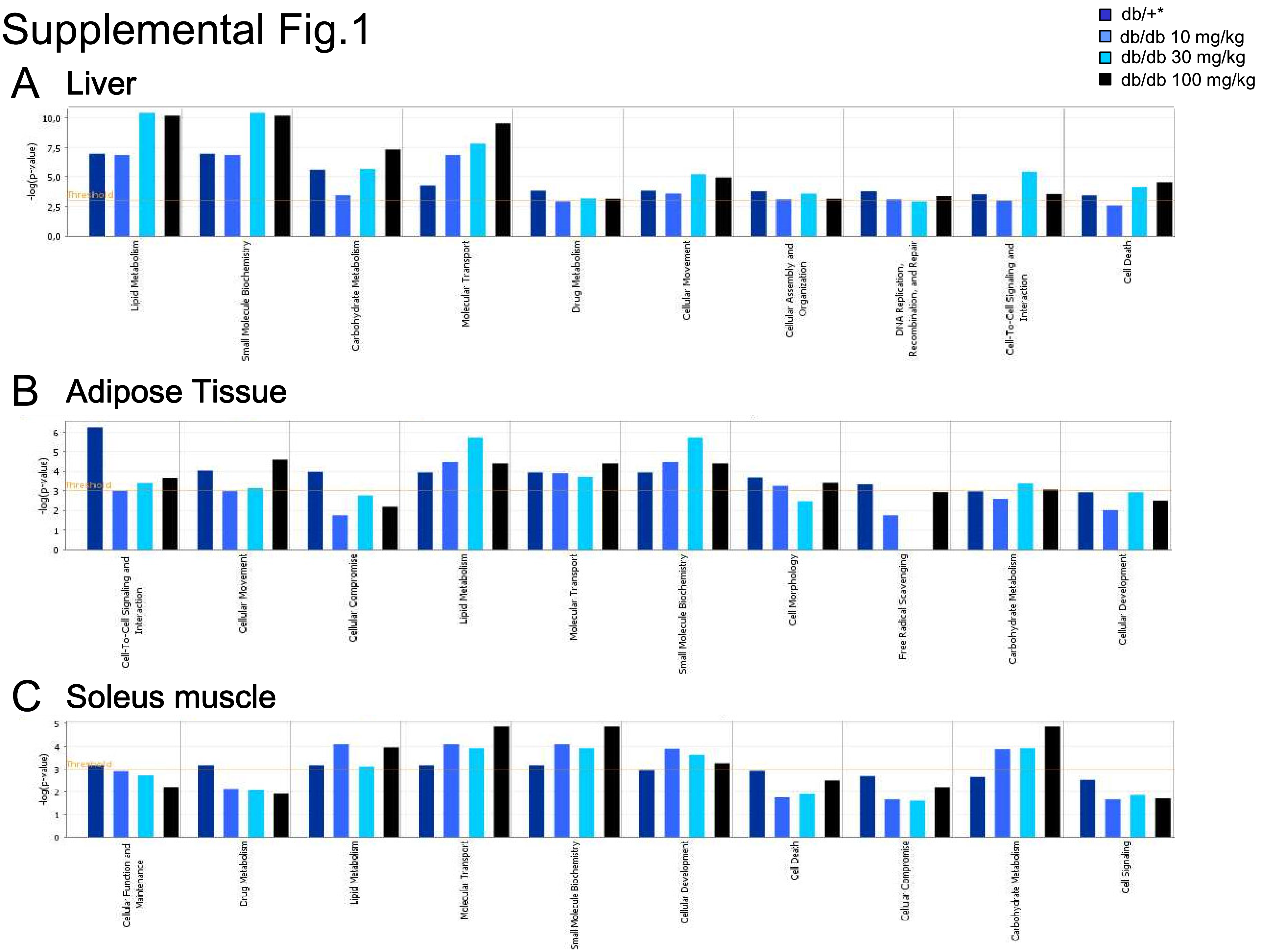

Supplemental Fig. 2A

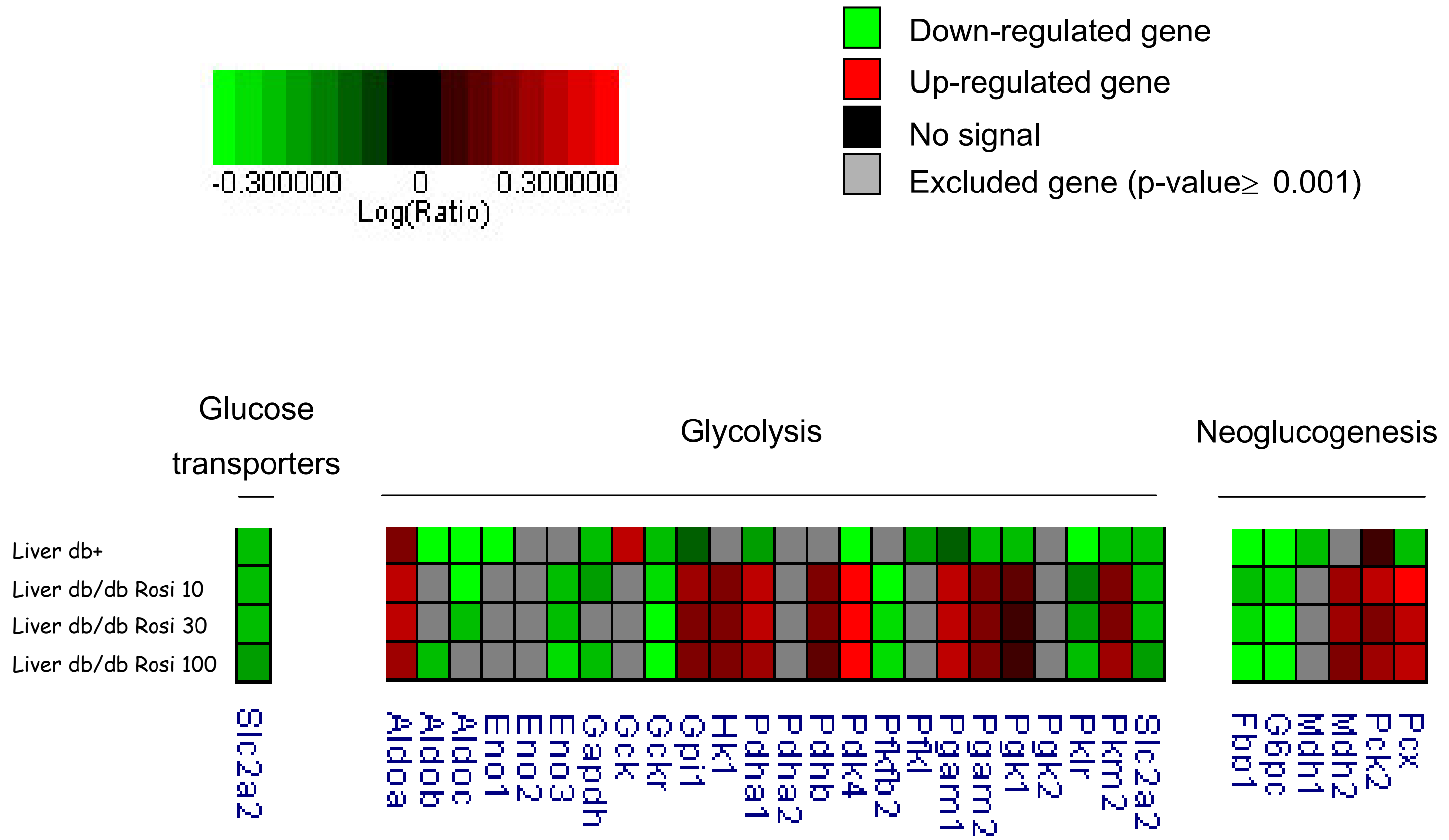

Supplemental Fig. 2B

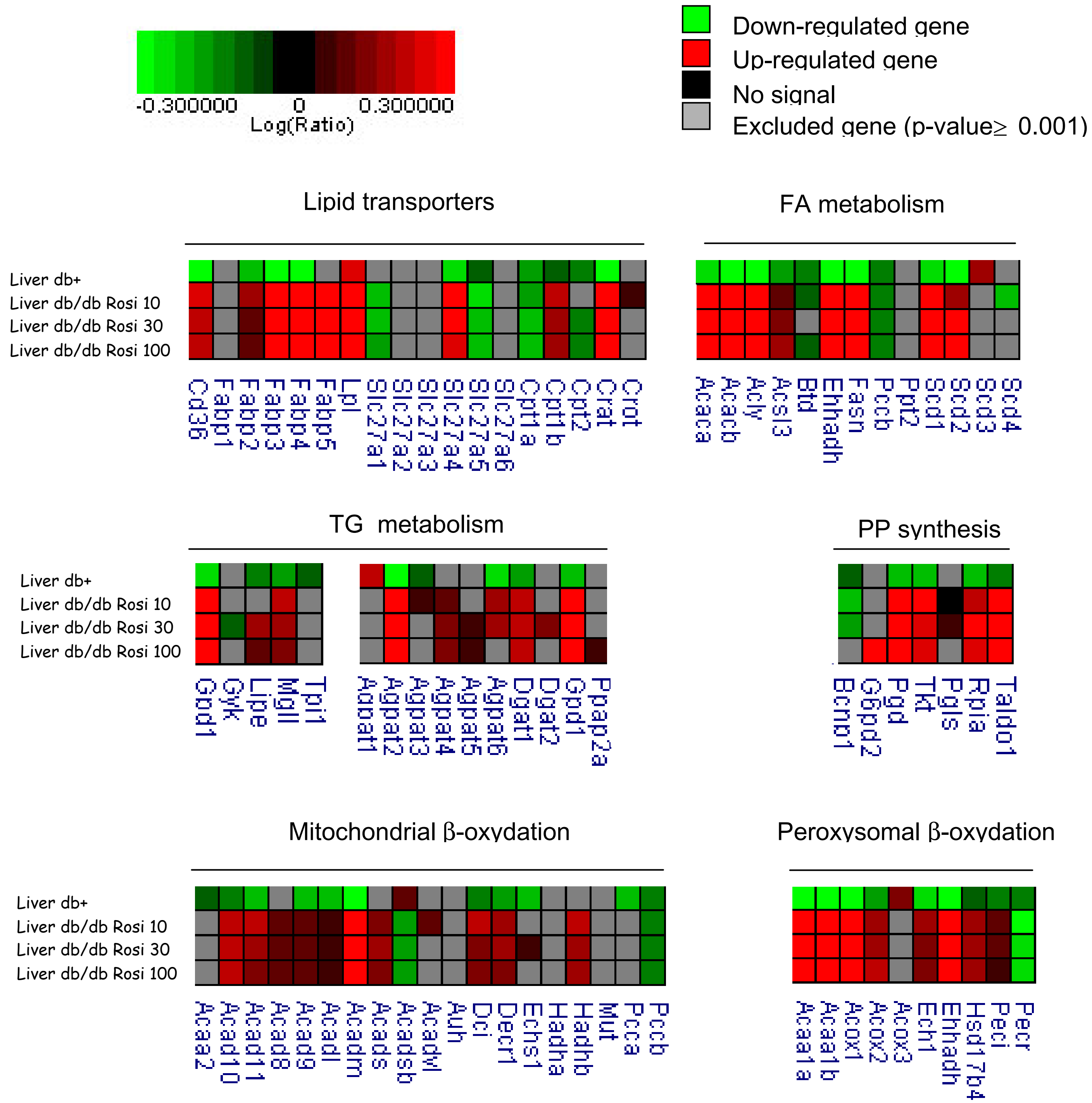

Supplemental Fig. 3

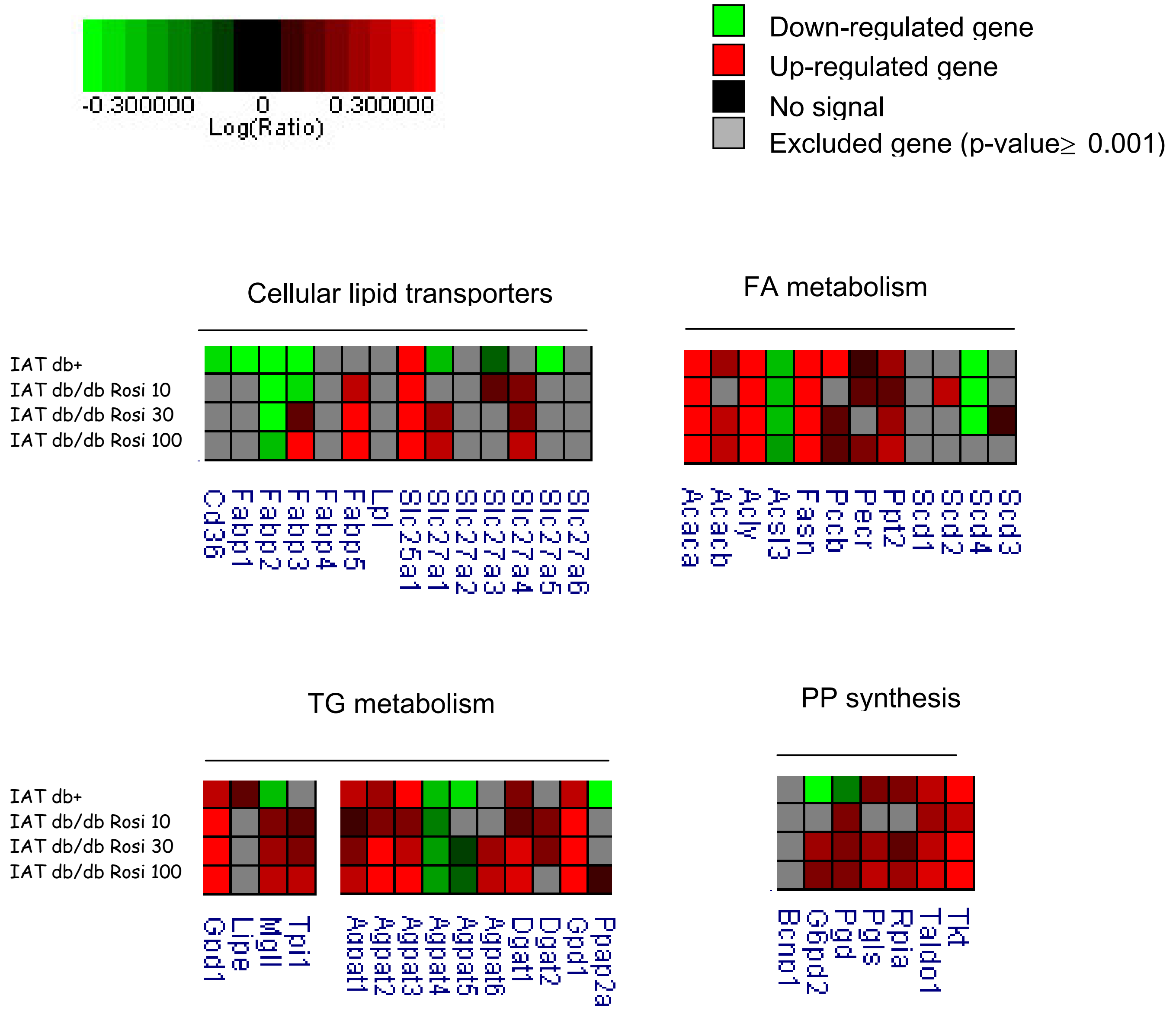

Supplemental Fig. 4

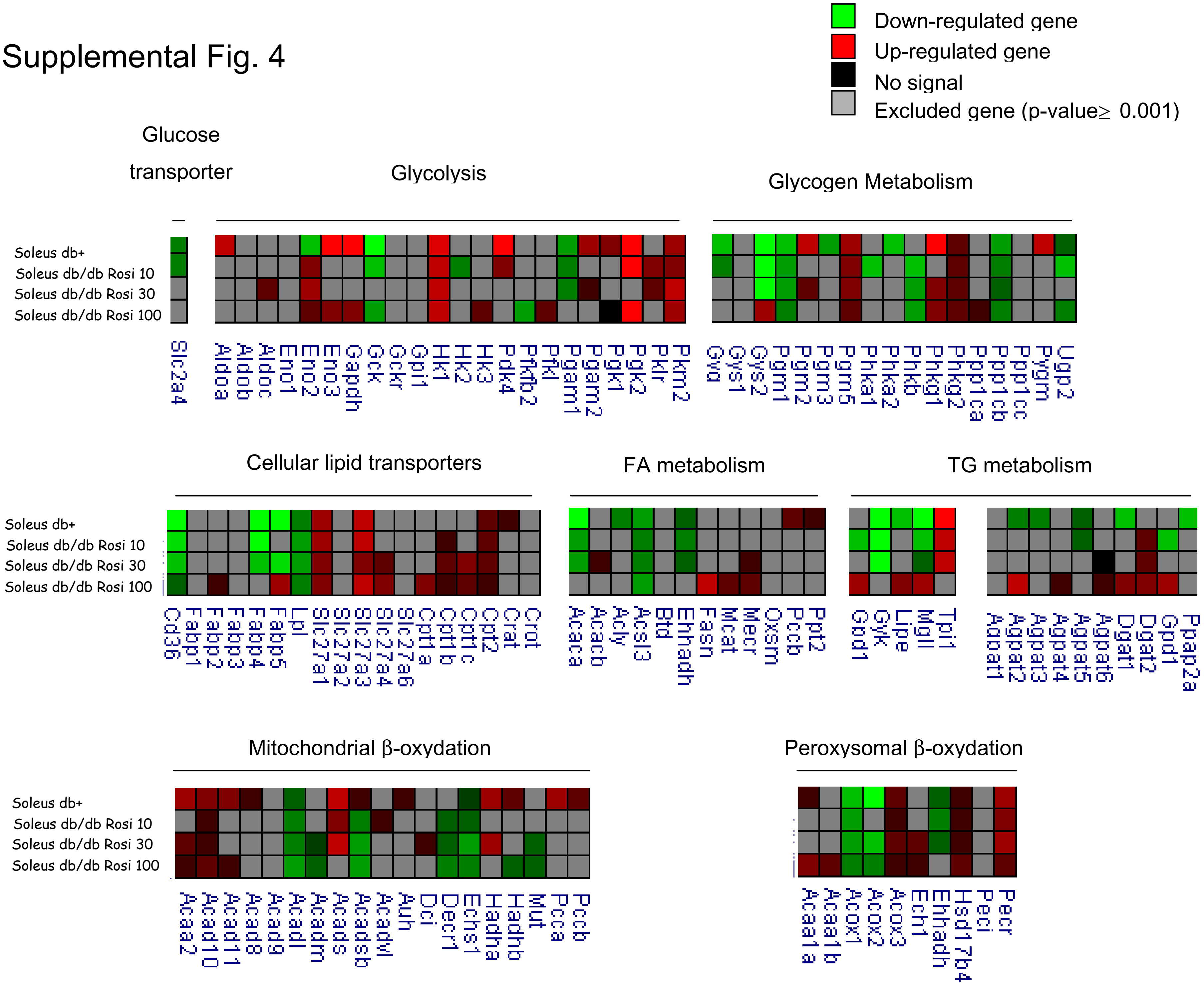

Supplemental Table 1

Overview of the number of sequences up- and down-regulated in db/+ and RSG-treated samples compared to db/db

Treatment vs db/db	**Liver**			**Inguinal Adipose Tissue**			**Soleus**		
	Up-regulated	Down-regulated	Total	Up-regulated	Down-regulated	Total	Up-regulated	Down-regulated	Total
db/+[*]	3317	3222	6539	7249	5958	13207	3817	4078	7895
db/db Rosi 10 mg/kg	2795	2772	5567	3146	2564	5710	1393	2020	3413
db/db Rosi 30 mg/kg	2385	2403	4788	5080	3925	9005	1641	2003	3644
db/db Rosi 100 mg/kg	2401	2148	4549	5766	4569	10335	1417	2203	3620

Number of sequences with differential expression of at least ± 1.3-fold and with p-value $p<0.001$.

Supplemental Table 2: Differentially expressed genes (506 sequences probes) involved in lipid and carbohydrate metabolism. * Resolver unweighted average and microarray probes selected in Fig. 1, 4, 6. When several probes exist for the same gene, selection was based on quality control (signal intensity and spot quality). In grey : sequences that do not pass the statistic and /or fold change thresholds (see Material and Methods). F.C. Fold Change; *p*-value = 0 corresponds to *p*-value < 1E-45.

Tissue	Sequence Name	Sequence Code	Accession #	Sequence description	db+ ratio	db+ P-value	RSG 10MK ratio	RSG 10MK P-value	RSG 30MK ratio	RSG 30MK P-value	RSG 100MK ratio	RSG 100MK P-value
**Liver**	Acaa1a	A_51_P327075	NM_130864	acetyl-Coenzyme A acyltransferase 1A	0.75	1.74E-08	1.94	8.88E-23	1.97	7.62E-22	1.86	1.68E-19
	Acaa1a	A_52_P155990	NM_130864	acetyl-Coenzyme A acyltransferase 1A	0.60	9.07E-10	3.26	0	3.14	0	2.80	0
	Acaa1a	A_52_P423183	NM_130864	acetyl-Coenzyme A acyltransferase 1A	0.54	7.85E-44	3.76	0	3.85	0	3.62	0
	Acaa1b	A_52_P423174	NM_146230	acetyl-Coenzyme A acyltransferase 1B	0.57	0	3.51	4.22E-34	3.42	0	3.05	0
	Acaca	A_51_P439426	NM_133360	acetyl-Coenzyme A carboxylase alpha	0.81	0.037202	3.74	1.40E-45	3.22	1.75E-18	3.41	0
	Acaca	A_52_P132591	AF374167	acetyl-Coenzyme A carboxylase alpha	0.67	0.000031	2.14	1.22E-19	2.16	1.86E-15	2.03	1.21E-12
	Acaca	A_52_P23177	NM_133360	acetyl-Coenzyme A carboxylase alpha	0.97	0.194564	1.75	6.47E-15	1.98	0	1.68	8.13E-37
	Acaca	A_52_P318040	AK132628	acetyl-Coenzyme A carboxylase alpha	0.63	1.60E-21	3.49	4.16E-43	3.43	7.43E-27	3.32	0
	*Acaca	A_52_P595124	NM_133360	acetyl-Coenzyme A carboxylase alpha	0.89	0.074292	1.87	5.47E-24	2.22	0	1.74	3.58E-23
	Acacb	A_51_P239236	BC022940	acetyl-Coenzyme A carboxylase beta	0.58	1.73E-30	2.40	0	2.49	0	2.55	0
	*Acacb	A_52_P340167	NM_133904	acetyl-Coenzyme A carboxylase beta	0.75	0.000015	1.94	0	2.32	2.35E-08	1.71	4.27E-15
	Acad10	A_51_P187507	NM_028037	acyl-Coenzyme A dehydrogenase family, member 10	0.84	2.26E-15	1.46	0	1.51	0	1.49	0
	Acad11	A_52_P85152	NM_175324	acyl-Coenzyme A dehydrogenase family, member 11	0.75	1.03E-28	1.47	0	1.39	2.19E-28	1.30	5.16E-19
	Acad9	A_51_P341379	NM_172678	acyl-Coenzyme A dehydrogenase family, member 9	0.65	0	1.20	6.77E-26	1.16	1.97E-08	1.13	6.78E-07
	Acadl	A_51_P149455	NM_007381	acyl-Coenzyme A dehydrogenase, long-chain	0.69	2.80E-45	1.13	2.09E-07	1.09	1.61E-11	1.09	1.13E-08
	Acadm	A_51_P319879	NM_007382	acyl-Coenzyme A dehydrogenase, medium chain	0.56	0	1.95	0	1.82	0	1.72	0
	Acads	A_52_P367745	NM_007383	acyl-Coenzyme A dehydrogenase, short chain	0.84	0.024186	1.31	6.11E-06	1.35	7.24E-07	1.34	2.43E-06
	Acat1	A_51_P319449	NM_144784	acetyl-Coenzyme A acetyltransferase 1	0.68	1.36E-27	1.08	0.053153	1.00	0.9906	1.05	0.348222
	Acat3	A_51_P139745	NM_153151	acetyl-Coenzyme A acetyltransferase 3	0.96	0.049656	0.84	6.41E-07	0.77	1.11E-37	0.77	0
	Acat3	A_52_P11959	NM_153151	acetyl-Coenzyme A acetyltransferase 3	0.86	0.010657	0.82	0.000034	0.76	1.42E-08	0.73	2.78E-09
	*Acly	A_51_P261718	NM_134037	ATP citrate lyase	0.55	0	2.24	1.54E-14	2.20	1.86E-35	2.01	1.26E-22
	Acly	A_52_P373556	AK043466	ATP citrate lyase	0.82	0.282286	1.80	3.81E-22	1.64	0.00013	1.72	5.91E-08
	Aco2	A_52_P200359	NM_080633	aconitase 2, mitochondrial	0.83	2.41E-11	1.41	2.75E-26	1.37	5.17E-23	1.16	0.000202

Acox1	A_51_P366704	NM_015729	acyl-Coenzyme A oxidase 1, palmitoyl	0.59	7.02E-15	2.12	5.14E-11	2.04	1.82E-09	2.16	5.79E-12
Acox1	A_52_P615096	NM_015729	acyl-Coenzyme A oxidase 1, palmitoyl	0.59	2.27E-16	2.29	5.47E-43	2.22	0	2.26	0
Acox2	A_51_P206708	NM_053115	acyl-Coenzyme A oxidase 2, branched chain	0.76	3.60E-08	1.38	0	1.37	5.64E-25	1.40	2.77E-35
Acsl1	A_51_P463452	NM_007981	acyl-CoA synthetase long-chain family member 1	1.25	8.55E-17	2.48	0	2.69	0	2.15	0
Acsl1	A_51_P496432	BC006692	acyl-CoA synthetase long-chain family member 1	1.19	2.18E-16	2.36	0	2.51	0	2.14	0
Acsl1	A_52_P597618	NM_007981	acyl-CoA synthetase long-chain family member 1	1.17	2.65E-13	2.42	0	2.63	0	2.15	0
Acsl3	A_51_P511560	NM_028817	acyl-CoA synthetase long-chain family member 3	0.75	9.90E-07	1.22	6.74E-07	1.25	0.000285	1.32	1.37E-10
Acsl4	A_51_P268154	NM_019477	acyl-CoA synthetase long-chain family member 4	1.43	5.20E-22	1.88	4.13E-43	1.95	0	2.11	2.80E-45
Acsl4	A_52_P78203	NM_207625	acyl-CoA synthetase long-chain family member 4	1.04	0.179276	1.55	0	1.60	0	1.69	0
Acsl5	A_52_P593037	NM_027976	acyl-CoA synthetase long-chain family member 5	0.68	3.91E-32	1.81	0	1.80	0	1.79	0
Acsl6	A_51_P380699	NM_144823	acyl-CoA synthetase long-chain family member 6	3.08	2.24E-10	1.00	1	1.00	1	1.00	1
Adcy1	A_52_P240164	NM_009622	adenylate cyclase 1	1.39	9.03E-09	0.91	0.422411	0.89	0.152402	0.93	0.187814
Adcy1	A_52_P240170	BC050125	adenylate cyclase 1	1.33	1.40E-45	0.72	4.40E-18	0.68	2.23E-27	0.75	8.99E-28
Adcy4	A_51_P229911	NM_080435	adenylate cyclase 4	0.95	0.073846	1.12	0.00004	1.32	7.03E-07	1.14	8.44E-07
Adcy9	A_51_P501538	NM_009624	adenylate cyclase 9	1.23	7.23E-13	0.64	2.98E-43	0.76	1.25E-13	0.73	7.16E-12
Adh1	A_52_P629895	NM_007409	alcohol dehydrogenase 1 (class I)	0.91	0.152117	1.18	0.011872	1.22	0.004316	1.19	0.00505
Adh4	A_51_P189442	NM_011996	alcohol dehydrogenase 4 (class II), pi polypeptide	1.35	1.21E-10	0.48	7.43E-44	0.45	0	0.53	1.32E-32
Adh6-ps1	A_51_P120027	AK004863	alcohol dehydrogenase 6 (class V), pseudogene 1	24.38	0	1.00	1	1.00	1	1.03	0.77865
Adh7	A_51_P233797	NM_009626	alcohol dehydrogenase 7 (class IV), mu or sigma polypeptide	0.83	0.002635	1.51	5.03E-12	1.51	3.60E-17	1.51	1.18E-41
Agpat1	A_51_P322612	NM_018862	1-acylglycerol-3-phosphate O-acyltransferase 1 (lysophosphatidic acid acyltransferase, alpha)	1.45	1.28E-41	1.00	0.871515	1.02	0.547802	0.99	0.802776
Agpat2	A_51_P238563	NM_026212	1-acylglycerol-3-phosphate O-acyltransferase 2 (lysophosphatidic acid acyltransferase, beta)	0.57	2.72E-11	2.09	0	2.04	0	1.98	0
*Agpat2	A_51_P238565	NM_026212	1-acylglycerol-3-phosphate O-acyltransferase 2 (lysophosphatidic acid acyltransferase, beta)	0.60	6.53E-13	1.99	0	2.09	0	1.80	0
Agpat5	A_51_P361286	NM_026792	1-acylglycerol-3-phosphate O-acyltransferase 5 (lysophosphatidic acid acyltransferase, epsilon)	1.31	3.40E-07	1.01	0.877538	1.11	0.140076	1.05	0.36821
*Agpat6	A_51_P335710	NM_018743	1-acylglycerol-3-phosphate O-acyltransferase 6 (lysophosphatidic acid acyltransferase, zeta)	0.58	4.12E-06	1.44	0.000494	1.45	0.000525	1.28	0.240079
Agpat6	A_51_P335716	NM_018743	1-acylglycerol-3-phosphate O-acyltransferase 6 (lysophosphatidic acid acyltransferase, zeta)	0.66	2.80E-10	1.44	4.63E-09	1.41	1.58E-08	1.21	0.007527
Agpat6	A_52_P24986	NM_018743	1-acylglycerol-3-phosphate O-acyltransferase 6 (lysophosphatidic acid acyltransferase, zeta)	0.58	0	1.23	1.45E-19	1.42	0	1.19	6.93E-14
Aldh1a1	A_51_P334942	NM_013467	aldehyde dehydrogenase family 1, subfamily A1	0.55	1.35E-13	0.81	0.012059	0.81	0.000799	0.76	0.000033
Aldh1a3	A_52_P87843	NM_053080	aldehyde dehydrogenase family 1, subfamily A3	0.37	0	0.59	4.73E-19	0.59	8.51E-17	0.62	5.20E-39
Aldh1a7	A_51_P383399	NM_011921	aldehyde dehydrogenase family 1, subfamily A7	0.61	0	1.36	4.01E-23	1.21	4.30E-22	1.27	0
Aldh1b1	A_51_P510418	NM_028270	aldehyde dehydrogenase 1 family, member B1	0.52	0	1.31	2.60E-09	1.24	1.54E-13	1.17	6.97E-08
Aldh2	A_52_P13109	AK163452	aldehyde dehydrogenase 2, mitochondrial	0.57	0	0.64	1.52E-24	0.64	3.70E-25	0.64	0
Aldh3a2	A_51_P464175	NM_007437	aldehyde dehydrogenase family 3, subfamily A2	0.34	0	2.12	0	1.95	0	1.71	0

Aldh4a1	A_51_P337918	NM_175438	aldehyde dehydrogenase 4 family, member A1	0.75	1.61E-27	0.95	0.16603	1.04	0.071561	1.04	0.154621
Aldh7a1	A_51_P470414	NM_138600	aldehyde dehydrogenase family 7, member A1	0.78	1.55E-13	0.76	5.31E-13	0.87	0.002202	0.70	1.12E-29
Aldh9a1	A_51_P106211	NM_019993	aldehyde dehydrogenase 9, subfamily A1	0.77	6.44E-13	1.10	0.000079	1.09	0.001279	1.06	0.032171
Aldh9a1	A_52_P265877	NM_019993	aldehyde dehydrogenase 9, subfamily A1	0.70	0	0.96	0.34659	1.01	0.723912	1.03	0.321975
Aldoa	A_51_P216905	NM_007438	aldolase 1, A isoform	1.33	1.29E-12	1.54	4.15E-40	1.54	4.20E-45	1.45	2.80E-31
*Aldoa	A_52_P98387	NM_007438	aldolase 1, A isoform	1.32	3.78E-17	1.53	0	1.56	0	1.40	0
*Aldob	A_51_P337269	NM_144903	aldolase 2, B isoform	0.53	6.33E-13	0.98	0.813021	0.80	0.002103	0.69	9.81E-11
Aldob	A_52_P450038	NM_144903	aldolase 2, B isoform	0.57	0	0.98	0.501143	0.79	3.75E-19	0.71	3.61E-27
Aldob	A_52_P468023	NM_144903	aldolase 2, B isoform	0.53	0	0.92	0.004378	0.78	8.51E-26	0.70	1.80E-31
Aldoc	A_51_P220681	NM_009657	aldolase 3, C isoform	0.59	2.74E-28	0.53	0	0.66	2.73E-23	0.85	0.032285
Aldoc	A_51_P425284	AK039267	aldolase 3, C isoform	0.35	1.53E-17	0.50	1.57E-23	0.69	9.15E-09	0.78	0.001372
Apoa1	A_51_P408082	NM_009692	apolipoprotein A-I	4.85	0	1.84	6.15E-35	1.93	4.42E-36	2.69	0
Apoa4	A_51_P327491	NM_007468	apolipoprotein A-IV	0.24	0	1.32	0.003995	0.64	1.73E-13	0.61	2.79E-13
Apoa4	A_51_P327496	NM_007468	apolipoprotein A-IV	0.28	0	1.22	0.048667	0.58	3.54E-14	0.57	1.06E-06
Apoa5	A_51_P259930	NM_080434	apolipoprotein A-V	0.69	3.83E-08	0.46	0	0.40	0	0.46	0
Apoa5	A_52_P292404	NM_080434	apolipoprotein A-V	0.69	3.38E-13	0.53	0	0.49	0	0.56	7.50E-42
Apoc2	A_51_P334979	NM_009695	apolipoprotein C-II	0.76	4.35E-15	1.24	4.78E-17	1.04	0.041548	0.96	0.079965
*Apoc3	A_51_P310629	NM_023114	apolipoprotein C-III	0.83	5.90E-18	0.72	9.30E-27	0.70	3.21E-35	0.70	0
Apoc3	A_52_P574306	NM_023114	apolipoprotein C-III	0.85	2.42E-22	0.74	1.31E-30	0.72	9.39E-43	0.71	0
Atp12a	A_52_P329398	NM_138652	ATPase, H+/K+ transporting, nongastric, alpha polypeptide	7.65	0	1.10	0.157139	1.21	0.001529	1.18	0.000915
Atp6v1h	A_51_P105604	NM_133826	ATPase, H+ transporting, lysosomal V1 subunit H	0.81	1.17E-13	1.20	5.02E-21	1.20	2.00E-08	1.64	1.16E-06
Auh	A_52_P107290	AK003929	AU RNA binding protein/enoyl-coenzyme A hydratase	1.05	0.295136	1.35	1.96E-09	1.65	0	1.36	1.08E-09
Bcnp1	A_52_P1053160	AK158308	BCNP1 homolog	0.88	0.000934	0.73	8.08E-07	0.80	0.000151	0.84	0.000814
Bdh2	A_51_P470935	NM_027208	3-hydroxybutyrate dehydrogenase, type 2	1.09	0.034396	0.62	0	0.59	3.12E-36	0.68	1.50E-22
Bdh2	A_52_P195809	NM_027208	3-hydroxybutyrate dehydrogenase, type 2	1.03	0.352063	0.61	0	0.64	9.90E-28	0.67	1.04E-30
Cd36	A_51_P375138	L23108	CD36 antigen	0.09	0	1.80	0	1.76	0	1.82	0
*Cd36	A_51_P375146	NM_007643	CD36 antigen	0.08	0	1.62	0	1.69	0	1.60	1.09E-14
Cox6b2	A_51_P300506	NM_183405	cytochrome c oxidase subunit VIb polypeptide 2	1.00	0.989976	1.34	3.97E-19	1.27	2.59E-16	1.23	6.70E-13
Cox7a1	A_51_P148612	NM_009944	cytochrome c oxidase, subunit VIIa 1	1.25	7.57E-10	2.57	0	2.27	0	2.30	0
Cpt1a	A_51_P427674	NM_013495	carnitine palmitoyltransferase 1a, liver	0.85	0.002288	0.79	9.77E-06	0.72	4.91E-23	0.73	1.42E-24
Cpt1a	A_52_P219753	NM_013495	carnitine palmitoyltransferase 1a, liver	0.78	4.06E-07	0.79	0.000028	0.67	1.66E-27	0.69	2.75E-22
Cpt1b	A_51_P232913	NM_009948	carnitine palmitoyltransferase 1b, muscle	0.90	0.000156	1.55	2.84E-13	1.37	2.25E-24	1.39	6.61E-19
Cpt1b	A_52_P625508	NM_009948	carnitine palmitoyltransferase 1b, muscle	0.55	2.96E-08	2.04	3.23E-28	1.55	8.90E-09	2.05	1.84E-42
Cpt1c	A_51_P177562	NM_153679	carnitine palmitoyltransferase 1c	1.00	1	1.00	1	0.93	0.561109	1.00	1
Crat	A_51_P440807	NM_007760	carnitine acetyltransferase	0.43	0	2.66	0	2.53	0	2.22	0

Cs	A_51_P390260	NM_026444	citrate synthase	1.24	2.36E-08	1.50	4.20E-43	1.39	7.41E-18	1.44	1.12E-15
Cs	A_52_P182659	NM_026444	citrate synthase	0.96	0.118852	1.47	3.24E-28	1.40	1.36E-20	1.33	1.92E-34
Cyp1a1	A_51_P279693	NM_009992	cytochrome P450, family 1, subfamily a, polypeptide 1	0.68	0.000011	0.83	0.134841	1.00	0.990557	0.91	0.262586
Cyp1a2	A_51_P450140	NM_009993	cytochrome P450, family 1, subfamily a, polypeptide 2	2.42	0	0.37	0	0.42	0	0.58	5.78E-23
Cyp1a2	A_52_P595871	NM_009993	cytochrome P450, family 1, subfamily a, polypeptide 2	2.46	0	0.34	0	0.41	0	0.58	0
Cyp1b1	A_51_P255456	NM_009994	cytochrome P450, family 1, subfamily b, polypeptide 1	0.59	0.000425	1.13	0.321098	1.13	0.600004	0.95	0.684035
Cyp2a12	A_51_P206736	NM_133657	cytochrome P450, family 2, subfamily a, polypeptide 12	0.81	2.21E-08	0.72	7.20E-18	0.67	5.62E-22	0.72	8.30E-15
Cyp2a5	A_52_P246252	NM_007812	cytochrome P450, family 2, subfamily a, polypeptide 5	0.48	1.97E-08	0.20	0	0.25	0	0.35	1.82E-09
Cyp2b10	A_52_P472486	NM_009999	cytochrome P450, family 2, subfamily b, polypeptide 10	0.04	0	0.60	4.00E-15	0.62	1.26E-37	0.66	4.76E-11
Cyp2b13	A_51_P492339	NM_007813	cytochrome P450, family 2, subfamily b, polypeptide 13	0.07	0	0.83	2.77E-08	0.83	0.000075	0.79	1.99E-06
Cyp2b13	A_52_P289091	NM_007813	cytochrome P450, family 2, subfamily b, polypeptide 13	0.06	0	0.51	8.94E-12	0.45	1.74E-30	0.49	1.51E-19
Cyp2c29	A_51_P103706	NM_007815	cytochrome P450, family 2, subfamily c, polypeptide 29	0.96	0.072755	0.30	0	0.37	0	0.46	7.98E-23
Cyp2c37	A_51_P498882	NM_010001	cytochrome P450, family 2. subfamily c, polypeptide 37	0.69	2.30E-20	0.23	0	0.32	0	0.39	4.71E-28
Cyp2c37	A_52_P625249	NM_010001	cytochrome P450, family 2. subfamily c, polypeptide 37	0.65	8.30E-30	0.22	0	0.30	0	0.37	8.70E-34
Cyp2c38	A_51_P342206	NM_010002	cytochrome P450, family 2, subfamily c, polypeptide 38	0.15	0	0.86	2.74E-07	0.82	2.15E-10	0.80	9.63E-08
Cyp2c38	A_52_P468564	NM_010002	cytochrome P450, family 2, subfamily c, polypeptide 38	0.12	0	0.72	7.49E-26	0.70	1.28E-20	0.64	0
Cyp2c39	A_51_P304109	NM_010003	cytochrome P450, family 2, subfamily c, polypeptide 39	0.73	5.65E-16	0.34	0	0.40	0	0.47	1.38E-35
Cyp2c44	A_51_P209782	NM_001001446	cytochrome P450, family 2, subfamily c, polypeptide 44	1.55	6.48E-37	0.45	0	0.52	1.58E-26	0.65	1.24E-14
Cyp2c54	A_52_P154580	NM_206537	cytochrome P450, family 2, subfamily c, polypeptide 54	0.58	3.52E-29	0.19	0	0.28	0	0.36	1.26E-32
Cyp2c55	A_51_P447785	NM_028089	cytochrome P450, family 2, subfamily c, polypeptide 55	1.05	0.327253	3.20	0	3.21	0	4.19	0
Cyp2c70	A_51_P134142	NM_145499	cytochrome P450, family 2, subfamily c, polypeptide 70	4.53	0	0.62	8.27E-30	1.08	0.183788	1.22	0.000753
Cyp2d10	A_51_P111192	NM_010005	cytochrome P450, family 2, subfamily d, polypeptide 10	1.19	2.69E-21	0.72	0	0.74	0	0.74	0
Cyp2d10	A_52_P527775	NM_010005	cytochrome P450, family 2, subfamily d, polypeptide 10	1.28	8.83E-17	0.63	0	0.62	0	0.61	0
Cyp2d13	A_52_P372062	NM_133695	cytochrome P450, family 2, subfamily d, polypeptide 13 (Cyp2d13) on chromosome 15.	1.78	0	0.53	0	0.56	0	0.55	0
Cyp2d22	A_52_P478420	AK086555	cytochrome P450, family 2, subfamily d, polypeptide 22	0.94	0.159817	0.64	0	0.68	1.35E-14	0.69	2.53E-31
Cyp2d22	A_52_P49391	AK090296	cytochrome P450, family 2, subfamily d, polypeptide 22	0.76	5.32E-30	0.68	0	0.68	0	0.73	3.36E-29
Cyp2d9	A_51_P124133	NM_010006	cytochrome P450, family 2, subfamily d, polypeptide 9	1.47	1.13E-22	0.73	7.26E-11	0.75	4.53E-17	0.78	4.80E-10
Cyp2d9	A_51_P478303	NM_010006	cytochrome P450, family 2, subfamily d, polypeptide 9	1.60	9.81E-45	0.75	3.13E-13	0.77	5.65E-06	0.77	1.89E-11
Cyp2f2	A_51_P453909	NM_007817	cytochrome P450, family 2, subfamily f, polypeptide 2	1.45	9.69E-16	0.93	0.074673	0.92	0.032242	1.01	0.65503
Cyp2g1	A_51_P137452	NM_013809	cytochrome P450, family 2, subfamily g, polypeptide 1	0.86	0.052464	0.23	0	0.25	1.16E-42	0.28	1.99E-22
Cyp2j6	A_51_P506328	NM_010008	cytochrome P450, family 2, subfamily j, polypeptide 6	1.17	7.20E-14	1.41	3.62E-21	1.36	5.73E-14	1.31	3.75E-10
Cyp2j9	A_51_P330044	NM_028979	cytochrome P450, family 2, subfamily j, polypeptide 9	0.63	0	0.48	2.40E-36	0.57	1.44E-15	0.53	0
Cyp3a13	A_51_P114941	NM_007819	cytochrome P450, family 3, subfamily a, polypeptide 13	0.49	0	0.56	1.03E-41	0.64	6.41E-37	0.67	5.69E-30
Cyp3a25	A_51_P489367	NM_019792	cytochrome P450, family 3, subfamily a, polypeptide 25	0.75	3.44E-09	0.45	0	0.51	0	0.50	0

Cyp3a44	A_52_P366803	NM_177380	cytochrome P450, family 3, subfamily a, polypeptide 44	0.56	9.81E-23	0.61	1.34E-17	0.70	3.18E-22	0.83	0.000068
Cyp4a10	A_52_P257774	NM_010011	cytochrome P450, family 4, subfamily a, polypeptide 10	0.23	0	0.83	0.000044	0.92	0.113012	1.07	0.227687
Cyp4a10	A_52_P340136	X71478	cytochrome P450, family 4, subfamily a, polypeptide 10	0.35	0	0.99	0.849133	1.01	0.946687	1.08	0.326539
Cyp4a10	A_52_P6588	NM_010011	cytochrome P450, family 4, subfamily a, polypeptide 10	0.11	0	0.89	0.171495	0.94	0.546311	1.12	0.246269
Cyp4a12a	A_51_P433360	NM_177406	cytochrome P450, family 4, subfamily a, polypeptide 12a	0.38	0	1.16	2.65E-08	1.18	0.000523	1.25	1.90E-10
Cyp4a12a	A_52_P44914	NM_177406	cytochrome P450, family 4, subfamily a, polypeptide 12a	0.91	0.030951	1.41	3.16E-13	1.26	0.004666	1.34	2.30E-09
Cyp4a14	A_51_P238576	NM_007822	cytochrome P450, family 4, subfamily a, polypeptide 14	0.03	0	1.13	0.122563	1.22	0.009042	1.39	2.67E-06
Cyp4b1	A_51_P118704	NM_007823	cytochrome P450, family 4, subfamily b, polypeptide 1	0.95	0.566855	1.20	5.75E-08	1.28	1.16E-09	1.25	2.98E-06
Cyp4f14	A_51_P452768	NM_022434	cytochrome P450, family 4, subfamily f, polypeptide 14	1.64	6.22E-33	0.29	0	0.39	0	0.36	0
Cyp4f15	A_51_P136303	NM_134127	cytochrome P450, family 4, subfamily f, polypeptide 15	0.90	0.000799	0.67	0	0.75	0	0.79	3.21E-29
Cyp51	A_51_P485791	NM_020010	cytochrome P450, family 51	0.72	0.005145	0.95	0.4062	1.05	0.494765	1.34	1.47E-10
Cyp51	A_52_P164161	NM_020010	cytochrome P450, family 51	0.65	0.0007	1.00	0.999137	1.03	0.669876	1.30	2.20E-11
Dci	A_51_P105589	NM_010023	dodecenoyl-Coenzyme A delta isomerase (3,2 trans-enoyl-Coenyme A isomerase)	0.83	2.16E-18	1.45	0	1.30	4.43E-35	1.29	1.03E-27
Decr1	A_51_P208555	NM_026172	2,4-dienoyl CoA reductase 1, mitochondrial	0.82	8.62E-19	1.39	2.04E-30	1.27	8.78E-29	1.26	9.98E-12
Decr1	A_52_P335907	NM_026172	2,4-dienoyl CoA reductase 1, mitochondrial	0.75	4.92E-23	1.43	0	1.35	4.42E-19	1.27	1.38E-08
Dgat1	A_51_P510059	NM_010046	diacylglycerol O-acyltransferase 1	0.81	6.90E-07	1.58	2.43E-20	1.64	9.08E-16	1.40	3.07E-10
Dgat1	A_52_P456134	NM_010046	diacylglycerol O-acyltransferase 1	0.80	2.10E-44	1.51	0	1.51	4.55E-26	1.40	3.38E-43
Dgat2	A_52_P592909	NM_026384	diacylglycerol O-acyltransferase 2	0.88	0.005614	1.11	0.001209	1.31	3.25E-12	1.06	0.011493
Dlat	A_51_P265106	NM_145614	dihydrolipoamide S-acetyltransferase (E2 component of pyruvate dehydrogenase complex)	0.88	0.000033	1.92	0	1.91	0	1.75	0
Dlat	A_52_P374642	NM_145614	dihydrolipoamide S-acetyltransferase (E2 component of pyruvate dehydrogenase complex)	0.89	0.000354	1.83	1.78E-36	1.93	0	1.80	3.10E-39
Dld	A_51_P184284	NM_007861	dihydrolipoamide dehydrogenase	0.97	0.406823	1.32	2.94E-25	1.25	7.94E-17	1.24	6.08E-09
Dlst	A_51_P290139	NM_030225	dihydrolipoamide S-succinyltransferase (E2 component of 2-oxo-glutarate complex)	0.84	1.78E-06	1.37	6.53E-22	1.37	2.55E-38	1.24	2.10E-08
Dlst	A_52_P140072	NM_030225	dihydrolipoamide S-succinyltransferase (E2 component of 2-oxo-glutarate complex)	0.82	0.009767	1.40	4.54E-07	1.38	0.000013	1.29	0.006086
Ech1	A_51_P421846	NM_016772	enoyl coenzyme A hydratase 1, peroxisomal	0.61	0	1.35	3.52E-27	1.32	2.28E-20	1.28	6.85E-13
Echs1	A_51_P409039	NM_053119	enoyl Coenzyme A hydratase, short chain, 1, mitochondrial	0.74	0	1.08	0.002361	1.10	6.03E-13	1.08	0.000037
Ehhadh	A_51_P462918	NM_023737	enoyl-Coenzyme A, hydratase/3-hydroxyacyl Coenzyme A dehydrogenase	0.59	0	2.36	0	2.59	0	3.17	0
Eno1	A_52_P349939	NM_023119	enolase 1, alpha non-neuron	0.60	1.86E-09	1.23	0.008352	1.08	0.411163	0.97	0.780398
Eno3	A_51_P328539	NM_007933	enolase 3, beta muscle	0.96	0.243778	0.67	9.70E-27	0.68	3.95E-17	0.64	1.01E-24
Fabp2	A_51_P313581	NM_007980	fatty acid binding protein 2, intestinal	0.65	0	1.38	2.27E-15	1.19	0.000048	1.13	0.013179
Fabp2	A_52_P453013	NM_007980	fatty acid binding protein 2, intestinal	0.57	0	1.30	5.72E-20	1.16	9.70E-08	1.10	0.055047
Fabp3	A_51_P167535	NM_010174	fatty acid binding protein 3, muscle and heart	0.56	1.14E-33	2.71	0	2.38	8.80E-24	1.82	2.40E-07
Fabp4	A_51_P336830	NM_024406	fatty acid binding protein 4, adipocyte	0.52	3.56E-26	8.96	0	10.73	0	9.87	3.98E-34

*Fabp4	A_51_P336833	NM_024406	fatty acid binding protein 4, adipocyte	0.53	0	9.70	0	13.46	0	12.13	4.09E-32
Fabp4	A_52_P564544	NM_024406	fatty acid binding protein 4, adipocyte	0.33	8.34E-30	10.50	0	12.57	0	14.53	0
Fabp5	A_51_P387764	NM_010634	fatty acid binding protein 5, epidermal	2.34	0.001486	14.61	0	7.69	0	16.17	0
Fasn	A_51_P321126	NM_007988	fatty acid synthase	0.38	1.09E-41	3.24	1.40E-45	4.22	1.25E-31	4.11	0
*Fasn	A_52_P100252	NM_007988	fatty acid synthase	0.37	2.88E-41	3.70	0	4.19	0	3.55	0
*Fbp1	A_51_P474701	NM_019395	fructose bisphosphatase 1	0.50	6.45E-18	0.71	0.000066	0.63	7.07E-10	0.51	3.16E-18
Fdft1	A_51_P485946	NM_010191	farnesyl diphosphate farnesyl transferase 1	0.54	1.41E-32	0.79	7.02E-14	0.86	0.006478	1.02	0.619059
Fdps	A_51_P379798	NM_134469	farnesyl diphosphate synthetase	0.68	6.93E-06	1.21	0.001048	1.52	3.11E-17	1.64	9.14E-14
Fdps	A_52_P593965	AK077979	farnesyl diphosphate synthetase	0.49	2.66E-16	1.39	6.62E-07	2.07	1.93E-17	2.04	0
Fh1	A_51_P359333	NM_010209	fumarate hydratase 1	0.76	3.03E-16	0.94	0.005397	0.92	0.002433	0.88	1.30E-17
Fntb	A_51_P517680	NM_145927	farnesyltransferase, CAAX box, beta	1.17	0.040671	1.45	1.85E-15	1.16	0.222952	1.02	0.748158
*G6pc	A_51_P462385	NM_008061	glucose-6-phosphatase, catalytic	0.46	0.000013	0.62	2.73E-23	0.59	1.48E-35	0.40	8.40E-39
G6pd2	A_51_P353735	NM_019468	glucose-6-phosphate dehydrogenase 2	1.00	1	1.53	0.178323	1.37	0.174128	2.96	9.02E-08
G6pdx	A_51_P187082	NM_008062	glucose-6-phosphate dehydrogenase X-linked	0.99	0.828139	2.64	0	4.05	0	3.72	0
*Gapdh	A_52_P589321	AK169742	glyceraldehyde-3-phosphate dehydrogenase	0.61	1.33E-07	0.77	0.000011	0.83	0.162769	0.72	9.59E-14
Gck	A_52_P66580	BC011139	glucokinase	1.49	6.85E-23	0.97	0.427453	0.87	0.001911	0.99	0.658739
Gckr	A_51_P198694	NM_144909	glucokinase regulatory protein	0.74	6.07E-14	0.61	3.32E-26	0.59	1.59E-26	0.55	2.43E-27
*Gpd1	A_51_P293853	NM_010271	glycerol-3-phosphate dehydrogenase 1 (soluble)	0.71	5.06E-15	2.59	0	2.39	0	1.97	8.59E-19
Gpd1	A_52_P16419	NM_010271	glycerol-3-phosphate dehydrogenase 1 (soluble)	0.64	3.11E-24	2.95	0	2.58	0	2.31	1.04E-32
Gpi1	A_51_P426886	NM_008155	glucose phosphate isomerase 1	0.91	4.96E-09	1.43	0	1.33	4.02E-25	1.26	4.93E-30
Gpt2	A_51_P493886	NM_173866	glutamic pyruvate transaminase (alanine aminotransferase) 2	0.43	0	0.93	0.02	0.90	0.00059	0.87	2.58E-06
Gyg	A_51_P240019	NM_013755	glycogenin	0.86	0.000102	1.25	4.40E-13	1.31	2.82E-11	1.29	8.31E-13
Gyk	A_52_P1020291	AK037633	glycerol kinase	1.00	0.997854	0.81	0.009583	0.75	0.001213	0.69	8.37E-07
Gyk	A_52_P379205	AK086200	Mus musculus 16 days neonate thymus cDNA	1.00	1	1.03	0.724656	1.00	1	1.03	0.799772
Gys2	A_51_P440315	NM_145572	glycogen synthase 2	1.12	0.022211	1.31	0	1.25	3.60E-42	1.21	1.36E-14
Hadhb	A_51_P217990	NM_145558	hydroxyacyl-Coenzyme A dehydrogenase/3-ketoacyl-Coenzyme A thiolase/enoyl-Coenzyme A hydratase beta subunit	0.94	0.03782	1.53	0	1.39	2.69E-31	1.32	2.82E-11
Hk1	A_52_P102773	BC072628	hexokinase 1	0.94	0.190425	1.27	2.14E-18	1.30	1.95E-15	1.25	1.40E-12
Hk1	A_52_P9337	NM_010438	hexokinase 1	1.49	6.69E-08	1.15	0.548976	0.91	0.508841	1.09	0.531832
Hmgcl	A_51_P308961	NM_008254	3-hydroxy-3-methylglutaryl-Coenzyme A lyase	0.78	6.91E-09	1.97	0	2.00	0	1.88	4.20E-45
Hmgcr	A_51_P507410	NM_008255	3-hydroxy-3-methylglutaryl-Coenzyme A reductase	0.52	5.89E-44	0.66	3.67E-09	0.92	0.386439	1.20	0.000061
Hmgcr	A_52_P137371	NM_008255	3-hydroxy-3-methylglutaryl-Coenzyme A reductase	0.48	0	0.67	3.48E-06	0.90	0.355384	1.34	2.75E-08
Hmgcr	A_52_P232287	NM_008255	3-hydroxy-3-methylglutaryl-Coenzyme A reductase	0.52	0	0.66	2.16E-11	0.95	0.601767	1.20	0.000469
Hmgcr	A_52_P578922	AK159899	3-hydroxy-3-methylglutaryl-Coenzyme A reductase	0.43	4.09E-37	0.68	5.73E-06	0.94	0.580606	1.43	7.16E-13
Hmgcr	A_52_P770765	AK033165	Mus musculus 15 days embryo male testis cDNA, RIKEN	0.68	4.60E-07	0.67	2.93E-06	1.09	0.431686	1.57	9.55E-13

			full-length enriched library, clone:8030450I20								
Hmgcs1	A_51_P146941	NM_145942	3-hydroxy-3-methylglutaryl-Coenzyme A synthase 1	0.35	6.00E-16	1.27	1.18E-07	1.45	0.000065	1.91	2.44E-37
Hmgcs1	A_52_P119039	AK078743	3-hydroxy-3-methylglutaryl-Coenzyme A synthase 1	0.39	3.28E-13	2.85	1.10E-10	1.17	0.398813	2.66	3.51E-14
Hmgcs1	A_52_P388072	NM_145942	3-hydroxy-3-methylglutaryl-Coenzyme A synthase 1	0.40	2.20E-23	1.30	7.51E-09	1.44	3.22E-08	1.67	0
Hmgcs2	A_51_P116039	NM_008256	3-hydroxy-3-methylglutaryl-Coenzyme A synthase 2	0.76	5.87E-06	0.91	0.109208	0.78	6.29E-06	0.73	5.20E-06
Hsd17b4	A_51_P445662	NM_008292	hydroxysteroid (17-beta) dehydrogenase 4	0.89	4.48E-10	1.43	0	1.44	0	1.35	0
Idh2	A_51_P331507	NM_173011	isocitrate dehydrogenase 2 (NADP+), mitochondrial	0.59	0	1.25	7.74E-13	1.23	2.82E-14	1.13	1.81E-06
Idi1	A_51_P329711	NM_177960	isopentenyl-diphosphate delta isomerase	0.62	0.000572	0.94	0.282964	1.01	0.864314	1.40	2.78E-23
Idi1	A_52_P441634	NM_177960	isopentenyl-diphosphate delta isomerase	0.64	4.74E-12	0.86	0.040286	1.03	0.693076	1.38	8.77E-20
Ldha	A_51_P364140	NM_010699	lactate dehydrogenase A	0.65	1.90E-11	1.06	0.489608	0.91	0.183186	0.87	0.01318
Ldha	A_51_P364146	NM_010699	lactate dehydrogenase A	0.70	2.12E-09	1.12	0.000081	0.98	0.432887	0.95	0.178837
Ldhb	A_51_P409173	NM_008492	lactate dehydrogenase B	1.13	5.39E-06	1.60	2.05E-41	1.62	8.44E-32	1.67	0
Lipe	A_51_P435363	NM_010719	lipase, hormone sensitive	0.82	0.000756	1.20	0.005894	1.34	1.13E-16	1.25	1.77E-07
*Lipe	A_51_P435366	NM_010719	lipase, hormone sensitive	0.84	6.30E-08	1.22	0.059524	1.40	1.39E-12	1.24	9.41E-13
Lpl	A_51_P259296	NM_008509	lipoprotein lipase	1.76	1.81E-24	4.19	0	5.63	0	6.89	0
*Lpl	A_52_P257812	NM_008509	lipoprotein lipase	1.66	1.34E-24	3.24	0	4.41	0	4.56	0
Mdh1	A_52_P407796	NM_008618	malate dehydrogenase 1, NAD (soluble)	0.75	1.21E-11	1.01	0.808442	1.09	0.072866	0.99	0.909665
Mdh2	A_51_P384879	NM_008617	malate dehydrogenase 2, NAD (mitochondrial)	0.94	0.038595	1.43	0	1.34	3.39E-30	1.33	2.66E-41
Mgll	A_51_P139920	NM_011844	monoglyceride lipase	0.80	1.08E-29	1.49	0	1.35	1.26E-44	1.23	5.04E-07
*Mgll	A_52_P568977	AK006949	monoglyceride lipase	0.77	0.000274	0.90	0.232786	0.88	0.255047	0.66	0.000346
Mgll	A_52_P609778	NM_011844	monoglyceride lipase	0.84	3.57E-12	1.59	0	1.41	0	1.29	3.28E-08
Mvd	A_51_P355943	NM_138656	mevalonate (diphospho) decarboxylase	0.40	1.36E-43	1.29	0.000837	1.35	0.000156	1.62	1.17E-18
Mvk	A_51_P169527	NM_023556	mevalonate kinase	0.93	0.253595	0.77	0.000037	0.77	0.000305	0.93	0.225421
Mvk	A_52_P569067	NM_023556	mevalonate kinase	0.82	0.001071	0.73	1.51E-12	0.80	0.000418	0.93	0.00352
Oxct1	A_51_P107321	NM_024188	3-oxoacid CoA transferase 1	1.30	7.23E-10	1.38	1.86E-11	1.29	2.77E-14	1.40	7.08E-11
Oxct1	A_51_P107326	NM_024188	3-oxoacid CoA transferase 1	1.35	0	1.28	2.20E-17	1.24	8.59E-11	1.44	1.76E-33
Oxct1	A_52_P430058	NM_024188	3-oxoacid CoA transferase 1	1.19	0.000017	1.33	1.70E-08	1.37	2.19E-07	1.50	1.73E-06
Pcca	A_51_P484254	NM_144844	propionyl-Coenzyme A carboxylase, alpha polypeptide	0.73	2.61E-26	1.04	0.200605	1.03	0.33632	1.00	0.936793
Pck2	A_52_P21659	NM_028994	phosphoenolpyruvate carboxykinase 2 (mitochondrial)	1.11	0.000025	1.45	0	1.26	5.82E-09	1.35	2.13E-13
Pcx	A_51_P480578	NM_008797	pyruvate carboxylase	0.74	7.36E-10	1.81	4.88E-24	1.62	2.03E-21	1.63	8.96E-17
Pdha1	A_51_P321391	NM_008810	pyruvate dehydrogenase E1 alpha 1	0.95	0.022316	1.35	0	1.37	0	1.29	0
Pdha1	A_52_P581435	NM_008810	pyruvate dehydrogenase E1 alpha 1	0.80	4.68E-15	1.45	0	1.46	0	1.39	0
*Pdk4	A_51_P350453	NM_013743	pyruvate dehydrogenase kinase, isoenzyme 4	0.25	1.85E-37	3.37	0	2.22	0	2.05	0
Pecr	A_51_P291749	NM_023523	peroxisomal trans-2-enoyl-CoA reductase	0.82	9.44E-31	0.57	0	0.62	0	0.64	1.14E-36
Pfkfb2	A_51_P184849	NM_008825	6-phosphofructo-2-kinase/fructose-2,6-biphosphatase 2	0.86	0.031749	0.58	0	0.63	6.43E-24	0.64	1.40E-45

Pfkfb2	A_52_P142912	NM_008825	6-phosphofructo-2-kinase/fructose-2,6-biphosphatase 2	0.85	0.06141	0.57	2.05E-17	0.69	3.58E-06	0.67	4.04E-09
Pfkl	A_51_P310896	NM_008826	phosphofructokinase, liver, B-type	0.76	2.77E-06	1.01	0.902909	1.01	0.829962	0.91	0.152082
Pgam1	A_51_P342669	NM_023418	phosphoglycerate mutase 1	0.87	7.17E-12	1.56	0	1.59	0	1.49	0
Pgam2	A_51_P264495	NM_018870	phosphoglycerate mutase 2	0.71	4.07E-12	1.30	0.000013	1.34	4.56E-09	1.33	6.16E-11
Pgd	A_51_P391996	BC011329	phosphogluconate dehydrogenase	0.69	1.56E-10	1.93	0	1.82	2.35E-28	1.82	4.84E-30
*Pgd	A_52_P84096	BC011329	phosphogluconate dehydrogenase	0.67	3.17E-40	2.17	0	2.16	0	1.92	0
Pgk1	A_51_P182828	NM_008828	phosphoglycerate kinase 1	0.73	0	1.21	0	1.16	8.26E-21	1.09	4.40E-12
Pgk1	A_52_P600398	NM_008828	phosphoglycerate kinase 1	0.73	3.74E-36	1.19	3.80E-26	1.14	5.21E-12	1.10	1.43E-07
Pgm1	A_51_P427530	NM_025700	phosphoglucomutase 1	0.76	5.53E-13	1.02	0.427022	1.12	0.013984	1.16	4.47E-07
Pgm3	A_51_P208987	NM_028352	phosphoglucomutase 3	3.16	0	1.12	0.014164	1.05	0.182808	1.07	0.085847
Pgm3	A_52_P341449	NM_028352	phosphoglucomutase 3	0.96	0.402342	1.37	9.63E-20	1.45	2.67E-13	1.35	2.05E-18
Pgm5	A_52_P229924	NM_175013	phosphoglucomutase 5	0.81	0.00044	1.67	1.86E-12	1.40	0.001732	1.25	0.000377
Phkb	A_51_P307624	NM_199446	phosphorylase kinase beta	1.10	0.000013	1.35	9.18E-34	1.34	1.97E-17	1.30	5.20E-15
Phkb	A_52_P513347	NM_199446	phosphorylase kinase beta	1.07	0.140703	1.45	5.61E-45	1.47	1.77E-25	1.38	2.37E-19
Phkg2	A_51_P235909	NM_026888	phosphorylase kinase, gamma 2 (testis)	1.05	0.207425	0.75	4.23E-18	0.77	2.50E-14	0.79	5.19E-13
Phkg2	A_52_P19446	AK089062	phosphorylase kinase, gamma 2 (testis)	1.07	0.354708	0.61	9.04E-06	0.68	1.24E-09	0.49	0.002143
*Pklr	A_51_P176042	NM_013631	pyruvate kinase liver and red blood cell	0.49	0	0.83	1.13E-09	0.76	6.83E-18	0.67	1.41E-11
Pkm2	A_51_P428913	D38379	pyruvate kinase, muscle	0.68	8.81E-11	1.29	1.07E-14	1.27	2.33E-10	1.31	5.43E-08
Pkm2	A_52_P150212	NM_011099	pyruvate kinase, muscle	0.66	1.55E-12	1.25	9.24E-06	1.33	1.15E-07	1.25	0.000367
Pkm2	A_52_P328249	AK083076	pyruvate kinase, muscle	0.82	0.002	1.28	0.000395	1.05	0.601695	1.29	6.97E-06
Pmvk	A_51_P492408	NM_026784	phosphomevalonate kinase	0.66	1.97E-12	1.33	1.22E-11	1.59	1.72E-26	1.68	0
Pmvk	A_51_P492410	NM_026784	phosphomevalonate kinase	0.66	4.92E-10	1.36	5.92E-10	1.60	1.19E-28	1.71	0
Pparg	A_51_P106799	NM_011146	peroxisome proliferator activated receptor gamma	0.41	0	1.28	8.17E-20	1.36	7.05E-32	1.23	2.11E-29
Ppargc1a	A_51_P279038	NM_008904	peroxisome proliferative activated receptor, gamma, coactivator 1 alpha	0.76	1.34E-06	0.65	7.51E-19	0.62	0	0.63	1.39E-19
Ppargc1a	A_52_P5945	AK032149	peroxisome proliferative activated receptor, gamma, coactivator 1 alpha	0.61	8.10E-20	0.59	5.19E-24	0.57	2.51E-28	0.57	0
Ppargc1b	A_52_P526724	NM_133249	peroxisome proliferative activated receptor, gamma, coactivator 1 beta	1.06	0.377187	0.72	1.20E-21	0.67	8.78E-30	0.80	5.72E-14
Pygb	A_51_P485810	NM_153781	brain glycogen phosphorylase	0.62	0.006731	1.24	0.022984	1.17	0.242466	1.48	0.001698
Pygb	A_52_P350664	NM_153781	brain glycogen phosphorylase	0.86	0.039899	1.35	0.000021	1.20	0.007881	1.39	0.00035
*Scd1	A_52_P682382	NM_009127	stearoyl-Coenzyme A desaturase 1	0.55	1.29E-25	5.55	0	5.84	0	4.87	0
Scd2	A_51_P129464	NM_009128	stearoyl-Coenzyme A desaturase 2	0.56	0	1.43	1.03E-08	2.07	7.01E-45	2.51	0
Scd3	A_51_P446045	NM_024450	stearoyl-coenzyme A desaturase 3	1.35	1.26E-15	1.04	0.508828	1.05	0.624767	1.11	0.031111
Scd4	A_52_P374877	NM_183216	stearoyl-coenzyme A desaturase 4	1.09	0.319806	0.69	0.00002	0.72	0.069299	0.76	0.002199
Slc27a1	A_51_P117477	NM_011977	solute carrier family 27 (fatty acid transporter), member 1	0.93	0.023386	0.67	0	0.70	4.98E-22	0.76	1.63E-17
Slc27a1	A_52_P199905	CB588406	AGENCOURT_12600125 NIH_MGC_136 Mus musculus	0.57	0	0.78	2.87E-11	0.96	0.296208	1.02	0.703983

			cDNA clone IMAGE:30290747 5', mRNA sequence.								
Slc27a4	A_51_P408609	NM_011989	solute carrier family 27 (fatty acid transporter), member 4	0.60	3.38E-07	1.87	8.61E-14	2.16	3.07E-15	1.62	8.40E-17
Slc27a4	A_52_P61854	NM_011989	solute carrier family 27 (fatty acid transporter), member 4	0.63	2.49E-16	2.01	0	2.07	0	1.79	1.30E-18
Slc27a5	A_51_P289742	NM_009512	solute carrier family 27 (fatty acid transporter), member 5	0.87	6.64E-14	0.61	0	0.69	0	0.73	5.80E-11
*Slc2a2	A_51_P185693	NM_031197	solute carrier family 2 (facilitated glucose transporter), member 2	0.73	2.28E-17	0.73	1.40E-45	0.68	0	0.75	3.67E-35
Slc2a2	A_52_P409988	NM_031197	solute carrier family 2 (facilitated glucose transporter), member 2	0.74	5.71E-16	0.78	2.50E-26	0.70	0	0.75	4.88E-22
Slc2a4	A_51_P217498	NM_009204	solute carrier family 2 (facilitated glucose transporter), member 4	0.23	0	0.49	1.75E-33	0.60	0	0.54	1.18E-23
Sqle	A_51_P450487	NM_009270	squalene epoxidase	0.43	4.39E-07	1.00	0.997371	1.12	0.06438	1.45	1.38E-08
Suclg1	A_51_P491227	NM_019879	succinate-CoA ligase, GDP-forming, alpha subunit	0.87	0.000817	1.48	0	1.44	0	1.34	5.36E-11
Suclg2	A_51_P478672	NM_011507	succinate-Coenzyme A ligase, GDP-forming, beta subunit	0.67	1.22E-16	1.43	7.93E-21	1.37	6.10E-11	1.21	2.88E-06
Suclg2	A_52_P58181	NM_011507	succinate-Coenzyme A ligase, GDP-forming, beta subunit	0.64	0	1.23	0	1.21	2.80E-45	1.19	6.71E-19
*Taldo1	A_51_P401958	NM_011528	transaldolase 1	0.86	0.000032	2.20	0	2.06	0	1.80	4.91E-32
*Tkt	A_51_P394515	NM_009388	transketolase	0.73	0	1.73	0	1.72	5.61E-45	1.52	1.62E-32
Tkt	A_52_P624447	NM_009388	transketolase	0.43	6.35E-39	1.88	2.82E-34	2.25	0	1.65	0
Ucp2	A_51_P297105	NM_011671	uncoupling protein 2 (mitochondrial, proton carrier)	0.66	1.96E-19	1.96	1.06E-32	1.80	7.75E-39	1.72	5.56E-22
Ucp2	A_52_P90265	NM_011671	uncoupling protein 2 (mitochondrial, proton carrier)	0.47	0.000083	2.21	2.15E-27	1.80	0.000043	1.76	8.45E-09
Ugp2	A_51_P475580	NM_139297	UDP-glucose pyrophosphorylase 2	0.89	3.23E-12	0.72	3.72E-30	0.74	1.40E-45	0.68	0
Ugp2	A_52_P42242	NM_139297	UDP-glucose pyrophosphorylase 2	0.94	0.083301	0.67	0	0.68	0	0.63	0
Uqcrc2	A_51_P128648	NM_025899	ubiquinol cytochrome c reductase core protein 2	1.04	0.035657	1.3	0	1.25	9.97E-24	1.27	1.31E-36
Acaa2	A_51_P125260	NM_177470	acetyl-Coenzyme A acyltransferase 2 (mitochondrial 3-oxoacyl-Coenzyme A thiolase)	0.88	0.00002	0.98	0.366542	0.92	0.004398	0.92	0.000624
Acad11	A_51_P299260	AK041180	acyl-Coenzyme A dehydrogenase family, member 11	1.04	0.428559	1.10	0.095141	1.05	0.525505	1.11	0.003956
Acad8	A_51_P244052	NM_025862	acyl-Coenzyme A dehydrogenase family, member 8	0.96	0.265814	1.22	2.17E-07	1.21	5.13E-09	1.18	0.000025
Acad9	A_52_P73208	AK049931	acyl-Coenzyme A dehydrogenase family, member 9	0.74	0.008099	0.82	0.001363	0.90	0.393173	0.75	0.010289
Acadsb	A_51_P435068	NM_025826	acyl-Coenzyme A dehydrogenase, short/branched chain	1.17	0.000014	0.78	1.58E-27	0.79	3.19E-10	0.78	3.71E-21
Acadvl	A_51_P518340	NM_017366	acyl-Coenzyme A dehydrogenase, very long chain	0.97	0.599988	1.18	0.000415	1.11	0.055613	1.11	0.047656
Acat2	A_51_P139748	NM_009338	acetyl-Coenzyme A acetyltransferase 2	0.92	0.000014	0.86	2.21E-07	0.78	3.15E-31	0.80	1.59E-22
Aco1	A_51_P391082	NM_007386	aconitase 1	0.88	4.04E-18	1.07	0.000019	1.05	0.068426	1.05	0.002156
Aco1	A_52_P299115	NM_007386	aconitase 1	0.87	8.09E-07	1.16	3.84E-06	1.12	0.000097	1.07	0.042343
Aco2	A_52_P899263	AK029122	Mus musculus 10 days neonate skin cDNA, RIKEN full-length enriched library, clone:4732494K09	1.76	0.047728	0.61	0.172401	0.87	0.302033	0.92	0.638024
Acox3	A_52_P745	NM_030721	acyl-Coenzyme A oxidase 3, pristanoyl	1.26	4.54E-14	1.02	0.456749	0.99	0.618229	0.96	0.116135
Acsl6	A_51_P518823	AK147421	acyl-CoA synthetase long-chain family member 6	1.00	1	1.00	1	1.00	1	1.00	1
Acsl6	A_52_P204459	AK147421	acyl-CoA synthetase long-chain family member 6	1.00	1	0.98	0.814953	1.00	1	1.00	1
Acsl6	A_52_P352187	NM_001033599	acyl-CoA synthetase long-chain family member 6	1.17	0.499166	0.99	0.971515	0.82	0.42818	1.14	0.346036

Adcy1	A_51_P196718	NM_009622	adenylate cyclase 1	1.00	1	1.00	1	1.00	1	1.00	1
Adcy1	A_51_P409900	NM_009622	adenylate cyclase 1	0.73	0.003189	1.06	0.759762	0.88	0.638585	1.16	0.476661
Adcy2	A_51_P337523	NM_153534	adenylate cyclase 2	0.97	0.83704	0.93	0.665948	1.00	1	0.71	0.195467
Adcy2	A_52_P165610	NM_153534	adenylate cyclase 2	1.00	1	1.00	1	0.96	0.773957	1.00	1
Adcy3	A_51_P484671	NM_138305	adenylate cyclase 3	0.89	0.55499	1.19	0.44025	0.85	0.481756	1.20	0.331218
Adcy3	A_52_P216525	NM_138305	adenylate cyclase 3	1.04	0.908022	1.12	0.577074	1.01	0.969592	1.19	0.518511
Adcy5	A_51_P199041	NM_001012765	adenylate cyclase 5	0.99	0.922111	1.15	0.210692	1.23	0.003304	1.14	0.221374
Adcy5	A_52_P449208	NM_001012765	adenylate cyclase 5	0.99	0.910976	1.42	0.170767	1.28	0.228457	0.80	0.354012
Adcy6	A_51_P243134	NM_007405	adenylate cyclase 6	0.95	0.174433	1.27	3.16E-32	1.26	1.47E-27	1.25	6.57E-16
Adcy7	A_51_P441622	NM_007406	adenylate cyclase 7	0.82	0.067813	1.22	0.038712	1.26	0.025238	1.18	0.185388
Adcy7	A_52_P291	AK012436	adenylate cyclase 7	0.74	0.029114	1.09	0.607726	1.74	0.012668	1.22	0.340609
Adcy7	A_52_P586141	NM_001037724	Mus musculus 11 days embryo whole body cDNA, RIKEN library, clone:2700055K03 product:adenylate cyclase 7	0.85	0.000634	1.14	5.97E-06	1.14	0.017144	1.18	0.014898
Adcy8	A_51_P303061	NM_009623	adenylate cyclase 8	0.99	0.935242	1.00	1	1.00	1	0.91	0.640409
Adh1	A_51_P428555	NM_007409	alcohol dehydrogenase 1 (class I)	0.96	0.096899	1.21	9.43E-10	1.18	2.67E-06	1.25	3.63E-18
Adh1	A_52_P835985	AK082149	alcohol dehydrogenase 1 (class I)	0.91	0.69527	1.30	0.111469	1.06	0.808921	1.54	0.004892
Adh5	A_51_P404275	NM_007410	alcohol dehydrogenase 5 (class III), chi polypeptide	0.89	0.000063	0.90	0.000247	0.90	7.30E-07	0.95	0.091475
Adh6a	A_51_P299608	AK028114	alcohol dehydrogenase 6A (class V)	1.00	1	1.00	1	1.00	1	1.00	1
Adh6a	A_52_P276348	AK007397	alcohol dehydrogenase 6A (class V)	1.00	0.997735	1.16	0.535519	1.00	1	1.14	0.499748
Agpat1	A_52_P200465	NM_018862	1-acylglycerol-3-phosphate O-acyltransferase 1 (lysophosphatidic acid acyltransferase, alpha)	0.80	0.000143	0.96	0.076962	0.98	0.33829	1.01	0.796498
Agpat3	A_51_P425490	NM_053014	1-acylglycerol-3-phosphate O-acyltransferase 3	0.90	0.000062	1.11	0.000046	1.01	0.844256	1.06	0.029224
Agpat3	A_52_P523569	NM_053014	1-acylglycerol-3-phosphate O-acyltransferase 3	0.86	2.27E-10	0.99	0.766537	0.92	5.45E-06	0.91	0.000878
Agpat4	A_51_P346165	NM_026644	1-acylglycerol-3-phosphate O-acyltransferase 4 (lysophosphatidic acid acyltransferase, delta)	0.99	0.705586	1.17	4.87E-11	1.26	4.45E-19	1.25	1.13E-11
Agpat4	A_52_P3029	NM_026644	1-acylglycerol-3-phosphate O-acyltransferase 4 (lysophosphatidic acid acyltransferase, delta)	0.99	0.607444	1.17	1.20E-08	1.26	1.90E-12	1.26	1.98E-15
Agpat5	A_52_P572447	NM_026792	1-acylglycerol-3-phosphate O-acyltransferase 5 (lysophosphatidic acid acyltransferase, epsilon)	1.00	0.996347	1.04	0.108555	1.09	8.78E-06	1.10	6.51E-15
Alb	A_51_P160713	NM_009654	albumin	0.82	0.063363	1.09	0.343172	1.01	0.949874	1.21	0.048001
Aldh1a2	A_52_P58145	NM_009022	aldehyde dehydrogenase family 1, subfamily A2	0.90	0.239352	0.82	0.011334	0.95	0.410671	0.98	0.753457
Aldh1a3	A_52_P113518	AK086764	aldehyde dehydrogenase family 1, subfamily A3	1.00	1	1.00	1	1.00	1	1.00	1
Aldh2	A_52_P116134	NM_009656	aldehyde dehydrogenase 2, mitochondrial	1.01	0.773145	0.80	3.20E-07	0.82	3.08E-06	0.82	0.000027
Aldh3a1	A_51_P310594	NM_007436	aldehyde dehydrogenase family 3, subfamily A1	1.00	1	1.00	1	1.00	1	0.99	0.833108
Aldh4a1	A_52_P123230	NM_175438	aldehyde dehydrogenase 4 family, member A1	0.74	0.015549	1.02	0.846919	0.91	0.393784	1.12	0.328169
Aldh5a1	A_51_P491504	NM_172532	aldhehyde dehydrogenase family 5, subfamily A1	1.01	0.689756	1.21	5.56E-22	1.25	1.27E-29	1.26	1.30E-29
Aldh5a1	A_52_P30273	AK051603	aldhehyde dehydrogenase family 5, subfamily A1	0.84	0.253295	1.31	0.204899	1.08	0.692244	1.11	0.554028
Aldh7a1	A_52_P503071	NM_138600	aldehyde dehydrogenase family 7, member A1	0.76	0.00726	0.92	0.387891	0.91	0.364665	0.88	0.26421

Apob	A_51_P380650	XM_137955	Mus musculus apolipoprotein B (Apob), mRNA.	1.21	7.90E-06	1.12	0.004106	0.98	0.503855	1.10	0.050494
Apob	A_51_P413088	XM_137955	Mus musculus apolipoprotein B (Apob), mRNA.	1.22	4.20E-08	1.10	0.032571	1.09	0.024028	1.19	2.69E-06
Apob	A_51_P436690	BC028880	Mus musculus apolipoprotein B (Apob), mRNA.	1.28	3.06E-13	1.13	0.001723	1.09	0.002936	1.17	5.80E-09
Apob	A_51_P470542	XM_137955	apolipoprotein B	1.03	0.384535	0.90	0.000306	0.86	0.000043	0.81	9.83E-10
Apob	A_52_P441070	NM_009693	apolipoprotein B	1.01	0.788954	0.95	0.009214	0.87	3.52E-15	0.82	1.92E-14
Apoc1	A_51_P164504	NM_007469	apolipoprotein C-I	1.04	0.792442	0.98	0.898478	1.00	0.97685	0.95	0.693941
Apoe	A_51_P171999	NM_009696	apolipoprotein E	1.02	0.896869	1.19	0.211645	1.26	0.098964	1.15	0.312869
Atp5a1	A_52_P674489	NM_007505	ATP synthase, H+ transporting, mitochondrial F1 complex, alpha subunit, isoform 1	0.9	0.012	1.02	0.633544	1.14	0.000845	1.03	0.490052
Atp5b	A_51_P207636	NM_016774	ATP synthase, H+ transporting mitochondrial F1 complex, beta subunit	0.98	0.356308	1.23	3.62E-22	1.22	0	1.16	2.30E-26
Atp5b	A_52_P328078	NM_016774	ATP synthase, H+ transporting mitochondrial F1 complex, beta subunit	0.95	0.40001	1.17	0.022786	1.19	0.012458	1.08	0.275166
Atp5b	A_52_P553841	NM_016774	ATP synthase, H+ transporting mitochondrial F1 complex, beta subunit	0.97	0.605303	1.21	0.000013	1.16	0.005251	1.16	0.003521
Atp5c1	A_51_P378087	NM_020615	ATP synthase, H+ transporting, mitochondrial F1 complex, gamma polypeptide 1	1.04	0.059963	1.15	0.000036	1.18	5.42E-13	1.15	1.69E-08
Atp5e	A_51_P362054	NM_025983	ATP synthase, H+ transporting, mitochondrial F1 complex, epsilon subunit	1.04	0.049145	1.01	0.655265	1.03	0.10105	0.99	0.605945
Atp5h	A_51_P264186	NM_027862	ATP synthase, H+ transporting, mitochondrial F0 complex, subunit d	0.99	0.671692	1.04	0.008003	1.07	0.000445	1.05	0.001581
Atp5h	A_52_P640355	NM_027862	ATP synthase, H+ transporting, mitochondrial F0 complex, subunit d	0.97	0.090714	1.03	0.27143	1.04	0.024492	1.03	0.215367
Atp5k	A_51_P301289	NM_007507	ATP synthase, H+ transporting, mitochondrial F1F0 complex, subunit e	1.07	0.048024	1.13	0.000039	1.16	6.70E-07	1.08	0.02929
Atp6ap1	A_51_P447988	NM_018794	ATPase, H+ transporting, lysosomal accessory protein 1	1.00	0.996873	0.95	0.14633	0.92	0.008952	0.93	0.033918
Atp6v0a1	A_51_P142089	NM_016920	ATPase, H+ transporting, lysosomal V0 subunit A1	0.90	4.70E-11	0.88	2.54E-07	0.87	2.13E-08	0.89	1.04E-13
Atp6v0a1	A_52_P399998	NM_016920	ATPase, H+ transporting, lysosomal V0 subunit A1	0.88	0.017629	0.97	0.597649	0.91	0.265117	1.09	0.351065
Atp6v0a1	A_52_P515336	AK045877	ATPase, H+ transporting, lysosomal V0 subunit A1	1.04	0.337179	0.89	0.000272	0.90	0.186745	0.97	0.336444
Atp6v1b2	A_51_P190845	NM_007509	ATPase, H+ transporting, lysosomal V1 subunit B2	0.86	0.024953	1.05	0.43546	1.00	0.979399	1.05	0.547874
Atp6v1c2	A_51_P518600	NM_133699	ATPase, H+ transporting, lysosomal V1 subunit C2	1.00	1	1.00	1	1.00	1	1.10	0.643168
Atp6v1e1	A_51_P166434	NM_007510	VATPase, H+ transporting, lysosomal V1 subunit E1	0.97	0.470883	1.22	9.40E-08	1.11	0.017259	1.18	0.002743
Atp6v1e1	A_52_P303388	NM_007510	VATPase, H+ transporting, lysosomal V1 subunit E1	1.02	0.738669	1.24	7.49E-06	1.13	0.015844	1.17	0.001813
Atp6v1g1	A_51_P140171	NM_024173	ATPase, H+ transporting, lysosomal V1 subunit G1	1.03	0.219316	1.05	0.085816	1.06	0.004339	1.11	0.000964
Atp6v1g1	A_52_P349738	NM_024173	ATPase, H+ transporting, lysosomal V1 subunit G1	0.98	0.195864	1.06	0.003334	1.09	0.000012	1.15	1.43E-13
Atp6v1h	A_52_P112888	NM_133826	ATPase, H+ transporting, lysosomal V1 subunit H	0.83	0.000342	1.19	1.09E-11	1.15	6.08E-09	1.68	4.31E-09
Atp6v1h	A_52_P403420	AK081492	ATPase, H+ transporting, lysosomal V1 subunit H	1.22	0.033537	1.11	0.169795	0.81	0.062047	1.22	0.075106
Atp7a	A_51_P312497	NM_009726	ATPase, Cu++ transporting, alpha polypeptide	1.13	6.03E-08	1.20	9.59E-31	1.29	2.30E-23	1.22	6.06E-15
Atp7a	A_51_P473179	AK033254	ATPase, Cu++ transporting, alpha polypeptide	0.98	0.861463	0.90	0.401433	0.85	0.303032	0.98	0.81128
Auh	A_51_P372473	NM_016709	AU RNA binding protein/enoyl-coenzyme A hydratase	1.05	0.027074	1.04	0.216899	1.05	0.034515	1.05	0.018087

	Auh	A_52_P4095	AK169661	AU RNA binding protein/enoyl-coenzyme A hydratase	1.19	0.039658	1.04	0.654592	1.29	0.029953	1.11	0.051057
	Bdh1	A_51_P163106	NM_175177	3-hydroxybutyrate dehydrogenase, type 1	0.96	0.231285	1.09	0.003551	0.95	0.162229	0.94	0.032961
	Bdh1	A_52_P468343	AK146962	3-hydroxybutyrate dehydrogenase, type 1	1.10	1.14E-06	0.86	4.27E-07	0.80	5.58E-24	0.82	1.61E-16
	Btd	A_51_P196158	NM_025295	biotinidase	0.81	1.79E-21	0.93	0.000532	0.94	0.001048	0.94	0.000016
	Cat	A_52_P679769	NM_009804	catalase	1.14	0.000204	0.97	0.216827	0.92	0.00555	0.93	0.042432
	Coq2	A_51_P447752	NM_027978	coenzyme Q2 homolog, prenyltransferase (yeast)	0.92	0.008449	1.29	9.84E-24	1.25	1.29E-13	1.20	1.79E-07
	Cox1	A_51_P315595	X57780	M.musculus mRNA for mitochondrial gene for subunit I of cytochrome c oxidase.	0.98	0.603027	1.06	0.0029	1.049	0.051597	1.07	0.012453
	Cox4i1	A_51_P165435	NM_009941	cytochrome c oxidase subunit IV isoform 1	1.19	0.000175	1.20	0.003221	1.24	0.000139	1.17	0.004552
	Cox6a1	A_51_P311540	NM_007748	cytochrome c oxidase, subunit VI a, polypeptide 1	1.04	0.429623	1.21	0.000472	1.22	0.000632	1.12	0.020661
	Cox6b1	A_51_P448032	NM_025628	cytochrome c oxidase, subunit VIb polypeptide 1	0.95	0.048421	1.19	4.31E-24	1.18	1.16E-13	1.12	0.000056
	Cox7b	A_51_P160664	NM_025379	cytochrome c oxidase subunit VIIb	1.03	0.497297	1.15	5.77E-07	1.16	6.16E-06	1.16	0.000016
	Cpt1c	A_52_P269942	NM_153679	carnitine palmitoyltransferase 1c	0.66	0.057013	1.52	0.124214	0.83	0.334891	0.97	0.905843
	Cpt2	A_51_P403388	NM_009949	carnitine palmitoyltransferase 2	0.84	0.000014	0.99	0.88152	0.85	0.000213	0.76	6.33E-18
	Crot	A_51_P489153	NM_023733	carnitine O-octanoyltransferase	0.92	0.280595	1.08	2.66E-06	0.98	0.466252	1.00	0.949289
	Crot	A_52_P656336	NM_023733	carnitine O-octanoyltransferase	0.86	0.087561	1.13	0.001593	0.97	0.522182	0.94	0.104907
	Cyp19a1	A_51_P474551	NM_007810	cytochrome P450, family 19, subfamily a, polypeptide 1	1.45	0.164478	0.93	0.64475	1.00	0.970276	1.15	0.561799
	Cyp19a1	A_52_P591961	NM_007810	cytochrome P450, family 19, subfamily a, polypeptide 1	1.00	1	1.00	1	1.00	1	1.00	1
	Cyp1b1	A_52_P297822	AK016699	cytochrome P450, family 1, subfamily b, polypeptide 1	0.95	0.733546	1.00	1	0.99	0.895876	1.16	0.432126
	Cyp2b10	A_51_P182362	NM_009998	Mus musculus cytochrome P450, family 2, subfamily b, polypeptide 10 (Cyp2b10), transcript variant 2, mRNA.	0.09	0	1.03	0.227611	1.00	0.940862	0.96	0.186907
	Cyp2b19	A_51_P352763	NM_007814	cytochrome P450, family 2, subfamily b, polypeptide 19	1.04	0.415928	1.08	0.180674	0.91	0.104567	1.01	0.905298
	Cyp2b19	A_52_P421626	AK029227	cytochrome P450, family 2, subfamily b, polypeptide 19	1.00	1	1.00	1	1.08	0.585023	1.00	1
	Cyp2c65	A_52_P652059	AK008688	cytochrome P450, family 2, subfamily c, polypeptide 65	1.00	0.981529	1.18	0.233766	1.17	0.392156	1.06	0.449655
	Cyp2c66	A_51_P471126	NM_001011707	cytochrome P450, family 2, subfamily c, polypeptide 66	0.97	0.72599	0.82	0.172389	1.11	0.536281	0.94	0.461801
	Cyp2d22	A_51_P124126	NM_019823	cytochrome P450, family 2, subfamily d, polypeptide 22	1.22	1.23E-10	0.78	4.11E-28	0.78	1.66E-21	0.82	1.82E-14
	Cyp2e1	A_51_P283456	NM_021282	cytochrome P450, family 2, subfamily e, polypeptide 1	1.11	0.04857	0.84	0.000209	0.88	0.002312	0.99	0.758209
	Cyp2g1	A_52_P481346	NM_013809	cytochrome P450, family 2, subfamily g, polypeptide 1	1.00	1	1.05	0.744023	1.17	0.42863	1.03	0.799866
	Cyp2j5	A_51_P307872	NM_010007	cytochrome P450, family 2, subfamily j, polypeptide 5	1.01	0.830123	1.02	0.640288	1.05	0.048849	1.06	0.153443
	Cyp2j5	A_52_P315766	NM_010007	cytochrome P450, family 2, subfamily j, polypeptide 5	1.04	0.083529	1.03	0.068206	1.05	0.002326	1.04	0.18742
	Cyp2s1	A_51_P180091	NM_028775	cytochrome P450, family 2, subfamily s, polypeptide 1	1.05	0.538386	0.97	0.548061	1.01	0.823561	1.02	0.61247
	Cyp2s1	A_52_P456837	AK054324	cytochrome P450, family 2, subfamily s, polypeptide 1	1.00	1	1.00	1	1.01	0.930048	1.00	1
	Cyp3a11	A_51_P355301	NM_007818	cytochrome P450, family 3, subfamily a, polypeptide 11	0.86	0.000177	0.96	0.324168	0.90	0.00769	1.08	0.001683
	Cyp3a16	A_51_P482051	NM_007820	cytochrome P450, family 3, subfamily a, polypeptide 16	0.87	0.002525	1.00	0.965319	0.98	0.707491	1.11	5.97E-06
	Cyp3a25	A_52_P380314	NM_019792	cytochrome P450, family 3, subfamily a, polypeptide 25	0.71	5.38E-11	0.41	0	0.41	0	0.45	0
	Cyp3a44	A_52_P571006	NM_177380	cytochrome P450, family 3, subfamily a, polypeptide 44	0.90	0.012206	1.04	0.291366	0.99	0.763606	1.13	8.65E-10

	Cyp51	A_52_P636752	NM_020010	cytochrome P450, family 51	0.79	0.051095	0.94	0.360558	1.01	0.923296	1.30	2.28E-09
	Cyp51	A_52_P778750	AK035338	Mus musculus adult male urinary bladder cDNA, RIKEN full-length enriched library, clone:9530019F09	1.00	1	1.00	1	1.00	1	1.00	1
	Dgat2	A_51_P396003	NM_026384	diacylglycerol O-acyltransferase 2	0.89	0.008861	1.14	0.00044	1.27	1.74E-08	1.06	0.157726
	Dgat2	A_52_P359739	NM_026384	diacylglycerol O-acyltransferase 2	0.88	0.051346	1.14	0.016177	1.28	0.000038	1.10	0.10071
	Dld	A_51_P184282	U73445	dihydrolipoamide dehydrogenase	0.90	0.001636	1.27	3.40E-14	1.22	2.96E-11	1.23	2.81E-10
	Dld	A_52_P536646	NM_007861	dihydrolipoamide dehydrogenase	0.80	5.69E-09	1.15	7.99E-08	1.11	1.46E-06	1.14	0.000644
	Dlst	A_52_P3383	AK171100	dihydrolipoamide S-succinyltransferase (E2 component of 2-oxo-glutarate complex)	0.85	0.006272	0.93	0.006719	0.98	0.678665	0.94	0.036806
	Eno2	A_51_P130028	NM_013509	enolase 2, gamma neuronal	1.34	0.271552	1.15	0.343749	1.00	1	0.98	0.912722
	Eno2	A_52_P748882	NM_013509	enolase 2, gamma neuronal	0.99	0.903121	0.98	0.761328	1.00	1	0.96	0.744186
	Fabp1	A_51_P487818	NM_017399	fatty acid binding protein 1, liver	1.26	0.060944	1.53	0.001301	1.31	0.068549	1.20	0.139975
	Fdft1	A_52_P136138	NM_010191	farnesyl diphosphate farnesyl transferase 1	0.60	0.017103	0.83	0.00426	0.80	0.189778	1.24	0.122908
	Fnta	A_51_P112116	NM_008033	farnesyltransferase, CAAX box, alpha	0.97	0.24712	1.03	0.173159	1.02	0.437387	0.97	0.040183
	Fntb	A_52_P235241	NM_145927	farnesyltransferase, CAAX box, beta	0.80	0.040606	0.97	0.861701	0.93	0.608578	1.08	0.481478
	Gck	A_51_P106294	NM_010292	glucokinase	0.97	0.476887	1.09	0.011222	0.93	0.125807	0.99	0.865216
	Gck	A_52_P259537	NM_010292	glucokinase	1.06	0.254595	1.28	3.19E-08	1.17	0.013355	1.19	2.51E-07
	Ggps1	A_51_P179604	NM_010282	PREDICTED: Mus musculus geranylgeranyl diphosphate synthase 1, transcript variant 4 (Ggps1), mRNA.	1.04	0.472344	1.12	0.003896	1.15	0.007875	1.11	0.000026
	Ggps1	A_51_P469902	XM_977926	geranylgeranyl diphosphate synthase 1	1.28	5.77E-12	0.89	0.00143	0.94	0.079858	0.95	0.170639
	Ggps1	A_52_P228247	NM_010282	geranylgeranyl diphosphate synthase 1	0.90	0.008867	1.14	0.000093	1.04	0.160856	1.04	0.215054
	Gpi1	A_52_P347412	L09104	glucose phosphate isomerase 1	1.29	0.000019	1.01	0.809703	0.97	0.570334	1.01	0.782621
	Gyk	A_51_P297671	NM_008194	glycerol kinase	0.98	0.570454	1.01	0.791418	0.88	0.000016	0.89	0.000473
	Gyk	A_52_P120066	NM_212444	glycerol kinase	1.04	0.040844	0.99	0.766235	0.85	0.000011	0.91	0.000024
	Gyk	A_52_P453517	NM_008194	glycerol kinase	0.94	0.069562	0.91	5.49E-06	0.79	1.01E-06	0.81	1.15E-08
	Gys1	A_52_P228932	NM_030678	glycogen synthase 1, muscle	0.86	0.047964	0.93	0.605257	0.84	0.204287	0.97	0.901676
	Hadha	A_52_P193029	NM_178878	hydroxyacyl-Coenzyme A dehydrogenase/3-ketoacyl-Coenzyme A thiolase/enoyl-Coenzyme A hydratase, alpha subunit	0.89	0.005546	1.14	0.006069	1.07	0.099692	1.03	0.55424
	Hadha	A_52_P338459	AK035316	hydroxyacyl-Coenzyme A dehydrogenase/3-ketoacyl-Coenzyme A thiolase/enoyl-Coenzyme A hydratase, alpha subunit	1.20	0.360011	1.07	0.719102	1.00	1	1.02	0.909089
	Hadha	A_52_P658044	AK050856	hydroxyacyl-Coenzyme A dehydrogenase/3-ketoacyl-Coenzyme A thiolase/enoyl-Coenzyme A hydratase, alpha subunit	1.15	0.096182	0.85	0.006564	0.85	0.007326	0.87	0.004653
	Hk1	A_51_P249024	NM_010438	hexokinase 1	1.18	7.57E-07	1.05	0.335763	1.09	0.03018	1.03	0.384791
	Hk1	A_52_P479599	NM_010438	hexokinase 1	0.87	0.522476	1.03	0.869145	1.06	0.757768	1.07	0.652623
	Hk1	A_52_P674530	NM_010438	hexokinase 1	0.85	0.0145	1.12	0.012129	1.05	0.273689	1.01	0.794436
	Hk1	A_52_P87141	NM_010438	hexokinase 1	0.98	0.81715	1.00	1	1.00	1	1.10	0.559198

	Hmgcll1	A_52_P197722	NM_173731	3-hydroxymethyl-3-methylglutaryl-Coenzyme A lyase-like 1	1.09	0.777923	0.77	0.218857	0.95	0.76741	1.22	0.208341
	Idh1	A_51_P132978	NM_010497	isocitrate dehydrogenase 1 (NADP+), soluble	0.78	1.59E-06	1.21	0.000172	1.14	0.038305	1.22	0.001804
	Idh3a	A_51_P268559	NM_029573	isocitrate dehydrogenase 3 (NAD+) alpha	1.11	0.028306	1.10	0.024044	1.10	0.057854	1.10	0.081078
	Idh3b	A_51_P130110	NM_130884	isocitrate dehydrogenase 3 (NAD+) beta	1.03	0.033662	1.19	2.29E-25	1.19	3.43E-20	1.13	1.31E-14
	Idh3g	A_51_P136729	NM_008323	isocitrate dehydrogenase 3 (NAD+), gamma	1.03	0.119279	1.17	9.42E-10	1.12	1.59E-06	1.07	0.025146
	Ldhc	A_51_P242403	NM_013580	lactate dehydrogenase C	1.00	1	1.07	0.562345	1.00	1	1.00	1
	Mcat	A_51_P314855	NM_001030014	malonyl CoA:ACP acyltransferase (mitochondrial)	1.04	0.277141	1.09	0.001639	1.18	3.41E-08	1.17	7.36E-10
	Mdh1	A_51_P218179	NM_008618	malate dehydrogenase 1, NAD (soluble)	0.84	6.43E-16	1.13	4.33E-14	1.15	2.95E-17	1.11	2.40E-06
	Mdh1	A_52_P470451	AK077724	malate dehydrogenase 1, NAD (soluble)	0.82	0.001691	0.89	0.003858	0.87	0.026208	0.88	0.028825
	Mecr	A_51_P386080	NM_025297	mitochondrial trans-2-enoyl-CoA reductase	1.08	0.000028	1.18	3.33E-24	1.20	2.09E-24	1.11	0.000106
	Mgll	A_51_P352549	AK079089	Mus musculus adult male diencephalon cDNA, RIKEN full-length enriched library, clone:9330168D17	1.00	1	1.00	1	1.00	1	1.00	1
	Mut	A_51_P193935	NM_008650	methylmalonyl-Coenzyme A mutase	0.97	0.068371	1.02	0.257427	1.05	0.014557	1.05	0.005997
	Mut	A_52_P362959	AK051851	methylmalonyl-Coenzyme A mutase	1.00	1	1.00	1	1.00	1	1.00	1
	Mut	A_52_P633163	NM_008650	methylmalonyl-Coenzyme A mutase	0.96	0.034159	0.99	0.638194	1.05	0.035929	1.05	0.049613
	Ndufa1	A_51_P472405	NM_019443	NADH dehydrogenase (ubiquinone) 1 alpha subcomplex, 1	1.24	4.76E-07	1.12	0.000217	1.14	0.002727	1.15	1.53E-06
	Ndufa1	A_52_P139747	NM_019443	NADH dehydrogenase (ubiquinone) 1 alpha subcomplex, 1	1.21	1.49E-28	1.13	2.70E-13	1.12	5.40E-10	1.10	0.000048
	Ndufa5	A_51_P170156	NM_026614	NADH dehydrogenase (ubiquinone) 1 alpha subcomplex, 5	0.99	0.882298	1.18	2.06E-30	1.18	1.26E-13	1.16	6.42E-22
	Ogdh	A_51_P122825	NM_010956	oxoglutarate dehydrogenase (lipoamide)	1.15	3.64E-09	1.15	8.42E-15	1.11	0.000541	1.03	0.161107
	Ogdh	A_52_P369310	NM_010956	oxoglutarate dehydrogenase (lipoamide)	1.18	0.003134	1.18	1.36E-07	1.16	0.000016	1.11	0.000404
	Oxct2a	A_51_P125056	NM_022033	3-oxoacid CoA transferase 2A	1.00	1	1.16	0.515849	1.00	1	1.04	0.854468
	Oxct2a	A_51_P125062	NM_022033	3-oxoacid CoA transferase 2A	1.13	0.164532	1.02	0.868195	0.90	0.446154	0.84	0.018639
	Oxsm	A_51_P312748	NM_027695	3-oxoacyl-ACP synthase, mitochondrial	0.79	3.76E-16	1.08	0.017127	1.05	0.096452	1.01	0.629888
	Oxsm	A_52_P231635	NM_027695	3-oxoacyl-ACP synthase, mitochondrial	0.79	9.32E-21	0.93	0.001567	0.94	0.000237	0.87	4.34E-11
	Pccb	A_51_P418259	NM_025835	propionyl Coenzyme A carboxylase, beta polypeptide	0.83	0.00004	0.82	1.48E-16	0.81	2.82E-10	0.84	3.26E-10
	Pdha2	A_51_P519857	NM_008811	pyruvate dehydrogenase E1 alpha 2	1.00	1	1.16	0.509431	1.00	1	1.00	1
	Pdhb	A_51_P321921	NM_024221	pyruvate dehydrogenase (lipoamide) beta	1.06	0.003688	1.27	3.32E-18	1.21	3.33E-10	1.19	2.46E-09
	Pdhb	A_52_P568792	NM_024221	pyruvate dehydrogenase (lipoamide) beta	1.07	0.00361	1.30	6.01E-25	1.29	8.97E-25	1.24	4.77E-19
	Peci	A_51_P394665	NM_011868	peroxisomal delta3, delta2-enoyl-Coenzyme A isomerase	0.82	3.43E-09	1.18	9.97E-22	1.17	3.97E-14	1.13	9.04E-07
	Pfkfb2	A_51_P335251	NM_008825	6-phosphofructo-2-kinase/fructose-2,6-biphosphatase 2	0.96	0.216859	0.84	9.89E-12	0.86	0.000024	0.99	0.77072
	Pfkfb2	A_52_P31125	BC018418	6-phosphofructo-2-kinase/fructose-2,6-biphosphatase 2	0.94	0.610521	0.86	0.356	1.08	0.594667	0.70	0.016372
	Pgk2	A_51_P125487	NM_031190	phosphoglycerate kinase 2	1.05	0.784724	1.03	0.697266	1.00	1	1.00	1
	Pgls	A_51_P321794	NM_025396	6-phosphogluconolactonase	1.03	0.291577	1.06	0.000076	1.11	2.74E-09	1.04	0.002036
	Pgm2	A_51_P368074	NM_028132	phosphoglucomutase 2	0.82	3.56E-10	0.85	5.77E-20	0.87	4.76E-11	0.90	0.000429
	Pgm5	A_51_P451052	NM_175013	phosphoglucomutase 5	0.98	0.671143	1.08	0.075133	1.05	0.084715	1.22	1.44E-07

Pgm5	A_52_P860000	NM_175013	phosphoglucomutase 5	1.00	1	0.98	0.818372	1.00	1	1.00	1
Phka1	A_52_P196825	AK078633	Mus musculus adult male adrenal gland cDNA, RIKEN full-length enriched library, clone:7330411B16	0.88	0.480679	0.62	0.014898	0.78	0.195584	1.08	0.648633
Phka1	A_52_P278311	NM_008832	phosphorylase kinase alpha 1	0.85	0.158018	0.78	0.11666	0.98	0.907624	0.86	0.090491
Phka2	A_51_P239970	AK051899	phosphorylase kinase alpha 2	1.00	1	1.15	0.545352	1.10	0.50509	1.04	0.686166
Phka2	A_51_P368950	AK018208	Mus musculus 12 days embryo eyeball cDNA, RIKEN full-length enriched library, clone:D230017A12	0.88	0.307474	0.93	0.702429	0.90	0.55921	0.94	0.674615
Phka2	A_52_P187217	NM_172783	phosphorylase kinase alpha 2	0.88	0.034853	0.90	0.423898	1.18	0.215217	1.03	0.869927
Phka2	A_52_P657963	AK045618	phosphorylase kinase alpha 2	1.16	0.001022	0.93	0.022303	0.92	0.119792	1.09	0.084881
Phka2	A_52_P671916	NM_172783	phosphorylase kinase alpha 2	0.93	0.004873	0.99	0.811022	0.96	0.088716	0.97	0.151425
Phkb	A_51_P270725	AK089493	phosphorylase kinase beta	1.14	0.000124	1.01	0.887567	0.94	0.05564	0.90	0.00001
Phkb	A_52_P342907	BC051503	phosphorylase kinase beta	1.00	1	1.01	0.953081	1.04	0.676405	1.00	1
Phkg1	A_51_P280890	NM_011079	phosphorylase kinase gamma 1	1.10	0.730057	1.03	0.892804	0.98	0.897851	0.78	0.296068
Ppap2a	A_51_P189104	NM_008903	phosphatidic acid phosphatase 2a	0.83	0.079681	1.04	0.286229	1.05	0.156363	1.15	3.81E-14
Ppap2a	A_51_P189105	AK077275	phosphatidic acid phosphatase 2a	0.83	0.056563	1.02	0.467365	1.03	0.348762	1.18	1.08E-09
Ppap2a	A_52_P7937	NM_008903	phosphatidic acid phosphatase 2a	0.72	0.016261	0.96	0.171748	0.98	0.615491	1.10	1.85E-06
Ppara	A_51_P348334	NM_011144	peroxisome proliferator activated receptor alpha	0.98	0.788081	1.17	8.26E-07	1.11	0.000408	1.27	1.26E-13
Ppard	A_51_P271556	NM_011145	peroxisome proliferator activator receptor delta	1.04	0.642773	0.73	0.150016	0.63	0.208207	1.02	0.878302
Ppargc1b	A_51_P294891	NM_133249	peroxisome proliferative activated receptor, gamma, coactivator 1 beta	1.29	0.000013	0.81	1.75E-07	0.78	5.26E-15	0.89	0.000557
Ppargc1b	A_52_P293222	AK042378	peroxisome proliferative activated receptor, gamma, coactivator 1 beta	1.25	0.003137	0.85	7.08E-07	0.78	0.000461	0.97	0.377482
Ppargc1b	A_52_P883463	AK045690	Mus musculus adult male corpora quadrigemina cDNA, RIKEN full-length enriched library, clone:B230304K20	1.15	0.158027	0.92	0.238098	0.82	0.031244	0.78	6.90E-06
Ppp1ca	A_51_P509389	NM_031868	protein phosphatase 1, catalytic subunit, alpha isoform	0.80	3.22E-14	1.06	0.033849	1.04	0.111977	1.05	0.085473
Ppp1cb	A_51_P342716	NM_172707	protein phosphatase 1, catalytic subunit, beta isoform	0.90	0.011973	0.97	0.410774	0.97	0.386287	0.94	0.177457
Ppp1cc	A_52_P519508	NM_013636	protein phosphatase 1, catalytic subunit, gamma isoform	0.81	5.63E-32	0.77	0	0.78	0	0.78	0
Ppp1cc	A_52_P540434	NM_013636	protein phosphatase 1, catalytic subunit, gamma isoform	0.80	6.94E-24	0.88	6.23E-13	0.88	1.82E-12	0.88	1.01E-15
Ppt2	A_52_P238816	NM_019441	palmitoyl-protein thioesterase 2	1.03	0.383235	0.95	0.096642	0.99	0.782912	0.96	0.270241
Pygl	A_51_P452779	NM_133198	liver glycogen phosphorylase	0.92	0.087105	1.18	0.000045	1.06	0.218167	1.10	0.044664
Pygl	A_52_P320181	AK083075	liver glycogen phosphorylase	0.76	0.004919	1.08	0.320657	1.03	0.694414	1.10	0.23566
Pygm	A_51_P458973	NM_011224	muscle glycogen phosphorylase	1.00	1	0.97	0.837067	1.00	1	0.98	0.863715
Scd4	A_52_P603237	BC038322	stearoyl-coenzyme A desaturase 4	1.00	1	1.07	0.666933	1.00	1	0.85	0.069816
Sdha	A_51_P410823	NM_023281	succinate dehydrogenase complex, subunit A, flavoprotein (Fp)	0.88	0.00022	1.10	1.53E-07	1.13	4.52E-10	1.08	0.001453
Sdha	A_52_P109503	NM_023281	succinate dehydrogenase complex, subunit A, flavoprotein (Fp)	0.93	0.04729	1.19	7.95E-07	1.16	0.000027	1.11	0.000756
Sdhb	A_51_P234853	NM_023374	succinate dehydrogenase complex, subunit B, iron sulfur (Ip)	0.84	7.31E-13	0.99	0.5721	1.03	0.066127	0.97	0.08288
Sdhc	A_51_P106373	NM_025321	succinate dehydrogenase complex, subunit C, integral	0.83	2.25E-15	0.96	0.090978	0.97	0.21256	0.95	0.058894

				membrane protein								
	Sdhc	A_52_P875481	AK031343	Mus musculus 13 days embryo male testis cDNA, RIKEN full-length enriched library, clone:6030410A06	1.08	0.644243	0.98	0.902773	1.00	0.970829	1.00	1
	Sdhd	A_51_P260871	NM_025848	succinate dehydrogenase complex, subunit D, integral membrane protein	1.01	0.756305	1.14	0.001428	1.10	0.043605	1.11	0.014283
	Slc25a1	A_51_P350922	NM_153150	solute carrier family 25 (mitochondrial carrier, citrate transporter), member 1	0.88	2.65E-06	1.23	2.18E-15	1.30	2.60E-23	1.27	3.12E-15
	Slc27a2	A_51_P484551	NM_011978	solute carrier family 27 (fatty acid transporter), member 2	0.94	0.00213	0.93	0.002681	0.97	0.231198	0.95	0.070287
	Slc27a2	A_52_P1083677	AK038847	solute carrier family 27 (fatty acid transporter), member 2	0.73	0.103408	1.14	0.607967	0.79	0.399642	0.99	0.952548
	Slc27a2	A_52_P154971	NM_011978	solute carrier family 27 (fatty acid transporter), member 2	0.99	0.631588	0.94	0.001587	1.02	0.302126	0.98	0.183616
	Slc27a3	A_51_P133252	NM_011988	solute carrier family 27 (fatty acid transporter), member 3	0.87	0.255597	0.95	0.300019	0.93	0.448725	1.00	0.992485
	Slc27a6	A_51_P211616	AK028699	solute carrier family 27 (fatty acid transporter), member 6	0.99	0.890495	1.00	1	1.00	1	1.07	0.578975
	Sucla2	A_51_P111554	NM_011506	succinate-Coenzyme A ligase, ADP-forming, beta subunit	0.96	0.08897	0.97	0.289304	0.98	0.535233	0.90	0.000028
	Sucla2	A_52_P533792	NM_011506	succinate-Coenzyme A ligase, ADP-forming, beta subunit	0.96	0.360955	1.12	0.000544	1.14	0.000179	1.12	0.000097
	Sucla2	A_52_P762514	AK016060	succinate-Coenzyme A ligase, ADP-forming, beta subunit	0.88	0.421099	0.87	0.590173	0.96	0.87672	0.89	0.412321
	Tkt	A_52_P631197	AK050793	transketolase	0.78	0.241876	1.48	0.186559	0.85	0.590191	1.53	0.174647
	Tpi1	A_51_P433824	NM_009415	triosephosphate isomerase 1	0.88	0.000041	1.00	0.969896	0.98	0.678977	0.90	0.008627
	Tpi1	A_52_P116102	AK053780	Mus musculus 0 day neonate eyeball cDNA, RIKEN library, clone:E130308D14 product:triosephosphate isomerase	0.97	0.812806	0.97	0.568244	0.93	0.459585	1.05	0.53092
	Ucp1	A_51_P426353	NM_009463	uncoupling protein 1 (mitochondrial, proton carrier)	1.00	1	1.00	1	1.00	1	1.00	1
	Ucp2	A_52_P499675	NM_011671	uncoupling protein 2 (mitochondrial, proton carrier)	1.00	1	1.01	0.960441	1.02	0.865651	1.08	0.64936
	Uqcrb	A_51_P472671	NM_026219	ubiquinol-cytochrome c reductase binding protein	1.04	0.434255	1.05	0.055891	1.12	0.010651	1.15	1.56E-06
	Uqcrc1	A_51_P109835	NM_025407	ubiquinol-cytochrome c reductase core protein 1	0.96	0.014244	1.09	6.31E-07	1.12	0.000044	1.16	9.23E-13
	Uqcrfs1	A_51_P361951	NM_025710	ubiquinol-cytochrome c reductase, Rieske iron-sulfur polypeptide 1	0.86	7.50E-37	1.13	2.30E-12	1.12	7.75E-17	1.08	0.001294
	Uqcrh	A_51_P431852	NM_025641	ubiquinol-cytochrome c reductase hinge protein	1.21	1.04E-08	1.08	0.002795	1.06	0.00994	1.09	0.005119
	Uqcrh	A_52_P541875	NM_025641	ubiquinol-cytochrome c reductase hinge protein	1.22	6.72E-07	1.07	0.043535	1.14	2.54E-06	1.06	0.035047
	Uqcrq	A_52_P370484	NM_025352	ubiquinol-cytochrome c reductase, complex III subunit VII	1.04	0.036	1.301	0	1.25	9.97E-24	1.27	1.31E-36
**IAT**	Acaa1a	A_51_P327075	NM_130864	acetyl-Coenzyme A acyltransferase 1A	1.10	0.000167	1.26	3.60E-15	1.41	4.04E-28	1.59	1.68E-27
	Acaa1a	A_52_P155990	NM_130864	acetyl-Coenzyme A acyltransferase 1A	1.16	0.002963	1.39	2.29E-09	1.92	2.15E-33	2.32	1.40E-45
	*Acaa1a	A_52_P423183	NM_130864	acetyl-Coenzyme A acyltransferase 1A	1.26	1.05E-14	1.50	1.15E-27	2.07	0	2.54	0
	*Acaa1b	A_52_P423174	NM_146230	acetyl-Coenzyme A acyltransferase 1B	1.35	5.22E-28	1.50	0	2.07	0	2.52	0
	*Acaa2	A_51_P125260	NM_177470	acetyl-Coenzyme A acyltransferase 2 (mitochondrial 3-oxoacyl-Coenzyme A thiolase)	1.21	0.024048	1.16	6.32E-12	1.81	2.80E-45	2.35	0
	Acaca	A_51_P439426	NM_133360	acetyl-Coenzyme A carboxylase alpha	7.02	0	2.04	5.09E-27	2.81	0	3.00	0
	Acaca	A_52_P132591	AF374167	acetyl-Coenzyme A carboxylase alpha	4.60	4.32E-40	1.80	5.95E-07	3.31	0	3.36	5.68E-23
	Acaca	A_52_P23177	NM_133360	acetyl-Coenzyme A carboxylase alpha	2.68	0	1.53	1.58E-21	1.78	0	2.07	0

Acaca	A_52_P318040	AK132628	acetyl-Coenzyme A carboxylase alpha	5.28	0	1.82	1.73E-25	2.50	0	2.80	0
*Acaca	A_52_P595124	NM_133360	acetyl-Coenzyme A carboxylase alpha	5.55	0	1.61	1.91E-17	2.64	0	2.70	4.36E-37
Acacb	A_51_P239236	BC022940	acetyl-Coenzyme A carboxylase beta	1.29	0.000146	1.03	0.398657	1.33	2.02E-08	1.44	4.24E-26
*Acacb	A_52_P340167	NM_133904	acetyl-Coenzyme A carboxylase beta	1.54	5.67E-11	1.20	0.001612	1.67	2.29E-12	1.56	2.35E-23
Acad10	A_51_P187507	NM_028037	acyl-Coenzyme A dehydrogenase family, member 10	1.38	0	1.11	0.00006	1.15	1.93E-20	1.23	5.76E-31
Acad11	A_51_P299260	AK041180	acyl-Coenzyme A dehydrogenase family, member 11	1.70	7.46E-18	1.17	0.056592	1.22	0.001247	1.31	5.06E-08
Acad11	A_52_P85152	NM_175324	acyl-Coenzyme A dehydrogenase family, member 11	1.06	0.014579	1.00	0.963039	1.34	7.19E-08	1.53	1.43E-22
Acad8	A_51_P244052	NM_025862	acyl-Coenzyme A dehydrogenase family, member 8	1.36	1.55E-21	1.05	0.264035	1.28	1.62E-11	1.33	2.93E-16
Acad9	A_51_P341379	NM_172678	acyl-Coenzyme A dehydrogenase family, member 9	1.11	1.95E-07	1.39	1.15E-43	1.50	0	1.77	0
Acad9	A_52_P73208	AK049931	acyl-Coenzyme A dehydrogenase family, member 9	1.84	2.64E-20	1.64	3.64E-26	1.70	4.45E-10	2.22	0
*Acadl	A_51_P149455	NM_007381	acyl-Coenzyme A dehydrogenase, long-chain	1.59	1.65E-28	1.21	9.13E-08	1.72	0	2.00	0
*Acadm	A_51_P319879	NM_007382	acyl-Coenzyme A dehydrogenase, medium chain	0.98	0.054679	1.23	2.24E-08	1.92	0	2.26	0
*Acads	A_52_P367745	NM_007383	acyl-Coenzyme A dehydrogenase, short chain	1.35	0.000141	1.34	7.62E-10	1.88	1.09E-35	2.08	1.15E-43
Acadsb	A_51_P435068	NM_025826	acyl-Coenzyme A dehydrogenase, short/branched chain	1.49	2.98E-32	0.97	0.33264	1.01	0.525237	0.97	0.356789
*Acadvl	A_51_P518340	NM_017366	acyl-Coenzyme A dehydrogenase, very long chain	1.79	8.97E-14	1.46	1.53E-17	2.37	0	3.00	0
Acat1	A_51_P319449	NM_144784	acetyl-Coenzyme A acetyltransferase 1	1.42	2.03E-17	1.13	0.0013	1.30	1.00E-10	1.45	7.33E-17
Acat2	A_51_P139748	NM_009338	acetyl-Coenzyme A acetyltransferase 2	1.70	1.97E-26	1.36	3.78E-16	1.53	0	1.77	0
Acat3	A_51_P139745	NM_153151	acetyl-Coenzyme A acetyltransferase 3	1.64	2.47E-28	1.32	1.26E-14	1.56	4.48E-44	1.74	0
Acat3	A_52_P11959	NM_153151	acetyl-Coenzyme A acetyltransferase 3	1.84	2.54E-23	1.47	3.04E-14	1.78	1.13E-27	1.93	2.07E-26
*Acly	A_51_P261718	NM_134037	ATP citrate lyase	7.28	0	2.11	1.72E-41	2.91	0	2.90	0
Acly	A_52_P373556	AK043466	ATP citrate lyase	3.42	7.18E-08	2.17	8.59E-28	2.49	1.63E-34	2.88	8.98E-13
Aco1	A_51_P391082	NM_007386	aconitase 1	1.77	0	1.37	8.08E-25	1.70	0	1.94	0
Aco1	A_52_P299115	NM_007386	aconitase 1	1.95	0	1.17	0.00943	1.53	0	1.83	0
Aco2	A_52_P200359	NM_080633	aconitase 2, mitochondrial	1.38	6.50E-21	1.18	5.86E-15	1.66	0	1.98	0
Aco2	A_52_P899263	AK029122	Mus musculus 10 days neonate skin cDNA, RIKEN full-length enriched library, clone:4732494K09	1.75	0.000022	0.82	0.065118	0.49	2.11E-12	0.60	1.69E-06
*Acox1	A_51_P366704	NM_015729	acyl-Coenzyme A oxidase 1, palmitoyl	1.38	7.60E-19	1.37	5.14E-30	2.06	0	2.37	0
Acox1	A_52_P615096	NM_015729	acyl-Coenzyme A oxidase 1, palmitoyl	1.08	0.246566	1.52	3.67E-15	2.58	1.30E-35	2.95	0
Acox2	A_51_P206708	NM_053115	acyl-Coenzyme A oxidase 2, branched chain	1.06	0.266203	0.93	0.525244	0.66	5.21E-10	1.26	0.001981
Acsl1	A_51_P463452	NM_007981	acyl-CoA synthetase long-chain family member 1	0.81	2.01E-09	1.63	7.23E-39	2.28	0	2.47	0
Acsl1	A_51_P496432	BC006692	acyl-CoA synthetase long-chain family member 1	0.72	2.51E-07	1.46	1.06E-06	1.80	8.04E-10	1.95	2.62E-16
Acsl1	A_52_P597618	NM_007981	acyl-CoA synthetase long-chain family member 1	0.84	2.39E-06	1.66	1.02E-30	2.37	0	2.51	0
Acsl3	A_51_P511560	NM_028817	acyl-CoA synthetase long-chain family member 3	0.72	2.25E-17	0.71	1.03E-33	0.73	6.14E-25	0.75	0
Acsl4	A_51_P268154	NM_019477	acyl-CoA synthetase long-chain family member 4	0.71	0	1.09	0.012564	0.96	0.087098	1.04	0.324966
Acsl4	A_52_P78203	NM_207625	acyl-CoA synthetase long-chain family member 4	0.73	7.22E-43	0.91	1.94E-07	0.80	1.02E-22	0.91	0.20608

Acsl6	A_51_P380699	NM_144823	acyl-CoA synthetase long-chain family member 6	0.76	0.004716	0.48	5.03E-25	0.69	0.073547	0.54	0.002169
Acsl6	A_51_P518823	AK147421	acyl-CoA synthetase long-chain family member 6	0.77	0.045444	0.40	1.30E-09	0.54	0.015007	0.45	6.85E-08
Acsl6	A_52_P204459	AK147421	acyl-CoA synthetase long-chain family member 6	0.71	0.184327	0.30	1.08E-16	0.58	0.040114	0.75	0.372019
Adcy1	A_51_P196718	NM_009622	adenylate cyclase 1	0.78	0.094192	0.73	0.019699	0.61	1.87E-06	0.56	0.004519
Adcy2	A_51_P337523	NM_153534	adenylate cyclase 2	0.99	0.805389	0.67	5.12E-08	0.80	0.343954	0.74	0.025816
Adcy2	A_52_P165610	NM_153534	adenylate cyclase 2	0.91	0.109069	0.64	2.28E-09	0.90	0.630131	0.62	0.010218
Adcy3	A_51_P484671	NM_138305	adenylate cyclase 3	0.75	6.13E-11	0.90	0.00195	1.02	0.64436	0.99	0.729287
Adcy3	A_52_P216525	NM_138305	adenylate cyclase 3	0.71	1.37E-12	0.93	0.040575	1.04	0.342114	0.95	0.234465
Adcy5	A_51_P199041	NM_001012765	adenylate cyclase 5	0.71	1.18E-07	1.27	1.80E-06	1.65	2.13E-16	1.85	5.32E-44
Adcy5	A_52_P449208	NM_001012765	adenylate cyclase 5	0.72	7.82E-08	1.26	8.07E-08	1.64	2.31E-43	1.79	7.41E-41
Adcy7	A_51_P441622	NM_007406	adenylate cyclase 7	0.58	2.18E-35	0.93	0.000528	0.87	0.00061	0.77	0.000061
Adcy7	A_52_P291	AK012436	adenylate cyclase 7	0.54	0	0.77	6.60E-09	0.72	0	0.60	2.69E-26
Adcy7	A_52_P586141	NM_001037724	Mus musculus 11 days embryo whole body cDNA, RIKEN full-length enriched library	0.51	0	0.90	0.006749	0.78	1.00E-07	0.64	1.51E-15
Adh1	A_51_P428555	NM_007409	alcohol dehydrogenase 1 (class I)	0.53	0	0.52	1.75E-13	0.41	0	0.62	1.18E-06
Adh1	A_52_P629895	NM_007409	alcohol dehydrogenase 1 (class I)	0.49	7.03E-40	0.52	1.86E-11	0.40	0	0.61	2.08E-08
Adh4	A_51_P189442	NM_011996	alcohol dehydrogenase 4 (class II), pi polypeptide	0.43	1.13E-12	1.11	0.007753	1.09	0.116031	1.14	0.136515
Adh6a	A_52_P276348	AK007397	alcohol dehydrogenase 6A (class V)	0.54	0.009564	1.40	0.42977	1.93	4.29E-08	0.86	0.189521
Adh7	A_51_P233797	NM_009626	alcohol dehydrogenase 7 (class IV), mu or sigma polypeptide	0.87	0.022823	1.06	0.758067	0.74	0.000032	1.45	0.000151
Agpat1	A_51_P322612	NM_018862	1-acylglycerol-3-phosphate O-acyltransferase 1 (lysophosphatidic acid acyltransferase, alpha)	1.45	2.53E-37	1.10	0.001214	1.30	0	1.38	0
Agpat1	A_52_P200465	NM_018862	1-acylglycerol-3-phosphate O-acyltransferase 1 (lysophosphatidic acid acyltransferase, alpha)	1.65	1.63E-33	1.15	4.13E-07	1.33	0	1.52	0
Agpat2	A_51_P238563	NM_026212	1-acylglycerol-3-phosphate O-acyltransferase 2 (lysophosphatidic acid acyltransferase, beta)	1.40	1.35E-10	1.33	8.95E-10	1.83	4.65E-38	2.09	0
Agpat2	A_51_P238565	NM_026212	1-acylglycerol-3-phosphate O-acyltransferase 2 (lysophosphatidic acid acyltransferase, beta)	1.40	1.07E-10	1.29	1.90E-08	1.82	0	2.07	0
*Agpat3	A_51_P425490	NM_053014	1-acylglycerol-3-phosphate O-acyltransferase 3	1.85	0	1.33	5.29E-40	1.65	0	2.11	0
Agpat3	A_52_P523569	NM_053014	1-acylglycerol-3-phosphate O-acyltransferase 3	1.28	2.20E-19	1.15	3.85E-13	1.35	1.40E-45	1.78	0
Agpat4	A_51_P346165	NM_026644	1-acylglycerol-3-phosphate O-acyltransferase 4 (lysophosphatidic acid acyltransferase, delta)	0.66	0	0.86	0.000154	0.80	3.11E-24	0.76	2.25E-16
Agpat4	A_52_P3029	NM_026644	1-acylglycerol-3-phosphate O-acyltransferase 4 (lysophosphatidic acid acyltransferase, delta)	0.67	0	0.89	0.000019	0.80	1.72E-38	0.76	1.06E-23
Agpat5	A_51_P361286	NM_026792	1-acylglycerol-3-phosphate O-acyltransferase 5 (lysophosphatidic acid acyltransferase, epsilon)	1.40	6.42E-32	1.05	0.442446	1.07	0.146953	0.84	0.000326
Agpat5	A_52_P572447	NM_026792	1-acylglycerol-3-phosphate O-acyltransferase 5 (lysophosphatidic acid acyltransferase, epsilon)	0.62	0	1.00	0.933406	0.95	0.000053	0.87	1.04E-15
*Agpat6	A_51_P335710	NM_018743	1-acylglycerol-3-phosphate O-acyltransferase 6 (lysophosphatidic acid acyltransferase, zeta)	1.14	0.053586	1.06	0.279785	1.34	0.001626	1.51	2.13E-07
Agpat6	A_51_P335716	NM_018743	1-acylglycerol-3-phosphate O-acyltransferase 6 (lysophosphatidic acid acyltransferase, zeta)	1.14	0.011291	1.02	0.706273	1.38	1.09E-11	1.50	7.17E-17

Agpat6	A_52_P24986	NM_018743	1-acylglycerol-3-phosphate O-acyltransferase 6 (lysophosphatidic acid acyltransferase, zeta)	1.25	1.16E-15	1.02	0.274867	1.19	8.99E-12	1.31	0
Alb	A_51_P160713	NM_009654	albumin	1.61	2.50E-20	0.91	0.259407	0.87	0.103915	1.13	0.422462
Aldh1a1	A_51_P334942	NM_013467	aldehyde dehydrogenase family 1, subfamily A1	0.59	1.36E-24	0.68	3.30E-19	0.63	0	0.52	1.60E-21
Aldh1a2	A_52_P58145	NM_009022	aldehyde dehydrogenase family 1, subfamily A2	0.92	0.357816	1.36	0.004352	1.30	1.89E-08	1.39	0.00009
Aldh1a3	A_52_P113518	AK086764	aldehyde dehydrogenase family 1, subfamily A3	0.88	0.126252	1.15	0.052183	1.23	0.001185	1.33	0.000159
Aldh1a3	A_52_P87843	NM_053080	aldehyde dehydrogenase family 1, subfamily A3	0.57	1.27E-12	1.15	0.000687	1.08	0.198151	1.14	0.009559
Aldh1a7	A_51_P383399	NM_011921	aldehyde dehydrogenase family 1, subfamily A7	1.48	0	1.41	8.19E-10	1.85	0	1.93	3.62E-40
Aldh1b1	A_51_P510418	NM_028270	aldehyde dehydrogenase 1 family, member B1	0.26	0	0.37	0	0.41	0	0.38	0
Aldh3a1	A_51_P310594	NM_007436	aldehyde dehydrogenase family 3, subfamily A1	0.95	0.594929	5.03	0.000023	0.88	0.403377	4.62	6.22E-07
Aldh3a2	A_51_P464175	NM_007437	aldehyde dehydrogenase family 3, subfamily A2	0.95	0.012784	1.23	0.004191	1.02	0.479465	1.65	1.07E-07
Aldh4a1	A_51_P337918	NM_175438	aldehyde dehydrogenase 4 family, member A1	2.20	0	1.32	5.93E-36	1.45	0	1.45	0
Aldh4a1	A_52_P123230	NM_175438	aldehyde dehydrogenase 4 family, member A1	1.48	0.001831	1.25	0.014334	1.53	3.93E-06	1.26	0.031732
Aldh5a1	A_51_P491504	NM_172532	aldhehyde dehydrogenase family 5, subfamily A1	1.60	0	1.33	8.38E-16	1.65	0	1.69	0
Aldh5a1	A_52_P30273	AK051603	aldhehyde dehydrogenase family 5, subfamily A1	2.50	6.94E-08	2.28	0.000092	1.36	0.13654	3.13	0.00007
Aldh7a1	A_52_P503071	NM_138600	aldehyde dehydrogenase family 7, member A1	1.30	0.010972	1.12	0.147386	1.45	0.000201	1.43	0.000019
Aldh9a1	A_51_P106211	NM_019993	aldehyde dehydrogenase 9, subfamily A1	1.03	0.440354	1.09	0.014949	1.34	6.55E-24	1.24	9.79E-10
Aldob	A_51_P337269	NM_144903	aldolase 2, B isoform	0.08	0	0.54	1.68E-19	0.46	2.46E-07	0.74	0.062678
Aldob	A_52_P450038	NM_144903	aldolase 2, B isoform	0.09	0	0.55	7.84E-20	0.45	4.84E-07	0.87	0.50957
Aldob	A_52_P468023	NM_144903	aldolase 2, B isoform	0.17	2.65E-25	0.51	8.07E-12	0.35	1.70E-07	0.79	0.103289
Aldoc	A_51_P220681	NM_009657	aldolase 3, C isoform	0.29	0	0.50	2.70E-12	0.40	0	0.64	0.00002
Aldoc	A_51_P425284	AK039267	aldolase 3, C isoform	0.16	0	0.56	4.90E-07	0.41	3.29E-29	0.74	0.00161
Apoa4	A_51_P327491	NM_007468	apolipoprotein A-IV	1.06	0.217866	1.33	5.98E-06	1.72	3.13E-28	1.59	1.84E-10
Apoa4	A_51_P327496	NM_007468	apolipoprotein A-IV	1.52	7.60E-08	0.77	0.000032	0.57	1.50E-29	0.63	2.57E-25
Apoa5	A_51_P259930	NM_080434	apolipoprotein A-V	0.65	1.27E-22	0.89	0.076361	0.81	0.001705	0.95	0.482612
Apoa5	A_52_P292404	NM_080434	apolipoprotein A-V	0.64	0.003239	0.80	0.170864	0.63	0.000396	1.20	0.461572
Apob	A_51_P413088	XM_137955	Mus musculus apolipoprotein B (Apob), mRNA.	0.59	0.000456	1.00	0.984225	0.78	0.005972	1.56	0.146049
Apob	A_51_P470542	XM_137955	Mus musculus apolipoprotein B (Apob), mRNA.	0.16	7.44E-38	0.57	0.003561	0.46	4.88E-09	0.82	0.325462
Apob	A_52_P441070	NM_009693	apolipoprotein B	0.13	1.82E-22	0.45	1.78E-14	0.53	0.000031	1.06	0.75443
Apoc1	A_51_P164504	NM_007469	apolipoprotein C-I	3.30	0	1.78	0	1.84	0	2.20	0
Apoc2	A_51_P334979	NM_009695	apolipoprotein C-II	0.54	0	1.24	0.000312	1.42	7.54E-34	1.27	4.09E-10
Apoc3	A_51_P310629	NM_023114	apolipoprotein C-III	2.63	3.46E-28	1.14	0.131834	1.34	1.70E-07	1.29	0.026807
Apoc3	A_52_P574306	NM_023114	apolipoprotein C-III	2.72	1.62E-17	1.27	0.001241	1.56	2.79E-09	1.39	0.014807
Apoe	A_51_P171999	NM_009696	apolipoprotein E	1.76	6.24E-18	1.10	0.074183	0.84	2.63E-06	0.87	0.000276
Atp12a	A_52_P329398	NM_138652	ATPase, H+/K+ transporting, nongastric, alpha polypeptide	0.11	0	0.29	0.002277	0.14	0	0.47	5.75E-10

*Atp5a1	A_52_P674489	NM_007505	ATP synthase, H+ transporting, mitochondrial F1 complex, alpha subunit, isoform 1	0.99	0.466908	1.06	0.171404	1.34	0.000012	1.45	0.000024
Atp5b	A_52_P328078	NM_016774	ATP synthase, H+ transporting mitochondrial F1 complex, beta subunit	1.11	0.356843	0.95	0.580589	1.33	0.037383	1.54	0.000785
*Atp5b	A_52_P553841	NM_016774	ATP synthase, H+ transporting mitochondrial F1 complex, beta subunit	1.38	1.13E-08	1.19	0.000225	1.62	1.65E-19	1.75	2.15E-40
Atp5c1	A_51_P378087	NM_020615	ATP synthase, H+ transporting, mitochondrial F1 complex, gamma polypeptide 1	1.52	2.87E-14	1.06	0.142672	1.31	1.99E-10	1.36	2.09E-10
Atp5e	A_51_P362054	NM_025983	ATP synthase, H+ transporting, mitochondrial F1 complex, epsilon subunit	1.47	7.85E-30	0.98	0.214906	1.13	1.36E-07	1.19	2.63E-40
Atp5h	A_51_P264186	NM_027862	ATP synthase, H+ transporting, mitochondrial F0 complex, subunit d	1.21	2.26E-30	1.07	0.000193	1.26	2.80E-45	1.41	2.65E-40
Atp5h	A_52_P640355	NM_027862	ATP synthase, H+ transporting, mitochondrial F0 complex, subunit d	1.23	3.53E-32	1.06	0.003234	1.26	1.37E-24	1.47	0
Atp5k	A_51_P301289	NM_007507	ATP synthase, H+ transporting, mitochondrial F1F0 complex, subunit e	2.08	0	1.25	1.54E-13	1.68	0	1.89	0
Atp6v0a1	A_51_P142089	NM_016920	ATPase, H+ transporting, lysosomal V0 subunit A1	0.50	0	1.00	0.941876	1.16	7.49E-31	1.32	9.59E-19
Atp6v0a1	A_52_P399998	NM_016920	ATPase, H+ transporting, lysosomal V0 subunit A1	0.35	0	1.05	0.176223	1.08	0.039803	1.19	3.75E-11
Atp6v0a1	A_52_P515336	AK045877	ATPase, H+ transporting, lysosomal V0 subunit A1	0.69	3.83E-15	0.84	0.000012	0.80	1.26E-07	0.87	0.00159
Atp6v1b2	A_51_P190845	NM_007509	ATPase, H+ transporting, lysosomal V1 subunit B2	0.59	3.09E-32	1.13	0.006734	1.10	0.029943	1.10	0.071469
Atp6v1c2	A_51_P518600	NM_133699	ATPase, H+ transporting, lysosomal V1 subunit C2	0.56	1.03E-22	1.05	0.583413	0.96	0.395648	1.88	0.004793
Atp6v1e1	A_51_P166434	NM_007510	VATPase, H+ transporting, lysosomal V1 subunit E1	0.86	0.000917	1.11	0.01802	1.25	0.000013	1.36	1.93E-11
Atp6v1e1	A_52_P303388	NM_007510	VATPase, H+ transporting, lysosomal V1 subunit E1	0.87	0.006562	1.11	0.01675	1.24	2.90E-07	1.34	1.04E-09
Atp6v1h	A_52_P403420	AK081492	ATPase, H+ transporting, lysosomal V1 subunit H	1.54	5.30E-25	1.47	1.31E-13	1.46	1.65E-15	1.45	1.39E-10
Auh	A_51_P372473	NM_016709	AU RNA binding protein/enoyl-coenzyme A hydratase	1.52	1.49E-35	1.10	0.002853	1.14	2.10E-07	1.14	0.00002
Auh	A_52_P107290	AK003929	AU RNA binding protein/enoyl-coenzyme A hydratase	1.39	1.84E-09	1.15	0.012556	1.38	4.36E-13	1.26	9.30E-08
Auh	A_52_P4095	AK169661	AU RNA binding protein/enoyl-coenzyme A hydratase	1.35	7.61E-23	1.02	0.563088	1.19	2.40E-08	1.08	0.038711
Bdh1	A_51_P163106	NM_175177	3-hydroxybutyrate dehydrogenase, type 1	0.27	3.76E-25	0.22	0	0.24	4.39E-26	0.38	1.54E-13
Bdh1	A_52_P468343	AK146962	3-hydroxybutyrate dehydrogenase, type 1	0.55	1.66E-09	0.48	2.53E-36	0.45	7.38E-43	0.54	1.29E-23
Bdh2	A_51_P470935	NM_027208	3-hydroxybutyrate dehydrogenase, type 2	1.69	9.17E-16	1.08	0.046784	0.98	0.445862	0.83	0.000161
Bdh2	A_52_P195809	NM_027208	3-hydroxybutyrate dehydrogenase, type 2	1.50	2.08E-08	1.17	1.94E-08	1.11	0.001263	0.92	0.102139
Btd	A_51_P196158	NM_025295	biotinidase	1.08	0.005504	1.17	1.89E-09	1.29	8.06E-38	1.33	7.00E-32
Cat	A_52_P679769	NM_009804	catalase	1.23	1.09E-06	1.28	2.06E-06	1.80	6.59E-43	1.77	0
Cd36	A_51_P375138	L23108	CD36 antigen	0.64	0	1.09	0.001594	1.00	0.844402	1.02	0.529989
*Cd36	A_51_P375146	NM_007643	CD36 antigen	0.62	1.40E-45	0.96	0.88613	0.97	0.300512	0.96	0.150383
*Cox1	A_51_P315595	X57780	M.musculus mRNA for mitochondrial gene for subunit I of cytochrome c oxidase.	1.42	3.18E-06	1.14	8.75E-08	1.75	6.46E-22	1.92	5.67E-15
Cox4i1	A_51_P165435	NM_009941	cytochrome c oxidase subunit IV isoform 1	1.20	1.66E-06	1.07	0.118701	1.40	2.31E-19	1.64	0
Cox6a1	A_51_P311540	NM_007748	cytochrome c oxidase, subunit VI a, polypeptide 1	0.95	0.558589	1.19	0.010689	1.35	0.008191	1.36	0.000064
Cox6b1	A_51_P448032	NM_025628	cytochrome c oxidase, subunit VIb polypeptide 1	1.54	2.99E-34	1.39	5.96E-24	1.82	0	2.16	0

Cox6b2	A_51_P300506	NM_183405	cytochrome c oxidase subunit VIb polypeptide 2	1.35	0	1.08	0.012779	1.37	0	1.59	0
Cox7a1	A_51_P148612	NM_009944	cytochrome c oxidase, subunit VIIa 1	1.37	4.83E-06	0.66	1.82E-29	1.45	7.69E-18	1.98	0
Cox7b	A_51_P160664	NM_025379	cytochrome c oxidase subunit VIIb	1.96	5.42E-32	1.33	5.47E-16	1.87	2.94E-44	2.15	0
Cpt1a	A_51_P427674	NM_013495	carnitine palmitoyltransferase 1a, liver	0.55	0	1.00	0.984125	0.99	0.702855	1.00	0.878462
Cpt1a	A_52_P219753	NM_013495	carnitine palmitoyltransferase 1a, liver	0.52	0	1.13	0.002937	1.11	0.001723	1.19	6.08E-06
Cpt1b	A_51_P232913	NM_009948	carnitine palmitoyltransferase 1b, muscle	0.93	0.31128	1.12	0.001497	2.06	0	3.07	0
Cpt1b	A_52_P625508	NM_009948	carnitine palmitoyltransferase 1b, muscle	0.92	0.198682	1.32	3.57E-18	2.31	0	3.25	0
*Cpt2	A_51_P403388	NM_009949	carnitine palmitoyltransferase 2	1.76	4.81E-27	1.36	7.84E-16	1.77	3.53E-31	1.95	2.80E-45
*Crat	A_51_P440807	NM_007760	carnitine acetyltransferase	1.17	0.00104	1.08	0.025608	1.27	6.84E-12	1.37	0
*Cs	A_51_P390260	NM_026444	citrate synthase	1.96	7.01E-45	1.58	3.38E-27	2.28	0	2.52	0
Cs	A_52_P182659	NM_026444	citrate synthase	1.02	0.610386	1.30	1.55E-10	1.74	3.18E-13	1.78	6.44E-15
Cyp1a1	A_51_P279693	NM_009992	cytochrome P450, family 1, subfamily a, polypeptide 1	2.38	4.47E-29	0.74	0.002613	0.52	3.19E-34	1.01	0.944742
Cyp2a12	A_51_P206736	NM_133657	cytochrome P450, family 2, subfamily a, polypeptide 12	0.73	0.196501	1.02	0.951948	0.66	0.05122	2.50	0.008273
Cyp2a5	A_52_P246252	NM_007812	cytochrome P450, family 2, subfamily a, polypeptide 5	0.17	0.000019	0.51	0.000013	0.35	1.53E-14	1.04	0.810724
Cyp2b10	A_51_P182362	NM_009998	Mus musculus cytochrome P450, family 2, subfamily b, polypeptide 10 (Cyp2b10), transcript variant 2, mRNA.	0.53	2.61E-10	0.96	0.362713	1.03	0.555584	0.99	0.842969
Cyp2b10	A_52_P472486	NM_009999	cytochrome P450, family 2, subfamily b, polypeptide 10	0.34	1.20E-18	0.81	0.000066	0.98	0.764697	0.94	0.320957
Cyp2b13	A_51_P492339	NM_007813	cytochrome P450, family 2, subfamily b, polypeptide 13	0.21	0	0.97	0.688834	1.08	0.160102	1.37	1.24E-22
Cyp2b13	A_52_P289091	NM_007813	cytochrome P450, family 2, subfamily b, polypeptide 13	0.84	2.38E-07	1.13	0.00404	1.36	2.94E-11	1.41	2.65E-15
Cyp2b19	A_51_P352763	NM_007814	cytochrome P450, family 2, subfamily b, polypeptide 19	0.94	0.054748	1.41	0.008078	1.14	0.000045	2.55	9.21E-06
Cyp2c29	A_51_P103706	NM_007815	cytochrome P450, family 2, subfamily c, polypeptide 29	0.08	0	0.18	0	0.17	0	0.27	0
Cyp2c39	A_51_P304109	NM_010003	cytochrome P450, family 2, subfamily c, polypeptide 39	0.09	0	0.12	0	0.12	0	0.15	0
Cyp2c44	A_51_P209782	NM_001001446	cytochrome P450, family 2, subfamily c, polypeptide 44	0.49	4.45E-16	1.34	5.96E-10	1.14	0.015473	0.83	0.001953
Cyp2c54	A_52_P154580	NM_206537	cytochrome P450, family 2, subfamily c, polypeptide 54	0.76	0.165844	0.62	0.036596	0.43	1.19E-08	0.86	0.639185
Cyp2c55	A_51_P447785	NM_028089	cytochrome P450, family 2, subfamily c, polypeptide 55	0.21	0	0.19	0	0.17	0	0.15	0
Cyp2c65	A_52_P652059	AK008688	cytochrome P450, family 2, subfamily c, polypeptide 65	0.14	0	0.14	0	0.11	0	0.11	0
Cyp2c66	A_51_P471126	NM_001011707	cytochrome P450, family 2, subfamily c, polypeptide 66	0.33	0	0.33	0	0.30	0	0.23	0
Cyp2c70	A_51_P134142	NM_145499	cytochrome P450, family 2, subfamily c, polypeptide 70	0.78	4.50E-15	1.27	1.97E-08	1.45	1.68E-33	1.60	2.48E-35
Cyp2d10	A_51_P111192	NM_010005	cytochrome P450, family 2, subfamily d, polypeptide 10	0.46	0	0.57	0	0.54	0	0.60	2.88E-33
Cyp2d10	A_52_P527775	NM_010005	cytochrome P450, family 2, subfamily d, polypeptide 10	0.52	0	0.53	0	0.66	4.20E-44	0.61	6.25E-21
Cyp2d13	A_52_P372062	NM_133695	Mus musculus cytochrome P450, family 2, subfamily d, polypeptide 13 (Cyp2d13) on chromosome 15.	0.57	8.95E-19	0.62	7.66E-18	0.61	0	0.64	5.20E-17
Cyp2d22	A_51_P124126	NM_019823	cytochrome P450, family 2, subfamily d, polypeptide 22	0.59	0	0.82	1.45E-06	0.84	1.18E-06	0.81	2.48E-09
Cyp2d22	A_52_P478420	AK086555	cytochrome P450, family 2, subfamily d, polypeptide 22	1.00	0.910578	0.94	0.143409	0.77	8.50E-10	0.69	4.07E-28
Cyp2d22	A_52_P49391	AK090296	cytochrome P450, family 2, subfamily d, polypeptide 22	0.60	0	0.80	5.03E-13	0.79	6.37E-09	0.70	2.70E-43
Cyp2d9	A_51_P124133	NM_010006	cytochrome P450, family 2, subfamily d, polypeptide 9	0.43	0	0.52	0	0.49	0	0.50	0

Cyp2d9	A_51_P478303	NM_010006	cytochrome P450, family 2, subfamily d, polypeptide 9	0.58	0	0.64	8.64E-41	0.62	1.12E-44	0.64	0
Cyp2e1	A_51_P283456	NM_021282	cytochrome P450, family 2, subfamily e, polypeptide 1	8.01	0	0.95	0.577506	1.69	9.36E-08	1.53	2.80E-22
Cyp2f2	A_51_P453909	NM_007817	cytochrome P450, family 2, subfamily f, polypeptide 2	1.00	0.99556	0.32	0.00813	0.08	0	0.48	1.37E-13
Cyp2j5	A_51_P307872	NM_010007	cytochrome P450, family 2, subfamily j, polypeptide 5	0.37	1.09E-09	0.98	0.917797	0.74	0.123239	1.54	0.100907
Cyp2j5	A_52_P315766	NM_010007	cytochrome P450, family 2, subfamily j, polypeptide 5	0.28	3.93E-17	1.06	0.725807	0.63	0.052138	2.09	0.042897
Cyp2j6	A_51_P506328	NM_010008	cytochrome P450, family 2, subfamily j, polypeptide 6	0.56	0	0.85	3.16E-16	0.80	9.22E-20	0.78	7.32E-30
Cyp2j9	A_51_P330044	NM_028979	cytochrome P450, family 2, subfamily j, polypeptide 9	0.83	4.22E-11	0.81	0.00009	0.76	7.59E-23	0.71	3.96E-17
Cyp2s1	A_51_P180091	NM_028775	cytochrome P450, family 2, subfamily s, polypeptide 1	0.06	0	0.25	7.71E-23	0.18	0	0.31	4.99E-26
Cyp3a11	A_51_P355301	NM_007818	cytochrome P450, family 3, subfamily a, polypeptide 11	0.63	3.83E-34	0.92	0.118121	0.99	0.880588	1.27	0.025105
Cyp3a13	A_51_P114941	NM_007819	cytochrome P450, family 3, subfamily a, polypeptide 13	0.06	0	0.08	0	0.05	0	0.08	0
Cyp3a16	A_51_P482051	NM_007820	cytochrome P450, family 3, subfamily a, polypeptide 16	0.69	0.009302	1.07	0.515747	1.34	0.001489	1.85	0.000115
Cyp3a25	A_51_P489367	NM_019792	cytochrome P450, family 3, subfamily a, polypeptide 25	0.13	0	0.92	0.658534	0.73	0.011648	1.39	2.37E-06
Cyp3a44	A_52_P571006	NM_177380	cytochrome P450, family 3, subfamily a, polypeptide 44	0.32	1.43E-11	1.02	0.900719	1.34	0.063993	2.44	2.58E-11
Cyp4a10	A_52_P257774	NM_010011	cytochrome P450, family 4, subfamily a, polypeptide 10	0.64	0.000127	1.11	0.613712	1.32	0.023382	1.99	4.79E-14
Cyp4a10	A_52_P340136	X71478	cytochrome P450, family 4, subfamily a, polypeptide 10	0.64	0	0.99	0.662613	1.20	0.000048	1.32	1.12E-12
Cyp4a12a	A_51_P433360	NM_177406	cytochrome P450, family 4, subfamily a, polypeptide 12a	0.53	0.000028	1.03	0.822771	1.37	0.028421	3.72	1.32E-13
Cyp4a12a	A_52_P44914	NM_177406	cytochrome P450, family 4, subfamily a, polypeptide 12a	0.96	0.470709	1.18	0.020545	1.13	0.001775	1.54	5.97E-06
Cyp4a14	A_51_P238576	NM_007822	cytochrome P450, family 4, subfamily a, polypeptide 14	0.43	2.66E-44	1.12	0.250461	1.22	0.024474	1.80	0.000437
Cyp4b1	A_51_P118704	NM_007823	cytochrome P450, family 4, subfamily b, polypeptide 1	0.52	0	1.06	0.164935	1.37	2.52E-12	1.76	0
Cyp4f14	A_51_P452768	NM_022434	cytochrome P450, family 4, subfamily f, polypeptide 14	0.32	0	0.17	0	0.14	0	0.14	0
Cyp4f15	A_51_P136303	NM_134127	cytochrome P450, family 4, subfamily f, polypeptide 15	0.96	0.727041	13.53	2.29E-07	1.82	0.060869	9.24	8.58E-20
Cyp51	A_51_P485791	NM_020010	cytochrome P450, family 51	0.52	0	1.36	4.60E-20	1.35	4.60E-14	1.37	1.66E-13
Cyp51	A_52_P164161	NM_020010	cytochrome P450, family 51	0.59	1.07E-22	1.16	0.003806	1.12	0.006027	1.17	0.00003
Cyp51	A_52_P636752	NM_020010	cytochrome P450, family 51	0.50	0	1.32	2.89E-14	1.25	3.79E-09	1.26	8.85E-14
*Dci	A_51_P105589	NM_010023	dodecenoyl-Coenzyme A delta isomerase (3,2 trans-enoyl-Coenyme A isomerase)	1.68	7.62E-29	1.26	3.86E-14	1.68	0	1.88	0
Decr1	A_51_P208555	NM_026172	2,4-dienoyl CoA reductase 1, mitochondrial	1.32	2.48E-10	1.49	4.97E-13	2.14	0	2.25	0
Decr1	A_52_P335907	NM_026172	2,4-dienoyl CoA reductase 1, mitochondrial	1.32	1.83E-12	1.55	4.02E-16	2.49	0	2.56	0
*Dgat1	A_51_P510059	NM_010046	diacylglycerol O-acyltransferase 1	1.28	0.000018	1.24	4.08E-16	1.70	7.27E-21	1.78	4.01E-27
Dgat1	A_52_P456134	NM_010046	diacylglycerol O-acyltransferase 1	1.43	0	1.29	0	1.57	0	1.75	0
Dgat2	A_51_P396003	NM_026384	diacylglycerol O-acyltransferase 2	1.05	0.106841	1.32	1.76E-06	1.25	7.51E-11	1.09	0.032628
Dlat	A_51_P265106	NM_145614	dihydrolipoamide S-acetyltransferase (E2 component of pyruvate dehydrogenase complex)	1.77	9.44E-27	1.49	2.80E-45	2.10	0	2.72	0
Dlat	A_52_P374642	NM_145614	dihydrolipoamide S-acetyltransferase (E2 component of pyruvate dehydrogenase complex)	1.46	1.05E-18	1.72	0	2.75	0	3.41	0
Dld	A_51_P184282	U73445	dihydrolipoamide dehydrogenase	1.42	2.52E-28	1.30	3.29E-12	1.88	0	2.11	0
Dld	A_51_P184284	NM_007861	dihydrolipoamide dehydrogenase	1.36	3.35E-31	1.33	1.66E-14	1.88	0	2.07	0

Dld	A_52_P536646	NM_007861	dihydrolipoamide dehydrogenase	1.06	0.478605	1.19	0.002094	1.83	0	2.09	0
Dlst	A_51_P290139	NM_030225	dihydrolipoamide S-succinyltransferase (E2 component of 2-oxo-glutarate complex)	1.43	2.30E-20	1.16	0.000016	1.63	3.19E-43	1.83	2.03E-31
Dlst	A_52_P140072	NM_030225	dihydrolipoamide S-succinyltransferase (E2 component of 2-oxo-glutarate complex)	1.34	4.97E-07	1.21	0.000484	1.72	2.91E-25	1.94	1.71E-30
Dlst	A_52_P3383	AK171100	dihydrolipoamide S-succinyltransferase (E2 component of 2-oxo-glutarate complex)	1.34	2.40E-14	0.86	0.000415	0.92	0.000487	1.00	0.98328
Ech1	A_51_P421846	NM_016772	enoyl coenzyme A hydratase 1, peroxisomal	1.01	0.687831	1.09	0.000067	1.30	3.22E-22	1.40	8.18E-32
*Echs1	A_51_P409039	NM_053119	enoyl Coenzyme A hydratase, short chain, 1, mitochondrial	1.84	0	1.22	1.77E-22	1.47	0	1.59	0
*Ehhadh	A_51_P462918	NM_023737	enoyl-Coenzyme A, hydratase/3-hydroxyacyl Coenzyme A dehydrogenase	1.61	0	1.45	7.20E-24	2.75	0	3.66	0
Eno2	A_51_P130028	NM_013509	enolase 2, gamma neuronal	0.75	3.76E-15	0.84	0.000113	0.78	1.81E-08	0.69	1.15E-14
Eno2	A_52_P748882	NM_013509	enolase 2, gamma neuronal	0.67	5.96E-25	0.86	0.005395	0.79	7.89E-06	0.64	6.37E-12
Eno3	A_51_P328539	NM_007933	enolase 3, beta muscle	1.58	0.000042	0.41	1.63E-11	0.54	0.052807	0.62	0.02941
Fabp1	A_51_P487818	NM_017399	fatty acid binding protein 1, liver	0.14	3.83E-09	1.13	0.228753	0.99	0.905421	1.54	0.140999
Fabp2	A_51_P313581	NM_007980	fatty acid binding protein 2, intestinal	0.52	0	0.57	9.77E-16	0.58	0	0.68	1.55E-08
Fabp2	A_52_P453013	NM_007980	fatty acid binding protein 2, intestinal	0.64	0.01274	0.45	0.021387	0.34	7.56E-34	0.37	1.09E-14
Fabp3	A_51_P167535	NM_010174	fatty acid binding protein 3, muscle and heart	0.60	2.79E-06	0.62	5.97E-15	1.20	0.000045	1.82	1.39E-39
Fabp5	A_51_P387764	NM_010634	fatty acid binding protein 5, epidermal	0.91	0.109156	1.59	9.96E-25	2.05	0	2.15	0
Fasn	A_51_P321126	NM_007988	fatty acid synthase	6.27	0	2.20	1.03E-09	3.50	0	2.92	0
*Fasn	A_52_P100252	NM_007988	fatty acid synthase	7.73	0	2.57	1.21E-43	3.40	0	3.16	0
Fbp1	A_51_P474701	NM_019395	fructose bisphosphatase 1	0.19	0	0.62	2.04E-21	0.50	5.25E-13	0.81	0.000476
Fdft1	A_51_P485946	NM_010191	farnesyl diphosphate farnesyl transferase 1	1.15	7.56E-07	0.82	5.37E-14	0.78	2.79E-12	0.74	0
Fdft1	A_52_P136138	NM_010191	farnesyl diphosphate farnesyl transferase 1	0.74	0.000017	1.03	0.615007	0.92	0.391482	0.95	0.44805
Fdps	A_51_P379798	NM_134469	farnesyl diphosphate synthetase	0.71	5.02E-16	1.10	0.089033	1.09	0.009047	1.13	0.017302
Fdps	A_52_P593965	AK077979	farnesyl diphosphate synthetase	0.89	0.195482	0.84	0.000392	0.75	1.85E-13	1.08	0.104434
*Fh1	A_51_P359333	NM_010209	fumarate hydratase 1	1.67	0	1.24	1.54E-44	1.62	0	1.79	0
Fnta	A_51_P112116	NM_008033	farnesyltransferase, CAAX box, alpha	1.36	0	1.14	2.02E-08	1.24	2.76E-30	1.28	3.45E-40
Fntb	A_51_P517680	NM_145927	farnesyltransferase, CAAX box, beta	1.48	3.00E-38	1.43	7.61E-17	1.55	6.82E-13	1.49	1.25E-23
G6pd2	A_51_P353735	NM_019468	glucose-6-phosphate dehydrogenase 2	0.52	2.95E-36	1.04	0.490293	1.34	1.26E-09	1.26	3.30E-06
G6pdx	A_51_P187082	NM_008062	glucose-6-phosphate dehydrogenase X-linked	0.56	1.64E-43	0.95	0.227074	1.16	0.004811	1.21	3.10E-06
Gapdh	A_52_P589321	AK169742	glyceraldehyde-3-phosphate dehydrogenase	1.06	0.352271	0.97	0.425108	1.20	0.000285	1.40	9.75E-07
Gck	A_51_P106294	NM_010292	glucokinase	1.93	6.19E-07	0.97	0.793299	1.15	0.185138	1.07	0.592539
Gck	A_52_P259537	NM_010292	glucokinase	1.85	1.04E-14	1.10	0.256474	1.14	0.335489	1.22	0.000102
Ggps1	A_52_P228247	NM_010282	geranylgeranyl diphosphate synthase 1	0.76	2.37E-21	1.27	1.64E-14	1.21	2.22E-11	1.16	3.02E-09
*Gpd1	A_51_P293853	NM_010271	glycerol-3-phosphate dehydrogenase 1 (soluble)	2.30	7.61E-43	2.37	4.74E-39	4.02	0	4.35	0
Gpd1	A_52_P16419	NM_010271	glycerol-3-phosphate dehydrogenase 1 (soluble)	1.58	0.000013	1.96	1.28E-09	3.39	3.52E-33	3.50	2.31E-23

Gpt2	A_51_P493886	NM_173866	glutamic pyruvate transaminase (alanine aminotransferase) 2	2.35	0	0.83	6.61E-08	0.94	0.006933	0.78	1.57E-18
Gyg	A_51_P240019	NM_013755	glycogenin	1.03	0.405407	1.36	9.99E-16	1.56	0	1.52	3.27E-36
Gyk	A_51_P297671	NM_008194	glycerol kinase	0.53	0	2.32	0	3.93	0	5.33	0
Gyk	A_52_P1020291	AK037633	Mus musculus 16 days neonate thymus cDNA, RIKEN full-length enriched library, clone:A130030N17	0.64	2.50E-13	0.99	0.916818	1.24	0.001514	1.82	9.84E-36
Gyk	A_52_P120066	NM_212444	glycerol kinase	0.46	0	2.00	8.33E-36	3.28	0	4.27	0
Gyk	A_52_P453517	NM_008194	glycerol kinase	0.38	9.99E-11	2.03	1.96E-10	3.34	0	4.10	0
Gys1	A_52_P228932	NM_030678	glycogen synthase 1, muscle	1.67	8.06E-34	1.09	0.041643	1.23	0.000097	1.38	1.56E-14
Gys2	A_51_P440315	NM_145572	glycogen synthase 2	4.32	0	4.01	0	7.11	0	9.53	0
Hadha	A_52_P193029	NM_178878	hydroxyacyl-Coenzyme A dehydrogenase/3-ketoacyl-Coenzyme A thiolase/enoyl-Coenzyme A hydratase, alpha subunit	1.27	0.000044	1.44	1.66E-11	1.93	6.98E-42	2.43	0
Hadha	A_52_P338459	AK035316	hydroxyacyl-Coenzyme A dehydrogenase/3-ketoacyl-Coenzyme A thiolase/enoyl-Coenzyme A hydratase, alpha subunit	1.74	0.003632	1.64	0.00065	1.29	0.029932	2.21	7.03E-21
Hadha	A_52_P658044	AK050856	hydroxyacyl-Coenzyme A dehydrogenase/3-ketoacyl-Coenzyme A thiolase/enoyl-Coenzyme A hydratase, alpha subunit	1.18	0.047648	0.84	0.011521	1.04	0.481568	1.40	2.37E-09
*Hadhb	A_51_P217990	NM_145558	hydroxyacyl-Coenzyme A dehydrogenase/3-ketoacyl-Coenzyme A thiolase/enoyl-Coenzyme A hydratase, beta subunit	1.43	0.000205	1.38	1.08E-20	2.01	0	2.34	0
Hk1	A_52_P102773	BC072628	hexokinase 1	0.73	2.51E-40	0.76	2.21E-41	0.69	0	0.78	7.17E-24
Hk1	A_52_P479599	NM_010438	hexokinase 1	0.67	1.84E-07	0.79	0.000047	0.82	0.000066	0.90	0.00309
Hk1	A_52_P674530	NM_010438	hexokinase 1	0.72	1.45E-11	0.83	4.61E-25	0.81	2.47E-36	0.83	5.85E-14
Hk1	A_52_P87141	NM_010438	hexokinase 1	1.05	0.896584	2.14	0.000741	1.15	0.574305	1.44	0.024512
Hk1	A_52_P9337	NM_010438	hexokinase 1	1.14	0.083664	1.13	0.048549	1.36	9.13E-06	1.20	0.023784
Hmgcr	A_51_P507410	NM_008255	3-hydroxy-3-methylglutaryl-Coenzyme A reductase	0.26	0	1.07	0.384694	0.91	0.071765	1.25	0.042226
Hmgcr	A_52_P137371	NM_008255	3-hydroxy-3-methylglutaryl-Coenzyme A reductase	0.36	0	1.09	0.307084	0.83	2.82E-08	1.46	0.009554
Hmgcr	A_52_P232287	NM_008255	3-hydroxy-3-methylglutaryl-Coenzyme A reductase	0.33	0	1.11	0.103932	0.96	0.237023	1.39	0.002
Hmgcr	A_52_P578922	AK159899	3-hydroxy-3-methylglutaryl-Coenzyme A reductase	0.57	0	1.20	0.028011	0.77	7.64E-09	1.53	0.008988
Hmgcr	A_52_P770765	AK033165	Mus musculus 15 days embryo male testis cDNA, RIKEN full-length enriched library, clone:8030450I20	0.57	0	1.09	0.291929	0.79	0.000077	1.25	0.024748
Hmgcs1	A_51_P146941	NM_145942	3-hydroxy-3-methylglutaryl-Coenzyme A synthase 1	0.65	2.59E-24	1.27	4.00E-20	1.34	6.68E-27	1.63	0
Hmgcs1	A_52_P119039	AK078743	3-hydroxy-3-methylglutaryl-Coenzyme A synthase 1	1.28	0.000061	1.38	2.66E-18	1.22	6.36E-09	1.91	0
Hmgcs1	A_52_P388072	NM_145942	3-hydroxy-3-methylglutaryl-Coenzyme A synthase 1	0.68	5.30E-09	1.24	7.66E-18	1.52	0	1.75	1.24E-29
Hmgcs2	A_51_P116039	NM_008256	3-hydroxy-3-methylglutaryl-Coenzyme A synthase 2	0.70	6.16E-15	0.51	2.45E-16	0.44	0	0.56	1.44E-17
Hsd17b4	A_51_P445662	NM_008292	hydroxysteroid (17-beta) dehydrogenase 4	0.87	3.91E-15	1.23	1.14E-23	1.45	1.30E-35	1.51	5.72E-39
Idh1	A_51_P132978	NM_010497	isocitrate dehydrogenase 1 (NADP+), soluble	1.30	2.94E-06	1.20	0.000123	1.44	9.30E-10	1.59	1.67E-16
Idh3a	A_51_P268559	NM_029573	isocitrate dehydrogenase 3 (NAD+) alpha	1.05	0.404739	1.42	8.26E-10	1.98	1.41E-22	2.32	0

Idh3b	A_51_P130110	NM_130884	isocitrate dehydrogenase 3 (NAD+) beta	1.49	3.50E-38	1.18	3.05E-06	1.73	0	1.91	0
Idh3g	A_51_P136729	NM_008323	isocitrate dehydrogenase 3 (NAD+), gamma	1.67	0	1.18	7.65E-08	1.59	0	1.91	0
Idi1	A_52_P441634	NM_177960	isopentenyl-diphosphate delta isomerase	0.66	7.58E-26	1.34	0.017993	1.18	0.000134	2.59	0.000029
Ldha	A_51_P364146	NM_010699	lactate dehydrogenase A	1.32	9.85E-43	1.01	0.72351	1.07	0.009341	1.07	1.24E-07
Ldhb	A_51_P409173	NM_008492	lactate dehydrogenase B	1.76	0	1.07	0.070957	1.36	2.59E-23	1.81	0
Ldhc	A_51_P242403	NM_013580	lactate dehydrogenase C	1.00	1	0.99	0.900343	9.87	5.24E-27	36.24	1.00E-18
Mcat	A_51_P314855	NM_001030014	malonyl CoA:ACP acyltransferase (mitochondrial)	1.57	4.07E-34	1.29	3.84E-19	1.54	0	1.66	0
Mdh1	A_51_P218179	NM_008618	malate dehydrogenase 1, NAD (soluble)	1.52	0	1.40	3.88E-23	1.96	0	2.30	0
Mdh1	A_52_P407796	NM_008618	malate dehydrogenase 1, NAD (soluble)	1.63	1.06E-16	1.44	2.93E-14	2.01	5.33E-21	2.17	7.81E-29
Mdh1	A_52_P470451	AK077724	malate dehydrogenase 1, NAD (soluble)	1.93	1.70E-43	1.17	0.003558	1.91	0	1.95	0
*Mdh2	A_51_P384879	NM_008617	malate dehydrogenase 2, NAD (mitochondrial)	1.17	0.016985	1.21	0.000345	1.67	1.71E-11	1.89	1.12E-14
Mecr	A_51_P386080	NM_025297	mitochondrial trans-2-enoyl-CoA reductase	1.20	7.13E-16	1.21	7.43E-06	1.61	0	1.70	2.08E-29
Mgll	A_51_P139920	NM_011844	monoglyceride lipase	0.65	4.29E-21	1.19	0.000015	1.42	2.76E-06	1.50	5.83E-12
Mgll	A_52_P568977	AK006949	monoglyceride lipase	1.68	3.89E-22	1.48	1.57E-17	1.75	1.12E-21	2.28	0
*Mgll	A_52_P609778	NM_011844	monoglyceride lipase	1.57	2.82E-41	1.47	2.31E-17	1.95	4.21E-13	2.25	9.44E-19
Mut	A_51_P193935	NM_008650	methylmalonyl-Coenzyme A mutase	1.83	0	1.19	3.68E-06	1.30	3.21E-31	1.32	7.73E-17
Mut	A_52_P633163	NM_008650	methylmalonyl-Coenzyme A mutase	2.08	0	1.23	1.43E-14	1.36	0	1.41	2.79E-43
Mvd	A_51_P355943	NM_138656	mevalonate (diphospho) decarboxylase	0.74	2.46E-11	1.37	1.74E-10	1.28	3.91E-09	1.48	1.56E-30
Ndufa1	A_51_P472405	NM_019443	NADH dehydrogenase (ubiquinone) 1 alpha subcomplex, 1	1.44	1.67E-12	1.13	0.004442	1.42	2.92E-13	1.52	2.27E-20
Ndufa1	A_52_P139747	NM_019443	NADH dehydrogenase (ubiquinone) 1 alpha subcomplex, 1	1.35	9.93E-16	1.09	2.80E-06	1.31	3.39E-19	1.46	2.23E-29
Ndufa5	A_51_P170156	NM_026614	NADH dehydrogenase (ubiquinone) 1 alpha subcomplex, 5	1.43	3.05E-24	0.97	0.195335	1.15	1.92E-10	1.31	5.32E-32
Ogdh	A_51_P122825	NM_010956	oxoglutarate dehydrogenase (lipoamide)	1.22	3.45E-21	1.01	0.593564	1.27	2.43E-37	1.41	4.55E-28
Ogdh	A_52_P369310	NM_010956	oxoglutarate dehydrogenase (lipoamide)	1.26	3.66E-12	1.04	0.188474	1.28	4.41E-19	1.41	3.85E-23
Oxct1	A_51_P107321	NM_024188	3-oxoacid CoA transferase 1	1.47	3.80E-12	1.29	2.32E-07	1.25	1.72E-07	1.12	0.02451
Oxct1	A_51_P107326	NM_024188	3-oxoacid CoA transferase 1	1.31	2.18E-34	1.25	5.09E-14	1.18	1.10E-11	1.08	0.006906
Oxct1	A_52_P430058	NM_024188	3-oxoacid CoA transferase 1	1.41	6.46E-10	1.29	1.37E-09	1.25	5.79E-08	1.16	0.001094
Oxct2a	A_51_P125056	NM_022033	3-oxoacid CoA transferase 2A	1.13	0.433155	1.27	0.074115	1.80	0.00009	2.33	0.006431
Oxsm	A_51_P312748	NM_027695	3-oxoacyl-ACP synthase, mitochondrial	1.37	1.91E-26	1.40	8.27E-08	1.75	0	2.17	0
Oxsm	A_52_P231635	NM_027695	3-oxoacyl-ACP synthase, mitochondrial	1.17	6.10E-07	1.47	3.69E-15	2.15	0	2.62	0
Pcca	A_51_P484254	NM_144844	propionyl-Coenzyme A carboxylase, alpha polypeptide	2.34	0	1.41	2.14E-12	1.78	0	1.72	4.74E-27
Pccb	A_51_P418259	NM_025835	propionyl Coenzyme A carboxylase, beta polypeptide	1.80	0	1.04	0.10855	1.16	4.03E-08	1.21	2.18E-09
Pcx	A_51_P480578	NM_008797	pyruvate carboxylase	2.53	0	1.67	8.92E-28	1.95	2.69E-38	2.20	0
Pdha1	A_51_P321391	NM_008810	pyruvate dehydrogenase E1 alpha 1	2.92	0	1.46	0	2.15	0	2.54	0
Pdha1	A_52_P581435	NM_008810	pyruvate dehydrogenase E1 alpha 1	2.30	0	1.28	1.39E-28	1.67	0	1.88	0
Pdhb	A_51_P321921	NM_024221	pyruvate dehydrogenase (lipoamide) beta	2.04	0	1.65	0	2.32	0	2.82	0

Pdhb	A_52_P568792	NM_024221	pyruvate dehydrogenase (lipoamide) beta	2.19	0	1.69	0	2.49	0	3.05	0
*Pdk4	A_51_P350453	NM_013743	pyruvate dehydrogenase kinase, isoenzyme 4	1.08	5.25E-07	0.67	8.05E-06	0.73	2.17E-07	1.31	0.037345
Peci	A_51_P394665	NM_011868	peroxisomal delta3, delta2-enoyl-Coenzyme A isomerase	1.03	0.293581	1.06	0.092592	1.24	8.88E-12	1.43	0
Pecr	A_51_P291749	NM_023523	peroxisomal trans-2-enoyl-CoA reductase	1.10	0.000388	1.19	0.00003	1.06	0.009964	1.32	3.40E-07
Pfkfb2	A_51_P184849	NM_008825	6-phosphofructo-2-kinase/fructose-2,6-biphosphatase 2	1.59	0	0.92	0.031929	0.86	2.12E-08	0.84	0.0002
Pfkfb2	A_51_P335251	NM_008825	6-phosphofructo-2-kinase/fructose-2,6-biphosphatase 2	1.54	1.11E-37	1.20	0.001143	0.96	0.699468	1.25	0.000217
Pfkfb2	A_52_P142912	NM_008825	6-phosphofructo-2-kinase/fructose-2,6-biphosphatase 2	1.60	5.09E-13	0.91	0.124836	0.90	0.1106	0.81	6.64E-06
Pgam1	A_51_P342669	NM_023418	phosphoglycerate mutase 1	1.10	0.000529	1.38	8.25E-25	1.73	0	2.03	0
Pgam2	A_51_P264495	NM_018870	phosphoglycerate mutase 2	1.99	1.48E-11	0.53	1.52E-11	0.58	0.056025	0.69	0.027313
*Pgd	A_52_P84096	BC011329	phosphogluconate dehydrogenase	0.81	5.75E-12	1.21	1.33E-12	1.31	7.47E-39	1.19	2.69E-19
Pgk1	A_51_P182828	NM_008828	phosphoglycerate kinase 1	1.32	9.26E-40	0.97	0.274628	1.20	1.79E-20	1.34	0
Pgk1	A_52_P600398	NM_008828	phosphoglycerate kinase 1	1.31	1.12E-17	1.00	0.956624	1.17	4.03E-11	1.39	0
Pgk2	A_51_P125487	NM_031190	phosphoglycerate kinase 2	1.57	7.19E-06	0.68	0.000755	0.49	2.48E-12	0.77	0.254138
Pgls	A_51_P321794	NM_025396	6-phosphogluconolactonase	1.28	2.95E-24	1.07	0.001801	1.38	6.77E-27	1.66	0
Pgm1	A_51_P427530	NM_025700	phosphoglucomutase 1	0.61	2.09E-43	0.80	2.96E-22	0.75	3.81E-40	0.68	0
Pgm2	A_51_P368074	NM_028132	phosphoglucomutase 2	1.43	1.01E-18	0.87	0.000045	0.93	0.051034	0.98	0.350838
Pgm5	A_51_P451052	NM_175013	phosphoglucomutase 5	0.37	0	0.43	0	0.35	1.62E-29	0.33	1.52E-26
Pgm5	A_52_P860000	NM_175013	phosphoglucomutase 5	0.28	1.90E-20	0.31	3.02E-15	0.39	0.000886	0.32	0.000022
Phka1	A_52_P196825	AK078633	Mus musculus adult male adrenal gland cDNA, RIKEN full-length enriched library, clone:7330411B16	1.49	8.91E-08	0.76	6.55E-11	0.70	0.000159	0.71	8.41E-07
Phka1	A_52_P278311	NM_008832	phosphorylase kinase alpha 1	1.47	5.17E-08	0.52	1.55E-09	0.49	0.000101	0.60	8.37E-07
Phka2	A_51_P239970	AK051899	phosphorylase kinase alpha 2	2.56	0.000086	1.80	0.000915	1.82	0.000945	0.93	0.75141
Phka2	A_51_P368950	AK018208	Mus musculus 12 days embryo eyeball cDNA, RIKEN full-length enriched library, clone:D230017A12	3.01	0	1.38	2.80E-06	1.27	0.000957	1.25	0.00109
Phka2	A_52_P187217	NM_172783	phosphorylase kinase alpha 2	1.93	3.77E-12	1.11	0.264477	1.28	0.006506	1.14	0.156832
Phka2	A_52_P657963	AK045618	phosphorylase kinase alpha 2	2.55	0	1.35	1.54E-06	1.34	5.29E-16	1.26	0.008224
Phka2	A_52_P671916	NM_172783	phosphorylase kinase alpha 2	1.61	1.19E-38	0.93	0.019065	0.92	0.004802	0.94	0.074785
Phkb	A_51_P270725	AK089493	phosphorylase kinase beta	1.42	0	0.99	0.82274	1.02	0.252887	0.86	0.000272
Phkb	A_51_P307624	NM_199446	phosphorylase kinase beta	1.66	6.81E-36	0.99	0.721901	1.02	0.651257	1.02	0.725821
Phkb	A_52_P513347	NM_199446	phosphorylase kinase beta	1.77	1.52E-27	0.94	0.134205	1.01	0.684222	0.96	0.353904
Phkg1	A_51_P280890	NM_011079	phosphorylase kinase gamma 1	1.80	4.52E-13	0.42	4.54E-15	0.16	0	0.47	5.20E-10
Pmvk	A_51_P492408	NM_026784	phosphomevalonate kinase	1.21	2.96E-10	1.39	1.06E-19	1.53	2.40E-31	1.65	5.42E-31
Pmvk	A_51_P492410	NM_026784	phosphomevalonate kinase	1.22	5.67E-12	1.43	1.03E-22	1.61	5.96E-31	1.68	3.29E-31
Ppap2a	A_51_P189104	NM_008903	phosphatidic acid phosphatase 2a	0.58	0	0.95	0.304266	1.04	0.203503	1.09	0.000674
Ppap2a	A_51_P189105	AK077275	phosphatidic acid phosphatase 2a	0.62	1.66E-39	1.06	0.081349	1.15	4.39E-08	1.22	5.26E-13
Ppap2a	A_52_P7937	NM_008903	phosphatidic acid phosphatase 2a	0.52	0	1.09	0.062953	1.18	7.25E-15	1.24	7.56E-12

Ppara	A_51_P348334	NM_011144	peroxisome proliferator activated receptor alpha	2.40	0	1.60	0	2.16	0	3.17	0
Pparg	A_51_P106799	NM_011146	peroxisome proliferator activated receptor gamma	1.31	0	1.12	5.73E-07	1.10	4.63E-06	1.06	0.13698
Ppargc1a	A_51_P279038	NM_008904	peroxisome proliferative activated receptor, gamma, coactivator 1 alpha	2.61	0	0.80	1.24E-10	1.13	0.000617	1.65	0
Ppargc1a	A_52_P5945	AK032149	peroxisome proliferative activated receptor, gamma, coactivator 1 alpha	1.98	0	0.77	6.40E-11	1.04	0.202626	1.34	4.12E-27
Ppargc1b	A_51_P294891	NM_133249	peroxisome proliferative activated receptor, gamma, coactivator 1 beta	1.63	4.13E-17	0.95	0.011936	1.37	6.41E-23	1.83	0
Ppargc1b	A_52_P293222	AK042378	peroxisome proliferative activated receptor, gamma, coactivator 1 beta	2.11	7.29E-34	1.10	0.00366	1.67	2.97E-14	2.33	1.54E-44
Ppargc1b	A_52_P526724	NM_133249	Mus musculus adult male corpora quadrigemina cDNA, RIKEN full-length enriched library, clone:B230304K20	1.41	3.72E-14	0.91	0.000335	1.30	6.50E-14	1.80	0
Ppargc1b	A_52_P883463	AK045690	peroxisome proliferative activated receptor, gamma, coactivator 1 beta	1.84	0	0.83	7.91E-07	1.21	9.04E-13	1.50	3.25E-26
Ppp1cb	A_51_P342716	NM_172707	protein phosphatase 1, catalytic subunit, beta isoform	0.62	0	0.78	3.30E-21	0.79	5.03E-30	0.77	1.00E-18
Ppp1cc	A_52_P519508	NM_013636	protein phosphatase 1, catalytic subunit, gamma isoform	0.85	1.36E-09	0.84	4.87E-17	0.76	7.39E-33	0.72	3.44E-33
Ppt2	A_52_P238816	NM_019441	palmitoyl-protein thioesterase 2	1.43	8.38E-15	1.19	0.000216	1.41	7.11E-40	1.54	1.23E-39
Pygb	A_52_P350664	NM_153781	brain glycogen phosphorylase	1.41	6.87E-11	0.91	0.019785	0.90	0.006818	0.85	0.000025
Pygl	A_51_P452779	NM_133198	liver glycogen phosphorylase	2.46	0	1.23	0.002814	1.42	5.72E-14	1.46	1.33E-16
Pygl	A_52_P320181	AK083075	liver glycogen phosphorylase	4.86	0	2.33	9.14E-24	2.76	0	2.32	2.37E-15
Pygm	A_51_P458973	NM_011224	muscle glycogen phosphorylase	1.51	3.50E-08	0.46	6.02E-13	0.46	0.0064	0.49	5.45E-06
Scd2	A_51_P129464	NM_009128	stearoyl-Coenzyme A desaturase 2	1.00	0.987206	1.48	1.01E-06	1.23	0.001449	0.90	0.079231
Scd4	A_52_P374877	NM_183216	stearoyl-coenzyme A desaturase 4	0.26	1.95E-17	0.36	7.46E-09	0.28	9.81E-45	0.90	0.515866
*Sdha	A_51_P410823	NM_023281	succinate dehydrogenase complex, subunit A, flavoprotein (Fp)	1.86	1.47E-43	1.02	0.568497	1.42	6.81E-32	1.58	0
Sdha	A_52_P109503	NM_023281	succinate dehydrogenase complex, subunit A, flavoprotein (Fp)	1.65	8.95E-43	1.23	3.71E-13	1.66	0	1.84	0
*Sdhb	A_51_P234853	NM_023374	succinate dehydrogenase complex, subunit B, iron sulfur (Ip)	1.76	0	1.20	8.27E-09	1.71	0	1.96	0
Sdhc	A_51_P106373	NM_025321	succinate dehydrogenase complex, subunit C, integral membrane protein	1.32	1.27E-21	1.10	4.33E-06	1.35	6.47E-37	1.48	1.90E-35
Sdhc	A_52_P875481	AK031343	Mus musculus 13 days embryo male testis cDNA, RIKEN full-length enriched library, clone:6030410A06	1.83	0.000105	1.01	0.957342	0.78	0.236953	1.16	0.361315
Sdhd	A_51_P260871	NM_025848	succinate dehydrogenase complex, subunit D, integral membrane protein	1.29	1.13E-10	1.11	0.001444	1.57	3.53E-27	1.90	0
Slc25a1	A_51_P350922	NM_153150	solute carrier family 25 (mitochondrial carrier, citrate transporter), member 1	2.20	0	2.02	0	2.79	0	2.92	0
*Slc27a1	A_51_P117477	NM_011977	solute carrier family 27 (fatty acid transporter), member 1	0.75	0	99.00	0.256111	1.39	4.66E-33	1.56	0
Slc27a1	A_52_P199905	CB588406	AGENCOURT_12600125 NIH_MGC_136 Mus musculus cDNA clone IMAGE:30290747 5', mRNA sequence.	0.84	3.58E-13	1.10	0.063034	1.60	1.01E-23	1.95	0
Slc27a2	A_52_P154971	NM_011978	solute carrier family 27 (fatty acid transporter), member 2	0.66	0.08825	0.95	0.352499	1.05	0.478478	2.65	3.86E-11
Slc27a4	A_51_P408609	NM_011989	solute carrier family 27 (fatty acid transporter), member 4	0.96	0.385821	1.25	0.000383	1.31	2.76E-06	1.49	7.09E-19
Slc27a4	A_52_P61854	NM_011989	solute carrier family 27 (fatty acid transporter), member 4	0.83	0.000053	1.20	0.000044	1.43	1.85E-21	1.67	1.00E-34

Slc27a5	A_51_P289742	NM_009512	solute carrier family 27 (fatty acid transporter), member 5	0.40	3.57E-13	0.78	0.028052	0.79	0.004282	1.53	0.005496
Slc2a2	A_51_P185693	NM_031197	solute carrier family 2 (facilitated glucose transporter), member 2	0.30	9.17E-09	0.96	0.735134	0.65	0.020819	0.98	0.910207
*Slc2a4	A_51_P217498	NM_009204	solute carrier family 2 (facilitated glucose transporter), member 4	2.14	0	1.06	0.125701	1.45	8.00E-26	1.58	0
Sqle	A_51_P450487	NM_009270	squalene epoxidase	0.51	6.70E-42	0.87	0.015337	0.75	3.28E-08	0.73	4.16E-14
Sucla2	A_51_P111554	NM_011506	succinate-Coenzyme A ligase, ADP-forming, beta subunit	1.15	5.07E-06	1.32	4.54E-24	1.88	0	2.07	0
*Sucla2	A_52_P533792	NM_011506	succinate-Coenzyme A ligase, ADP-forming, beta subunit	1.50	2.87E-19	1.46	2.92E-38	2.33	1.15E-42	2.89	0
Sucla2	A_52_P762514	AK016060	succinate-Coenzyme A ligase, ADP-forming, beta subunit	0.76	0.035691	0.73	0.121576	0.70	0.000017	0.80	0.029037
Suclg1	A_51_P491227	NM_019879	succinate-CoA ligase, GDP-forming, alpha subunit	1.21	6.74E-18	1.13	1.75E-11	1.58	0	1.98	0
*Taldo1	A_51_P401958	NM_011528	transaldolase 1	1.60	3.91E-38	1.33	6.41E-12	1.61	0	1.62	0
*Tkt	A_51_P394515	NM_009388	transketolase	4.05	0	1.51	6.24E-13	1.86	0	2.04	0
Tkt	A_52_P624447	NM_009388	transketolase	2.70	1.16E-43	1.66	2.07E-12	1.99	0	1.96	0
Tkt	A_52_P631197	AK050793	transketolase	4.56	1.32E-28	2.61	1.49E-21	2.13	6.89E-12	2.11	4.33E-08
Tpi1	A_51_P433824	NM_009415	triosephosphate isomerase 1	1.00	0.899227	1.23	8.20E-16	1.34	4.50E-28	1.49	0
Tpi1	A_52_P116102	AK053780	Mus musculus 0 day neonate eyeball cDNA, RIKEN library, clone:E130308D14 product:triosephosphate isomerase	1.40	3.38E-11	1.17	0.000215	1.01	0.784684	1.29	2.62E-11
Ucp1	A_51_P426353	NM_009463	uncoupling protein 1 (mitochondrial, proton carrier)	0.42	0.000062	3.13	0	6.79	0	10.46	0
Ucp2	A_51_P297105	NM_011671	uncoupling protein 2 (mitochondrial, proton carrier)	0.37	0	0.96	0.124993	0.97	0.202268	0.93	0.020408
Ucp2	A_52_P499675	NM_011671	uncoupling protein 2 (mitochondrial, proton carrier)	0.64	0.003502	1.54	6.23E-12	1.82	1.02E-08	1.40	0.000568
Ucp2	A_52_P90265	NM_011671	uncoupling protein 2 (mitochondrial, proton carrier)	0.25	0	0.99	0.904595	1.02	0.48046	0.90	0.18525
Ugp2	A_51_P475580	NM_139297	UDP-glucose pyrophosphorylase 2	1.24	5.26E-25	0.79	7.29E-33	0.75	0	0.81	2.22E-13
Uqcrb	A_51_P472671	NM_026219	ubiquinol-cytochrome c reductase binding protein	1.81	0	1.40	1.77E-34	1.82	0	2.14	0
*Uqcrc1	A_51_P109835	NM_025407	ubiquinol-cytochrome c reductase core protein 1	1.41	1.47E-19	0.99	0.49	1.35	2.17E-31	1.54	1.43E-32
*Uqcrc2	A_51_P128648	NM_025899	ubiquinol cytochrome c reductase core protein 2	1.77	1.98E-36	1.25	0	1.79	0	2.1	0
*Uqcrfs1	A_51_P361951	NM_025710	ubiquinol-cytochrome c reductase, Rieske iron-sulfur polypeptide 1	1.70	1.02E-39	1.28	4.81E-26	1.89	0	2.23	0
Uqcrh	A_51_P431852	NM_025641	ubiquinol-cytochrome c reductase hinge protein	1.65	7.97E-10	1.28	1.05E-07	1.57	6.97E-12	1.83	1.44E-16
Uqcrh	A_52_P541875	NM_025641	ubiquinol-cytochrome c reductase hinge protein	1.89	7.02E-22	1.43	4.96E-20	1.81	6.82E-33	1.97	0
*Uqcrq	A_52_P370484	NM_025352	ubiquinol-cytochrome c reductase, complex III subunit VII	1.35	3.90E-07	1.18	3.94E-08	1.60	1.50E-14	1.80	4.07E-18
Acox3	A_52_P745	NM_030721	acyl-Coenzyme A oxidase 3, pristanoyl	1.07	0.012963	1.01	0.591271	0.99	0.691992	1.08	0.006656
Acsl5	A_52_P593037	NM_027976	acyl-CoA synthetase long-chain family member 5	1.02	0.355204	0.97	0.454445	0.81	2.28E-19	1.11	0.077691
Acsl6	A_52_P352187	NM_001033599	acyl-CoA synthetase long-chain family member 6	0.89	0.347724	0.78	0.063197	0.87	0.230061	1.00	0.989916
Adcy1	A_51_P409900	NM_009622	adenylate cyclase 1	1.20	0.182849	0.84	0.152138	1.03	0.757305	0.92	0.402893
Adcy1	A_52_P240164	NM_009622	adenylate cyclase 1	1.23	0.001905	0.99	0.773788	1.26	2.54E-08	1.27	6.90E-08
Adcy1	A_52_P240170	BC050125	adenylate cyclase 1	1.29	3.33E-22	1.10	0.010514	1.14	0.000899	1.13	0.004864
Adcy4	A_51_P229911	NM_080435	adenylate cyclase 4	1.05	0.028168	1.10	0.000121	1.00	0.862828	1.12	7.65E-07

Adcy6	A_51_P243134	NM_007405	adenylate cyclase 6	0.87	0.000478	1.03	0.50625	1.15	0.000063	1.24	4.61E-10
Adcy8	A_51_P303061	NM_009623	adenylate cyclase 8	0.95	0.684212	0.77	0.243998	0.91	0.536823	2.65	0.00169
Adcy9	A_51_P501538	NM_009624	adenylate cyclase 9	1.19	2.12E-09	0.92	0.038269	0.89	0.000108	0.90	0.005309
Adh1	A_52_P835985	AK082149	alcohol dehydrogenase 1 (class I)	0.99	0.876288	0.77	0.381341	1.07	0.669994	0.95	0.734211
Adh5	A_51_P404275	NM_007410	alcohol dehydrogenase 5 (class III), chi polypeptide	0.92	0.000204	0.96	0.248971	0.97	0.043223	0.96	0.086322
Adh6a	A_51_P299608	AK028114	alcohol dehydrogenase 6A (class V)	1.00	1	0.98	0.820767	1.00	1	0.95	0.678137
Adh6-ps1	A_51_P120027	AK004863	alcohol dehydrogenase 6 (class V), pseudogene 1	1.00	1	1.10	0.595158	1.00	1	1.00	1
Aldh2	A_52_P116134	NM_009656	aldehyde dehydrogenase 2, mitochondrial	1.14	0.000701	0.87	0.000112	0.79	9.73E-08	0.84	0.000233
Aldh2	A_52_P13109	AK163452	aldehyde dehydrogenase 2, mitochondrial	0.85	0.000098	1.10	0.00041	1.07	0.010617	0.96	0.3178
Aldh7a1	A_51_P470414	NM_138600	aldehyde dehydrogenase family 7, member A1	1.36	0.02373	1.23	0.009783	1.28	0.012894	1.52	0.034321
Aldh9a1	A_52_P265877	NM_019993	aldehyde dehydrogenase 9, subfamily A1	1.04	0.418261	0.86	9.77E-06	1.02	0.478382	1.01	0.782009
Aldoa	A_51_P216905	NM_007438	aldolase 1, A isoform	0.86	0.058289	0.93	0.131511	1.00	0.975145	1.02	0.794427
Aldoa	A_52_P98387	NM_007438	aldolase 1, A isoform	0.88	0.101458	0.92	0.032198	1.02	0.746366	1.03	0.545286
Apoa1	A_51_P408082	NM_009692	apolipoprotein A-I	0.83	0.019394	1.02	0.776411	1.17	0.005719	1.72	0.00998
Apob	A_51_P380650	XM_137955	Mus musculus apolipoprotein B (Apob), mRNA.	1.39	0.231049	0.99	0.95816	1.20	0.449147	0.94	0.743505
Apob	A_51_P436690	BC028880	apolipoprotein B	1.00	1	0.81	0.408211	0.96	0.656631	1.29	0.211563
Atp5b	A_51_P207636	NM_016774	ATP synthase, H+ transporting mitochondrial F1 complex, beta subunit	1.14	0.000011	1.21	0.000014	1.53	0	1.65	5.10E-27
Atp6ap1	A_51_P447988	NM_018794	ATPase, H+ transporting, lysosomal accessory protein 1	0.93	0.018787	0.96	0.188751	0.95	0.031868	0.86	1.23E-06
Atp6v1g1	A_51_P140171	NM_024173	ATPase, H+ transporting, lysosomal V1 subunit G1	1.21	1.51E-11	0.99	0.678773	1.00	0.881593	1.03	0.211064
Atp6v1g1	A_52_P349738	NM_024173	ATPase, H+ transporting, lysosomal V1 subunit G1	0.96	0.06312	0.89	4.54E-06	0.91	1.20E-12	0.90	0.000475
Atp6v1h	A_51_P105604	NM_133826	ATPase, H+ transporting, lysosomal V1 subunit H	0.95	0.002579	1.13	0.000013	1.25	2.42E-19	1.30	2.80E-18
Atp6v1h	A_52_P112888	NM_133826	ATPase, H+ transporting, lysosomal V1 subunit H	0.87	1.95E-06	1.03	0.474404	1.26	2.45E-17	1.24	9.44E-13
Atp7a	A_51_P312497	NM_009726	ATPase, Cu++ transporting, alpha polypeptide	0.84	2.37E-26	0.94	0.025972	0.82	1.50E-14	0.84	1.29E-09
Atp7a	A_51_P473179	AK033254	ATPase, Cu++ transporting, alpha polypeptide	1.24	3.32E-13	1.09	0.02678	0.88	0.000192	0.90	0.07406
Bcnp1	A_52_P1053160	AK158308	BCNP1 homolog	1.14	0.023369	0.95	0.137616	0.95	0.312075	1.11	0.005399
Coq2	A_51_P447752	NM_027978	coenzyme Q2 homolog, prenyltransferase (yeast)	1.24	3.56E-14	1.07	0.005512	1.13	0.000575	1.19	1.37E-15
Cpt1c	A_51_P177562	NM_153679	carnitine palmitoyltransferase 1c	1.09	0.428115	0.96	0.669906	0.87	0.101224	0.80	0.04668
Cpt1c	A_52_P269942	NM_153679	carnitine palmitoyltransferase 1c	1.12	0.007636	1.00	0.977338	0.88	1.20E-06	0.81	1.06E-10
Crot	A_51_P489153	NM_023733	carnitine O-octanoyltransferase	1.21	5.39E-32	1.00	0.901364	1.02	0.394437	1.02	0.411174
Crot	A_52_P656336	NM_023733	carnitine O-octanoyltransferase	1.02	0.714633	1.05	0.106651	1.26	8.64E-16	1.16	0.002621
Cyp19a1	A_51_P474551	NM_007810	cytochrome P450, family 19, subfamily a, polypeptide 1	1.50	0.043947	1.12	0.457929	0.99	0.923294	1.11	0.495814
Cyp19a1	A_52_P591961	NM_007810	cytochrome P450, family 19, subfamily a, polypeptide 1	1.00	1	1.00	1	1.00	1	1.00	1
Cyp1a2	A_51_P450140	NM_009993	cytochrome P450, family 1, subfamily a, polypeptide 2	1.24	0.31197	0.97	0.874319	0.86	0.227157	0.82	0.195549
Cyp1a2	A_52_P595871	NM_009993	cytochrome P450, family 1, subfamily a, polypeptide 2	1.00	1	0.79	0.19904	1.00	0.924017	1.02	0.814531

Cyp1b1	A_51_P255456	NM_009994	cytochrome P450, family 1, subfamily b, polypeptide 1	0.86	0.040205	0.92	0.218161	0.83	0.000799	0.80	0.000028
Cyp1b1	A_52_P297822	AK016699	cytochrome P450, family 1, subfamily b, polypeptide 1	0.59	0.001119	0.90	0.194968	1.01	0.955016	0.85	0.269662
Cyp2b19	A_52_P421626	AK029227	cytochrome P450, family 2, subfamily b, polypeptide 19	1.00	1	1.30	0.331601	1.00	1	1.97	0.071727
Cyp2c37	A_51_P498882	NM_010001	cytochrome P450, family 2. subfamily c, polypeptide 37	1.02	0.709079	0.93	0.048272	0.99	0.740926	1.05	0.514247
Cyp2c37	A_52_P625249	NM_010001	cytochrome P450, family 2. subfamily c, polypeptide 37	1.07	0.095917	0.95	0.27194	1.07	0.00042	1.15	0.00029
Cyp2c38	A_51_P342206	NM_010002	cytochrome P450, family 2, subfamily c, polypeptide 38	1.00	1	1.00	1	1.00	1	1.00	1
Cyp2c38	A_52_P468564	NM_010002	cytochrome P450, family 2, subfamily c, polypeptide 38	1.00	1	1.00	1	1.00	1	1.00	1
Cyp2g1	A_51_P137452	NM_013809	cytochrome P450, family 2, subfamily g, polypeptide 1	1.04	0.850515	1.05	0.644875	1.24	0.122211	1.09	0.501205
Cyp2g1	A_52_P481346	NM_013809	cytochrome P450, family 2, subfamily g, polypeptide 1	1.00	1	1.00	1	1.00	1	0.97	0.721233
Cyp2s1	A_52_P456837	AK054324	cytochrome P450, family 2, subfamily s, polypeptide 1	1.00	1	1.00	1	1.00	1	1.00	1
Cyp3a25	A_52_P380314	NM_019792	cytochrome P450, family 3, subfamily a, polypeptide 25	1.02	0.780025	0.67	0.158943	1.03	0.881445	1.12	0.536849
Cyp3a44	A_52_P366803	NM_177380	cytochrome P450, family 3, subfamily a, polypeptide 44	1.00	1	1.00	1	1.00	1	1.00	1
Cyp4a10	A_52_P6588	NM_010011	cytochrome P450, family 4, subfamily a, polypeptide 10	0.81	0.217329	1.01	0.94825	1.38	0.011993	1.25	0.266291
Cyp51	A_52_P778750	AK035338	Mus musculus adult male urinary bladder cDNA, RIKEN full-length enriched library, clone:9530019F09	1.00	1	1.00	1	1.00	1	1.00	1
Dgat2	A_52_P359739	NM_026384	diacylglycerol O-acyltransferase 2	1.16	0.029512	1.26	0.005157	1.24	0.000893	1.16	0.02989
Dgat2	A_52_P592909	NM_026384	diacylglycerol O-acyltransferase 2	0.78	0.009147	1.16	0.121883	1.11	0.304754	0.92	0.448932
Eno1	A_52_P349939	NM_023119	enolase 1, alpha non-neuron	0.95	0.550728	1.00	0.981093	1.11	0.24385	1.10	0.259116
Fabp4	A_51_P336830	NM_024406	fatty acid binding protein 4, adipocyte	0.82	0.214911	0.97	0.808568	1.32	0.065254	1.04	0.833539
Fabp4	A_51_P336833	NM_024406	fatty acid binding protein 4, adipocyte	0.79	0.017142	1.00	0.943548	1.12	0.175331	1.06	0.490436
Fabp4	A_52_P564544	NM_024406	fatty acid binding protein 4, adipocyte	0.82	1.01E-08	1.00	0.949544	1.09	0.040001	1.19	4.98E-10
Fntb	A_52_P235241	NM_145927	farnesyltransferase, CAAX box, beta	0.87	1.23E-07	1.23	2.03E-10	1.29	1.26E-15	1.11	0.000075
G6pc	A_51_P462385	NM_008061	glucose-6-phosphatase, catalytic	1.06	0.088893	0.90	1.59E-10	0.87	0.000101	1.00	0.958128
Gck	A_52_P66580	BC011139	glucokinase	1.14	0.000029	1.01	0.815681	1.08	0.01196	1.08	0.017214
Gckr	A_51_P198694	NM_144909	glucokinase regulatory protein	1.28	0.0001	0.96	0.479682	0.87	0.023119	1.18	0.113194
Ggps1	A_51_P179604	NM_010282	PREDICTED: Mus musculus geranylgeranyl diphosphate synthase 1, transcript variant 4 (Ggps1), mRNA.	0.87	0.000012	1.09	0.030017	1.12	0.000841	1.06	0.111861
Ggps1	A_51_P469902	XM_977926	geranylgeranyl diphosphate synthase 1	1.21	7.18E-32	1.03	0.328169	1.05	0.003303	1.06	0.103975
Gpi1	A_51_P426886	NM_008155	glucose phosphate isomerase 1	0.94	0.014148	0.94	0.001549	1.04	0.009042	1.10	1.02E-09
Gpi1	A_52_P347412	L09104	glucose phosphate isomerase 1	1.18	2.34E-08	0.99	0.800149	1.05	0.419632	1.01	0.825067
Gyk	A_52_P379205	AK086200	glycerol kinase	1.00	1	1.00	1	1.00	1	1.00	1
Hk1	A_51_P249024	NM_010438	hexokinase 1	1.10	0.0013	0.97	0.117679	1.04	0.225444	1.02	0.386539
Hmgcl	A_51_P308961	NM_008254	3-hydroxy-3-methylglutaryl-Coenzyme A lyase	1.27	2.34E-11	0.94	0.019789	0.99	0.859586	1.05	0.066699
Hmgcll1	A_52_P197722	NM_173731	3-hydroxymethyl-3-methylglutaryl-Coenzyme A lyase-like 1	0.93	0.597122	0.95	0.589395	0.65	0.001177	0.66	0.014421
Idh2	A_51_P331507	NM_173011	isocitrate dehydrogenase 2 (NADP+), mitochondrial	1.09	0.002825	0.92	0.016238	1.03	0.499745	1.01	0.574054
Idi1	A_51_P329711	NM_177960	isopentenyl-diphosphate delta isomerase	0.77	1.17E-09	1.26	0.064896	0.98	0.666972	2.07	0.004069

Ldha	A_51_P364140	NM_010699	lactate dehydrogenase A	1.24	0.050787	1.00	0.969275	1.19	0.071834	0.93	0.4733
Lipe	A_51_P435363	NM_010719	lipase, hormone sensitive	1.24	0.000579	0.89	0.032418	0.98	0.77148	1.07	0.269167
Lipe	A_51_P435366	NM_010719	lipase, hormone sensitive	1.29	1.52E-12	0.89	0.010097	1.04	0.365704	1.05	0.165093
Lpl	A_51_P259296	NM_008509	lipoprotein lipase	0.86	0.219408	1.10	0.133678	1.04	0.723662	0.89	0.230061
Lpl	A_52_P257812	NM_008509	lipoprotein lipase	0.79	0.000038	1.00	0.978509	0.97	0.484361	0.96	0.502204
Mgll	A_51_P352549	AK079089	Mus musculus adult male diencephalon cDNA, RIKEN full-length enriched library, clone:9330168D17	1.04	0.799752	1.59	0.029078	1.29	0.217801	1.34	0.238741
Mut	A_52_P362959	AK051851	methylmalonyl-Coenzyme A mutase	1.00	1	1.00	1	1.00	1	1.00	1
Mvk	A_51_P169527	NM_023556	mevalonate kinase	0.98	0.716153	1.14	0.002949	1.03	0.364409	1.14	0.014182
Mvk	A_52_P569067	NM_023556	mevalonate kinase	0.89	4.62E-08	1.10	0.001679	0.97	0.391819	1.11	0.005468
Oxct2a	A_51_P125062	NM_022033	3-oxoacid CoA transferase 2A	1.11	0.128763	1.02	0.841826	1.04	0.659152	1.16	0.138178
Pck2	A_52_P21659	NM_028994	phosphoenolpyruvate carboxykinase 2 (mitochondrial)	0.95	0.106455	0.93	0.000957	0.81	1.76E-15	0.83	4.53E-15
Pdha2	A_51_P519857	NM_008811	pyruvate dehydrogenase E1 alpha 2	1.00	1	1.00	1	1.00	1	1.04	0.673396
Pfkfb2	A_52_P31125	BC018418	6-phosphofructo-2-kinase/fructose-2,6-biphosphatase 2	1.17	0.15459	1.27	1.75E-07	1.19	0.042557	1.07	0.387925
Pfkl	A_51_P310896	NM_008826	phosphofructokinase, liver, B-type	0.95	0.456149	0.96	0.512786	0.98	0.737186	1.03	0.578316
Pgd	A_51_P391996	BC011329	phosphogluconate dehydrogenase	0.81	0.000035	1.23	0.000035	1.29	2.07E-06	1.30	6.00E-07
Pgm3	A_51_P208987	NM_028352	phosphoglucomutase 3	1.22	0.000028	0.99	0.705489	1.12	0.021804	1.11	0.033879
Pgm3	A_52_P341449	NM_028352	phosphoglucomutase 3	0.88	0.000056	0.87	0.00035	0.94	0.186558	0.90	0.001835
Pgm5	A_52_P229924	NM_175013	phosphoglucomutase 5	0.83	0.000092	0.82	0.005585	0.89	0.007757	0.86	0.000302
Phkb	A_52_P342907	BC051503	phosphorylase kinase beta	1.00	1	1.01	0.908122	1.10	0.56797	0.95	0.71096
Phkg2	A_51_P235909	NM_026888	phosphorylase kinase, gamma 2 (testis)	1.09	0.01052	0.91	0.008279	0.92	0.031081	0.95	0.134684
Phkg2	A_52_P19446	AK089062	phosphorylase kinase, gamma 2 (testis)	1.07	0.260054	0.93	0.277702	0.87	0.022259	0.94	0.415741
Pklr	A_51_P176042	NM_013631	pyruvate kinase liver and red blood cell	0.88	0.0008	0.86	0.00399	0.85	0.000051	0.92	0.276819
Pkm2	A_51_P428913	D38379	pyruvate kinase, muscle	1.28	5.22E-08	0.81	3.81E-10	0.82	6.03E-06	0.85	1.66E-09
Pkm2	A_52_P150212	NM_011099	pyruvate kinase, muscle	1.22	9.00E-06	0.87	0.003785	0.86	0.002962	0.88	0.004118
Pkm2	A_52_P328249	AK083076	pyruvate kinase, muscle	1.01	0.819911	1.11	0.007559	1.14	0.000067	1.19	0.000051
Ppard	A_51_P271556	NM_011145	peroxisome proliferator activator receptor delta	0.87	0.049211	1.05	0.529051	1.07	0.180561	1.17	0.031455
Ppp1ca	A_51_P509389	NM_031868	protein phosphatase 1, catalytic subunit, alpha isoform	1.01	0.704571	1.01	0.68392	1.08	0.000249	1.11	2.42E-07
Ppp1cc	A_52_P540434	NM_013636	protein phosphatase 1, catalytic subunit, gamma isoform	0.85	1.18E-19	0.90	2.66E-08	0.84	4.49E-14	0.83	2.44E-30
Pygb	A_51_P485810	NM_153781	brain glycogen phosphorylase	1.25	0.010486	0.96	0.679262	0.88	0.18676	0.91	0.345052
Scd1	A_52_P682382	NM_009127	stearoyl-Coenzyme A desaturase 1	0.98	0.870532	0.82	0.041844	0.89	0.340263	0.65	0.001507
Scd3	A_51_P446045	NM_024450	stearoyl-coenzyme A desaturase 3	0.95	0.143426	1.00	0.943486	1.14	1.08E-07	1.08	0.085823
Scd4	A_52_P603237	BC038322	stearoyl-coenzyme A desaturase 4	0.91	0.501782	0.61	0.060544	0.95	0.626895	0.99	0.96248
Slc27a2	A_51_P484551	NM_011978	solute carrier family 27 (fatty acid transporter), member 2	1.53	0.31249	0.89	0.600611	1.00	1	1.76	0.001391
Slc27a2	A_52_P1083677	AK038847	solute carrier family 27 (fatty acid transporter), member 2	1.00	1	1.00	1	1.00	1	1.00	1

	Slc27a3	A_51_P133252	NM_011988	solute carrier family 27 (fatty acid transporter), member 3	0.92	0.000292	1.17	5.27E-10	1.02	0.386599	0.93	0.028266
	Slc27a6	A_51_P211616	AK028699	solute carrier family 27 (fatty acid transporter), member 6	0.75	0.377984	1.07	0.739109	1.06	0.743573	1.11	0.581475
	Slc2a2	A_52_P409988	NM_031197	solute carrier family 2 (facilitated glucose transporter), member 2	1.18	4.74E-09	1.06	0.094112	1.17	0.000185	1.25	1.30E-07
	Suclg2	A_51_P478672	NM_011507	succinate-Coenzyme A ligase, GDP-forming, beta subunit	1.30	5.77E-08	0.99	0.742431	1.05	0.241363	1.09	0.053324
	Suclg2	A_52_P58181	NM_011507	succinate-Coenzyme A ligase, GDP-forming, beta subunit	1.18	8.07E-13	0.89	1.03E-09	0.99	0.77572	1.01	0.51572
	Ugp2	A_52_P42242	NM_139297	UDP-glucose pyrophosphorylase 2	1.24	2.28E-15	0.80	2.67E-22	0.80	8.70E-10	0.80	5.89E-44
**Soleus**	Acaa1a	A_52_P155990	NM_130864	acetyl-Coenzyme A acyltransferase 1A	1.32	1.17E-11	1.14	0.028427	1.22	0.0002	1.20	0.000123
	Acaa2	A_51_P125260	NM_177470	acetyl-Coenzyme A acyltransferase 2 (mitochondrial 3-oxoacyl-Coenzyme A thiolase)	1.37	3.94E-36	1.14	0.010651	1.19	1.59E-09	1.09	0.000115
	*Acaca	A_51_P439426	NM_133360	acetyl-Coenzyme A carboxylase alpha	0.57	2.15E-08	0.75	0.004111	0.60	1.07E-21	0.84	0.001293
	Acaca	A_52_P132591	AF374167	acetyl-Coenzyme A carboxylase alpha	0.80	0.150741	0.73	0.000566	0.88	0.12172	0.70	0.002174
	Acaca	A_52_P318040	AK132628	acetyl-Coenzyme A carboxylase alpha	0.56	5.10E-26	0.76	3.88E-10	0.80	9.13E-09	0.90	0.007875
	Acaca	A_52_P595124	NM_133360	acetyl-Coenzyme A carboxylase alpha	0.74	3.08E-14	0.81	0.000278	0.98	0.798377	0.82	1.03E-10
	Acads	A_52_P367745	NM_007383	acyl-Coenzyme A dehydrogenase, short chain	1.62	1.55E-10	1.26	0.000114	1.59	7.86E-16	1.20	0.009755
	Acadsb	A_51_P435068	NM_025826	acyl-Coenzyme A dehydrogenase, short/branched chain	1.13	0.000142	0.81	3.79E-07	0.78	1.70E-13	0.77	2.11E-39
	Acat2	A_51_P139748	NM_009338	acetyl-Coenzyme A acetyltransferase 2	1.06	0.043541	1.39	2.92E-13	1.30	1.10E-16	1.39	1.19E-11
	Aco1	A_52_P299115	NM_007386	aconitase 1	1.59	5.22E-15	1.34	1.01E-06	1.14	0.062715	1.42	9.39E-07
	Aco2	A_52_P200359	NM_080633	aconitase 2, mitochondrial	1.37	0	1.23	9.73E-12	1.19	1.83E-10	1.22	2.64E-08
	Aco2	A_52_P899263	AK029122	Mus musculus 10 days neonate skin cDNA, RIKEN full-length enriched library, clone:4732494K09	2.49	1.19E-08	1.65	0.001998	0.89	0.242534	1.14	0.163357
	Acox1	A_51_P366704	NM_015729	acyl-Coenzyme A oxidase 1, palmitoyl	0.68	0	0.79	2.67E-15	0.79	6.65E-19	0.86	8.37E-23
	Acox1	A_52_P615096	NM_015729	acyl-Coenzyme A oxidase 1, palmitoyl	0.53	6.75E-09	0.66	0.000472	0.78	0.022822	0.74	0.004115
	Acox2	A_51_P206708	NM_053115	acyl-Coenzyme A oxidase 2, branched chain	0.32	0	0.79	0.010544	0.73	1.88E-22	0.85	0.000097
	Acsl1	A_52_P597618	NM_007981	acyl-CoA synthetase long-chain family member 1	0.79	5.11E-07	0.76	1.41E-11	0.81	9.22E-09	0.90	0.000112
	Acsl6	A_52_P204459	AK147421	acyl-CoA synthetase long-chain family member 6	1.03	0.497727	0.78	0.00034	0.76	5.34E-07	0.81	0.000027
	Acsl6	A_52_P352187	NM_001033599	acyl-CoA synthetase long-chain family member 6	1.12	0.002862	1.31	0.004379	1.32	2.41E-08	1.25	0.000024
	Adcy4	A_51_P229911	NM_080435	adenylate cyclase 4	1.43	1.45E-09	1.22	8.53E-10	1.29	2.03E-10	1.42	1.39E-16
	Adcy5	A_51_P199041	NM_001012765	adenylate cyclase 5	0.30	0	0.67	0.000117	0.66	0.000034	1.36	0.000068
	Adcy5	A_52_P449208	NM_001012765	adenylate cyclase 5	0.23	0	0.55	1.28E-07	0.66	9.41E-09	1.57	3.94E-12
	Agpat2	A_51_P238563	NM_026212	1-acylglycerol-3-phosphate O-acyltransferase 2 (lysophosphatidic acid acyltransferase, beta)	0.90	0.072554	1.03	0.746427	1.01	0.83659	1.53	4.39E-14
	Agpat2	A_51_P238565	NM_026212	1-acylglycerol-3-phosphate O-acyltransferase 2 (lysophosphatidic acid acyltransferase, beta)	0.84	0.000521	1.01	0.902578	1.00	0.970781	1.51	3.48E-18
	Aldh1a1	A_51_P334942	NM_013467	aldehyde dehydrogenase family 1, subfamily A1	0.73	1.62E-09	0.96	0.399181	0.95	0.236953	0.94	0.090587
	Aldh1a2	A_52_P58145	NM_009022	aldehyde dehydrogenase family 1, subfamily A2	1.91	0	1.43	0.000013	1.25	1.17E-08	1.32	4.81E-20
	Aldh1a7	A_51_P383399	NM_011921	aldehyde dehydrogenase family 1, subfamily A7	0.32	0	0.63	0.000012	0.73	3.13E-10	1.39	9.64E-18

Aldh1b1	A_51_P510418	NM_028270	aldehyde dehydrogenase 1 family, member B1	1.18	0.000068	1.45	0.000602	1.64	0	1.44	2.31E-14
Aldh2	A_52_P116134	NM_009656	aldehyde dehydrogenase 2, mitochondrial	1.33	0.000015	1.13	0.119625	1.17	0.054956	1.18	0.017117
Aldh2	A_52_P13109	AK163452	aldehyde dehydrogenase 2, mitochondrial	1.54	9.23E-20	1.08	0.060114	1.17	0.018255	1.23	3.55E-07
Aldh5a1	A_51_P491504	NM_172532	aldhehyde dehydrogenase family 5, subfamily A1	1.47	0	1.02	0.543144	1.04	0.14417	1.10	7.47E-08
Aldh5a1	A_52_P30273	AK051603	aldhehyde dehydrogenase family 5, subfamily A1	3.04	3.67E-09	1.81	0.000602	1.72	0.022997	1.00	0.979372
Aldh9a1	A_51_P106211	NM_019993	aldehyde dehydrogenase 9, subfamily A1	1.09	0.033176	1.17	0.002505	1.23	6.19E-09	1.31	3.05E-08
Aldoa	A_51_P216905	NM_007438	aldolase 1, A isoform	1.56	9.30E-10	0.90	0.191273	0.86	0.007786	1.01	0.940985
*Aldoa	A_52_P98387	NM_007438	aldolase 1, A isoform	1.58	3.62E-06	1.23	0.790626	0.98	0.636253	0.99	0.380557
Apoa4	A_51_P327496	NM_007468	apolipoprotein A-IV	1.48	0.000172	1.48	3.75E-06	1.13	0.005237	1.20	0.005083
Apoc1	A_51_P164504	NM_007469	apolipoprotein C-I	0.83	1.40E-06	0.99	0.913887	0.92	0.16719	1.36	2.26E-18
Apoc2	A_51_P334979	NM_009695	apolipoprotein C-II	0.72	1.94E-12	0.90	0.020195	0.89	0.000016	1.07	0.027594
Atp5e	A_51_P362054	NM_025983	ATP synthase, H+ transporting, mitochondrial F1 complex, epsilon subunit	1.48	3.79E-39	1.04	0.1607	1.06	0.04033	1.01	0.735085
Atp5k	A_51_P301289	NM_007507	ATP synthase, H+ transporting, mitochondrial F1F0 complex, subunit e	1.67	3.35E-30	1.30	1.87E-09	1.30	1.07E-11	1.35	4.00E-19
Atp6v0a1	A_52_P399998	NM_016920	ATPase, H+ transporting, lysosomal V0 subunit A1	0.51	9.39E-23	0.75	4.78E-10	0.77	1.61E-07	0.97	0.659058
Atp6v0a1	A_52_P515336	AK045877	ATPase, H+ transporting, lysosomal V0 subunit A1	0.77	1.35E-07	0.89	0.080552	1.01	0.905102	1.00	0.981941
Atp6v1c2	A_51_P518600	NM_133699	ATPase, H+ transporting, lysosomal V1 subunit C2	0.85	0.569534	1.39	0.050871	0.88	0.419675	1.86	0.00001
Atp6v1g1	A_52_P349738	NM_024173	ATPase, H+ transporting, lysosomal V1 subunit G1	0.76	1.58E-36	0.92	0.003571	0.91	8.52E-07	0.88	4.56E-12
Atp6v1h	A_51_P105604	NM_133826	ATPase, H+ transporting, lysosomal V1 subunit H	0.36	0	0.63	1.02E-23	0.66	6.21E-36	0.68	2.22E-08
Atp6v1h	A_52_P112888	NM_133826	ATPase, H+ transporting, lysosomal V1 subunit H	0.35	0	0.62	0	0.63	1.29E-35	0.68	3.34E-14
Atp6v1h	A_52_P403420	AK081492	ATPase, H+ transporting, lysosomal V1 subunit H	0.45	5.26E-36	0.46	1.01E-35	0.46	5.40E-31	0.41	2.37E-43
Bdh2	A_51_P470935	NM_027208	3-hydroxybutyrate dehydrogenase, type 2	1.39	3.07E-08	1.26	9.11E-12	1.19	0.000063	1.10	0.011388
Cd36	A_51_P375138	L23108	CD36 antigen	0.51	0	0.66	0	0.77	1.82E-22	0.91	3.60E-06
*Cd36	A_51_P375146	NM_007643	CD36 antigen	0.51	0	0.58	3.82E-37	0.70	9.42E-13	0.89	0.000299
Cox4i1	A_51_P165435	NM_009941	cytochrome c oxidase subunit IV isoform 1	1.39	3.12E-23	1.08	0.01417	1.04	0.040811	1.03	0.396332
Cox6b1	A_51_P448032	NM_025628	cytochrome c oxidase, subunit VIb polypeptide 1	1.51	0	1.26	9.80E-17	1.28	8.13E-44	1.23	2.04E-32
Cox7a1	A_51_P148612	NM_009944	cytochrome c oxidase, subunit VIIa 1	2.45	0	1.29	0.000072	1.28	6.75E-22	1.29	3.31E-15
Cox7b	A_51_P160664	NM_025379	cytochrome c oxidase subunit VIIb	1.31	0.000086	1.04	0.499824	1.15	0.007799	1.07	0.219412
Cpt1a	A_52_P219753	NM_013495	carnitine palmitoyltransferase 1a, liver	1.20	0.010108	1.11	0.112697	1.02	0.640973	1.33	2.67E-13
Cs	A_51_P390260	NM_026444	citrate synthase	1.31	0.000192	1.13	0.058684	1.09	0.1476	1.03	0.612688
Cyp1a1	A_51_P279693	NM_009992	cytochrome P450, family 1, subfamily a, polypeptide 1	0.44	6.62E-23	0.68	7.93E-11	1.27	0.019932	0.95	0.502448
Cyp1b1	A_51_P255456	NM_009994	cytochrome P450, family 1, subfamily b, polypeptide 1	0.40	5.26E-11	0.62	4.99E-07	0.62	0.000034	0.64	2.90E-06
Cyp2c37	A_51_P498882	NM_010001	cytochrome P450, family 2. subfamily c, polypeptide 37	1.19	2.42E-07	1.01	0.898496	1.31	0.000091	1.19	9.95E-08
Cyp2c37	A_52_P625249	NM_010001	cytochrome P450, family 2. subfamily c, polypeptide 37	1.31	1.34E-20	1.18	0.001686	1.26	8.70E-23	1.18	2.80E-21
Cyp2e1	A_51_P283456	NM_021282	cytochrome P450, family 2, subfamily e, polypeptide 1	0.91	0.305965	0.42	3.08E-09	0.82	0.005241	1.07	0.473579

Cyp2f2	A_51_P453909	NM_007817	cytochrome P450, family 2, subfamily f, polypeptide 2	8.13	5.18E-23	1.59	0.000064	1.40	0.061488	1.57	0.000442
Cyp2j6	A_51_P506328	NM_010008	cytochrome P450, family 2, subfamily j, polypeptide 6	0.54	0	0.95	0.324548	0.88	1.67E-13	0.85	6.97E-06
Cyp2s1	A_51_P180091	NM_028775	cytochrome P450, family 2, subfamily s, polypeptide 1	1.40	3.83E-09	1.50	1.30E-10	1.34	3.01E-14	1.45	2.08E-18
Cyp2s1	A_52_P456837	AK054324	cytochrome P450, family 2, subfamily s, polypeptide 1	1.00	1	1.00	1	1.00	1	1.00	1
Cyp3a11	A_51_P355301	NM_007818	cytochrome P450, family 3, subfamily a, polypeptide 11	1.18	0.007357	1.32	1.41E-11	1.15	0.002934	1.15	0.000234
Cyp4a12a	A_52_P44914	NM_177406	cytochrome P450, family 4, subfamily a, polypeptide 12a	1.25	3.45E-09	1.27	0.004302	1.46	2.06E-07	1.31	3.01E-06
Cyp4b1	A_51_P118704	NM_007823	cytochrome P450, family 4, subfamily b, polypeptide 1	0.64	7.57E-21	0.51	8.41E-45	0.74	2.28E-07	1.60	7.73E-14
Cyp51	A_52_P164161	NM_020010	cytochrome P450, family 51	0.73	1.02E-15	0.90	0.082314	0.92	0.03594	0.92	0.024694
Cyp51	A_52_P636752	NM_020010	cytochrome P450, family 51	0.65	2.95E-14	0.88	0.054461	0.97	0.484686	1.03	0.48922
Dgat1	A_51_P510059	NM_010046	diacylglycerol O-acyltransferase 1	0.72	1.52E-06	1.00	0.974134	1.07	0.231587	1.32	0.000047
Dgat1	A_52_P456134	NM_010046	diacylglycerol O-acyltransferase 1	0.82	5.48E-11	1.06	0.040049	1.10	4.43E-07	1.41	6.39E-36
*Dgat2	A_52_P359739	NM_026384	diacylglycerol O-acyltransferase 2	0.98	0.76263	1.12	0.000396	1.24	0.000945	1.36	1.46E-07
Dlat	A_51_P265106	NM_145614	dihydrolipoamide S-acetyltransferase (E2 component of pyruvate dehydrogenase complex)	1.71	0	0.97	0.516877	1.02	0.335563	1.08	0.000154
Dld	A_51_P184284	NM_007861	dihydrolipoamide dehydrogenase	0.92	0.026784	0.74	1.09E-21	0.79	2.34E-11	0.83	2.01E-25
Dlst	A_51_P290139	NM_030225	dihydrolipoamide S-succinyltransferase (E2 component of 2-oxo-glutarate complex)	1.34	1.39E-11	1.05	0.28849	1.16	0.000591	1.17	0.000112
Eno2	A_51_P130028	NM_013509	enolase 2, gamma neuronal	0.69	0	1.26	2.69E-07	1.38	1.16E-13	1.18	1.17E-08
Eno2	A_52_P748882	NM_013509	enolase 2, gamma neuronal	0.69	2.54E-08	1.06	0.402799	1.36	6.02E-12	1.11	0.005316
*Eno3	A_51_P328539	NM_007933	enolase 3, beta muscle	2.83	0	1.72	0.646717	1.18	0.260241	1.24	1.30E-09
Fabp4	A_51_P336830	NM_024406	fatty acid binding protein 4, adipocyte	0.33	1.40E-37	0.48	3.38E-10	0.63	3.48E-11	1.20	0.033423
*Fabp4	A_51_P336833	NM_024406	fatty acid binding protein 4, adipocyte	0.30	0	0.40	2.42E-22	0.65	1.62E-15	1.30	0.010388
Fabp4	A_52_P564544	NM_024406	fatty acid binding protein 4, adipocyte	0.37	0	0.47	9.21E-39	0.71	3.42E-36	1.39	7.62E-36
Fabp5	A_51_P387764	NM_010634	fatty acid binding protein 5, epidermal	0.50	1.45E-06	0.62	0.026251	0.62	0.00005	1.36	0.000192
*Fasn	A_51_P321126	NM_007988	fatty acid synthase	0.84	0.067219	1.06	0.388131	1.14	0.84722	1.63	3.19E-10
Fasn	A_52_P100252	NM_007988	fatty acid synthase	0.92	0.475208	1.27	0.052164	1.16	0.128215	1.73	1.55E-29
Fh1	A_51_P359333	NM_010209	fumarate hydratase 1	1.36	0	1.00	0.888022	1.06	0.007412	1.04	0.020558
G6pd2	A_51_P353735	NM_019468	glucose-6-phosphate dehydrogenase 2	0.33	6.69E-12	0.39	3.95E-08	0.56	0.00062	0.99	0.861014
G6pdx	A_51_P187082	NM_008062	glucose-6-phosphate dehydrogenase X-linked	0.35	0	0.67	1.84E-10	0.63	7.44E-27	1.23	3.84E-13
Gapdh	A_52_P589321	AK169742	glyceraldehyde-3-phosphate dehydrogenase	1.82	4.67E-23	0.90	0.129224	1.10	0.253773	1.28	0.000291
Gck	A_51_P106294	NM_010292	glucokinase	0.58	5.42E-08	0.72	3.63E-06	0.82	0.004276	0.77	0.000195
Gck	A_52_P259537	NM_010292	glucokinase	0.47	1.16E-26	0.82	0.079532	0.71	2.90E-13	0.70	2.35E-19
Ggps1	A_52_P228247	NM_010282	geranylgeranyl diphosphate synthase 1	0.66	0	0.83	0.000969	0.85	2.90E-12	0.80	6.77E-07
*Gpd1	A_51_P293853	NM_010271	glycerol-3-phosphate dehydrogenase 1 (soluble)	1.10	0.772727	1.10	0.000046	0.88	0.954744	1.42	0.000212
Gpd1	A_52_P16419	NM_010271	glycerol-3-phosphate dehydrogenase 1 (soluble)	1.10	0.00167	1.09	0.000216	0.90	0.20562	1.39	0.000145
Gpt2	A_51_P493886	NM_173866	glutamic pyruvate transaminase (alanine aminotransferase) 2	1.45	7.26E-12	0.58	3.40E-13	0.56	0	0.61	0

Gyg	A_51_P240019	NM_013755	glycogenin	0.65	1.92E-17	0.84	0.000634	0.88	0.00568	0.85	0.002792
Gyk	A_51_P297671	NM_008194	glycerol kinase	0.52	2.93E-39	0.71	1.29E-07	0.79	0.000013	0.90	0.022782
Gyk	A_52_P1020291	AK037633	Mus musculus 16 days neonate thymus cDNA, RIKEN full-length enriched library, clone:A130030N17	0.74	0.136567	0.69	0.02115	1.04	0.77475	0.68	0.001291
Gyk	A_52_P120066	NM_212444	glycerol kinase	0.40	0	0.51	3.93E-18	0.50	1.70E-22	0.82	0.015727
Gys2	A_51_P440315	NM_145572	glycogen synthase 2	0.09	0	0.43	1.81E-07	0.55	8.26E-09	1.39	4.68E-10
Hadha	A_52_P193029	NM_178878	hydroxyacyl-Coenzyme A dehydrogenase/3-ketoacyl-Coenzyme A thiolase/enoyl-Coenzyme A hydratase, alpha subunit	1.39	2.84E-07	1.25	0.001498	1.37	5.44E-14	1.17	0.005489
*Hk1	A_51_P249024	NM_010438	hexokinase 1	1.80	0	1.57	4.03E-27	1.60	0	1.69	0
Hk1	A_52_P9337	NM_010438	hexokinase 1	1.49	0.000363	1.26	0.119948	1.29	0.002023	1.62	1.06E-17
Hmgcr	A_51_P507410	NM_008255	3-hydroxy-3-methylglutaryl-Coenzyme A reductase	0.69	8.62E-22	0.79	0.00002	0.81	9.64E-09	0.92	0.072297
Hmgcr	A_52_P137371	NM_008255	3-hydroxy-3-methylglutaryl-Coenzyme A reductase	0.61	4.59E-28	0.65	1.27E-27	0.65	2.60E-35	0.74	3.04E-10
Hmgcr	A_52_P232287	NM_008255	3-hydroxy-3-methylglutaryl-Coenzyme A reductase	0.71	5.93E-14	0.88	0.011546	0.82	0.000098	0.95	0.239244
Hmgcs1	A_51_P146941	NM_145942	3-hydroxy-3-methylglutaryl-Coenzyme A synthase 1	0.71	7.01E-44	0.82	0.000584	0.80	2.23E-10	1.09	0.008677
Hmgcs1	A_52_P388072	NM_145942	3-hydroxy-3-methylglutaryl-Coenzyme A synthase 1	0.67	0.000032	0.82	0.001709	0.83	0.010221	0.97	0.769464
Idh1	A_51_P132978	NM_010497	isocitrate dehydrogenase 1 (NADP+), soluble	0.68	0.000084	0.77	0.000016	0.77	7.27E-09	0.92	0.166721
Idh2	A_51_P331507	NM_173011	isocitrate dehydrogenase 2 (NADP+), mitochondrial	0.71	8.96E-08	0.86	0.002463	0.90	0.002773	0.86	0.000441
Idh3b	A_51_P130110	NM_130884	isocitrate dehydrogenase 3 (NAD+) beta	1.45	4.63E-35	1.17	8.78E-07	1.15	5.38E-08	1.12	1.18E-06
Idh3g	A_51_P136729	NM_008323	isocitrate dehydrogenase 3 (NAD+), gamma	1.54	0	1.12	0.000573	1.08	8.06E-09	1.06	0.038974
Idi1	A_52_P441634	NM_177960	isopentenyl-diphosphate delta isomerase	0.69	0.00434	0.92	0.53265	0.86	0.042166	0.76	0.000139
Ldha	A_51_P364140	NM_010699	lactate dehydrogenase A	1.54	1.39E-09	1.12	0.158779	1.21	0.003578	1.19	0.01037
Ldha	A_51_P364146	NM_010699	lactate dehydrogenase A	1.69	1.96E-21	1.27	0.000064	1.43	1.69E-12	1.35	2.58E-18
Ldhb	A_51_P409173	NM_008492	lactate dehydrogenase B	1.21	0.012288	1.20	0.000219	1.31	4.82E-08	1.15	0.035811
*Lipe	A_51_P435363	NM_010719	lipase, hormone sensitive	0.73	1.22E-06	0.91	0.286523	0.93	0.125576	1.33	3.06E-07
*Lpl	A_52_P257812	NM_008509	lipoprotein lipase	0.86	1.68E-07	0.72	2.81E-08	0.81	2.94E-12	0.80	2.98E-07
*Mgll	A_51_P139920	NM_011844	monoglyceride lipase	0.46	0	0.69	7.58E-11	0.86	4.92E-06	1.33	3.15E-41
Mgll	A_52_P568977	AK006949	monoglyceride lipase	0.31	5.72E-15	0.82	0.109462	0.79	0.015795	1.56	7.16E-06
Mgll	A_52_P609778	NM_011844	monoglyceride lipase	0.61	0	0.86	0.002675	0.96	0.209898	1.39	2.33E-43
Mvd	A_51_P355943	NM_138656	mevalonate (diphospho) decarboxylase	0.66	1.42E-13	1.09	0.042214	1.04	0.240567	0.90	0.000196
Mvk	A_51_P169527	NM_023556	mevalonate kinase	1.07	0.291723	1.24	0.00019	1.36	1.77E-06	1.27	0.000043
Mvk	A_52_P569067	NM_023556	mevalonate kinase	1.14	1.26E-06	1.29	7.39E-13	1.29	1.43E-18	1.34	8.97E-44
Ndufa1	A_51_P472405	NM_019443	NADH dehydrogenase (ubiquinone) 1 alpha subcomplex, 1	1.45	1.01E-10	1.14	0.019957	1.11	0.02005	1.14	0.000319
Ndufa1	A_52_P139747	NM_019443	NADH dehydrogenase (ubiquinone) 1 alpha subcomplex, 1	1.48	2.10E-27	1.09	0.004168	1.09	0.000408	1.07	0.004441
Ndufa5	A_51_P170156	NM_026614	NADH dehydrogenase (ubiquinone) 1 alpha subcomplex, 5	1.38	1.64E-10	1.07	0.078265	1.04	0.226138	1.05	0.061121
Oxct1	A_51_P107321	NM_024188	3-oxoacid CoA transferase 1	1.88	2.42E-32	0.97	0.644723	1.01	0.762601	1.05	0.272572

Oxct1	A_51_P107326	NM_024188	3-oxoacid CoA transferase 1	2.00	0	1.04	0.596856	1.11	0.000891	1.05	0.018299
Oxct1	A_52_P430058	NM_024188	3-oxoacid CoA transferase 1	2.06	1.75E-26	1.02	0.768714	1.08	0.049389	1.08	0.17035
Oxct2a	A_51_P125062	NM_022033	3-oxoacid CoA transferase 2A	1.40	0.018547	1.40	0.110332	0.98	0.906874	1.71	0.000074
Pcca	A_51_P484254	NM_144844	propionyl-Coenzyme A carboxylase, alpha polypeptide	1.40	2.47E-29	1.08	0.018049	1.15	0.001581	1.11	0.003675
Pdk4	A_51_P350453	NM_013743	pyruvate dehydrogenase kinase, isoenzyme 4	2.44	0	1.28	0.000148	1.21	0.025135	1.03	0.760499
Pecr	A_51_P291749	NM_023523	peroxisomal trans-2-enoyl-CoA reductase	1.42	2.54E-14	1.28	3.28E-08	1.34	4.14E-12	1.16	0.000148
Pgam2	A_51_P264495	NM_018870	phosphoglycerate mutase 2	1.43	0	1.07	0.067581	1.12	0.000358	1.10	0.001147
Pgd	A_51_P391996	BC011329	phosphogluconate dehydrogenase	0.66	1.57E-16	0.99	0.806217	1.00	0.997956	1.18	0.000767
Pgd	A_52_P84096	BC011329	phosphogluconate dehydrogenase	0.64	0	0.95	0.139419	0.90	0.012729	1.15	1.16E-11
Pgk2	A_51_P125487	NM_031190	phosphoglycerate kinase 2	3.73	1.01E-06	2.34	0.000835	0.95	0.710556	2.31	0.000036
Pgm1	A_51_P427530	NM_025700	phosphoglucomutase 1	0.67	3.59E-07	0.79	3.61E-12	0.78	2.57E-08	0.85	0.00018
*Pgm2	A_51_P368074	NM_028132	phosphoglucomutase 2	1.60	4.32E-21	1.41	0.002642	1.21	0.000066	1.13	0.001763
Pgm5	A_51_P451052	NM_175013	phosphoglucomutase 5	1.39	4.44E-24	1.30	4.10E-12	1.26	7.24E-24	1.21	7.10E-09
Phka1	A_52_P196825	AK078633	Mus musculus adult male adrenal gland cDNA, RIKEN full-length enriched library, clone:7330411B16	0.57	1.33E-15	0.84	0.036055	0.96	0.496911	1.04	0.621088
Phka1	A_52_P278311	NM_008832	phosphorylase kinase alpha 1	0.75	0.005421	0.70	3.55E-06	0.89	0.15683	0.79	0.003431
Phka2	A_52_P187217	NM_172783	phosphorylase kinase alpha 2	0.54	2.62E-06	1.01	0.937226	1.07	0.663911	0.96	0.766446
Phka2	A_52_P671916	NM_172783	phosphorylase kinase alpha 2	0.71	8.95E-10	0.95	0.154461	0.95	0.248076	1.03	0.481344
Phkb	A_51_P307624	NM_199446	phosphorylase kinase beta	1.13	0.031243	0.73	6.54E-08	0.83	0.000019	0.79	8.19E-15
Phkg1	A_51_P280890	NM_011079	phosphorylase kinase gamma 1	3.45	0	1.12	0.274725	1.29	7.44E-08	1.30	0.000027
Pklr	A_51_P176042	NM_013631	pyruvate kinase liver and red blood cell	1.08	0.115087	1.33	1.17E-06	1.22	0.000106	1.23	0.009216
Pkm2	A_51_P428913	D38379	pyruvate kinase, muscle	1.54	1.53E-28	1.33	2.62E-06	1.45	3.18E-26	1.44	1.30E-24
*Pkm2	A_52_P150212	NM_011099	pyruvate kinase, muscle	1.46	5.65E-07	1.44	0.000023	1.45	5.86E-14	1.42	4.25E-11
Pmvk	A_51_P492408	NM_026784	phosphomevalonate kinase	1.36	1.33E-35	1.06	0.145874	1.10	0.008097	1.31	7.49E-23
Pmvk	A_51_P492410	NM_026784	phosphomevalonate kinase	1.37	1.24E-39	1.10	0.00535	1.07	0.0029	1.28	1.21E-22
Ppap2a	A_51_P189104	NM_008903	phosphatidic acid phosphatase 2a	0.76	3.18E-10	1.06	0.045156	1.04	0.313021	1.16	4.41E-12
Ppap2a	A_51_P189105	AK077275	phosphatidic acid phosphatase 2a	0.62	4.63E-32	0.91	0.073834	0.88	0.000138	0.93	0.043749
Ppap2a	A_52_P7937	NM_008903	phosphatidic acid phosphatase 2a	0.65	2.09E-21	0.96	0.174077	0.95	0.124923	1.01	0.633173
Pparg	A_51_P106799	NM_011146	peroxisome proliferator activated receptor gamma	0.99	0.550338	0.97	0.492418	1.04	0.011986	1.39	4.02E-34
Ppargc1a	A_51_P279038	NM_008904	peroxisome proliferative activated receptor, gamma, coactivator 1 alpha	1.38	4.71E-16	0.89	0.025043	0.86	0.000178	0.79	0.008952
Ppargc1a	A_52_P5945	AK032149	peroxisome proliferative activated receptor, gamma, coactivator 1 alpha	0.98	0.547165	0.73	8.04E-09	0.81	1.30E-08	0.94	0.373556
Ppargc1b	A_51_P294891	NM_133249	peroxisome proliferative activated receptor, gamma, coactivator 1 beta	1.95	0	1.32	1.40E-44	1.15	0.000013	1.23	0.000371
Ppargc1b	A_52_P293222	AK042378	peroxisome proliferative activated receptor, gamma, coactivator 1 beta	2.07	0	1.17	0.012676	1.12	0.054584	1.05	0.337101
Ppargc1b	A_52_P526724	NM_133249	peroxisome proliferative activated receptor, gamma,	1.72	0	1.25	2.02E-13	1.09	0.007647	1.16	0.001458

			coactivator 1 beta								
Ppp1cb	A_51_P342716	NM_172707	protein phosphatase 1, catalytic subunit, beta isoform	0.76	0	0.83	9.55E-08	0.89	4.49E-06	0.86	7.01E-11
Pygb	A_52_P350664	NM_153781	brain glycogen phosphorylase	0.88	0.102715	1.23	0.020399	1.36	6.83E-08	1.22	0.000313
Pygl	A_51_P452779	NM_133198	liver glycogen phosphorylase	0.68	0.000168	0.95	0.510055	0.95	0.51978	1.41	2.89E-11
*Pygm	A_51_P458973	NM_011224	muscle glycogen phosphorylase	1.66	2.82E-31	1.26	0.271705	1.05	0.524452	0.99	0.232421
Scd1	A_52_P682382	NM_009127	stearoyl-Coenzyme A desaturase 1	0.42	3.25E-26	0.87	0.263589	0.97	0.685961	1.12	0.016046
Scd2	A_51_P129464	NM_009128	stearoyl-Coenzyme A desaturase 2	0.55	2.56E-34	0.98	0.823701	0.86	0.000119	0.91	0.011277
Sdha	A_51_P410823	NM_023281	succinate dehydrogenase complex, subunit A, flavoprotein (Fp)	1.33	4.71E-25	0.92	0.024253	0.93	0.039836	0.93	0.005933
Sdhc	A_52_P875481	AK031343	Mus musculus 13 days embryo male testis cDNA, RIKEN full-length enriched library, clone:6030410A06	0.77	0.164235	0.47	0.000076	0.47	5.50E-06	0.57	0.005292
Slc25a1	A_51_P350922	NM_153150	solute carrier family 25 (mitochondrial carrier, citrate transporter), member 1	0.44	2.25E-35	0.73	0.000243	0.66	2.68E-07	1.46	8.98E-29
Slc27a1	A_51_P117477	NM_011977	solute carrier family 27 (fatty acid transporter), member 1	1.37	2.49E-10	1.40	6.09E-08	1.29	0.000279	1.33	5.86E-08
Slc27a3	A_51_P133252	NM_011988	solute carrier family 27 (fatty acid transporter), member 3	1.63	7.89E-09	1.49	1.21E-07	1.33	5.71E-08	1.44	2.42E-07
Sucla2	A_51_P111554	NM_011506	succinate-Coenzyme A ligase, ADP-forming, beta subunit	1.50	2.69E-29	0.81	0.000049	0.91	0.047026	0.88	0.000016
Sucla2	A_52_P762514	AK016060	succinate-Coenzyme A ligase, ADP-forming, beta subunit	0.66	0.028146	0.86	0.564567	0.62	0.000271	0.70	0.204455
Suclg1	A_51_P491227	NM_019879	succinate-CoA ligase, GDP-forming, alpha subunit	1.49	1.40E-45	1.03	0.451148	1.06	0.010828	1.06	0.005051
Taldo1	A_51_P401958	NM_011528	transaldolase 1	0.75	1.11E-11	0.95	0.300857	0.91	0.054578	0.96	0.481772
Tkt	A_51_P394515	NM_009388	transketolase	1.02	0.752069	0.94	0.128476	0.88	0.001529	1.39	4.17E-15
Tkt	A_52_P624447	NM_009388	transketolase	0.61	0.016841	1.03	0.902915	0.69	0.01895	1.80	0.000054
Tpi1	A_51_P433824	NM_009415	triosephosphate isomerase 1	1.54	1.07E-35	1.05	0.414226	1.14	1.07E-06	1.23	5.77E-15
Tpi1	A_52_P116102	AK053780	Mus musculus 0 day neonate eyeball cDNA, RIKEN library, clone:E130308D14 product:triosephosphate isomerase	2.78	0	1.49	5.21E-06	1.56	1.19E-09	1.33	0.004216
Ucp1	A_51_P426353	NM_009463	uncoupling protein 1 (mitochondrial, proton carrier)	0.04	0	0.07	0	0.19	3.23E-14	0.65	0.000095
Ucp2	A_51_P297105	NM_011671	uncoupling protein 2 (mitochondrial, proton carrier)	0.44	0	0.82	0.00721	1.09	0.069029	1.14	4.35E-08
Ucp2	A_52_P90265	NM_011671	uncoupling protein 2 (mitochondrial, proton carrier)	0.33	4.67E-26	0.71	0.001089	0.99	0.930012	0.98	0.737949
Ugp2	A_52_P42242	NM_139297	UDP-glucose pyrophosphorylase 2	0.90	0.000077	0.70	1.54E-32	0.87	0.018978	0.83	8.34E-17
Uqcrb	A_51_P472671	NM_026219	ubiquinol-cytochrome c reductase binding protein	1.39	3.25E-20	1.14	0.00007	1.12	1.43E-06	1.09	1.22E-07
Uqcrc1	A_51_P109835	NM_025407	ubiquinol-cytochrome c reductase core protein 1	1.95	0	1.48	3.51E-10	1.71	0	1.49	2.49E-19
Uqcrh	A_51_P431852	NM_025641	ubiquinol-cytochrome c reductase hinge protein	1.33	0.000849	1.06	0.420679	1.01	0.935693	1.08	0.433436
Acaa1a	A_51_P327075	NM_130864	acetyl-Coenzyme A acyltransferase 1A	1.19	4.48E-16	1.06	0.124979	1.04	0.04893	1.13	1.93E-07
Acaa1a	A_52_P423183	NM_130864	acetyl-Coenzyme A acyltransferase 1A	1.14	0.000125	1.06	0.132409	1.11	0.015277	1.29	1.20E-17
Acaa1b	A_52_P423174	NM_146230	acetyl-Coenzyme A acyltransferase 1B	1.05	0.022239	0.96	0.322991	1.01	0.822135	1.23	2.65E-13
Acaca	A_52_P23177	NM_133360	acetyl-Coenzyme A carboxylase alpha	0.98	0.700773	1.04	0.367526	1.04	0.381595	1.07	0.048241
Acacb	A_51_P239236	BC022940	acetyl-Coenzyme A carboxylase beta	0.85	0.004376	0.92	0.067161	1.02	0.691765	0.96	0.488229
Acacb	A_52_P340167	NM_133904	acetyl-Coenzyme A carboxylase beta	0.98	0.667489	1.04	0.246585	1.14	7.58E-06	1.10	0.003934

Acad10	A_51_P187507	NM_028037	acyl-Coenzyme A dehydrogenase family, member 10	1.25	1.67E-20	1.11	0.000559	1.14	1.01E-08	1.21	2.58E-25
Acad11	A_51_P299260	AK041180	acyl-Coenzyme A dehydrogenase family, member 11	1.24	0.144892	1.39	0.092842	0.91	0.549948	0.95	0.498038
Acad11	A_52_P85152	NM_175324	acyl-Coenzyme A dehydrogenase family, member 11	1.28	2.41E-10	0.95	0.231552	1.06	0.114712	1.08	0.000169
Acad8	A_51_P244052	NM_025862	acyl-Coenzyme A dehydrogenase family, member 8	1.15	0.000204	1.06	0.028879	1.08	0.003063	1.03	0.283401
Acad9	A_51_P341379	NM_172678	acyl-Coenzyme A dehydrogenase family, member 9	1.08	0.009483	0.97	0.213639	0.95	0.004397	0.99	0.582952
Acad9	A_52_P73208	AK049931	acyl-Coenzyme A dehydrogenase family, member 9	0.87	0.117121	0.91	0.307918	0.94	0.317526	0.90	0.063788
Acadl	A_51_P149455	NM_007381	acyl-Coenzyme A dehydrogenase, long-chain	0.87	9.52E-10	0.83	9.03E-13	0.82	1.81E-15	0.79	1.10E-28
Acadm	A_51_P319879	NM_007382	acyl-Coenzyme A dehydrogenase, medium chain	0.97	0.179736	0.90	0.004395	0.94	0.000947	0.88	0.000507
Acadvl	A_51_P518340	NM_017366	acyl-Coenzyme A dehydrogenase, very long chain	1.10	0.033193	1.15	0.000501	1.13	0.003419	1.05	0.382295
Acat1	A_51_P319449	NM_144784	acetyl-Coenzyme A acetyltransferase 1	0.99	0.778864	0.94	0.132995	0.97	0.360534	0.90	0.009468
Acat3	A_51_P139745	NM_153151	acetyl-Coenzyme A acetyltransferase 3	1.02	0.641872	1.21	0.000011	1.16	1.99E-06	1.27	1.75E-07
Acat3	A_52_P11959	NM_153151	acetyl-Coenzyme A acetyltransferase 3	0.72	0.006707	0.74	0.010481	0.97	0.80882	1.02	0.781088
Acly	A_51_P261718	NM_134037	ATP citrate lyase	0.81	2.59E-06	0.91	0.021868	0.92	0.052358	0.96	0.287573
Acly	A_52_P373556	AK043466	ATP citrate lyase	1.06	0.520694	1.12	0.619508	1.54	0.003309	0.87	0.138099
Aco1	A_51_P391082	NM_007386	aconitase 1	0.93	0.000178	1.01	0.530912	1.04	0.089094	1.18	2.32E-16
Acox3	A_52_P745	NM_030721	acyl-Coenzyme A oxidase 3, pristanoyl	1.17	1.45E-23	1.10	0.00069	1.18	2.96E-12	1.15	1.49E-12
Acsl1	A_51_P463452	NM_007981	acyl-CoA synthetase long-chain family member 1	0.84	2.77E-06	0.77	3.30E-09	0.83	8.62E-06	0.89	8.05E-08
Acsl1	A_51_P496432	BC006692	acyl-CoA synthetase long-chain family member 1	0.78	2.20E-09	0.80	1.52E-11	0.80	2.71E-09	0.83	1.17E-20
Acsl3	A_51_P511560	NM_028817	acyl-CoA synthetase long-chain family member 3	0.80	1.77E-09	0.81	3.16E-16	0.83	2.50E-07	0.80	1.95E-07
Acsl4	A_51_P268154	NM_019477	acyl-CoA synthetase long-chain family member 4	1.04	0.096446	1.01	0.818853	0.96	0.093896	0.94	0.011828
Acsl4	A_52_P78203	NM_207625	acyl-CoA synthetase long-chain family member 4	1.06	0.002837	0.94	0.02299	0.87	3.36E-26	0.89	1.28E-12
Acsl5	A_52_P593037	NM_027976	acyl-CoA synthetase long-chain family member 5	1.15	4.36E-06	1.13	0.000777	1.08	0.00297	1.23	1.01E-24
Acsl6	A_51_P380699	NM_144823	acyl-CoA synthetase long-chain family member 6	1.04	0.249149	1.07	0.308739	0.99	0.786668	0.99	0.897006
Acsl6	A_51_P518823	AK147421	acyl-CoA synthetase long-chain family member 6	1.27	0.000011	0.98	0.707002	1.00	0.936123	1.02	0.650641
Adcy1	A_51_P196718	NM_009622	adenylate cyclase 1	0.80	2.99E-06	0.83	0.000024	1.05	0.531828	0.95	0.329481
Adcy1	A_51_P409900	NM_009622	adenylate cyclase 1	0.98	0.83287	0.84	0.365628	1.20	0.221148	0.91	0.177997
Adcy1	A_52_P240164	NM_009622	adenylate cyclase 1	1.03	0.465751	1.07	0.251423	1.01	0.808106	1.09	0.058635
Adcy1	A_52_P240170	BC050125	adenylate cyclase 1	1.20	0.000011	1.11	0.134943	1.02	0.577492	1.04	0.337451
Adcy2	A_51_P337523	NM_153534	adenylate cyclase 2	1.02	0.290852	0.95	0.183087	1.02	0.488724	1.01	0.857267
Adcy2	A_52_P165610	NM_153534	adenylate cyclase 2	0.96	0.470969	0.97	0.530995	1.02	0.656706	1.00	0.982121
Adcy3	A_51_P484671	NM_138305	adenylate cyclase 3	0.75	0.04966	0.83	0.040127	0.86	0.003016	1.12	0.315755
Adcy3	A_52_P216525	NM_138305	adenylate cyclase 3	0.85	0.101386	0.84	0.068027	0.97	0.808796	0.88	0.2514
Adcy6	A_51_P243134	NM_007405	adenylate cyclase 6	1.05	0.340208	1.10	0.037898	1.16	0.00061	1.12	0.016211
Adcy7	A_51_P441622	NM_007406	adenylate cyclase 7	0.98	0.792695	1.15	0.068732	1.14	0.001941	1.08	0.06989
Adcy7	A_52_P291	AK012436	adenylate cyclase 7	1.22	0.000405	1.12	0.192001	1.24	4.64E-06	1.17	0.016065

Adcy7	A_52_P586141	NM_001037724	Mus musculus 11 days embryo whole body cDNA, RIKEN library, clone:2700055K03 product:adenylate cyclase 7	1.05	0.289626	1.08	0.237832	1.08	0.085512	1.09	0.031379
Adcy8	A_51_P303061	NM_009623	adenylate cyclase 8	1.05	0.712023	0.99	0.963571	1.06	0.668835	1.02	0.78393
Adcy9	A_51_P501538	NM_009624	adenylate cyclase 9	1.12	0.106816	0.89	0.02162	0.91	0.116732	0.94	0.169668
Adh1	A_51_P428555	NM_007409	alcohol dehydrogenase 1 (class I)	0.79	0.037549	1.11	0.003409	0.81	2.98E-06	0.87	0.008729
Adh1	A_52_P629895	NM_007409	alcohol dehydrogenase 1 (class I)	0.84	0.140847	1.21	6.49E-07	0.90	0.016049	0.92	0.064815
Adh1	A_52_P835985	AK082149	alcohol dehydrogenase 1 (class I)	0.67	0.119992	1.08	0.607756	1.08	0.647858	0.98	0.893785
Adh4	A_51_P189442	NM_011996	alcohol dehydrogenase 4 (class II), pi polypeptide	0.98	0.905575	0.92	0.72232	1.27	0.31604	1.04	0.817235
Adh5	A_51_P404275	NM_007410	alcohol dehydrogenase 5 (class III), chi polypeptide	0.94	0.003139	0.93	0.00977	0.97	0.307343	0.91	7.09E-12
Adh6a	A_51_P299608	AK028114	alcohol dehydrogenase 6A (class V)	1.00	1	1.00	1	1.00	1	1.00	1
Adh6a	A_52_P276348	AK007397	alcohol dehydrogenase 6A (class V)	0.99	0.971309	0.94	0.701672	0.96	0.707833	1.22	0.367623
Adh6-ps1	A_51_P120027	AK004863	alcohol dehydrogenase 6 (class V), pseudogene 1	1.00	1	1.00	1	1.00	1	1.00	1
Adh7	A_51_P233797	NM_009626	alcohol dehydrogenase 7 (class IV), mu or sigma polypeptide	1.00	1	0.85	0.521454	1.00	1	0.99	0.872715
Agpat1	A_51_P322612	NM_018862	1-acylglycerol-3-phosphate O-acyltransferase 1 (lysophosphatidic acid acyltransferase, alpha)	1.15	1.63E-08	1.08	0.014635	1.07	0.000684	1.09	3.10E-10
Agpat1	A_52_P200465	NM_018862	1-acylglycerol-3-phosphate O-acyltransferase 1 (lysophosphatidic acid acyltransferase, alpha)	0.95	0.035511	0.94	0.148671	0.90	0.003636	0.96	0.247308
Agpat3	A_51_P425490	NM_053014	1-acylglycerol-3-phosphate O-acyltransferase 3	0.83	2.69E-10	1.01	0.774893	0.98	0.634009	0.98	0.4885
Agpat3	A_52_P523569	NM_053014	1-acylglycerol-3-phosphate O-acyltransferase 3	0.87	7.08E-11	0.97	0.362706	0.92	0.00007	0.97	0.109094
Agpat4	A_51_P346165	NM_026644	1-acylglycerol-3-phosphate O-acyltransferase 4 (lysophosphatidic acid acyltransferase, delta)	1.04	0.066346	1.12	0.001252	1.06	0.075331	1.11	3.92E-07
Agpat4	A_52_P3029	NM_026644	1-acylglycerol-3-phosphate O-acyltransferase 4 (lysophosphatidic acid acyltransferase, delta)	1.07	0.003171	1.09	0.009373	1.05	0.201351	1.12	1.09E-09
Agpat5	A_51_P361286	NM_026792	1-acylglycerol-3-phosphate O-acyltransferase 5 (lysophosphatidic acid acyltransferase, epsilon)	1.20	0.000231	0.89	0.023252	0.87	0.002017	0.90	0.002004
Agpat5	A_52_P572447	NM_026792	1-acylglycerol-3-phosphate O-acyltransferase 5 (lysophosphatidic acid acyltransferase, epsilon)	0.88	0.000267	0.88	0.000424	0.91	0.004933	0.94	0.049811
Agpat6	A_51_P335710	NM_018743	1-acylglycerol-3-phosphate O-acyltransferase 6 (lysophosphatidic acid acyltransferase, zeta)	0.84	0.117017	0.90	0.266944	0.98	0.890974	1.04	0.674377
Agpat6	A_51_P335716	NM_018743	1-acylglycerol-3-phosphate O-acyltransferase 6 (lysophosphatidic acid acyltransferase, zeta)	0.87	0.110022	0.99	0.83477	1.07	0.323694	1.08	0.221706
Agpat6	A_52_P24986	NM_018743	1-acylglycerol-3-phosphate O-acyltransferase 6 (lysophosphatidic acid acyltransferase, zeta)	0.96	0.013561	1.02	0.506785	1.07	2.42E-07	1.15	1.52E-26
Alb	A_51_P160713	NM_009654	albumin	1.18	0.364614	1.45	0.021919	1.13	0.542957	1.75	0.046628
Aldh1a3	A_52_P113518	AK086764	aldehyde dehydrogenase family 1, subfamily A3	1.10	0.509334	1.57	0.04757	1.07	0.748608	1.24	0.290435
Aldh1a3	A_52_P87843	NM_053080	aldehyde dehydrogenase family 1, subfamily A3	1.03	0.743357	1.13	0.083279	1.14	0.000788	1.16	0.000516
Aldh3a1	A_51_P310594	NM_007436	aldehyde dehydrogenase family 3, subfamily A1	1.00	1	1.00	1	1.00	1	1.06	0.640629
Aldh3a2	A_51_P464175	NM_007437	aldehyde dehydrogenase family 3, subfamily A2	0.90	0.013501	0.92	0.009309	0.95	0.198421	0.94	0.094363
Aldh4a1	A_51_P337918	NM_175438	aldehyde dehydrogenase 4 family, member A1	1.26	4.27E-21	0.97	0.167397	1.00	0.964569	1.06	0.00895
Aldh4a1	A_52_P123230	NM_175438	aldehyde dehydrogenase 4 family, member A1	1.01	0.891926	0.98	0.884763	1.13	0.431356	0.95	0.471991
Aldh7a1	A_51_P470414	NM_138600	aldehyde dehydrogenase family 7, member A1	1.00	0.972001	0.84	0.434428	1.16	0.547829	1.29	0.283871

	Aldh7a1	A_52_P503071	NM_138600	aldehyde dehydrogenase family 7, member A1	1.06	0.681393	1.13	0.336483	1.06	0.67815	1.09	0.516753
	Aldh9a1	A_52_P265877	NM_019993	aldehyde dehydrogenase 9, subfamily A1	0.91	0.05481	0.98	0.750604	0.94	0.393878	1.08	0.273649
	Aldob	A_51_P337269	NM_144903	aldolase 2, B isoform	0.79	0.128264	0.63	0.08003	1.25	0.522415	1.04	0.803962
	Aldob	A_52_P450038	NM_144903	aldolase 2, B isoform	1.00	1	1.00	1	1.00	1	1.00	1
	Aldob	A_52_P468023	NM_144903	aldolase 2, B isoform	1.00	1	1.00	1	1.00	1	1.04	0.699964
	Aldoc	A_51_P220681	NM_009657	aldolase 3, C isoform	1.01	0.914675	1.09	0.045509	1.19	6.52E-06	1.01	0.868568
	Aldoc	A_51_P425284	AK039267	aldolase 3, C isoform	0.96	0.689349	0.90	0.479271	1.15	0.373789	1.95	0.004851
	Apoa1	A_51_P408082	NM_009692	apolipoprotein A-I	1.17	0.008365	1.13	0.109316	1.08	0.363485	1.04	0.498525
	Apoa4	A_51_P327491	NM_007468	apolipoprotein A-IV	1.13	0.111229	1.39	0.116943	1.11	0.451716	1.43	0.006038
	Apoa5	A_51_P259930	NM_080434	apolipoprotein A-V	1.04	0.530319	1.02	0.697154	1.06	0.459395	1.07	0.365893
	Apoa5	A_52_P292404	NM_080434	apolipoprotein A-V	1.00	1	1.02	0.876394	1.06	0.578807	1.03	0.765289
	Apob	A_51_P380650	XM_137955	Mus musculus apolipoprotein B (Apob), mRNA.	0.86	0.401579	1.18	0.470212	0.91	0.60581	1.05	0.82701
	Apob	A_51_P413088	XM_137955	Mus musculus apolipoprotein B (Apob), mRNA.	0.90	0.661632	1.06	0.756031	0.91	0.557853	0.86	0.571067
	Apob	A_51_P436690	BC028880	Mus musculus apolipoprotein B (Apob), mRNA.	0.98	0.853085	1.20	0.401549	1.00	1	1.00	1
	Apob	A_51_P470542	XM_137955	apolipoprotein B	1.17	0.461651	0.93	0.666246	1.24	0.392811	0.95	0.797845
	Apob	A_52_P441070	NM_009693	apolipoprotein B	1.02	0.800112	0.98	0.774689	1.00	1	1.04	0.728297
	Apoc3	A_51_P310629	NM_023114	apolipoprotein C-III	0.75	0.125071	0.70	0.027229	0.80	0.33056	1.12	0.588091
	Apoc3	A_52_P574306	NM_023114	apolipoprotein C-III	1.00	1	1.04	0.724699	1.00	1	1.00	1
	Apoe	A_51_P171999	NM_009696	apolipoprotein E	0.83	0.000102	0.99	0.77505	0.98	0.682914	1.01	0.778489
	Atp12a	A_52_P329398	NM_138652	ATPase, H+/K+ transporting, nongastric, alpha polypeptide	1.07	0.18906	1.22	0.013612	1.06	0.168039	1.07	0.092125
	Atp5a1	A_52_P674489	NM_007505	ATP synthase, H+ transporting, mitochondrial F1 complex, alpha subunit, isoform 1	1.13	0.195469	1	0.94	1.016	0.804455	0.99	0.95
	Atp5b	A_51_P207636	NM_016774	ATP synthase, H+ transporting mitochondrial F1 complex, beta subunit	1.21	3.70E-08	1.03	0.081285	1.10	0.003466	1.11	0.000014
	Atp5b	A_52_P328078	NM_016774	ATP synthase, H+ transporting mitochondrial F1 complex, beta subunit	1.04	0.682844	0.98	0.866268	1.00	0.981293	1.14	0.333119
	Atp5b	A_52_P553841	NM_016774	ATP synthase, H+ transporting mitochondrial F1 complex, beta subunit	1.15	0.058434	1.01	0.836146	0.97	0.676376	0.97	0.67955
	Atp5c1	A_51_P378087	NM_020615	ATP synthase, H+ transporting, mitochondrial F1 complex, gamma polypeptide 1	0.99	0.894279	1.03	0.669621	0.81	0.023186	0.90	0.312507
	Atp5h	A_51_P264186	NM_027862	ATP synthase, H+ transporting, mitochondrial F0 complex, subunit d	1.09	0.111437	0.90	0.003453	0.92	0.012271	0.91	0.016054
	Atp5h	A_52_P640355	NM_027862	ATP synthase, H+ transporting, mitochondrial F0 complex, subunit d	1.19	2.02E-15	1.01	0.695467	1.02	0.220206	1.02	0.500562
	Atp6ap1	A_51_P447988	NM_018794	ATPase, H+ transporting, lysosomal accessory protein 1	0.87	0.008409	0.95	0.204446	0.94	0.0464	0.96	0.160106
	Atp6v0a1	A_51_P142089	NM_016920	ATPase, H+ transporting, lysosomal V0 subunit A1	0.80	3.47E-31	1.04	0.115276	1.06	0.000244	1.14	2.33E-07
	Atp6v1b2	A_51_P190845	NM_007509	ATPase, H+ transporting, lysosomal V1 subunit B2	0.95	0.385013	0.97	0.64691	0.95	0.404868	0.98	0.70339
	Atp6v1e1	A_51_P166434	NM_007510	VATPase, H+ transporting, lysosomal V1 subunit E1	0.87	0.000149	0.86	4.56E-07	0.86	4.05E-09	0.90	0.000157
	Atp6v1e1	A_52_P303388	NM_007510	VATPase, H+ transporting, lysosomal V1 subunit E1	0.90	0.002498	0.91	0.016895	0.89	0.001121	0.90	0.007861

	Atp6v1g1	A_51_P140171	NM_024173	ATPase, H+ transporting, lysosomal V1 subunit G1	0.89	3.16E-12	0.92	0.000067	0.93	0.000174	0.96	0.032797
	Atp7a	A_51_P312497	NM_009726	ATPase, Cu++ transporting, alpha polypeptide	1.00	0.929925	0.94	0.017379	0.94	0.00008	0.96	0.040515
	Atp7a	A_51_P473179	AK033254	ATPase, Cu++ transporting, alpha polypeptide	1.24	0.149263	0.88	0.064741	0.98	0.891809	0.95	0.524953
	Auh	A_51_P372473	NM_016709	AU RNA binding protein/enoyl-coenzyme A hydratase	1.10	0.000293	1.01	0.799326	1.06	0.009388	1.05	0.017079
	Auh	A_52_P107290	AK003929	AU RNA binding protein/enoyl-coenzyme A hydratase	0.56	1.87E-23	0.99	0.908521	0.93	0.198815	1.16	0.020361
	Auh	A_52_P4095	AK169661	AU RNA binding protein/enoyl-coenzyme A hydratase	0.89	0.187684	1.05	0.616422	1.02	0.855085	1.04	0.487423
	Bcnp1	A_52_P1053160	AK158308	BCNP1 homolog	0.91	0.060879	0.88	0.003021	0.92	0.035934	1.06	0.144708
	Bdh1	A_51_P163106	NM_175177	3-hydroxybutyrate dehydrogenase, type 1	1.01	0.789445	1.00	0.96248	0.91	0.125171	1.03	0.46998
	Bdh1	A_52_P468343	AK146962	3-hydroxybutyrate dehydrogenase, type 1	1.11	3.58E-08	0.96	0.250564	1.07	0.007208	1.11	0.000014
	Bdh2	A_52_P195809	NM_027208	3-hydroxybutyrate dehydrogenase, type 2	1.29	7.84E-07	1.21	0.000109	1.01	0.843347	0.99	0.865289
	Btd	A_51_P196158	NM_025295	biotinidase	0.92	0.005392	1.01	0.750581	1.01	0.674532	1.09	0.001397
	Cat	A_52_P679769	NM_009804	catalase	0.77	2.17E-07	0.95	0.277851	0.91	0.00025	0.91	0.016005
	Coq2	A_51_P447752	NM_027978	coenzyme Q2 homolog, prenyltransferase (yeast)	1.23	1.08E-14	1.12	0.00002	1.11	9.31E-08	1.14	1.63E-09
	Cox1	A_51_P315595	X57780	M.musculus mRNA for mitochondrial gene for subunit I of cytochrome c oxidase.	1.35	0.004183	0.9	0.300792	0.81	0.048438	0.94	0.501250
	Cox6a1	A_51_P311540	NM_007748	cytochrome c oxidase, subunit VI a, polypeptide 1	0.92	0.00068	0.90	0.027116	0.93	0.028754	1.11	0.000188
	Cox6b2	A_51_P300506	NM_183405	cytochrome c oxidase subunit VIb polypeptide 2	1.22	8.67E-35	1.12	0.000074	1.15	1.23E-17	1.12	1.34E-07
	Cpt1a	A_51_P427674	NM_013495	carnitine palmitoyltransferase 1a, liver	1.10	0.037464	0.79	0.001022	0.82	0.004255	1.27	2.30E-07
	Cpt1b	A_51_P232913	NM_009948	carnitine palmitoyltransferase 1b, muscle	0.93	0.182985	0.97	0.501011	1.13	0.005288	0.95	0.124335
	Cpt1b	A_52_P625508	NM_009948	carnitine palmitoyltransferase 1b, muscle	1.05	0.21845	1.10	0.000836	1.17	5.44E-12	1.13	3.20E-06
	Cpt1c	A_51_P177562	NM_153679	carnitine palmitoyltransferase 1c	0.99	0.921547	1.00	0.994404	1.06	0.510973	1.10	0.432697
	Cpt1c	A_52_P269942	NM_153679	carnitine palmitoyltransferase 1c	1.15	0.036602	1.07	0.198124	1.26	4.53E-25	1.25	8.10E-07
	Cpt2	A_51_P403388	NM_009949	carnitine palmitoyltransferase 2	1.22	4.59E-06	1.17	0.000793	1.20	6.34E-07	1.13	0.000924
	Crat	A_51_P440807	NM_007760	carnitine acetyltransferase	1.08	0.000211	1.03	0.254661	1.00	0.813586	0.98	0.254275
	Crot	A_51_P489153	NM_023733	carnitine O-octanoyltransferase	0.97	0.480869	0.90	0.004802	0.92	0.009612	0.93	0.024226
	Crot	A_52_P656336	NM_023733	carnitine O-octanoyltransferase	0.75	0.011302	0.90	0.233425	0.83	0.047341	0.89	0.16889
	Cs	A_52_P182659	NM_026444	citrate synthase	1.04	0.471665	0.89	0.006963	0.99	0.864901	0.98	0.707034
	Cyp19a1	A_51_P474551	NM_007810	cytochrome P450, family 19, subfamily a, polypeptide 1	1.10	0.384145	1.39	0.019697	1.24	0.116454	1.33	0.005912
	Cyp19a1	A_52_P591961	NM_007810	cytochrome P450, family 19, subfamily a, polypeptide 1	1.00	1	1.00	1	1.00	1	1.00	1
	Cyp1a2	A_51_P450140	NM_009993	cytochrome P450, family 1, subfamily a, polypeptide 2	1.31	0.137706	0.82	0.162217	1.38	0.287854	1.12	0.397882
	Cyp1a2	A_52_P595871	NM_009993	cytochrome P450, family 1, subfamily a, polypeptide 2	0.99	0.890036	0.95	0.712519	1.00	1	0.95	0.777477
	Cyp1b1	A_52_P297822	AK016699	cytochrome P450, family 1, subfamily b, polypeptide 1	1.00	0.984492	1.30	0.277073	1.00	0.976492	1.36	0.273099
	Cyp2a12	A_51_P206736	NM_133657	cytochrome P450, family 2, subfamily a, polypeptide 12	1.00	1	1.00	1	1.00	1	1.00	1
	Cyp2a5	A_52_P246252	NM_007812	cytochrome P450, family 2, subfamily a, polypeptide 5	1.10	0.543826	1.06	0.773843	0.95	0.758716	0.79	0.518717
	Cyp2b10	A_51_P182362	NM_009998	Mus musculus cytochrome P450, family 2, subfamily b, polypeptide 10 (Cyp2b10), transcript variant 2, mRNA.	0.89	0.347002	1.19	0.287759	1.19	0.351252	1.36	0.001351

	Cyp2b10	A_52_P472486	NM_009999	cytochrome P450, family 2, subfamily b, polypeptide 10	0.98	0.84385	0.98	0.879554	1.07	0.561755	1.01	0.923178
	Cyp2b13	A_51_P492339	NM_007813	cytochrome P450, family 2, subfamily b, polypeptide 13	0.94	0.798648	0.94	0.613588	0.98	0.917	0.80	0.256791
	Cyp2b13	A_52_P289091	NM_007813	cytochrome P450, family 2, subfamily b, polypeptide 13	1.20	0.023833	1.03	0.660369	0.90	0.23214	1.03	0.612147
	Cyp2b19	A_51_P352763	NM_007814	cytochrome P450, family 2, subfamily b, polypeptide 19	1.18	2.23E-06	1.15	0.013364	1.11	0.010258	1.15	0.000106
	Cyp2b19	A_52_P421626	AK029227	cytochrome P450, family 2, subfamily b, polypeptide 19	1.00	1	1.00	1	1.00	1	1.00	1
	Cyp2c29	A_51_P103706	NM_007815	cytochrome P450, family 2, subfamily c, polypeptide 29	1.04	0.738679	0.94	0.503851	1.03	0.908494	1.33	0.033906
	Cyp2c38	A_51_P342206	NM_010002	cytochrome P450, family 2, subfamily c, polypeptide 38	1.00	1	1.00	1	1.00	1	1.00	1
	Cyp2c38	A_52_P468564	NM_010002	cytochrome P450, family 2, subfamily c, polypeptide 38	1.00	1	1.00	1	1.00	1	1.05	0.62758
	Cyp2c39	A_51_P304109	NM_010003	cytochrome P450, family 2, subfamily c, polypeptide 39	1.00	1	1.00	1	1.00	1	1.00	1
	Cyp2c44	A_51_P209782	NM_001001446	cytochrome P450, family 2, subfamily c, polypeptide 44	0.77	0.207521	1.14	0.52184	1.07	0.807855	1.15	0.373625
	Cyp2c54	A_52_P154580	NM_206537	cytochrome P450, family 2, subfamily c, polypeptide 54	0.95	0.558826	1.11	0.527632	1.10	0.4169	1.08	0.676863
	Cyp2c55	A_51_P447785	NM_028089	cytochrome P450, family 2, subfamily c, polypeptide 55	0.95	0.324697	0.87	0.001004	0.88	0.010709	0.94	0.023592
	Cyp2c65	A_52_P652059	AK008688	cytochrome P450, family 2, subfamily c, polypeptide 65	0.85	0.010483	1.04	0.63575	1.11	0.040806	0.99	0.866459
	Cyp2c66	A_51_P471126	NM_001011707	cytochrome P450, family 2, subfamily c, polypeptide 66	1.12	0.3592	1.43	0.00157	1.09	0.467904	1.28	0.023425
	Cyp2c70	A_51_P134142	NM_145499	cytochrome P450, family 2, subfamily c, polypeptide 70	1.01	0.696792	0.95	0.146067	0.94	0.038226	0.97	0.183637
	Cyp2d10	A_51_P111192	NM_010005	cytochrome P450, family 2, subfamily d, polypeptide 10	1.03	0.40488	1.12	3.64E-06	1.03	0.41001	0.99	0.79725
	Cyp2d10	A_52_P527775	NM_010005	cytochrome P450, family 2, subfamily d, polypeptide 10	1.06	0.112615	1.02	0.580152	1.08	0.121467	1.07	0.128513
	Cyp2d13	A_52_P372062	NM_133695	Mus musculus cytochrome P450, family 2, subfamily d, polypeptide 13 (Cyp2d13) on chromosome 15.	0.99	0.963699	0.93	0.768373	0.99	0.960795	1.24	0.363521
	Cyp2d22	A_51_P124126	NM_019823	cytochrome P450, family 2, subfamily d, polypeptide 22	0.95	0.412927	1.08	0.028459	0.94	0.436326	0.93	0.191909
	Cyp2d22	A_52_P478420	AK086555	cytochrome P450, family 2, subfamily d, polypeptide 22	1.07	0.125863	1.15	0.00285	1.06	0.18605	0.90	0.028834
	Cyp2d22	A_52_P49391	AK090296	cytochrome P450, family 2, subfamily d, polypeptide 22	0.84	0.000546	0.87	0.000756	0.90	0.040986	0.84	0.000016
	Cyp2d9	A_51_P124133	NM_010006	cytochrome P450, family 2, subfamily d, polypeptide 9	1.00	0.965657	1.03	0.176462	1.06	0.036487	1.00	0.994884
	Cyp2d9	A_51_P478303	NM_010006	cytochrome P450, family 2, subfamily d, polypeptide 9	1.25	1.19E-12	1.18	1.69E-10	1.14	2.90E-13	1.09	2.15E-06
	Cyp2g1	A_51_P137452	NM_013809	cytochrome P450, family 2, subfamily g, polypeptide 1	1.24	0.233244	1.10	0.678732	1.01	0.968408	1.07	0.605405
	Cyp2g1	A_52_P481346	NM_013809	cytochrome P450, family 2, subfamily g, polypeptide 1	1.00	1	1.00	1	1.00	1	1.10	0.469839
	Cyp2j5	A_51_P307872	NM_010007	cytochrome P450, family 2, subfamily j, polypeptide 5	0.99	0.922366	1.12	0.641582	1.00	1	1.00	1
	Cyp2j5	A_52_P315766	NM_010007	cytochrome P450, family 2, subfamily j, polypeptide 5	1.07	0.577594	1.02	0.869785	1.00	1	1.00	1
	Cyp2j9	A_51_P330044	NM_028979	cytochrome P450, family 2, subfamily j, polypeptide 9	0.96	0.147853	0.92	0.060985	0.88	0.000999	0.96	0.292933
	Cyp3a13	A_51_P114941	NM_007819	cytochrome P450, family 3, subfamily a, polypeptide 13	0.84	0.275835	0.84	0.379511	0.62	0.013203	0.84	0.23384
	Cyp3a16	A_51_P482051	NM_007820	cytochrome P450, family 3, subfamily a, polypeptide 16	0.93	0.754141	1.49	0.232588	1.06	0.777923	0.89	0.494197
	Cyp3a25	A_51_P489367	NM_019792	cytochrome P450, family 3, subfamily a, polypeptide 25	0.97	0.7486	1.00	1	1.00	1	1.00	1
	Cyp3a25	A_52_P380314	NM_019792	cytochrome P450, family 3, subfamily a, polypeptide 25	1.00	1	1.00	1	1.03	0.650984	1.00	1
	Cyp3a44	A_52_P366803	NM_177380	cytochrome P450, family 3, subfamily a, polypeptide 44	1.00	1	1.00	1	1.00	1	1.00	1
	Cyp3a44	A_52_P571006	NM_177380	cytochrome P450, family 3, subfamily a, polypeptide 44	1.00	1	1.00	1	1.00	1	1.00	1

	Cyp4a10	A_52_P257774	NM_010011	cytochrome P450, family 4, subfamily a, polypeptide 10	1.04	0.818078	1.00	1	1.00	1	0.90	0.448251
	Cyp4a10	A_52_P340136	X71478	cytochrome P450, family 4, subfamily a, polypeptide 10	0.90	0.197337	1.19	0.117584	1.11	0.047816	1.15	0.016387
	Cyp4a10	A_52_P6588	NM_010011	cytochrome P450, family 4, subfamily a, polypeptide 10	0.94	0.503	1.19	0.335823	1.21	0.450878	0.84	0.590592
	Cyp4a12a	A_51_P433360	NM_177406	cytochrome P450, family 4, subfamily a, polypeptide 12a	0.91	0.58028	1.18	0.642361	1.21	0.335134	1.27	0.309903
	Cyp4a14	A_51_P238576	NM_007822	cytochrome P450, family 4, subfamily a, polypeptide 14	1.04	0.380304	0.99	0.844162	1.05	0.669173	1.04	0.256985
	Cyp4f14	A_51_P452768	NM_022434	cytochrome P450, family 4, subfamily f, polypeptide 14	1.11	0.496822	0.89	0.641397	1.26	0.305074	1.01	0.926879
	Cyp4f15	A_51_P136303	NM_134127	cytochrome P450, family 4, subfamily f, polypeptide 15	1.00	1	1.00	1	1.00	1	1.00	1
	Cyp51	A_51_P485791	NM_020010	cytochrome P450, family 51	0.76	0.014647	0.93	0.30741	0.95	0.531057	1.13	0.088029
	Cyp51	A_52_P778750	AK035338	Mus musculus adult male urinary bladder cDNA, RIKEN full-length enriched library, clone:9530019F09	1.00	1	1.00	1	1.00	1	1.00	1
	Dci	A_51_P105589	NM_010023	dodecenoyl-Coenzyme A delta isomerase (3,2 trans-enoyl-Coenyme A isomerase)	1.01	0.749644	1.18	0.001632	1.10	0.000564	0.96	0.129832
	Decr1	A_51_P208555	NM_026172	2,4-dienoyl CoA reductase 1, mitochondrial	0.92	0.136953	0.86	0.004363	0.83	0.002527	0.78	4.49E-06
	Decr1	A_52_P335907	NM_026172	2,4-dienoyl CoA reductase 1, mitochondrial	0.93	0.07435	0.88	0.000706	0.87	3.50E-06	0.83	1.20E-21
	Dgat2	A_51_P396003	NM_026384	diacylglycerol O-acyltransferase 2	0.96	0.551136	1.21	0.000067	1.17	0.000962	1.28	6.42E-10
	Dgat2	A_52_P592909	NM_026384	diacylglycerol O-acyltransferase 2	0.94	0.309034	1.12	0.007574	1.09	0.014086	1.24	4.21E-15
	Dlat	A_52_P374642	NM_145614	dihydrolipoamide S-acetyltransferase (E2 component of pyruvate dehydrogenase complex)	1.08	0.002847	0.90	0.000011	0.97	0.2901	0.96	0.094663
	Dld	A_51_P184282	U73445	dihydrolipoamide dehydrogenase	1.07	0.014602	0.88	0.000048	0.91	0.000495	0.94	0.00069
	Dld	A_52_P536646	NM_007861	dihydrolipoamide dehydrogenase	0.92	0.141547	0.79	0.011496	0.84	0.014865	0.86	0.01186
	Dlst	A_52_P140072	NM_030225	dihydrolipoamide S-succinyltransferase (E2 component of 2-oxo-glutarate complex)	1.17	0.028261	1.07	0.360744	1.09	0.198018	1.15	0.042536
	Dlst	A_52_P3383	AK171100	dihydrolipoamide S-succinyltransferase (E2 component of 2-oxo-glutarate complex)	1.07	0.028959	0.86	0.000755	0.85	0.000194	1.05	0.156966
	Ech1	A_51_P421846	NM_016772	enoyl coenzyme A hydratase 1, peroxisomal	0.97	0.452515	1.21	0.001107	1.22	2.82E-15	1.09	0.000419
	Echs1	A_51_P409039	NM_053119	enoyl Coenzyme A hydratase, short chain, 1, mitochondrial	0.93	0.000959	0.87	7.62E-07	0.80	1.13E-35	0.84	9.23E-26
	Ehhadh	A_51_P462918	NM_023737	enoyl-Coenzyme A, hydratase/3-hydroxyacyl Coenzyme A dehydrogenase	0.89	0.000136	0.84	3.02E-06	0.86	0.000055	1.12	0.001829
	Eno1	A_52_P349939	NM_023119	enolase 1, alpha non-neuron	1.14	0.146494	1.00	0.97817	1.11	0.148264	1.10	0.223193
	Fabp1	A_51_P487818	NM_017399	fatty acid binding protein 1, liver	0.88	0.281956	0.74	0.090117	0.83	0.303685	0.88	0.264696
	Fabp2	A_51_P313581	NM_007980	fatty acid binding protein 2, intestinal	1.12	0.002604	1.08	0.148284	1.04	0.437777	1.13	0.000022
	Fabp2	A_52_P453013	NM_007980	fatty acid binding protein 2, intestinal	0.66	0.008903	1.15	0.690694	1.08	0.621853	1.15	0.311106
	Fabp3	A_51_P167535	NM_010174	fatty acid binding protein 3, muscle and heart	0.85	0.303578	1.11	0.488699	1.09	0.557694	0.91	0.583787
	Fbp1	A_51_P474701	NM_019395	fructose bisphosphatase 1	0.92	0.684408	0.96	0.805518	0.95	0.799475	0.75	0.045375
	Fdft1	A_51_P485946	NM_010191	farnesyl diphosphate farnesyl transferase 1	1.15	2.36E-11	1.17	8.16E-11	1.11	8.22E-08	1.15	9.09E-09
	Fdft1	A_52_P136138	NM_010191	farnesyl diphosphate farnesyl transferase 1	1.23	0.003257	0.91	0.480531	1.17	0.282651	1.16	0.210927
	Fdps	A_51_P379798	NM_134469	farnesyl diphosphate synthetase	0.83	9.31E-15	1.10	0.018792	1.16	8.87E-06	1.17	5.14E-09
	Fdps	A_52_P593965	AK077979	farnesyl diphosphate synthetase	0.89	0.003054	0.97	0.50233	0.94	0.283164	1.13	0.0768

Fnta	A_51_P112116	NM_008033	farnesyltransferase, CAAX box, alpha	1.08	0.000385	1.02	0.228188	1.12	0.000031	1.10	0.000018
Fntb	A_51_P517680	NM_145927	farnesyltransferase, CAAX box, beta	0.97	0.365488	1.00	0.986099	1.03	0.547523	1.06	0.09753
Fntb	A_52_P235241	NM_145927	farnesyltransferase, CAAX box, beta	0.84	7.06E-09	0.97	0.463486	1.00	0.985179	1.00	0.930817
G6pc	A_51_P462385	NM_008061	glucose-6-phosphatase, catalytic	1.14	0.000183	1.21	0.001319	1.08	0.106185	1.14	0.000322
Gck	A_52_P66580	BC011139	glucokinase	1.10	0.061698	0.97	0.42253	1.11	0.029489	1.11	0.000289
Gckr	A_51_P198694	NM_144909	glucokinase regulatory protein	0.96	0.760948	1.05	0.774406	1.17	0.297766	1.47	0.001433
Ggps1	A_51_P179604	NM_010282	PREDICTED: Mus musculus geranylgeranyl diphosphate synthase 1, transcript variant 4 (Ggps1), mRNA.	0.89	0.010082	0.93	0.12768	0.85	0.000595	0.90	0.012398
Ggps1	A_51_P469902	XM_977926	geranylgeranyl diphosphate synthase 1	1.04	0.198474	0.89	0.002363	0.88	0.000012	0.87	9.86E-10
Gpi1	A_51_P426886	NM_008155	glucose phosphate isomerase 1	0.96	0.065756	1.00	0.959669	1.03	0.047054	1.06	0.017767
Gpi1	A_52_P347412	L09104	glucose phosphate isomerase 1	1.01	0.705449	1.07	0.085853	1.10	0.061533	1.07	0.105344
Gyk	A_52_P379205	AK086200	glycerol kinase	1.12	0.522919	1.00	1	1.00	1	1.00	1
Gyk	A_52_P453517	NM_008194	glycerol kinase	1.00	0.974152	1.21	0.486486	1.14	0.64433	0.97	0.823924
Gys1	A_52_P228932	NM_030678	glycogen synthase 1, muscle	0.95	0.292576	0.98	0.65637	1.01	0.815913	0.99	0.875561
Hadha	A_52_P338459	AK035316	hydroxyacyl-Coenzyme A dehydrogenase/3-ketoacyl-Coenzyme A thiolase/enoyl-Coenzyme A hydratase, alpha subunit	1.64	0.03785	1.25	0.250483	0.94	0.597202	1.04	0.792
Hadha	A_52_P658044	AK050856	hydroxyacyl-Coenzyme A dehydrogenase/3-ketoacyl-Coenzyme A thiolase/enoyl-Coenzyme A hydratase, alpha subunit	0.81	0.144005	0.85	0.129918	0.84	0.091232	0.85	0.124467
Hadhb	A_51_P217990	NM_145558	hydroxyacyl-Coenzyme A dehydrogenase/3-ketoacyl-Coenzyme A thiolase/enoyl-Coenzyme A hydratase, beta subunit	1.23	3.30E-11	1.11	0.030362	1.08	0.01388	0.92	0.000161
Hk1	A_52_P102773	BC072628	hexokinase 1	1.04	0.301878	1.20	2.73E-12	1.16	2.51E-11	1.19	2.62E-18
Hk1	A_52_P479599	NM_010438	hexokinase 1	0.79	0.039717	0.94	0.507839	0.97	0.799103	1.05	0.437709
Hk1	A_52_P674530	NM_010438	hexokinase 1	0.93	0.042922	1.07	0.20426	1.11	0.000139	1.16	1.85E-07
Hk1	A_52_P87141	NM_010438	hexokinase 1	1.38	0.176002	1.10	0.662841	0.94	0.653176	1.10	0.544626
Hmgcl	A_51_P308961	NM_008254	3-hydroxy-3-methylglutaryl-Coenzyme A lyase	1.20	9.99E-12	1.13	7.85E-06	1.16	9.01E-13	1.08	0.00259
Hmgcll1	A_52_P197722	NM_173731	3-hydroxymethyl-3-methylglutaryl-Coenzyme A lyase-like 1	1.26	0.025439	1.02	0.880237	0.95	0.572675	0.85	0.094914
Hmgcr	A_52_P578922	AK159899	3-hydroxy-3-methylglutaryl-Coenzyme A reductase	0.92	0.029724	0.91	4.99E-07	0.92	0.000074	0.98	0.356645
Hmgcr	A_52_P770765	AK033165	Mus musculus 15 days embryo male testis cDNA, RIKEN full-length enriched library, clone:8030450I20	0.77	0.071924	0.84	0.433231	0.89	0.365471	0.90	0.318843
Hmgcs1	A_52_P119039	AK078743	3-hydroxy-3-methylglutaryl-Coenzyme A synthase 1	0.81	0.260819	0.90	0.614731	0.89	0.438221	0.78	0.029329
Hmgcs2	A_51_P116039	NM_008256	3-hydroxy-3-methylglutaryl-Coenzyme A synthase 2	1.03	0.900147	0.99	0.980713	0.96	0.807873	0.91	0.694633
Hsd17b4	A_51_P445662	NM_008292	hydroxysteroid (17-beta) dehydrogenase 4	1.11	3.30E-07	1.08	0.000519	1.10	1.93E-10	1.20	1.58E-20
Idh3a	A_51_P268559	NM_029573	isocitrate dehydrogenase 3 (NAD+) alpha	1.20	0.000315	0.78	2.56E-06	0.89	0.03488	0.98	0.635659
Idi1	A_51_P329711	NM_177960	isopentenyl-diphosphate delta isomerase	0.87	0.00062	1.10	0.110183	0.92	0.010864	0.92	0.010283
Ldhc	A_51_P242403	NM_013580	lactate dehydrogenase C	1.00	1	1.00	1	1.00	1	1.00	1
Lipe	A_51_P435366	NM_010719	lipase, hormone sensitive	0.90	0.005121	0.96	0.182903	0.92	0.004157	1.17	3.60E-06

	Lpl	A_51_P259296	NM_008509	lipoprotein lipase	0.90	0.342969	0.88	0.303114	0.79	0.075399	0.94	0.664394
	Mcat	A_51_P314855	NM_001030014	malonyl CoA:ACP acyltransferase (mitochondrial)	1.06	0.130558	1.07	0.060024	1.11	0.010059	1.17	0.000041
	Mdh1	A_51_P218179	NM_008618	malate dehydrogenase 1, NAD (soluble)	1.18	7.06E-15	1.12	0.000283	1.24	5.14E-15	1.24	2.18E-18
	Mdh1	A_52_P407796	NM_008618	malate dehydrogenase 1, NAD (soluble)	1.13	0.038715	0.96	0.585048	1.03	0.571308	1.07	0.355067
	Mdh1	A_52_P470451	AK077724	malate dehydrogenase 1, NAD (soluble)	1.12	0.026756	0.79	0.000021	0.79	2.19E-11	0.79	6.68E-10
	Mdh2	A_51_P384879	NM_008617	malate dehydrogenase 2, NAD (mitochondrial)	1.25	0.029863	0.91	0.342023	1.03	0.74713	1.07	0.378048
	Mecr	A_51_P386080	NM_025297	mitochondrial trans-2-enoyl-CoA reductase	1.06	0.03343	1.10	0.015291	1.12	5.45E-06	1.21	1.37E-13
	Mgll	A_51_P352549	AK079089	Mus musculus adult male diencephalon cDNA, RIKEN full-length enriched library, clone:9330168D17	1.00	1	1.00	1	1.00	1	1.00	1
	Mut	A_51_P193935	NM_008650	methylmalonyl-Coenzyme A mutase	1.10	0.026066	0.90	0.028543	0.85	0.008602	0.87	0.012252
	Mut	A_52_P362959	AK051851	methylmalonyl-Coenzyme A mutase	1.00	1	1.00	1	1.00	1	1.00	1
	Mut	A_52_P633163	NM_008650	methylmalonyl-Coenzyme A mutase	1.07	0.008433	0.90	0.002526	0.87	2.52E-06	0.90	0.000023
	Ogdh	A_51_P122825	NM_010956	oxoglutarate dehydrogenase (lipoamide)	1.16	0.000015	1.05	0.102776	1.11	0.006447	1.05	0.109291
	Ogdh	A_52_P369310	NM_010956	oxoglutarate dehydrogenase (lipoamide)	1.10	0.05833	1.03	0.502506	1.05	0.242531	1.02	0.688472
	Oxct2a	A_51_P125056	NM_022033	3-oxoacid CoA transferase 2A	1.36	0.217335	1.08	0.506497	1.03	0.815404	1.38	0.126361
	Oxsm	A_51_P312748	NM_027695	3-oxoacyl-ACP synthase, mitochondrial	0.99	0.866235	0.89	0.090421	0.90	0.044034	0.93	0.184463
	Oxsm	A_52_P231635	NM_027695	3-oxoacyl-ACP synthase, mitochondrial	1.21	3.92E-08	1.06	0.081873	1.17	8.55E-07	1.22	8.86E-23
	Pccb	A_51_P418259	NM_025835	propionyl Coenzyme A carboxylase, beta polypeptide	1.16	0.000048	0.92	0.044418	0.98	0.484509	0.98	0.485443
	Pck2	A_52_P21659	NM_028994	phosphoenolpyruvate carboxykinase 2 (mitochondrial)	1.02	0.518626	1.17	0.000433	1.11	3.68E-06	1.21	1.72E-20
	Pcx	A_51_P480578	NM_008797	pyruvate carboxylase	1.03	0.430291	0.99	0.826808	0.97	0.24447	1.16	0.000265
	Pdha1	A_51_P321391	NM_008810	pyruvate dehydrogenase E1 alpha 1	1.06	0.33274	0.89	0.017833	0.92	0.033921	0.93	0.087083
	Pdha1	A_52_P581435	NM_008810	pyruvate dehydrogenase E1 alpha 1	0.99	0.783287	0.83	9.88E-14	0.84	6.61E-07	0.85	1.78E-21
	Pdha2	A_51_P519857	NM_008811	pyruvate dehydrogenase E1 alpha 2	1.00	1	1.00	1	1.00	1	1.00	1
	Pdhb	A_51_P321921	NM_024221	pyruvate dehydrogenase (lipoamide) beta	1.07	0.006053	1.01	0.554244	1.10	0.000206	1.12	3.63E-13
	Pdhb	A_52_P568792	NM_024221	pyruvate dehydrogenase (lipoamide) beta	0.95	0.066615	0.85	4.11E-10	0.94	0.053274	0.99	0.577593
	Peci	A_51_P394665	NM_011868	peroxisomal delta3, delta2-enoyl-Coenzyme A isomerase	0.94	0.005073	1.04	0.081897	1.02	0.170276	1.01	0.629259
	Pfkfb2	A_51_P184849	NM_008825	6-phosphofructo-2-kinase/fructose-2,6-biphosphatase 2	1.16	8.95E-07	0.97	0.461637	1.03	0.186533	0.99	0.851213
	Pfkfb2	A_51_P335251	NM_008825	6-phosphofructo-2-kinase/fructose-2,6-biphosphatase 2	1.05	0.458276	0.90	0.040198	0.95	0.251436	0.80	0.000621
	Pfkfb2	A_52_P142912	NM_008825	6-phosphofructo-2-kinase/fructose-2,6-biphosphatase 2	1.11	0.004998	0.80	0.000813	0.95	0.319062	0.91	0.138245
	Pfkfb2	A_52_P31125	BC018418	6-phosphofructo-2-kinase/fructose-2,6-biphosphatase 2	1.35	0.001507	1.41	0.013794	1.21	0.291739	1.11	0.342817
	Pfkl	A_51_P310896	NM_008826	phosphofructokinase, liver, B-type	0.98	0.734219	1.18	0.009374	1.26	0.001513	1.23	0.000943
	Pgam1	A_51_P342669	NM_023418	phosphoglycerate mutase 1	0.80	2.23E-07	0.84	2.43E-06	0.83	0.000017	1.02	0.612479
	Pgk1	A_51_P182828	NM_008828	phosphoglycerate kinase 1	1.28	6.20E-15	1.02	0.526348	1.03	0.193371	1.07	0.000067
	Pgk1	A_52_P600398	NM_008828	phosphoglycerate kinase 1	1.25	3.55E-08	0.98	0.580435	1.03	0.372264	1.07	0.003277
	Pgls	A_51_P321794	NM_025396	6-phosphogluconolactonase	1.15	3.38E-08	1.20	1.33E-12	1.20	1.45E-23	1.21	1.10E-42

	Pgm3	A_51_P208987	NM_028352	phosphoglucomutase 3	1.07	0.278793	0.87	0.058821	0.96	0.592948	1.01	0.806057
	Pgm3	A_52_P341449	NM_028352	phosphoglucomutase 3	0.79	4.84E-09	0.99	0.827123	0.89	0.022728	0.93	0.059139
	Pgm5	A_52_P229924	NM_175013	phosphoglucomutase 5	1.20	0.028011	1.17	0.037112	1.14	0.064824	1.25	0.004129
	Pgm5	A_52_P860000	NM_175013	phosphoglucomutase 5	1.14	0.614435	1.10	0.258207	1.25	0.256178	0.98	0.93904
	Phka2	A_51_P239970	AK051899	phosphorylase kinase alpha 2	1.00	1	1.05	0.748184	1.00	1	1.00	1
	Phka2	A_51_P368950	AK018208	Mus musculus 12 days embryo eyeball cDNA, RIKEN full-length enriched library, clone:D230017A12	0.82	0.155074	0.89	0.522746	1.11	0.64997	0.87	0.136028
	Phka2	A_52_P657963	AK045618	phosphorylase kinase alpha 2	0.87	0.014459	1.11	0.046053	0.93	0.103166	0.82	1.50E-13
	Phkb	A_51_P270725	AK089493	phosphorylase kinase beta	1.18	0.001757	0.91	0.018839	0.99	0.64281	0.94	0.03896
	Phkb	A_52_P342907	BC051503	phosphorylase kinase beta	1.10	0.528878	0.92	0.562882	1.00	1	1.04	0.718673
	Phkb	A_52_P513347	NM_199446	phosphorylase kinase beta	1.16	0.015466	0.69	1.18E-07	0.80	0.000062	0.77	5.98E-14
	Phkg2	A_51_P235909	NM_026888	phosphorylase kinase, gamma 2 (testis)	1.17	0.000016	1.18	0.000019	1.19	1.08E-07	1.17	7.02E-06
	Phkg2	A_52_P19446	AK089062	phosphorylase kinase, gamma 2 (testis)	0.76	0.242253	0.92	0.60291	0.92	0.602133	0.89	0.385471
	Pkm2	A_52_P328249	AK083076	pyruvate kinase, muscle	1.10	0.004928	1.03	0.408358	1.14	0.001676	1.02	0.539087
	Ppara	A_51_P348334	NM_011144	peroxisome proliferator activated receptor alpha	0.98	0.489239	0.99	0.899554	1.00	0.891679	0.96	0.098172
	Ppard	A_51_P271556	NM_011145	peroxisome proliferator activator receptor delta	0.94	0.291546	1.15	0.005179	1.13	0.136575	1.05	0.434841
	Ppargc1b	A_52_P883463	AK045690	Mus musculus adult male corpora quadrigemina cDNA, RIKEN full-length enriched library, clone:B230304K20	0.81	0.000431	0.78	2.36E-14	0.78	1.35E-31	0.81	7.63E-09
	Ppp1ca	A_51_P509389	NM_031868	protein phosphatase 1, catalytic subunit, alpha isoform	0.95	0.114105	1.07	0.117592	1.06	0.034744	1.10	0.000596
	Ppp1cc	A_52_P519508	NM_013636	protein phosphatase 1, catalytic subunit, gamma isoform	1.05	0.114533	0.92	0.037383	0.96	0.174804	0.95	0.045847
	Ppp1cc	A_52_P540434	NM_013636	protein phosphatase 1, catalytic subunit, gamma isoform	1.08	0.021697	0.94	0.013063	0.94	0.049344	0.96	0.059605
	Ppt2	A_52_P238816	NM_019441	palmitoyl-protein thioesterase 2	1.09	0.000535	1.07	0.036481	1.05	0.006852	1.03	0.128012
	Pygb	A_51_P485810	NM_153781	brain glycogen phosphorylase	0.80	0.095792	1.16	0.227436	1.29	0.016167	1.30	0.008205
	Pygl	A_52_P320181	AK083075	liver glycogen phosphorylase	1.00	1	1.00	1	0.98	0.871164	1.00	1
	Scd3	A_51_P446045	NM_024450	stearoyl-coenzyme A desaturase 3	1.01	0.886387	1.16	0.000094	1.06	0.16702	1.09	0.06064
	Scd4	A_52_P374877	NM_183216	stearoyl-coenzyme A desaturase 4	1.00	1	1.00	1	1.02	0.907247	1.16	0.44379
	Scd4	A_52_P603237	BC038322	stearoyl-coenzyme A desaturase 4	1.00	1	0.96	0.764418	0.97	0.702267	1.26	0.127393
	Sdha	A_52_P109503	NM_023281	succinate dehydrogenase complex, subunit A, flavoprotein (Fp)	1.10	0.089656	0.97	0.580809	0.95	0.250442	0.94	0.218484
	Sdhb	A_51_P234853	NM_023374	succinate dehydrogenase complex, subunit B, iron sulfur (Ip)	1.29	5.32E-20	1.01	0.828356	1.04	0.265271	1.02	0.502067
	Sdhc	A_51_P106373	NM_025321	succinate dehydrogenase complex, subunit C, integral membrane protein	1.26	3.07E-06	1.17	0.009143	1.15	0.005156	1.04	0.393295
	Sdhd	A_51_P260871	NM_025848	succinate dehydrogenase complex, subunit D, integral membrane protein	1.19	0.000011	1.04	0.27651	1.06	0.156084	1.02	0.661669
	Slc27a1	A_52_P199905	CB588406	AGENCOURT_12600125 NIH_MGC_136 Mus musculus cDNA clone IMAGE:30290747 5', mRNA sequence.	1.02	0.500223	0.87	0.011824	0.93	0.216123	1.18	9.55E-10
	Slc27a2	A_51_P484551	NM_011978	solute carrier family 27 (fatty acid transporter), member 2	1.02	0.858915	1.02	0.792639	1.00	0.969122	0.86	0.497094
	Slc27a2	A_52_P1083677	AK038847	solute carrier family 27 (fatty acid transporter), member 2	1.00	1	1.00	1	1.00	1	1.00	1

	Slc27a2	A_52_P154971	NM_011978	solute carrier family 27 (fatty acid transporter), member 2	1.19	0.085735	1.09	0.687762	0.81	0.327119	1.24	0.279449
	Slc27a4	A_51_P408609	NM_011989	solute carrier family 27 (fatty acid transporter), member 4	1.00	0.991729	0.81	0.010115	1.00	0.97244	1.22	0.048962
	Slc27a4	A_52_P61854	NM_011989	solute carrier family 27 (fatty acid transporter), member 4	1.05	0.32337	1.14	0.010642	1.22	0.000104	1.27	0.000109
	Slc27a5	A_51_P289742	NM_009512	solute carrier family 27 (fatty acid transporter), member 5	0.92	0.352201	0.99	0.930106	1.20	0.137374	0.99	0.937775
	Slc27a6	A_51_P211616	AK028699	solute carrier family 27 (fatty acid transporter), member 6	1.12	0.468952	1.00	1	1.00	1	1.42	0.152761
	Slc2a2	A_51_P185693	NM_031197	solute carrier family 2 (facilitated glucose transporter), member 2	1.00	1	1.06	0.660862	1.00	1	1.19	0.474252
	Slc2a2	A_52_P409988	NM_031197	solute carrier family 2 (facilitated glucose transporter), member 2	1.18	0.006788	0.95	0.521785	1.16	0.014602	1.11	0.027911
	Slc2a4	A_51_P217498	NM_009204	solute carrier family 2 (facilitated glucose transporter), member 4	0.86	1.21E-15	0.85	1.42E-07	0.96	0.003481	0.98	0.245546
	Sqle	A_51_P450487	NM_009270	squalene epoxidase	0.89	0.043316	1.35	0.020212	1.13	0.031236	1.20	0.001692
	Sucla2	A_52_P533792	NM_011506	succinate-Coenzyme A ligase, ADP-forming, beta subunit	1.28	1.24E-16	0.93	0.009098	1.05	0.170076	1.01	0.892332
	Suclg2	A_51_P478672	NM_011507	succinate-Coenzyme A ligase, GDP-forming, beta subunit	1.15	0.000285	0.98	0.75009	1.02	0.767559	0.81	0.001085
	Suclg2	A_52_P58181	NM_011507	succinate-Coenzyme A ligase, GDP-forming, beta subunit	1.01	0.758032	0.90	0.000253	0.94	0.002485	0.92	1.80E-07
	Tkt	A_52_P631197	AK050793	transketolase	1.04	0.846415	1.08	0.73405	0.80	0.320987	1.19	0.416033
	Ucp2	A_52_P499675	NM_011671	uncoupling protein 2 (mitochondrial, proton carrier)	1.60	0.234356	1.97	0.003958	1.31	0.475073	1.39	0.325886
	Ugp2	A_51_P475580	NM_139297	UDP-glucose pyrophosphorylase 2	1.08	0.002661	0.89	0.009003	0.98	0.678936	0.97	0.201846
	Uqcrc2	A_51_P128648	NM_025899	ubiquinol cytochrome c reductase core protein 2	1.25	1.64E-31	1.11	0.000045	1.12	6.12E-06	1.07	4.80E-06
	Uqcrfs1	A_51_P361951	NM_025710	ubiquinol-cytochrome c reductase, Rieske iron-sulfur polypeptide 1	1.27	0	0.97	0.303646	0.99	0.806039	0.98	0.162563
	Uqcrh	A_52_P541875	NM_025641	ubiquinol-cytochrome c reductase hinge protein	1.29	0.000072	1.03	0.604336	1.00	0.937126	0.95	0.328395
	Uqcrq	A_52_P370484	NM_025352	ubiquinol-cytochrome c reductase, complex III subunit VII	1.28	0.008964	1.05	0.553213	1	0.93704	0.99	0.927733

Supplemental Table 3: **Liver and IAT real-time PCR data analysis and comparison with corresponding microarrays results.** Data are presented as ratio of db/$^+$ or RSG-treated mice versus db/db mice.

	qPCR Ratio *vs.* db/db						Microarrays Ratio *vs.* db/db					
**Sample LIVER**	**Assay ID**	**Pool dbdb**	**db+**	**RSG 10MK**	**RSG 30MK**	**RSG 100MK**	**Sequence code**	**Pool dbdb**	**db+**	**RSG 10MK**	**RSG 30MK**	**RSG 100MK**
Acaa1a	Mm00728460_s1	1	0,71	1.99	1.90	1.79	A_52_P423183	1	0.54	3.76	3.85	3.62
Acaa1b	Mm00728805_s1	1	0.44	4.05	3.68	3.45	A_52_P423174	1	0.57	3.51	3.42	3.05
Acaca	Mm01304285_m1	1	0.63	3.74	3.53	2.74	A_52_P595124	1	0.88	1.76	2.55	1.90
Acacb	Mm01204683_m1	1	0.46	2.28	2.22	2.01	A_52_P340167	1	0.72	2.05	2.27	1.77
Acad10	Mm00660975_m1	1	0.70	1.45	1.54	1.42	A_51_P187507	1	0.84	1.46	1.51	1.49
Acad11	Mm00614545_m1	1	0.77	1.64	1.55	1.37	A_52_P85152	1	0.75	1.47	1.39	1.30
Acadm	Mm00431611_m1	1	0.46	1.83	1.66	1.55	A_51_P319879	1	0.56	1.95	1.82	1.72
Acads	Mm00431617_m1	1	0.67	1.18	1.12	1.05	A_52_P367745	1	0.84	1.31	1.35	1.34
Acly	Mm00652520_m1	1	0.67	2.61	2.71	2.45	A_51_P261718	1	0.53	1.97	2.38	2.22
Aco1	Mm00801417_m1	1	0.80	1.17	1.12	1.08	A_51_P391082	1	0.87	1.16	1.12	1.09
Aco2	Mm00475673_g1	1	0.70	1.44	1.35	1.18	A_52_P200359	1	0.83	1.41	1.37	1.16
Acox1	Mm00443579_m1	1	0.53	2.31	2.35	2.25	A_51_P366704	1	0.59	2.12	2.04	2.16
Acox2	Mm00446408_m1	1	0.69	1.31	1.28	1.29	A_51_P206708	1	0.76	1.38	1.37	1.40
Acsl5	Mm01261084_g1	1	0.52	1.71	1.64	1.58	A_52_P593037	1	0.68	1.81	1.80	1.79
Adh4	Mm00478838_m1	1	1.22	0.47	0.43	0.47	A_51_P189442	1	1.35	0.48	0.45	0.53
Adh7	Mm00507750_m1	1	0.78	2.10	1.90	1.83	A_51_P233797	1	0.83	1.51	1.51	1.51
Agpat2	Mm00458880_m1	1	0.46	2.32	2.22	1.87	A_51_P238565	1	0.58	2.07	1.99	1.98
Agpat6	Mm00497622_m1	1	0.62	1.43	1.46	1.26	A_51_P335710	1	0.62	1.44	1.39	1.11
Aldh1a1	Mm00657317_m1	1	0.54	0.90	0.82	0.78	A_51_P334942	1	0.55	0.81	0.81	0.76
Aldh1a3	Mm00474049_m1	1	ND	ND	ND	ND	A_52_P87843	1	0.37	0.59	0.59	0.62
Aldh1a7	Mm00496380_m1	1	0.55	1.37	1.29	1.21	A_51_P383399	1	0.61	1.36	1.21	1.27
Aldh1b1	Mm00728303_s1	1	0.46	1.19	1.20	0.98	A_51_P510418	1	0.52	1.31	1.24	1.17
Aldh2	Mm00477463_m1	1	0.90	0.82	0.79	0.79	A_52_P13109	1	0.57	0.64	0.64	0.64
Aldh3a2	Mm00839320_m1	1	0.34	2.20	1.93	1.59	A_51_P464175	1	0.34	2.12	1.95	1.71
Aldoa	Mm00833172_g1	1	1.12	1.66	1.72	1.57	A_52_P98387	1	1.30	1.54	1.49	1.43
Aldob	Mm00523296_m1	1	0.47	0.90	0.75	0.64	A_51_P337269	1	0.59	0.98	0.83	0.69
Aldoc	Mm01298116_g1	1	0.57	0.56	0.65	0.85	A_51_P220681	1	0.59	0.53	0.66	0.85
Apoa1	Mm00437569_m1	1	4.75	1.88	2.03	2.77	A_51_P408082	1	4.85	1.84	1.93	2.69
Apoa5	Mm00475480_m1	1	0.60	0.49	0.40	0.51	A_52_P292404	1	0.69	0.53	0.49	0.56
Cd36	Mm01135198_m1	1	0.07	1.83	1.76	1.58	A_51_P375146	1	0.08	1.70	1.67	1.61
Cpt1b	Mm00487200_m1	1	0.54	2.96	2.62	2.61	A_52_P625508	1	0.55	2.04	1.55	2.05
Crat	Mm00483985_m1	1	0.29	2.86	2.60	2.14	A_51_P440807	1	0.43	2.66	2.53	2.22
Cs	Mm00466043_m1	1	0.96	1.55	1.43	1.27	A_51_P390260	1	1.24	1.50	1.39	1.44
Cyp2a4;Cyp2a5	Mm00487248_g1	1	0.54	0.24	0.27	0.42	A_52_P246252	1	0.48	0.20	0.25	0.35
Cyp2b10	Mm00456588_mH	1	0.13	1.02	1.07	1.11	A_52_P472486	1	0.04	0.60	0.62	0.66
Cyp2b13	Mm00771172_g1	1	ND	0.41	0.42	0.48	A_52_P289091	1	0.06	0.51	0.45	0.49
Cyp2c37	Mm00833845_m1	1	0.53	0.14	0.15	0.32	A_51_P498882	1	0.69	0.23	0.32	0.39
Cyp2c38	Mm00658527_m1	1	0.13	0.88	0.86	0.76	A_52_P468564	1	0.12	0.72	0.70	0.64
Cyp2c39	Mm00656110_gH	1	0.06	0.74	0.71	0.72	A_51_P304109	1	0.73	0.34	0.40	0.47
Cyp2d22	Mm00530542_m1	1	0.82	0.78	0.79	0.86	A_52_P49391	1	0.76	0.68	0.68	0.73
Cyp2j9	Mm00466426_m1	1	0.55	0.47	0.48	0.55	A_51_P330044	1	0.63	0.48	0.57	0.53
Cyp3a13	Mm00484110_m1	1	0.43	0.49	0.60	0.61	A_51_P114941	1	0.49	0.56	0.64	0.67
Dci	Mm00494452_m1	1	0.68	1.37	1.24	1.13	A_51_P105589	1	0.83	1.45	1.30	1.29

Decr1	Mm00470689_m1	1	0.61	1.45	1.32	1.21	A_51_P208555	1	0.82	1.39	1.27	1.26
Dgat1	Mm01197412_g1	1	0.66	1.57	1.63	1.42	A_51_P510059	1	0.81	1.58	1.64	1.40
Dlat	Mm00455160_m1	1	0.85	1.91	1.84	1.68	A_51_P265106	1	0.88	1.92	1.91	1.75
Dlst	Mm00513470_m1	1	0.75	1.50	1.43	1.31	A_51_P290139	1	0.84	1.37	1.37	1.24
Ech1	Mm00469322_m1	1	0.56	1.31	1.22	1.12	A_51_P421846	1	0.61	1.35	1.32	1.28
Fabp2	Mm00433188_m1	1	0.54	1.44	1.13	1.03	A_51_P313581	1	0.65	1.38	1.19	1.13
Fabp3	Mm02342495_m1	1	0.83	2.14	2.60	1.68	A_51_P167535	1	0.56	2.71	2.38	1.82
Fabp4	Mm00445880_m1	1	0.43	12.58	ND	ND	A_51_P336830	1	0.52	8.96	10.73	9.87
Fabp5	Mm00783731_s1	1	ND	10.88	ND	ND	A_51_P387764	1	2.34	14.61	7.69	16.17
Fasn	Mm00662322_g1	1	0.32	3.54	3.48	3.05	A_52_P100252	1	0.33	2.59	2.99	2.89
Fbp1	Mm00490181_m1	1	0.56	0.73	0.66	0.50	A_51_P474701	1	0.50	0.73	0.64	0.52
Fdps	Mm00836315_g1	1	0.71	1.42	1.57	1.87	A_51_P379798	1	0.68	1.21	1.52	1.64
G6pc	Mm00839363_m1	1	0.37	0.61	0.55	0.36	A_51_P462385	1	0.48	0.64	0.59	0.44
Gck	Mm00439129_m1	1	0.97	1.27	1.09	1.17	A_52_P259537	1	1.06	1.28	1.17	1.19
Gckr	Mm00523328_m1	1	0.71	0.63	0.59	0.55	A_51_P198694	1	0.74	0.61	0.59	0.55
Gpd1	Mm00515846_m1	1	0.61	3.19	2.69	2.18	A_51_P293853	1	0.73	2.61	2.34	1.93
Gyk	Mm00433896_m1	1	0.91	0.92	0.80	0.79	A_51_P297671	1	0.98	1.01	0.88	0.89
Hadhb	Mm00523880_g1	1	0.82	1.32	1.27	1.09	A_51_P217990	1	0.94	1.53	1.39	1.32
Hmgcr	Mm01282499_m1	1	0.41	0.74	0.93	1.46	A_52_P578922	1	0.43	0.68	0.94	1.43
Hmgcs2	Mm00550050_m1	1	0.64	1.21	1.07	0.93	A_51_P116039	1	0.76	0.91	0.78	2.66
Hsd17b4	Mm00500443_m1	1	0.64	1.67	1.53	1.36	A_51_P445662	1	0.89	1.43	1.44	1.35
Idi1	Mm00836417_g1	1	0.89	0.88	0.96	1.24	A_52_P441634	1	0.64	0.86	1.03	1.38
Ldhb	Mm00493146_m1	1	1.26	1.82	1.88	1.80	A_51_P409173	1	1.13	1.60	1.62	1.67
Lipe	Mm00495359_m1	1	0.80	1.44	1.50	1.31	A_51_P435366	1	0.82	1.10	1.40	1.26
Lpl	Mm00434770_m1	1	1.75	4.25	6.00	6.70	A_52_P257812	1	1.62	3.33	4.22	4.97
Mdh2	Mm00725890_s1	1	0.85	1.51	1.38	1.28	A_51_P384879	1	0.94	1.43	1.34	1.33
Mgll	Mm00449274_m1	1	0.76	1.60	1.37	1.20	A_52_P568977	1	0.44	1.29	0.81	0.61
Mvd	Mm00507014_m1	1	0.40	1.36	1.24	1.55	A_51_P355943	1	0.40	1.29	1.35	1.62
Oxct1	Mm00499303_m1	1	1.29	1.49	1.38	1.32	A_51_P107321	1	1.30	1.38	1.29	1.40
Pdha1	Mm00468675_m1	1	0.75	1.45	1.39	1.24	A_51_P321391	1	0.95	1.35	1.37	1.29
Pdk4	Mm00443325_m1	1	0.31	4.26	2.92	2.50	A_51_P350453	1	0.27	3.16	2.21	2.21
Pecr	Mm00502709_m1	1	0.68	0.57	0.57	0.60	A_51_P291749	1	0.82	0.57	0.62	0.64
Pgd	Mm00503037_m1	1	0.65	2.79	2.55	2.22	A_52_P84096	1	0.67	2.16	2.21	2.10
Pgm3	Mm00459270_m1	1	0.97	1.81	1.74	1.56	A_52_P341449	1	0.96	1.37	1.45	1.35
Pklr	Mm00443090_m1	1	0.43	0.71	0.64	0.56	A_51_P176042	1	0.53	0.83	0.78	0.74
Pmvk	Mm00503429_m1	1	0.70	1.65	1.62	1.73	A_51_P492408	1	0.66	1.33	1.59	1.68
Ppara	Mm00440939_m1	1	0.69	0.99	0.91	0.99	A_51_P348334	1	0.98	1.17	1.11	1.27
Ppard	Mm00803186_g1	1	1.11	1.09	1.01	1.02	A_51_P271556	1	1.04	0.73	0.63	1.02
Pparg	Mm01258518_m1	1	0.25	1.42	1.59	1.29	A_51_P106799	1	0.41	1.28	1.36	1.23
Ppargc1a	Mm00440945_m1	1	0.55	0.51	0.51	0.53	A_51_P279038	1	0.76	0.65	0.62	0.63
Ppargc1b	Mm01208832_m1	1	1.24	0.74	0.66	0.75	A_51_P294891	1	1.29	0.81	0.78	0.89
Scd1	Mm00772290_m1	1	0.48	5.73	5.60	4.71	A_52_P682382	1	0.60	5.00	5.27	5.77
Scd2	Mm01208542_m1	1	0.44	1.32	1.51	1.60	A_51_P129464	1	0.56	1.43	2.07	2.51
Slc2a2	Mm00446224_m1	1	0.78	0.78	0.70	0.74	A_51_P185693	1	0.72	0.74	0.70	0.75
Sqle	Mm00436772_m1	1	0.60	0.93	1.02	1.34	A_51_P450487	1	0.43	1.00	1.12	1.45
Taldo1	Mm00807080_g1	1	0.74	2.28	2.13	1.80	A_51_P401958	1	0.84	2.28	2.02	1.92
Tkt	Mm00447559_m1	1	0.63	1.81	1.66	1.45	A_51_P394515	1	0.73	1.80	1.71	1.61
Ugp2	Mm00454826_m1	1	0.86	0.72	0.68	0.60	A_51_P475580	1	0.89	0.72	0.74	0.68

	qPCR Ratio *vs.* db/db						Microarrays Ratio *vs.* db/db					
**Sample IAT**	**Assay ID**	**Pool dbdb**	**db+**	**RSG 10MK**	**RSG 30MK**	**RSG 100MK**	**Sequence code**	**Pool dbdb**	**db+**	**RSG 10MK**	**RSG 30MK**	**RSG 100MK**
Acaa1a	Mm00728460_s1	1	0.83	0.96	0.99	1.18	A_52_P423183	1	1.27	1.45	2.03	2.64
Acaa1b	Mm00728805_s1	1	0.86	1.50	2.57	3.44	A_52_P423174	1	1.31	1.45	2.04	2.47
Acaa2	Mm00624282_m1	1	0.84	0.91	1.36	1.83	A_51_P125260	1	1.05	1.17	1.75	2.37
Acaca	Mm01304285_m1	1	5.10	1.96	2.92	3.11	A_52_P595124	1	5.02	1.58	2.45	2.76
Acacb	Mm01204683_m1	1	1.12	1.09	1.52	1.55	A_52_P340167	1	1.35	1.11	1.54	1.53
Acad11	Mm00614545_m1	1	0.69	0.84	0.89	1.04	A_52_P85152	1	1.06	1.00	1.34	1.53
Acad8	Mm00482266_m1	1	0.92	0.73	0.80	0.82	A_51_P244052	1	1.36	1.05	1.28	1.33
Acad9	Mm00554429_m1	1	0.68	1.07	0.99	1.24	A_51_P341379	1	1.11	1.39	1.50	1.77
Acadl	Mm00599660_m1	1	0.84	0.98	1.42	1.63	A_51_P149455	1	1.40	1.17	1.71	1.95
Acads	Mm00431617_m1	1	0.90	1.07	1.38	1.54	A_52_P367745	1	1.23	1.33	1.86	2.12
Acadvl	Mm00444296_m1	1	1.50	1.33	2.28	2.85	A_51_P518340	1	1.52	1.46	2.41	3.09
Acat1	Mm00507463_m1	1	0.95	0.92	1.07	1.19	A_51_P319449	1	1.42	1.13	1.30	1.45
Acat2	Mm00782408_s1	1	1.05	1.00	1.15	1.27	A_51_P139748	1	1.70	1.36	1.53	1.77
Acat3	Mm00462484_m1	1	6.64	1.16	0.80	1.25	A_51_P139745	1	1.64	1.32	1.56	1.74
Acly-	Mm00652520_m1	1	2.28	0.86	1.42	1.48	A_51_P261718	1	6.23	2.07	2.90	3.13
Aco1	Mm00801417_m1	1	1.28	1.17	1.40	1.61	A_52_P299115	1	1.95	1.17	1.53	1.83
Aco2	Mm00475673_g1	1	1.19	0.99	1.58	1.92	A_52_P200359	1	1.38	1.18	1.66	1.98
Acox1	Mm00443579_m1	1	0.90	1.13	1.67	1.98	A_51_P366704	1	1.31	1.44	2.06	2.29
Acsl1	Mm00484217_m1	1	0.71	1.45	2.13	2.25	A_51_P496432	1	0.72	1.46	1.80	1.95
Acsl6	Mm00522786_m1	1	0.48	0.64	0.60	0.66	A_51_P380699	1	0.76	0.48	0.69	0.54
Adh1	Mm00507711_m1	1	0.30	0.42	0.26	0.41	A_52_P629895	1	0.49	0.52	0.40	0.61
Agpat1	Mm00479700_m1	1	0.89	1.09	1.38	1.46	A_52_P200465	1	1.65	1.15	1.33	1.52
Agpat2	Mm00458880_m1	1	1.11	1.43	2.27	2.33	A_51_P238565	1	1.40	1.29	1.82	2.07
Agpat3	Mm00474003_m1	1	1.12	1.22	1.51	2.01	A_51_P425490	1	1.81	1.31	1.65	2.07
Agpat4	Mm00509776_m1	1	0.48	0.80	0.79	0.68	A_51_P346165	1	0.66	0.86	0.80	0.76
Agpat6	Mm00497622_m1	1	1.09	1.15	1.43	1.68	A_51_P335710	1	1.16	1.07	1.31	1.50
Aldh1a1	Mm00657317_m1	1	0.28	0.46	0.40	0.39	A_51_P334942	1	0.59	0.68	0.63	0.52
Aldh1a7	Mm00496380_m1	1	1.02	1.25	1.67	1.72	A_51_P383399	1	1.48	1.41	1.85	1.93
Aldh1b1	Mm00728303_s1	1	0.09	0.23	0.19	0.18	A_51_P510418	1	0.26	0.37	0.41	0.38
Aldh4a1	Mm00615268_m1	1	1.52	1.14	1.23	1.18	A_51_P337918	1	2.20	1.32	1.45	1.45
Aldh5a1	Mm00553115_m1	1	1.07	1.03	1.12	1.10	A_51_P491504	1	1.60	1.33	1.65	1.69
Aldh7a1	Mm00519645_m1	1	0.89	0.82	0.83	0.76	A_51_P470414	1	1.36	1.23	1.28	1.52
Aldob	Mm00523296_m1	1	0.10	0.62	0.33	0.94	A_51_P337269	1	0.08	0.54	0.46	0.74
Aldoc	Mm01298116_g1	1	0.06	0.53	0.16	0.44	A_51_P220681	1	0.29	0.50	0.40	0.64
Apob	Mm01545156_m1	1	0.32	0.57	0.50	1.36	A_51_P470542	1	0.16	0.57	0.46	0.82
Apoc1	Mm00431816_m1	1	2.51	1.80	2.05	2.33	A_51_P164504	1	3.30	1.78	1.84	2.20
Atp12a	Mm00446786_m1	1	0.00	0.18	0.09	0.26	A_52_P329398	1	0.11	0.29	0.14	0.47
Atp5b	Mm00443967_g1	1	0.97	0.88	1.15	1.25	A_52_P553841	1	1.31	1.21	1.62	1.81
Atp5c1	Mm00662408_m1	1	1.20	0.85	1.03	1.11	A_51_P378087	1	1.52	1.06	1.31	1.36
Atp5k	Mm00833200_g1	1	1.35	0.99	1.37	1.41	A_51_P301289	1	2.08	1.25	1.68	1.89
Atp6v0a1	Mm00444210_m1	1	0.30	1.09	1.26	1.33	A_51_P142089	1	0.50	1.00	1.16	1.32
Atp6v1c2	Mm00505047_m1	1	0.31	3.33	0.93	3.04	A_51_P518600	1	0.56	1.05	0.96	1.88
Atp6v1h	Mm00505548_m1	1	0.54	0.83	0.84	0.82	A_51_P105604	1	0.95	1.13	1.25	1.30
Bdh1	Mm00558330_m1	1	0.18	0.28	0.12	0.30	A_51_P163106	1	0.27	0.22	0.24	0.38

Cox6b1	Mm00824357_m1	1	1.10	1.11	1.51	1.62	A_51_P448032	1	1.54	1.39	1.82	2.16
Cox6b2	Mm00839225_g1	1	ND	ND	ND	ND	A_51_P300506	1	1.35	1.08	1.37	1.59
Cox7a1	Mm00438296_m1	1	1.04	0.66	1.33	1.84	A_51_P148612	1	1.37	0.66	1.45	1.98
Cox7b	Mm00835076_g1	1	0.99	0.92	1.31	1.47	A_51_P160664	1	1.96	1.33	1.87	2.15
Cpt1b	Mm00487200_m1	1	0.71	1.47	3.20	4.58	A_52_P625508	1	0.92	1.32	2.31	3.25
Cpt2	Mm00487202_m1	1	1.40	1.39	1.93	2.21	A_51_P403388	1	1.66	1.42	1.66	2.02
Cs	Mm00466043_m1	1	1.02	1.12	1.59	1.69	A_51_P390260	1	1.81	1.65	2.21	2.67
Cyp2a12	Mm00504878_m1	1	0.24	0.67	0.28	1.14	A_51_P206736	1	0.73	1.02	0.66	2.50
Cyp2a4;Cyp2a5	Mm00487248_g1	1	0.14	0.76	0.21	0.75	A_52_P246252	1	0.17	0.51	0.35	1.04
Cyp2c29	Mm00725580_s1	1	0.36	0.36	0.12	0.37	A_51_P103706	1	0.08	0.18	0.17	0.27
Cyp2c39	Mm00656110_gH	1	0.20	3.26	1.24	2.56	A_51_P304109	1	0.09	0.12	0.12	0.15
Cyp2c55	Mm00472168_m1	1	ND	ND	ND	ND	A_51_P447785	1	0.21	0.19	0.17	0.15
Cyp2d10	Mm00731648_m1	1	0.04	0.08	0.07	0.19	A_51_P111192	1	0.46	0.57	0.54	0.60
Cyp2d9	Mm00651731_m1	1	0.24	0.17	0.08	0.27	A_51_P124133	1	0.43	0.52	0.49	0.50
Cyp2e1	Mm00491127_m1	1	6.88	0.68	1.83	1.36	A_51_P283456	1	8.01	0.95	1.69	1.53
Cyp2j5	Mm00487292_m1	1	0.11	1.23	0.54	2.11	A_52_P315766	1	0.28	1.06	0.63	2.09
Cyp2s1	Mm00512037_m1	1	0.03	0.25	0.08	0.15	A_51_P180091	1	0.06	0.25	0.18	0.31
Cyp3a13	Mm00484110_m1	1	0.04	0.11	0.02	0.04	A_51_P114941	1	0.06	0.08	0.05	0.08
Cyp3a16	Mm00655824_m1	1	ND	ND	ND	ND	A_51_P482051	1	0.69	1.07	1.34	1.85
Cyp4a10	Mm02601690_gH	1	0.03	0.93	0.61	1.65	A_52_P257774	1	0.64	1.11	1.32	1.99
Cyp4a12a	Mm00514494_m1	1	0.13	1.53	1.04	9.16	A_51_P433360	1	0.53	1.03	1.37	3.72
Cyp4a14	Mm00484132_m1	1	0.02	1.05	0.76	2.24	A_51_P238576	1	0.43	1.12	1.22	1.80
Cyp4b1	Mm00484138_m1	1	0.30	0.76	0.95	1.31	A_51_P118704	1	0.52	1.06	1.37	1.76
Cyp4f14	Mm00491623_m1	1	0.19	0.06	0.06	0.03	A_51_P452768	1	0.32	0.17	0.14	0.14
Cyp51	Mm00490968_m1	1	0.29	0.87	0.81	0.74	A_52_P636752	1	0.50	1.32	1.25	1.26
Dci	Mm00494452_m1	1	1.02	0.95	1.21	1.38	A_51_P105589	1	1.51	1.28	1.68	1.85
Decr1	Mm00470689_m1	1	0.93	1.18	1.67	1.88	A_51_P208555	1	1.32	1.49	2.14	2.25
Dgat1	Mm01197412_g1	1	0.95	1.28	1.87	2.01	A_51_P510059	1	1.31	1.18	1.69	1.77
Dlat	Mm00455160_m1	1	0.97	1.24	1.85	2.22	A_51_P265106	1	1.77	1.49	2.10	2.72
Dld	Mm00432831_m1	1	1.04	1.06	1.43	1.60	A_51_P184284	1	1.36	1.33	1.88	2.07
Dlst	Mm00513470_m1	1	0.98	0.97	1.36	1.54	A_51_P290139	1	1.43	1.16	1.63	1.83
Echs1	Mm00659670_g1	1	1.15	0.95	1.12	1.23	A_51_P409039	1	1.75	1.20	1.43	1.61
Eno2	Mm00469062_m1	1	0.43	0.64	0.45	0.40	A_52_P748882	1	0.67	0.86	0.79	0.64
Eno3	Mm00468267_m1	1	0.99	0.38	0.34	0.37	A_51_P328539	1	1.58	0.41	0.54	0.62
Fabp2	Mm00433188_m1	1	0.02	0.12	0.07	0.60	A_51_P313581	1	0.52	0.57	0.58	0.68
Fabp3	Mm02342495_m1	1	0.32	0.63	1.10	1.97	A_51_P167535	1	0.60	0.62	1.20	1.82
Fabp4	Mm00445880_m1	1	0.70	0.97	1.25	1.33	A_51_P336830	1	0.82	0.97	1.32	1.04
Fasn	Mm00662322_g1	1	9.15	2.94	4.30	4.17	A_52_P100252	1	7.31	2.49	3.18	3.55
Fbp1	Mm00490181_m1	1	0.34	0.51	0.24	0.60	A_51_P474701	1	0.19	0.62	0.50	0.81
Fh1	Mm00802588_m1	1	0.85	0.88	1.06	1.15	A_51_P359333	1	1.56	1.28	1.63	1.87
Fntb	Mm00521491_m1	1	0.38	0.81	0.78	0.65	A_51_P517680	1	1.48	1.43	1.55	1.49
G6pd2	Mm00658204_s1	1	0.29	0.37	0.08	0.24	A_51_P353735	1	0.52	1.04	1.34	1.26
Gpd1	Mm00515846_m1	1	2.09	2.89	5.40	6.36	A_51_P293853	1	2.11	2.37	4.02	4.86
Gyk	Mm00433896_m1	1	0.43	1.73	3.30	4.43	A_51_P297671	1	0.53	2.32	3.93	5.33
Gys1	Mm00472712_m1	1	1.17	0.93	1.20	1.24	A_52_P228932	1	1.67	1.09	1.23	1.38
Gys2	Mm00523953_m1	1	2.92	4.16	8.08	9.97	A_51_P440315	1	4.32	4.01	7.11	9.53
Hadha	Mm00805228_m1	1	0.89	1.13	1.75	2.10	A_52_P193029	1	1.27	1.44	1.93	2.43
Hadhb	Mm00523880_g1	1	1.05	1.32	2.03	2.37	A_51_P217990	1	1.19	1.38	1.99	2.41
Hk1	Mm00439344_m1	1	0.37	0.56	0.44	0.47	A_51_P249024	1	1.10	0.97	1.04	1.02
Hmgcr	Mm01282499_m1	1	0.20	1.20	0.53	1.28	A_52_P578922	1	0.57	1.20	0.77	1.53
Hmgcs1	Mm00524111_m1	1	0.54	0.96	1.04	1.14	A_51_P146941	1	0.65	1.27	1.34	1.63

Hmgcs2	Mm00550050_m1	1	0.21	0.42	0.22	0.45	A_51_P116039	1	0.70	0.51	0.44	0.56
Hsd17b4	Mm00500443_m1	1	1.06	1.36	2.07	2.13	A_51_P445662	1	0.87	1.23	1.45	1.51
Idh1	Mm00516030_m1	1	0.68	0.92	1.00	1.06	A_51_P132978	1	1.30	1.20	1.44	1.59
Idh3b	Mm00504589_m1	1	1.10	1.00	1.48	1.74	A_51_P130110	1	1.49	1.18	1.73	1.91
Idh3g	Mm00599689_m1	1	1.15	0.90	1.18	1.35	A_51_P136729	1	1.67	1.18	1.59	1.91
Idi1	Mm00836417_g1	1	0.35	2.11	0.65	2.47	A_52_P441634	1	0.66	1.34	1.18	2.59
Ldhb	Mm00493146_m1	1	1.01	0.77	1.02	1.41	A_51_P409173	1	1.76	1.07	1.36	1.81
Mdh1	Mm00485106_m1	1	1.11	1.11	1.54	1.78	A_51_P218179	1	1.52	1.40	1.96	2.30
Mgll	Mm00449274_m1	1	0.51	1.28	1.68	1.91	A_52_P568977	1	0.71	1.48	1.75	2.28
Mut	Mm00485312_m1	1	1.13	0.90	1.03	0.99	A_52_P633163	1	2.08	1.23	1.36	1.41
Mvd	Mm00507014_m1	1	0.60	1.07	0.97	1.14	A_51_P355943	1	0.74	1.37	1.28	1.48
Ndufa1	Mm00444593_m1	1	0.88	0.82	0.99	1.07	A_51_P472405	1	1.44	1.13	1.42	1.52
Pcca	Mm00454899_m1	1	1.37	0.96	1.11	1.06	A_51_P484254	1	2.34	1.41	1.78	1.72
Pcx	Mm00500992_m1	1	1.97	1.26	1.58	1.53	A_51_P480578	1	2.53	1.67	1.95	2.20
Pdha1	Mm00468675_m1	1	1.65	0.92	1.18	1.33	A_51_P321391	1	2.92	1.46	2.15	2.54
Pdhb	Mm00499323_m1	1	1.80	1.44	2.28	2.71	A_52_P568792	1	2.19	1.69	2.49	3.05
Pdk4	Mm00443325_m1	1	0.65	0.54	0.58	0.98	A_51_P350453	1	0.72	0.49	0.67	0.86
Pgam2	Mm00450782_g1	1	1.09	0.44	0.42	0.40	A_51_P264495	1	1.99	0.53	0.58	0.69
Pgk1	Mm01225301_m1	1	0.79	0.74	0.84	0.96	A_51_P182828	1	1.32	0.97	1.20	1.34
Pgk2	Mm00446208_s1	1	0.51	0.46	0.22	1.46	A_51_P125487	1	1.57	0.68	0.49	0.77
Pgm1	Mm00804141_m1	1	0.51	0.77	0.72	0.69	A_51_P427530	1	0.61	0.80	0.75	0.68
Pgm5	Mm00723432_m1	1	0.30	0.44	0.37	0.35	A_52_P860000	1	0.28	0.31	0.39	0.32
Pmvk	Mm00503429_m1	1	0.83	1.51	1.66	1.89	A_51_P492408	1	1.21	1.39	1.53	1.65
Ppara	Mm00440939_m1	1	1.50	1.26	1.84	2.77	A_51_P348334	1	2.40	1.60	2.16	3.17
Ppard	Mm00803186_g1	1	0.79	0.97	0.84	0.73	A_51_P271556	1	0.87	1.05	1.07	1.17
Pparg	Mm00440945_m1	1	0.85	1.08	1.15	1.17	A_51_P106799	1	1.31	1.12	1.10	1.06
Ppargc1a	Mm01208832_m1	1	1.25	0.56	0.94	1.34	A_51_P279038	1	2.61	0.80	1.13	1.65
Ppargc1b	Mm01258518_m1	1	3.89	0.75	1.12	1.59	A_51_P294891	1	1.63	0.95	1.37	1.83
Ppt2	Mm00450833_m1	1	0.97	1.15	1.36	1.48	A_52_P238816	1	1.43	1.19	1.41	1.54
Pygl	Mm00500078_m1	1	2.14	1.30	1.54	1.49	A_51_P452779	1	2.46	1.23	1.42	1.46
Pygm	Mm00478582_m1	1	0.99	0.49	0.48	0.51	A_51_P458973	1	1.51	0.46	0.46	0.49
Sdha	Mm01352360_m1	1	1.16	0.83	1.05	1.19	A_51_P410823	1	1.68	1.02	1.42	1.56
Sdhb	Mm00458268_m1	1	1.15	0.90	1.27	1.48	A_51_P234853	1	1.60	1.21	1.67	1.97
Sdhc	Mm00481172_m1	1	0.84	0.85	1.02	1.06	A_51_P106373	1	1.32	1.10	1.35	1.48
Slc27a1	Mm00449511_m1	1	0.50	1.21	1.99	2.33	A_51_P117477	1	0.75	1.04	1.39	1.58
Slc27a2	Mm00449517_m1	1	0.96	1.24	1.17	4.26	A_51_P484551	1	1.53	0.89	1.00	1.76
Slc27a4	Mm01327414_g1	1	0.72	1.41	1.46	1.84	A_52_P61854	1	0.83	1.20	1.43	1.67
Slc2a2	Mm00446224_m1	1	0.24	0.63	0.47	0.74	A_51_P185693	1	0.30	0.96	0.65	0.98
Slc2a4	Mm00436615_m1	1	1.46	0.82	1.05	1.10	A_51_P217498	1	2.17	1.08	1.41	1.53
Sqle	Mm00436772_m1	1	0.38	1.00	0.83	0.84	A_51_P450487	1	0.51	0.87	0.75	0.73
Taldo1	Mm00807080_g1	1	1.53	1.42	1.91	2.03	A_51_P401958	1	1.53	1.36	1.61	1.72
Tkt	Mm00447559_m1	1	4.12	1.57	2.05	2.05	A_51_P394515	1	4.05	1.51	1.86	2.04
Uqcrb	Mm00835346_gH	1	0.77	0.75	1.02	1.13	A_51_P472671	1	1.81	1.40	1.82	2.14
Uqcrfs1	Mm00481849_m1	1	1.27	1.00	1.44	1.67	A_51_P361951	1	1.57	1.24	1.87	2.25
Uqcrh	Mm00835199_g1	1	0.99	0.97	1.29	1.47	A_52_P541875	1	1.89	1.43	1.81	1.97

Supplemental Table 4: Common sequence predictors for liver trioleate concentrations and weight, determined using PLS analysis. Removing redundancy among the 20 predictors led to 16 genes from which 13 displayed PPRE in their proximal promoters as determined by MatInspector from Genomatix suit. Publication-based PPRE are indicated in Bibliography PMID column: PubMed identification number; F.C. Fold Change; *p*-value= 0 correspond to *p*-value<1E-45.

Sequence name	Sequence code	Sequence description	Predicted PPRE	Bibliography PMID	db/+ Foie	db/+ Foie	RGZ 10MK	RGZ 10MK	RGZ 30MK	RGZ 30MK	RGZ 100MK	RGZ 100MK
					F. C.	P-Value	F. C.	P-Value	F. C.	P-Value	F. C.	P-Value
Aldob	A_51_P337269	aldolase 2, B isoform	X	15226431	-1.70	6.33E-13	-1.02	0.813021	-1.20	0.002103	-1.45	9.81E-11
Cyp2a5	A_52_P246252	cytochrome P450, family 2, subfamily a, polypeptide 5		10454501	-2.1	1.97E-08	-5.00	0	-4.00	0	-2.85	1.82E-09
Cyp2c29	A_51_P103706	cytochrome P450, family 2, subfamily c, polypeptide 29	X	12130701, 10454501	-1.04	0.072755	-3.30	0	-2.7	0	-2.17	7.98E-23
Cyp2c37	A_52_P625249	cytochrome P450, family 2. subfamily c, polypeptide 37	X		-1.53	8.30E-30	-4.50	0	-3.33	0	-2.70	8.70E-34
Cyp2c37	A_51_P498882	cytochrome P450, family 2. subfamily c, polypeptide 37			-1.45	2.30E-20	-4.34	0	-3.12	0	-2.56	4.71E-28
Cyp2c39	A_51_P304109	cytochrome P450, family 2, subfamily c, polypeptide 39	X		-1.37	5.65E-16	-2.94	0	-2.5	0	-2.12	1.38E-35
Cyp2c54	A_52_P154580	cytochrome P450, family 2, subfamily c, polypeptide 54	X		-1.72	3.52E-29	-5.26	0	-3.57	0	-2.78	1.26E-32
Cyp2j6	A_51_P506328	cytochrome P450, family 2, subfamily j, polypeptide 6	X		1.17	7.20E-14	1.41	3.60E-21	1.36	5.73E-14	1.31	3.75E-10
Fabp3	A_51_P167535	fatty acid binding protein 3, muscle and heart	X	15491498, 15130092	-1.79	1.14E-33	2.71	0	2.38	8.80E-24	1.82	2.40E-07
Fb p1	A_51_P474701	fructose bisphosphatase 1	X	15226431, 12072378	-1.99	6.44E-18	-1.36	0.000066	-1.56	7.07E-10	-1.92	3.16E-18
Fh1	A_51_P359333	fumarate hydratase 1	X		-1.31	3.03E-16	-1.06	0.005397	-1.08	0.002433	-1.14	1.30E-17
Gpi1	A_51_P426886	glucose phosphate isomerase 1	X		-1.09	4.96E-09	1.43	0	1.33	4.02E-25	1.26	4.93E-30
Hadhb	A_51_P217990	hydroxyacyl-Coenzyme A dehydrogenase/3-ketoacyl-Coenzyme A thiolase/enoyl-Coenzyme A hydratase, beta subunit		19300518, 11181544	-1.06	0.03782	1.53	0	1.39	2.69E-31	1.32	2.82E-11
Oxct1	A_52_P430058	3-oxoacid CoA transferase 1	X		1.19	0.000017	1.33	1.70E-08	1.37	2.19E-07	1.50	1.73E-06
Oxct1	A_51_P107321	3-oxoacid CoA transferase 1			1.30	7.23E-10	1.38	1.86E-11	1.29	2.77E-14	1.40	7.08E-11
Phkb	A_52_P513347	phosphorylase kinase beta	X		1.07	0.140703	1.45	5.61E-45	1.47	1.77E-25	1.38	2.37E-19
